# Supplementary material for: Enantioselective alkylative cross-coupling of unactivated aromatic C–O electrophiles
Source: Nat Commun. 2022 May 26;13:2953. doi: 10.1038/s41467-022-30693-x (PMC9135759; doi:10.1038/s41467-022-30693-x)
Supplement: Supplementary file 1 — Supplementary Information [file 41467_2022_30693_MOESM1_ESM.pdf]

Supplementary Information:

Enantioselective Alkylative Cross-Coupling of Unactivated Aromatic C-O Electrophiles

Zishuo Zhang, [1,#] Jintong Zhang, [1,#] Quan Gao, [1,#] Yu Zhou,[2] Mingyu Yang,[3] Haiqun Cao, [1] Tingting Sun,[1] Gen Luo,[2,*] Zhi-Chao Cao[1,*]

[1] Anhui Province Engineering Laboratory for Green Pesticide Development and Application, College of Plant Protection, Anhui Agricultural University, Hefei, Anhui, China, 230036;

[2] Institute of Physical Science and Information Technology, Anhui University, Hefei 230601, China

[3] School of Chemistry & Chemical Engineering, Shaanxi Normal University, Xi'an, Shaanxi, China, 710119

[#]These authors contributed equally to this work.

*Email: luogen@ahu.edu.cn (G.L.); zc_cao@ahau.edu.cn (Z.C.C.).

Table of Contents

**1**	Supplementary notes	2
**2**	Supplementary figures of reported routes for the synthesis of MeOBIN	3
**3**	Supplementary figures of reported MeOBIN-derived catalysts and ligands	4
**4**	Supplementary methods and analytic data	6
**5**	Supplementary figures of determination of absolute stereochemistry	24
**6**	Supplementary tables of mechanistic investigation	27
**7**	Supplementary figures of computational details	28
**8**	Supplementary figures of NMR spectra of products	31
**9**	Supplementary figures of HPLC trace	70
**10**	Supplementary references	109

1. Supplementary notes

Unless otherwise noted, reagents received from commercial suppliers were used as received. Chiral phosphine ligands, including (*R*)-BINAP, (*R*)-Tol-BINAP, and (*S*)-^{i}Pr-Phox, were purchased from Innochem and used without further purification. Anhydrous toluene was distilled by using sodium with diphenyl ketone as the indicator. Other solvents, such as MTBE (methyl *tert*-butyl ether), CPME, THF and $^{i}Pr_2O$ were purchased from Adamas and stored under nitrogen.

Unless otherwise noted, all reactions were performed under an atmosphere of dry nitrogen. Flash column chromatography was performed by using silica gel (400 meshes). NMR spectra were collected on an Agilent 600 MHz or Bruke 400 MHz at ambient temperature; chemical shifts (δ) are reported in ppm downfield from tertramethylsilane (TMS), using the resonance of TMS as the internal standard.

HPLC analysis were carried out on Shimadzu Essentia LC-16 series and Waters 2998 series (2487 Dual λ Absorbance Detector and 1525 Binary HPLC Pump, UV detection monitored at 254 nm) with Daicel CHIRALPAK® or Daicel CHIRALCEL® columns (4.6 × 250 mm, particle size 5 μm). HRMS data were acquired on a Bruker Avance Mass Spectrometer (maXis, ESI or APCI). Optical-rotation data were obtained with an Anton Paar MCP-100 polarimeter at 589 nm, using a 100 mm pathlength cell in the solvent and at the concentration indicated.

2. Supplementary figures of reported routes for the synthesis of MeOBIN

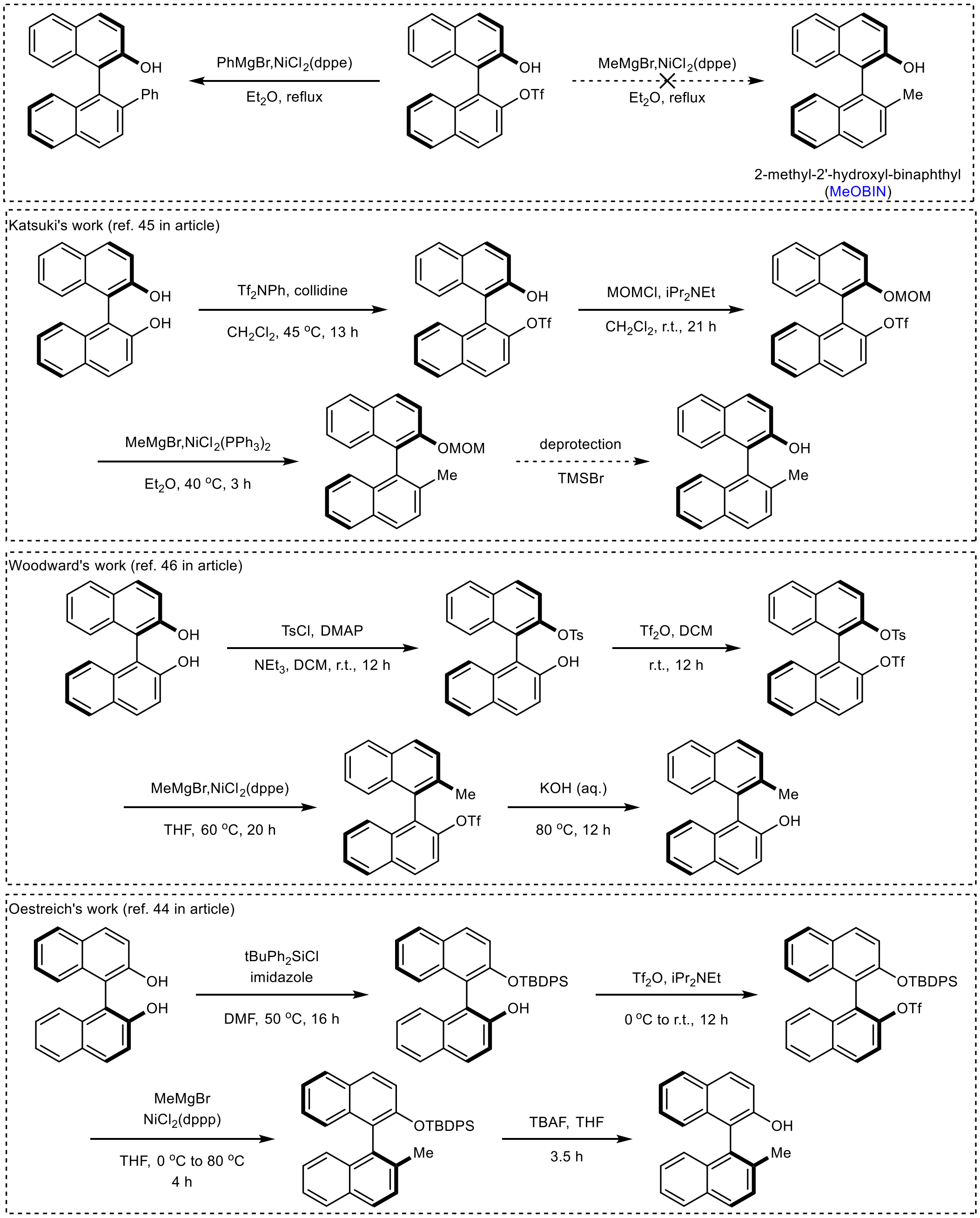

Supplementary Figure 1. Reported methods for the synthesis of 2-methyl-2′-hydroxyl binaphthyl compound.

3. Supplementary figures of reported MeOBIN-derived catalysts and ligands

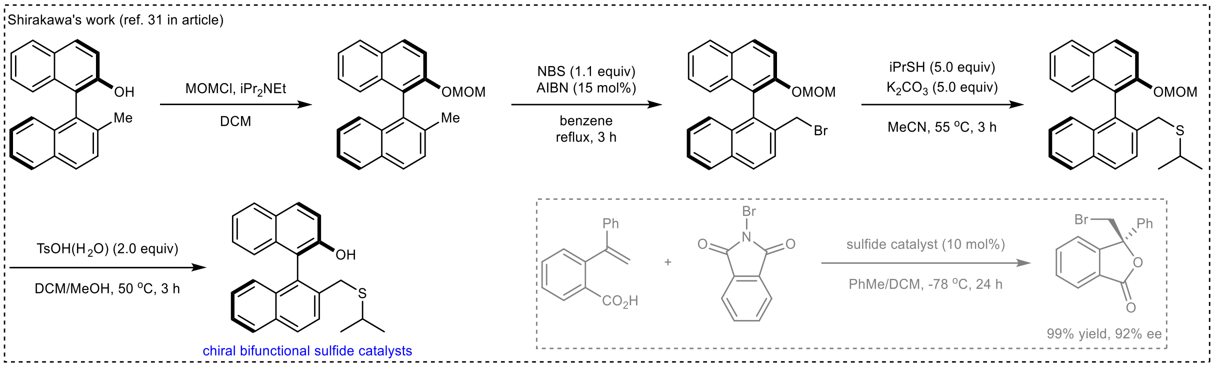

Supplementary Figure 2. Synthesis of bifunctional sulfide catalyst and its application in asymmetric synthesis of 3,3-disubstituted phthalides via bromolactonization

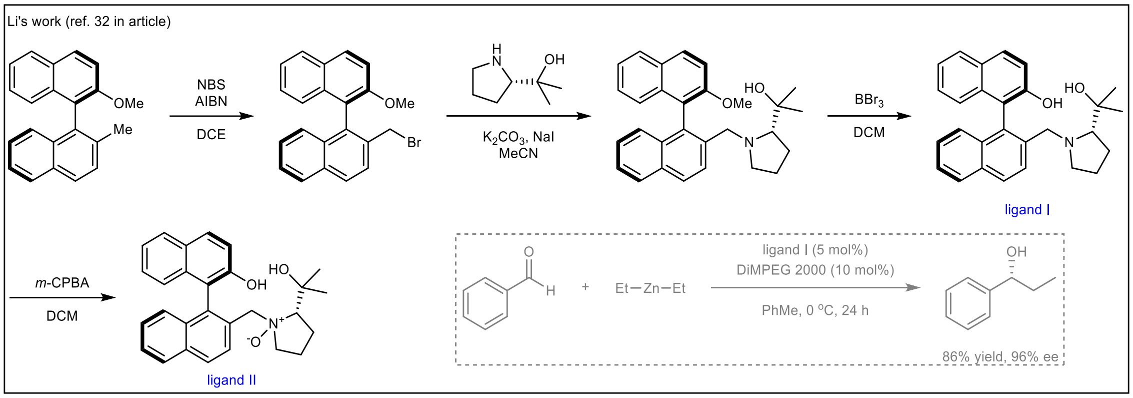

Supplementary Figure 3. Synthesis and application of binaphthyl-based chiral ligands

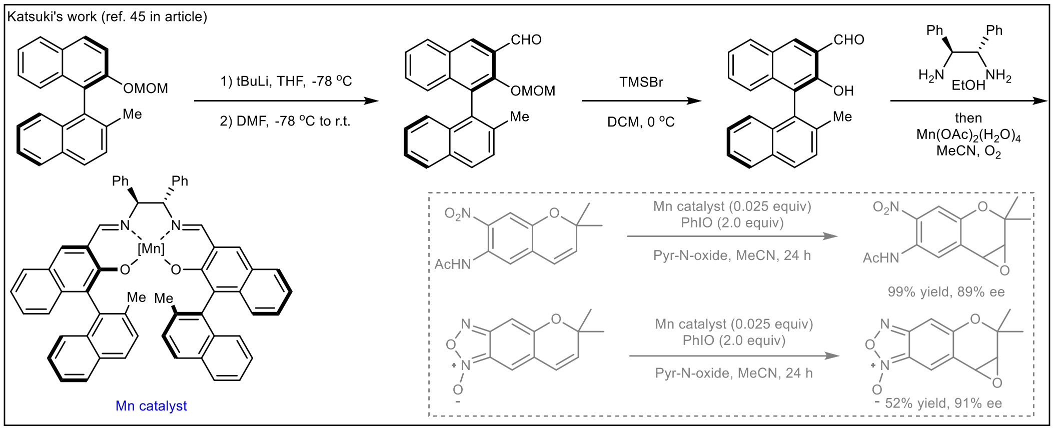

Supplementary Figure 4. Synthesis and application of Mn-Salen epoxidation catalyst

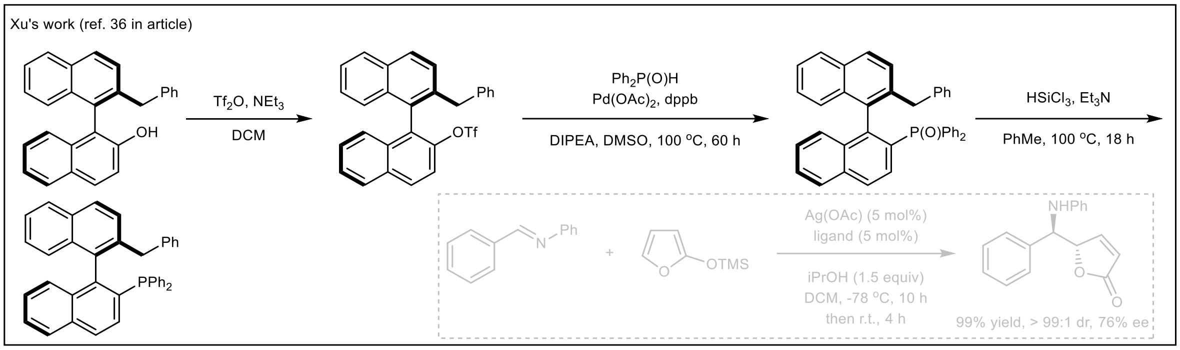

Supplementary Figure 5. Synthesis of monophosphine and its application in asymmetric Mannich reaction

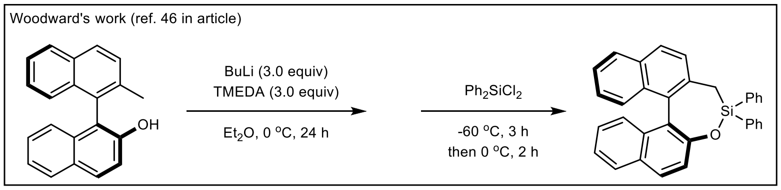

Supplementary Figure 6. Synthesis of axially chiral silane

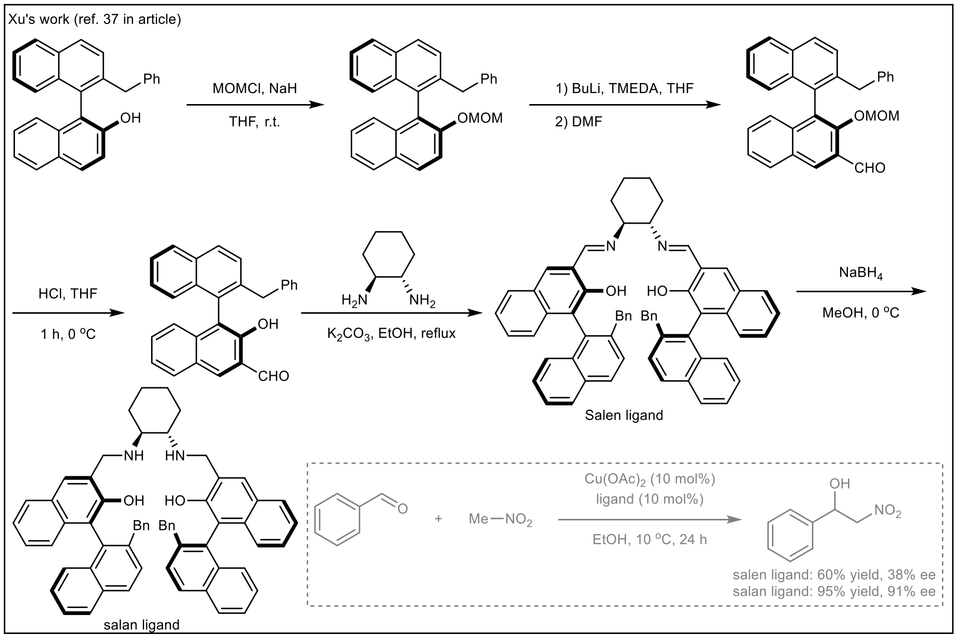

Supplementary Figure 7. Synthesis of chiral Cu-salan catalyst and its application in asymmetric Henry transformation of aromatic aldehyde

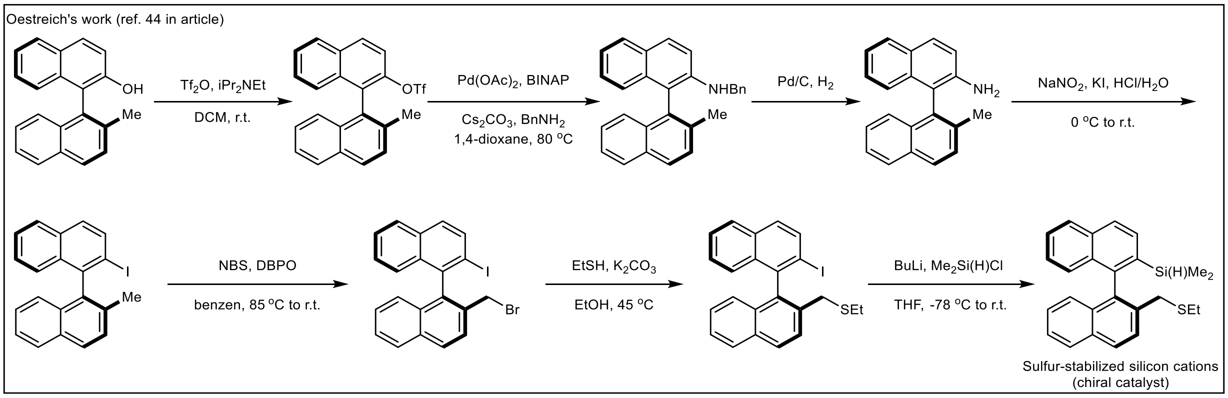

Supplementary Figure 8. Synthesis of intramolecularly sulfur-stabilized silicon cations with chiral binaphthyl backbones

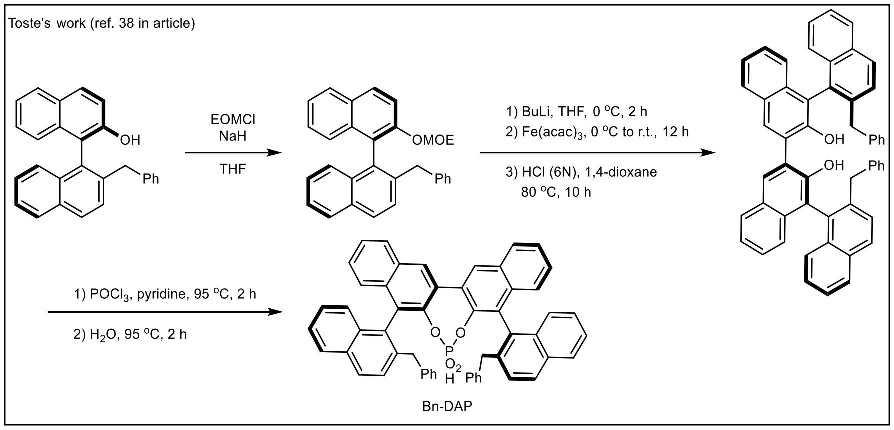

Supplementary Figure 9. Synthesis of doubly axially chiral phosphoric acid (Bn-DAP catalyst)

4. Supplementary methods and analytic data

4.1 Derivation experiments

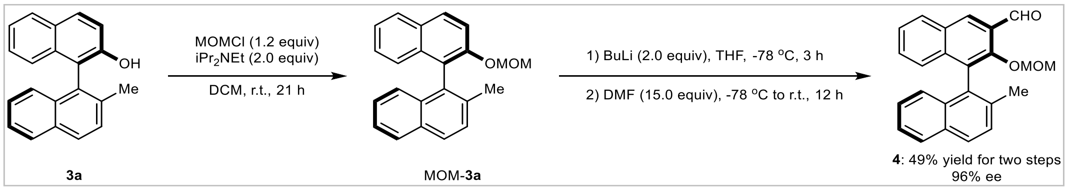

Supplementary Figure 10. Preparation of aldehyde **4** via *sp*^{2} C-H modification

To a solution of compound **3a** (1.0 equiv) in dichloromethane (0.2 M) were added *N,N*-diisopropylethylamine (^{i}Pr$_2$NEt) (2.0 equiv) and chloromethyl methyl ether (MOMCl) (1.2 equiv). After stirring for 21 hours at room temperature, the reaction mixture was quenched with water and extracted with dichloromethane. The organic layer was dried over Na_2SO_4, concentrated, and

chromatographed on silica gel to give compound MOM-**3a**. Furthermore, to a THF solution of compound MOM-**3a** (1.0 equiv) was added butyllithium (BuLi) (2.0 equiv) at -78 °C and the mixture was stirred at the same temperature for 3 hours. DMF (15.0 equiv) was then added and the mixture was allowed to warm to rt. After stirring for 12 hours, the reaction mixture was quenched with sat. aqueous NH_4Cl, extracted with ether, washed with sat. aqueous $NaHCO_3$ and brine, dried over Na_2SO_4, concentrated, and chromatographed on silica gel to give the corresponding aldehyde **4** in 49% yield as white solid. Optical Rotation: $[\alpha]^{20}{}_D$ = -61.9 (c 0.21, CH_2Cl_2). 96% ee, determined by HPLC, HPLC conditions: CHIRALPAK® IA, 10% i-PrOH in hexane, 1.00 mL min^{-1}, λ = 254 nm, τ_R (major) = 5.3 min; τ_R (minor) = 5.7 min. ^{1}H NMR (600 MHz, cdcl$_3$) δ 10.58 (s, 1H), 8.57 (s, 1H), 8.05 (d, *J* = 8.3 Hz, 1H), 7.90 (t, *J* = 8.9 Hz, 2H), 7.52 (d, *J* = 8.4 Hz, 1H), 7.49 – 7.45 (m, 1H), 7.44 – 7.39 (m, 1H), 7.35 (t, *J* = 7.5 Hz, 1H), 7.27 (d, *J* = 8.6 Hz, 1H), 7.16 (d, *J* = 8.5 Hz, 1H), 7.12 (d, *J* = 8.6 Hz, 1H), 4.65 – 4.56 (m, 2H), 2.91 (s, 3H), 2.16 (s, 3H). ^{13}C NMR (100 MHz, $CDCl_3$) δ 191.04, 152.95, 136.54, 135.68, 133.11, 132.02, 131.51, 130.91, 130.23, 130.16, 129.34, 128.95, 128.66, 128.32, 128.03, 126.41, 126.03, 125.87, 125.72, 125.06, 99.83, 56.97, 20.52. HRMS (ESI) m/z $[M+Na]^+$ calcd for $[C_{24}H_{20}NaO_3]^-$ 379.1305, found 373.1295.

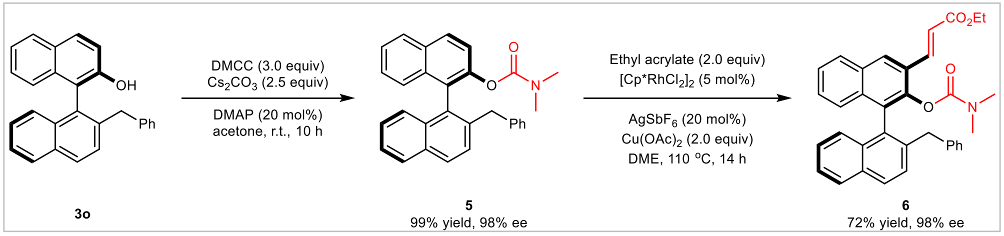

Supplementary Figure 11. Preparation of compound **6** via vinylation

Under argon atmosphere, to a mixture of **3o** (1.0 equiv), DMAP (20 mol%), and Cs_2CO_3 (2.5 equiv) in anhydrous acetone (0.1 M) was added dimethylcarbamoyl chloride (DMCC) (3.0 equiv). The reaction was stirred at room temperature for 10 hours. After filtration, the solvents were removed by evaporation under reduced pressure. The resulting residue was purified by column chromatography on silica gel to afford compound **5** in 99% yield as a white solid. Furthermore, under argon atmosphere, a mixture of compound **5** (1.0 equiv), $[Cp^*RhCl_2]_2$ (5 mol%), $AgSbF_6$ (20 mol%), $Cu(OAc)_2$ (2.0 equiv), and anhydrous DME (0.1 M) in a 25 mL sealed tube was heated at 110 °C for 14 h. After cooling to room temperature, the mixture was diluted with EtOAc, and filtered through a celite pad. The filter cake was washed with EtOAc and the filtrate was concentrated under reduced pressure. The remaining residue was purified by column chromatography on silica gel to afford compound **6** in 72% yield as a white solid.

Compound 5: Optical Rotation: $[\alpha]^{20}{}_D$ = 256.3 (c 0.32, CH_2Cl_2). 98% ee, determined by HPLC, HPLC conditions: CHIRALPAK® ID, 30% i-PrOH in hexane, 1.00 mL min^{-1}, λ = 254 nm, τ_R (major) = 9.3 min; τ_R (minor) = 16.1 min. ^{1}H NMR (600 MHz, cdcl$_3$) δ 7.99 (d, *J* = 9.0 Hz, 1H), 7.92 (d, *J* = 8.2 Hz, 1H), 7.84 (dd, *J* = 8.4, 3.0 Hz, 2H), 7.60 (d, *J* = 8.9 Hz, 1H), 7.44 – 7.36 (m, 3H), 7.23 (dd, *J* = 6.3, 3.5 Hz, 3H), 7.19 (d, *J* = 8.4 Hz, 1H), 7.15 – 7.00 (m, 3H), 6.93 (d, *J* = 6.9 Hz, 2H), 3.73 (s, 2H), 2.64 (s, 3H), 2.13 (s, 3H). ^{13}C NMR (151 MHz, cdcl$_3$) δ 154.10, 147.47, 140.63, 138.21, 133.45, 133.06, 132.12, 131.47, 131.44, 129.24, 128.94, 128.08, 128.05, 127.97, 127.63, 127.55, 126.91, 126.47, 126.44, 126.12, 125.88, 125.73, 125.32, 125.30, 122.60, 39.65, 36.27, 35.49. HRMS (APCI) m/z $[M+H]^+$ calcd for $[C_{30}H_{26}NO_2]^+$ 432.1958, found 432.1950.

Compound 6: Optical Rotation: $[\alpha]^{20}{}_D$ = 50 (c 0.72, CH_2Cl_2). 98% ee, determined by HPLC, HPLC conditions: CHIRALPAK® IA, 20% i-PrOH in hexane, 1.00 mL min^{-1}, λ = 254 nm, τ_R (major) = 7.2

min; τ_R (minor) = 11.5 min. ^{1}H NMR (600 MHz, cdcl$_3$) δ 8.29 (s, 1H), 7.97 – 7.91 (m, 2H), 7.85 (t, *J* = 7.8 Hz, 2H), 7.44 (ddd, *J* = 8.2, 6.7, 1.2 Hz, 1H), 7.39 (dq, *J* = 8.7, 4.5 Hz, 2H), 7.25 (d, *J* = 3.7 Hz, 3H), 7.16 (d, *J* = 8.4 Hz, 1H), 7.10 (dt, *J* = 14.1, 7.1 Hz, 3H), 6.95 (s, 2H), 6.67 (d, *J* = 15.9 Hz, 1H), 4.28 (q, *J* = 7.1 Hz, 2H), 3.88 – 3.60 (m, 2H), 2.49 (s, 3H), 2.09 (s, 3H), 1.35 (t, *J* = 7.1 Hz, 3H). ^{13}C NMR (100 MHz, CDCl$_3$) δ 166.86, 153.47, 146.22, 140.30, 139.70, 138.40, 134.10, 132.74, 132.04, 131.37, 131.19, 129.35, 129.17, 128.65, 128.59, 128.17, 128.06, 127.84, 127.53, 126.15, 126.10, 125.91, 125.80, 125.42, 120.30, 60.46, 39.62, 36.25, 35.46, 14.32. HRMS (APCI) m/z [M+H]$^+$ calcd for [$C_{35}H_{32}NO_4$]$^-$ 530.2326, found 530.2334.

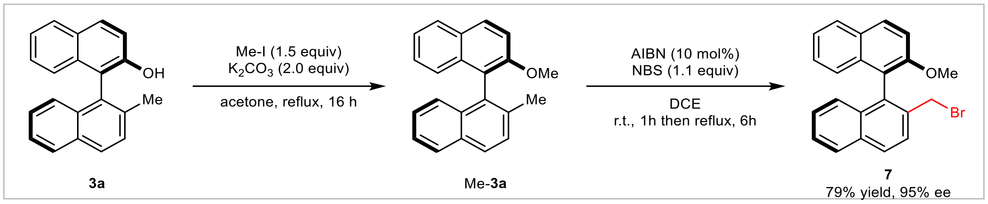

Supplementary Figure 12. Bromination by using *sp*^{3} C-H modifiable site

To a solution of compound **3a** (1.0 equiv) and potassium carbonate (1.5 equiv) in acetone (0.3 M) was added CH_3I (2.7 mL, 44 mmol) at room temperature, and the resulting mixture was stirred at 60 °C for 16 h. The reaction was monitored with TLC. After the completion of the reaction, the residue was washed with DCM (100 mL × 3). The combined organic layer was washed with saturated aqueous NaCl (100 mL), dried over MgSO4 and concentrated in vacuum. The crude product was purified by silica gel chromatography to give product Me-**3a** as white solid. To a solution of Me-**3a** (1.0 equiv) and AIBN (10 mol%) in DCE (0.2 M) was added NBS (1.1 equiv) in portions in a period of 1 hour at room temperature, and the resulting mixture was refluxed for another 6 hours. The reaction mixture was cooled to room temperature and was concentrated in vacuum. The residue was washed with DCM, the combined organic layer was washed with saturated aqueous NaCl, dried over $MgSO_4$ and concentrated in vacuum. The crude product was purified by silica gel chromatography to give product **7** as a white solid (79% yield). Optical Rotation: $[\alpha]^{20}{}_D$ = 26.7 (c 0.15, CH_2Cl_2). 95% ee, determined by HPLC, HPLC conditions: CHIRALPAK® OJ, 5% i-PrOH in hexane, 1.00 mL min^{-1}, λ = 254 nm, τ_R (minor) = 9.3 min; τ_R (major) = 10.3 min. ^{1}H NMR (600 MHz, cdcl$_3$) δ 8.01 (d, *J* = 9.1 Hz, 1H), 7.95 (d, *J* = 8.6 Hz, 1H), 7.88 (t, *J* = 7.3 Hz, 2H), 7.72 (d, *J* = 8.5 Hz, 1H), 7.48 – 7.39 (m, 2H), 7.32 (ddd, *J* = 8.1, 6.7, 1.2 Hz, 1H), 7.25 – 7.18 (m, 2H), 7.14 (d, *J* = 8.5 Hz, 1H), 6.97 (d, *J* = 8.5 Hz, 1H), 4.36 – 4.23 (m, 2H), 3.76 (s, 3H). ^{13}C NMR (101 MHz, CDCl$_3$) δ 154.79, 133.99, 133.81, 133.33, 132.97, 130.14, 128.94, 128.58, 128.02, 127.91, 127.63, 126.71, 126.59, 126.45, 126.26, 125.15, 123.74, 119.52, 113.39, 56.42, 32.69.; HRMS (ESI) m/z [M+Na]$^+$ calcd for [$C_{22}H_{17}BrNaO$]$^-$ 399.0355, found 399.0349.

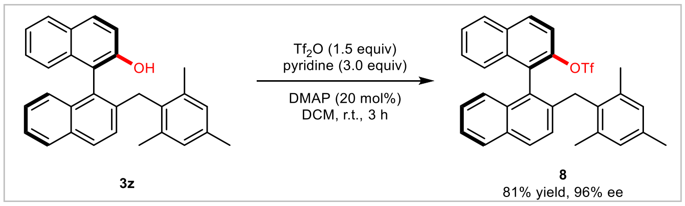

Supplementary Figure 13. Triflation of compound **3z**

Under argon atmosphere, to a solution of **3z** (1.0 equiv), DMAP (20 mol%), and pyridine (3.0 equiv) in anhydrous CH_2Cl_2 (0.1 M) was added Tf_2O (3.0 equiv) at rt. The reaction was stirred at r.t. for 3 hours. The mixture was quenched by addition of water, and extracted with CH_2Cl_2. The combined organic extracts were washed with brine, dried with anhydrous Na_2SO_4, filtrated, and evaporated under reduced pressure. The residue was purified by column chromatography on silica gel to afford compound **8** in 81% yield as a white solid. Optical Rotation: $[\alpha]^{20}{}_{D}$ = -73.3 (c 0.6, CH_2Cl_2). 96% ee, determined by HPLC, HPLC conditions: CHIRALPAK® IF, 10% i-PrOH in hexane, 1.00 mL min^{-1}, λ = 254 nm, τ_R (minor) = 4.3 min; τ_R (major) = 4.6 min. ^1H NMR (600 MHz, cdcl3) δ 8.09 (d, *J* = 9.0 Hz, 1H), 8.02 (d, *J* = 8.2 Hz, 1H), 7.85 (d, *J* = 8.2 Hz, 1H), 7.81 (d, *J* = 8.6 Hz, 1H), 7.64 (d, *J* = 9.0 Hz, 1H), 7.58 (t, *J* = 7.5 Hz, 1H), 7.44 – 7.36 (m, 2H), 7.33 (d, *J* = 8.5 Hz, 1H), 7.28 – 7.23 (m, 1H), 7.09 (d, *J* = 8.5 Hz, 1H), 6.92 (d, *J* = 8.6 Hz, 1H), 6.85 (s, 2H), 3.89 (d, *J* = 17.4 Hz, 1H), 3.45 (d, *J* = 17.5 Hz, 1H), 2.27 (s, 3H), 2.03 (s, 6H). ^{13}C NMR (100 MHz, $CDCl_3$) δ 145.00, 137.37, 137.00, 135.76, 133.13, 132.84, 132.71, 132.68, 132.15, 130.57, 129.46, 129.30, 128.83, 128.80, 128.41, 127.88, 127.75, 127.20, 126.60, 126.27, 125.64, 125.28, 124.77, 119.69, 32.81, 20.91, 19.90. HRMS (APCI) m/z $[M-H]^-$ calcd for $[C_{21}H_{16}O]^-$ 533.1401, found 533.1416.

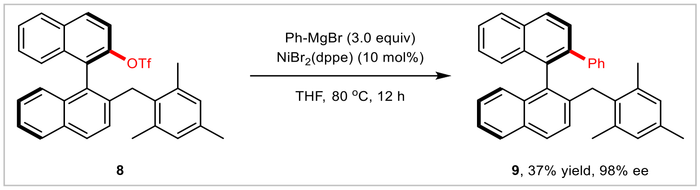

Supplementary Figure 14. Preparation of compound **9** via nickel catalyzed Kumada coupling of compound **8**

Under argon atmosphere, a mixture of compound **8** (1.0 equiv), NiBr2(dppe) (10 mol%), PhMgBr (5.0 equiv), and anhydrous Et_2O (0.1 M) in a 25 mL sealed tube was heated 80 oC for 12 hours. The mixture was cooled to room temperature. The reaction was quenched with HCl (1.0 M) in ice-water bath, and extracted with CH_2Cl_2 (20 mL x 2). The combined organic extracts were washed with brine, dried with anhydrous Na_2SO_4, filtrated, and evaporated under reduced pressure. The residue was purified by column chromatography on silica gel compound 9 in 37% yield as a white solid. Optical Rotation: $[\alpha]^{20}{}_{D}$ = 103.3 (c 0.3, CH_2Cl_2). 98% ee, determined by HPLC, HPLC conditions: CHIRALPAK® IA, 5% i-PrOH in hexane, 1.00 mL min^{-1}, λ = 254 nm, τ_R (major) = 3.7 min; τ_R (minor) = 4.0 min. ^1H NMR (600 MHz, cdcl3) δ 8.06 (d, *J* = 8.5 Hz, 1H), 7.98 (d, *J* = 8.2 Hz, 1H), 7.83 (d, *J* = 8.2 Hz, 1H), 7.74 (d, *J* = 8.5 Hz, 1H), 7.63 (d, *J* = 8.6 Hz, 1H), 7.48 (t, *J* = 6.8 Hz, 1H), 7.40 (t, *J* = 6.7 Hz, 1H), 7.33 (d, *J* = 8.4 Hz, 1H), 7.31 – 7.24 (m, 2H), 7.19 (d, *J* = 8.5 Hz, 1H), 7.10 (d, *J* = 6.9 Hz, 2H), 7.07 (d, *J* = 7.2 Hz, 1H), 7.03 (t, *J* = 7.2 Hz, 2H), 6.75 (s, 2H), 6.60 (d, *J* = 8.6 Hz, 1H), 3.40 – 3.21 (m, 2H), 2.22 (s, 3H), 1.67 (s, 6H). ^{13}C NMR (101 MHz, $CDCl_3$) δ 141.52, 139.22, 137.27, 135.70, 135.39, 134.92, 134.63, 134.21, 133.03, 133.01, 132.60, 131.72, 129.10, 128.57, 128.55, 128.28, 128.00, 127.83, 127.59, 126.61, 126.45, 126.40, 126.24, 125.91, 124.80, 124.67, 33.12, 20.87, 19.55. HRMS (ESI) m/z $[M+Na]^+$ calcd for $[C_{36}H_{30}Na]^-$ 485.2240, found 485.2233.

4.2 Synthesis of ligand L1: According to the reported procedure,[1,2] to a solution of chiral amine (2.0 equiv.) in AcOH (20 equiv.) heated to 60 °C, and then an aqueous solution of glyoxal (0.5 equiv., 40 wt.%, *c* = 8.7 M) and formaldehyde (0.5 equiv., 37 wt. %, *c* = 13.4 M) were added, the reaction was stirred at the same temperature for another 1 hour. Then the reaction was treated

with 10% HCl (aq.) (approximatively 25 mL for 200 mg of amine) and CH_2Cl_2 (approximatively 25 mL for 200 mg of amine). The bilayer system was vigorously stirred for 1 hour and then separated. The aqueous layer was extracted with CH_2Cl_2 (2x) and the combined organic layers dried (MgSO4), filtered, and the solvent was removed under reduced pressure. The resulting solid was dried under reduced pressure overnight, yielding the target compound in 78% yield. The characterized data is consistent with the reported in the literature.

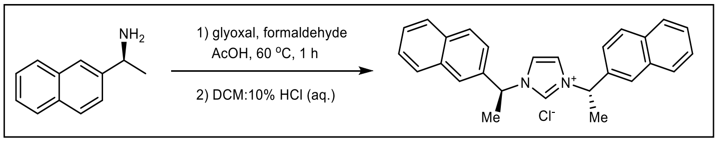

Supplementary Figure 15. Synthesis of chiral ligand

4.3 Synthesis of substrate 1a: According to the reported method,[3,4] to a flame-dried round bottom 500 mL flask were added racemic 1,1′-binaphthol (1.0 equiv.), *p*-TsOH 1.0 equiv.) and toluene (300 mL). The reaction mixture was refluxed at 140 °C for 36 hours. The solvent was then removed to give the crude product which was purified on silica gel by flash column chromatography (5% DCM in petroleum ether) to afford pure product dinaphthofuran as a white solid (88%). The characterized data is consistent with the reported in the literature.

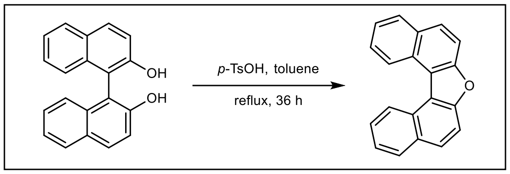

Supplementary Figure 16. Synthesis of substrate

4.4 General Procedure for enantioselective alkylative cross-coupling of C–O electrophiles: In a nitrogen-filled glovebox, $Ni(cod)_2$ (0.10 equiv.), **L1** (0.20 equiv.) were added to an oven-dried 15 mL sealed-tube which was charged with a stir bar. Next, the Grignard reagent in THF (3.0 equiv.) was added via syringe. The vial was capped and the mixture was stirred at room temperature for 10 minutes, at which time it was a brown homogeneous solution. The solvent THF was removed under vacuum. Then, the C-O electrophile (1.0 equiv.) and anhydrous PhMe (0.1 M) was added to the vial, and the cap was sealed. The mixture was stirred vigorously at 60 °C for 24 h or 36 h. **Work-up**: The reaction mixture was quenched with aqueous HCl (1.0 M) or saturated NH_4Cl (aq.), and washed with EtOAc (3.0 mL * 2). The organic layers were combined, the mixture was concentrated, and the residue was purified by flash chromatography on silica gel.

4.5 Analysis and characterization of products

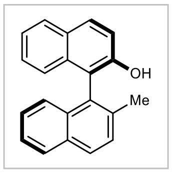

2'-methyl-[1,1'-binaphthalen]-2-ol (Compound **3a**). Prepared according to the general procedure by using $Ni(cod)_2$ (5.6 mg, 0.02 mmol), **L1** (9.8 mg, 0.024 mmol), C-O electrophile (53.7 mg, 0.20 mmol), Me-MgBr (3.0 M in THF, 0.2 mL, 0.60 mmol), 2.0 mL of PhMe. After 24 hours, the reaction afforded compound **3a** with 97% isolated yield as a white solid. Optical Rotation: $[\alpha]^{20}{}_D$ = -112 (c 0.5, CH_2Cl_2). 98% ee, determined by HPLC, HPLC conditions: CHIRALPAK® IA, 10% i-PrOH in hexane, 1.00 mL min^{-1}, λ = 254 nm, τ_R (minor) = 6.2 min; τ_R (major) = 6.8 min. ^1H NMR (600 MHz, $cdcl_3$) δ 7.94 – 7.87 (m, 3H), 7.85 (d, *J* = 8.2 Hz, 1H), 7.51 (d, *J* = 8.3 Hz, 1H), 7.42 (t, *J* = 7.7 Hz, 1H), 7.36 – 7.28 (m, 2H), 7.26 (t, *J* = 7.8 Hz, 1H), 7.21 (d, *J* = 7.7 Hz, 2H), 6.96 (d, *J* = 8.5 Hz, 1H), 4.80 (s, 1H), 2.13 (s, 3H). ^{13}C NMR (150 MHz, $cdcl_3$) δ 150.77, 137.15, 133.36, 133.27, 132.58, 129.77, 129.19, 128.97, 128.96, 128.83, 128.15, 128.11, 126.83, 126.67, 125.52, 125.45, 124.51, 123.39, 117.63, 117.42, 20.07. HRMS (ESI) m/z $[M-H]^-$ calcd for $[C_{21}H_{16}O]^-$ 283.1128, found 283.1143.

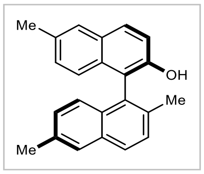

2',6,6'-trimethyl-[1,1'-binaphthalen]-2-ol (Compound **3b**). Prepared according to the general procedure by using $Ni(cod)_2$ (5.6 mg, 0.02 mmol), **L1** (9.8 mg, 0.024 mmol), C-O electrophile (59.3 mg, 0.20 mmol), Me-MgBr (3.0 M in THF, 0.2 mL, 0.60 mmol), 2.0 mL of PhMe. After 24 hours, the reaction afforded compound **3b** with 98% isolated yield as a white solid. Optical Rotation: $[\alpha]^{20}{}_D$ = -83.9 (c 0.56, CH_2Cl_2). 97% ee, determined by HPLC, HPLC conditions: CHIRALPAK® IA, 10% i-PrOH in hexane, 1.00 mL min^{-1}, λ = 254 nm, τ_R (minor) = 5.7 min; τ_R (major) = 6.3 min. ^1H NMR (600 MHz, $cdcl_3$) δ 7.80 (t, *J* = 6.0 Hz, 2H), 7.66 (s, 1H), 7.63 (s, 1H), 7.46 (d, *J* = 8.9 Hz, 1H), 7.29 (d, *J* = 8.8 Hz, 1H), 7.13 – 7.07 (m, 2H), 7.04 (d, *J* = 8.5 Hz, 1H), 6.87 (d, *J* = 8.5 Hz, 1H), 4.72 (s, 1H), 2.45 (s, 3H), 2.43 (s, 3H), 2.10 (s, 3H). ^{13}C NMR (151 MHz, $cdcl_3$) δ 150.11, 136.02, 135.12, 132.78, 132.75, 131.53, 131.51, 129.35, 129.02, 128.99, 128.97, 128.85, 128.76, 128.25, 127.23, 127.12, 125.36, 124.45, 117.67, 117.37, 21.41, 21.27, 19.92. HRMS (ESI) m/z $[M-H]^-$ calcd for $[C_{23}H_{20}O]^-$ 311.1441, found 311.1450.

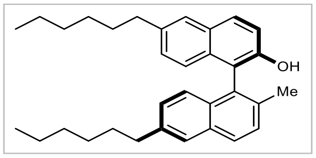

6,6'-dihexyl-2'-methyl-[1,1'-binaphthalen]-2-ol (Compound **3c**). Prepared according to the general procedure by using $Ni(cod)_2$ (5.6 mg, 0.02 mmol), **L9** (9.8 mg, 0.024 mmol), C-O electrophile (87.3

mg, 0.20 mmol), Me-MgBr (3.0 M in THF, 0.2 mL, 0.60 mmol), 2.0 mL of PhMe. After 24 hours, the reaction afforded compound **3c** with 93% isolated yield as a colorless oil. Optical Rotation: $[\alpha]^{20}{}_{D}$ = -95.7 (c 0.23, CH_2Cl_2). 97% ee, determined by HPLC, HPLC conditions: CHIRALPAK® IA, 3% i-PrOH in hexane, 1.00 mL min^{-1}, λ = 254 nm, τ_R (minor) = 5.2 min; τ_R (major) = 5.7 min. ^1H NMR (600 MHz, $cdcl_3$) δ 7.83 (dd, *J* = 12.9, 8.7 Hz, 2H), 7.66 (s, 1H), 7.63 (s, 1H), 7.49 (d, *J* = 8.4 Hz, 1H), 7.30 (d, *J* = 8.9 Hz, 1H), 7.12 (q, *J* = 8.7 Hz, 2H), 7.06 (d, *J* = 8.6 Hz, 1H), 6.89 (d, *J* = 8.6 Hz, 1H), 4.69 (s, 1H), 2.71 (dt, *J* = 14.8, 7.8 Hz, 3H), 2.11 (s, 3H), 1.71 – 1.61 (m, 4H), 1.39 – 1.28 (m, 12H), 0.92 – 0.82 (m, 6H). ^{13}C NMR (100 MHz, $CDCl_3$) δ 150.05, 140.14, 137.85, 136.08, 132.72, 131.67, 129.27, 129.11, 128.92, 128.65, 128.43, 128.36, 128.18, 126.54, 126.48, 125.36, 124.46, 117.66, 117.21, 35.90, 35.82, 31.75, 31.73, 31.34, 31.26, 29.12, 29.07, 22.60, 20.02, 14.09. HRMS (ESI) m/z $[M-H]^-$ calcd for $[C_{33}H_{39}O]^-$ 451.3006, found 451.3007.

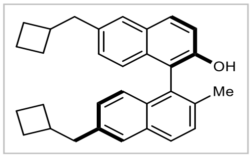

6,6'-bis(cyclobutylmethyl)-2'-methyl-[1,1'-binaphthalen]-2-ol (Compound **3d**). Prepared according to the general procedure by using Ni(cod)$_2$ (5.6 mg, 0.02 mmol), **L1** (9.8 mg, 0.024 mmol), C-O electrophile (80.9 mg, 0.20 mmol), Me-MgBr (3.0 M in THF, 0.2 mL, 0.60 mmol), 2.0 mL of PhMe. After 24 hours, the reaction afforded compound **3d** with 98% isolated yield as a white solid. Optical Rotation: $[\alpha]^{20}{}_{D}$ = -75 (c 0.76, CH_2Cl_2). 96% ee, determined by HPLC, HPLC conditions: CHIRALPAK® IA, 5% i-PrOH in hexane, 1.00 mL min^{-1}, λ = 254 nm, τ_R (minor) = 5.9 min; τ_R (major) = 6.4 min. ^1H NMR (600 MHz, $cdcl_3$) δ 7.82 (t, *J* = 9.0 Hz, 2H), 7.61 (s, 1H), 7.58 (s, 1H), 7.46 (d, *J* = 8.4 Hz, 1H), 7.28 (d, *J* = 8.9 Hz, 1H), 7.14 – 7.03 (m, 2H), 7.01 (d, *J* = 8.6 Hz, 1H), 6.87 (d, *J* = 8.6 Hz, 1H), 4.69 (s, 1H), 2.80 (dd, *J* = 13.2, 7.5 Hz, 4H), 2.63 (h, *J* = 7.8 Hz, 2H), 2.10 (s, 3H), 2.08 – 1.98 (m, 4H), 1.88 – 1.80 (m, 4H), 1.79 – 1.70 (m, 4H). ^{13}C NMR (151 MHz, $cdcl_3$) δ 150.12, 138.57, 136.28, 136.09, 132.75, 131.76, 129.30, 129.16, 128.88, 128.74, 128.47, 128.30, 126.71, 126.64, 125.33, 124.42, 117.70, 117.22, 42.95, 42.87, 37.15, 37.09, 28.38, 28.35, 28.33, 19.99, 18.38, 18.36. HRMS (ESI) m/z $[M-H]^-$ calcd for $[C_{31}H_{31}O]^-$ 419.2380, found 419.2372.

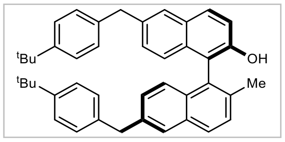

6,6'-bis(4-(tert-butyl)benzyl)-2'-methyl-[1,1'-binaphthalen]-2-ol (Compound **3e**). Prepared according to the general procedure by using Ni(cod)$_2$ (5.6 mg, 0.02 mmol), **L1** (9.8 mg, 0.024 mmol), C-O electrophile (112.2 mg, 0.20 mmol), Me-MgBr (3.0 M in THF, 0.2 mL, 0.60 mmol), 2.0 mL of PhMe. After 24 hours, the reaction afforded compound **3e** with 99% isolated yield as a white solid. Optical Rotation: $[\alpha]^{20}{}_{D}$ = -86.2 (c 1.09, CH_2Cl_2). 95% ee, determined by HPLC, HPLC conditions: CHIRALPAK® AD, 3% i-PrOH in hexane, 1.00 mL min^{-1}, λ = 254 nm, τ_R (minor) = 5.3 min; τ_R (major) = 5.8 min. ^1H NMR (600 MHz, $cdcl_3$) δ 7.80 (t, *J* = 9.4 Hz, 2H), 7.65 (d, *J* = 22.9 Hz, 2H), 7.45

(d, J = 8.3 Hz, 1H), 7.33 – 7.25 (m, 5H), 7.19 – 7.09 (m, 6H), 7.06 (d, J = 8.6 Hz, 1H), 6.87 (d, J = 8.7 Hz, 1H), 4.70 (s, 1H), 4.03 (d, J = 11.9 Hz, 4H), 2.08 (s, 3H), 1.28 (s, 18H). ^{13}C NMR (151 MHz, cdcl$_3$) δ 150.34, 148.96, 148.86, 138.56, 137.99, 137.72, 136.40, 136.23, 132.76, 131.89, 131.87, 129.32, 129.00, 128.73, 128.63, 128.59, 128.45, 127.33, 127.20, 125.63, 125.37, 125.32, 124.71, 117.65, 117.37, 41.41, 41.32, 34.35, 31.38, 20.01. HRMS (ESI) m/z [M-H]$^-$ calcd for $[C_{43}H_{44}O]^-$ 575.3319, found 575.3310.

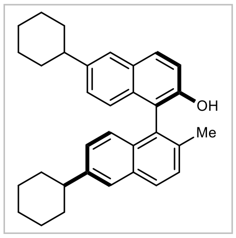

6,6'-dicyclohexyl-2'-methyl-[1,1'-binaphthalen]-2-ol (Compound **3f**). Prepared according to the general procedure by using Ni(cod)$_2$ (5.6 mg, 0.02 mmol), **L1** (9.8 mg, 0.024 mmol), C-O electrophile (86.5 mg, 0.20 mmol), Me-MgBr (3.0 M in THF, 0.2 mL, 0.60 mmol), 2.0 mL of PhMe. After 24 hours, the reaction afforded compound **3f** with 64% isolated yield as a colorless oil. Optical Rotation: $[\alpha]^{20}_{D}$ = -64 (c 0.5, CH_2Cl_2). 65% ee, determined by HPLC, HPLC conditions: CHIRALPAK® IA, 5% i-PrOH in hexane, 1.00 mL min^{-1}, λ = 254 nm, τ_R (major) = 4.5 min; τ_R (minor) = 4.9 min. ^{1}H NMR (600 MHz, cdcl$_3$) δ 7.84 (dd, J = 15.0, 8.5 Hz, 2H), 7.68 (s, 1H), 7.64 (s, 1H), 7.49 (d, J = 8.4 Hz, 1H), 7.29 (d, J = 8.9 Hz, 1H), 7.14 (s, 2H), 7.09 (d, J = 8.7 Hz, 1H), 6.90 (d, J = 8.6 Hz, 1H), 4.67 (s, 1H), 2.68 – 2.49 (m, 2H), 2.11 (s, 2H), 1.93 (d, J = 12.4 Hz, 4H), 1.88 – 1.82 (m, 4H), 1.76 (d, J = 13.0 Hz, 2H), 1.52 – 1.37 (m, 9H), 1.31 – 1.26 (m, 3H). ^{13}C NMR (150 MHz, cdcl$_3$) δ 150.14, 145.19, 142.93, 136.12, 132.83, 131.91, 129.36, 129.31, 128.87, 128.70, 128.64, 127.11, 126.92, 125.42, 124.79, 124.74, 124.52, 117.70, 117.17, 44.44, 44.30, 34.44, 34.42, 34.30, 26.99, 26.94, 26.25, 26.21, 20.04. HRMS (ESI) m/z [M-H]$^-$ calcd for $[C_{33}H_{35}O]^-$ 447.2693, found 447.2701.

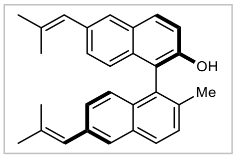

2'-methyl-6,6'-bis(2-methylprop-1-en-1-yl)-[1,1'-binaphthalen]-2-ol (Compound **3g**). Prepared according to the general procedure by using Ni(cod)$_2$ (5.6 mg, 0.02 mmol), **L1** (9.8 mg, 0.024 mmol), C-O electrophile (75.3 mg, 0.20 mmol), Me-MgBr (3.0 M in THF, 0.2 mL, 0.60 mmol), 2.0 mL of PhMe. After 24 hours, the reaction afforded compound **3g** with 99% isolated yield as a white solid. Optical Rotation: $[\alpha]^{20}_{D}$ = -224 (c 0.55, CH_2Cl_2). 97% ee, determined by HPLC, HPLC conditions: CHIRALPAK® OD, 3% i-PrOH in hexane, 1.00 mL min^{-1}, λ = 254 nm, τ_R (major) = 5.0 min; τ_R (minor) = 5.3 min. ^{1}H NMR (600 MHz, cdcl$_3$) δ 7.84 (d, J = 8.5 Hz, 2H), 7.70 (d, J = 13.9 Hz, 2H), 7.46 (d, J = 8.4 Hz, 1H), 7.30 (d, J = 8.9 Hz, 1H), 7.17 (s, 2H), 7.12 (d, J = 8.7 Hz, 1H), 6.92 (d, J = 8.7 Hz, 1H), 6.35 (d, J = 12.5 Hz, 2H), 4.78 (s, 1H), 2.11 (s, 3H), 1.91 (d, J = 8.9 Hz, 12H). ^{13}C NMR (150 MHz, cdcl$_3$) δ 150.49, 136.50, 136.08, 135.96, 135.24, 133.85, 132.57, 131.64, 131.59, 129.57, 129.13,

129.01, 128.78, 128.67, 128.46, 128.30, 127.41, 127.25, 125.09, 125.06, 124.95, 124.18, 117.60, 117.42, 26.92, 20.02, 19.53. HRMS (ESI) m/z $[M-H]^-$ calcd for $[C_{29}H_{28}O]^-$ 391.2067, found 391.2072.

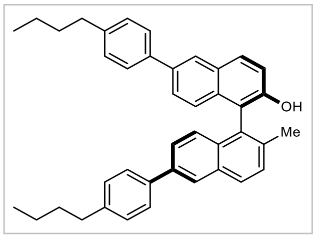

6,6'-bis(4-butylphenyl)-2'-methyl-[1,1'-binaphthalen]-2-ol (Compound **3h**). Prepared according to the general procedure by using Ni(cod)$_2$ (5.6 mg, 0.02 mmol), **L1** (9.8 mg, 0.024 mmol), C-O electrophile (106.5 mg, 0.20 mmol), Me-MgBr (3.0 M in THF, 0.2 mL, 0.60 mmol), 2.0 mL of PhMe. After 24 hours, the reaction afforded compound **3h** with 93% isolated yield as a white solid. Optical Rotation: $[\alpha]^{20}_{D}$ = -136 (c 0.95, CH_2Cl_2). 93% ee, determined by HPLC, HPLC conditions: CHIRALPAK® AD, 20% i-PrOH in hexane, 1.00 mL min^{-1}, λ = 254 nm, τ_R (major) = 5.8 min; τ_R (minor) = 7.3 min. ^{1}H NMR (600 MHz, cdcl$_3$) δ 8.07 (d, *J* = 17.2 Hz, 2H), 7.95 (t, *J* = 7.2 Hz, 2H), 7.63 – 7.55 (m, 4H), 7.53 (d, *J* = 8.3 Hz, 2H), 7.48 (d, *J* = 8.7 Hz, 1H), 7.36 (d, *J* = 8.4 Hz, 1H), 7.30 (d, *J* = 8.8 Hz, 1H), 7.25 (t, *J* = 7.9 Hz, 4H), 7.06 (d, *J* = 8.8 Hz, 1H), 4.83 (s, 1H), 2.64 (s, 4H), 2.16 (s, 3H), 1.63 (t, *J* = 7.8 Hz, 4H), 1.41 – 1.33 (m, 4H), 0.94 (t, *J* = 7.5 Hz, 6H). ^{13}C NMR (151 MHz, cdcl$_3$) δ 150.82, 142.23, 141.86, 138.40, 138.32, 138.14, 137.03, 136.26, 132.93, 132.42, 132.32, 130.05, 129.50, 129.38, 129.24, 128.93, 128.86, 128.68, 127.15, 127.02, 126.54, 126.38, 125.97, 125.82, 125.68, 125.03, 117.82, 117.57, 35.30, 33.58, 22.39, 20.12, 13.92. HRMS (ESI) m/z $[M-H]^-$ calcd for $[C_{41}H_{40}O]^-$ 547.3006, found 547.3007.

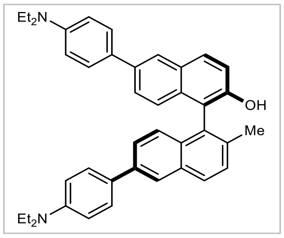

6,6'-bis(4-(diethylamino)phenyl)-2'-methyl-[1,1'-binaphthalen]-2-ol (Compound **3i**). Prepared according to the general procedure by using Ni(cod)$_2$ (5.6 mg, 0.02 mmol), **L1** (9.8 mg, 0.024 mmol), C-O electrophile (112.6 mg, 0.20 mmol), MeMgBr (3.0 M in THF, 0.2 mL, 0.60 mmol), 2.0 mL of PhMe. After 24 hours, the reaction afforded compound **3i** with 75% isolated yield as a pale-yellow solid. Optical Rotation: $[\alpha]^{20}_{D}$ = -240.3 (c 0.77, CH_2Cl_2). 87% ee, determined by HPLC, HPLC conditions: CHIRALPAK® IA, 30% i-PrOH in hexane, 1.00 mL min^{-1}, λ = 254 nm, τ_R (major) = 8.2 min; τ_R (minor) = 9.6 min. ^{1}H NMR (600 MHz, cdcl$_3$) δ 8.01 (d, *J* = 16.5 Hz, 2H), 7.96 – 7.87 (m, 2H), 7.62 – 7.44 (m, 7H), 7.35 (d, *J* = 9.2 Hz, 1H), 7.28 (d, *J* = 8.8 Hz, 1H), 7.05 (d, *J* = 8.8 Hz, 1H), 6.77 (s, 4H), 4.91 (s, 1H), 3.38 (d, *J* = 7.3 Hz, 8H), 2.16 (s, 3H), 1.18 (t, *J* = 7.1 Hz, 12H). ^{13}C NMR (150 MHz, cdcl$_3$) δ 150.42, 138.34, 136.30, 133.11, 131.90, 131.79, 129.73, 129.64, 129.20, 128.95, 128.77, 128.18, 128.10, 127.98, 126.14, 126.05, 125.87, 124.97, 124.46, 124.16, 123.95, 117.71, 117.61, 112.19, 44.43, 31.43, 30.24, 20.08, 12.59. HRMS (ESI) m/z $[M-H]^-$ calcd for $[C_{41}H_{41}N_2O]^-$ 577.3224, found 577.3231.

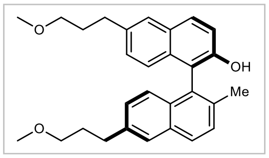

6,6'-bis(3-methoxypropyl)-2'-methyl-[1,1'-binaphthalen]-2-ol (Compound **3j**). Prepared according to the general procedure by using Ni(cod)$_2$ (5.6 mg, 0.02 mmol), **L1** (9.8 mg, 0.024 mmol), C-O electrophile (82.5 mg, 0.20 mmol), Me-MgBr (3.0 M in THF, 0.2 mL, 0.60 mmol), 2.0 mL of PhMe. After 24 hours, the reaction afforded compound **3j** with 92% isolated yield as a white solid. Optical Rotation: $[\alpha]^{20}{}_D$ = -64.2 (c 0.67, CH_2Cl_2). 96% ee, determined by HPLC, HPLC conditions: CHIRALPAK® IA, 5% i-PrOH in hexane, 1.00 mL min^{-1}, λ = 254 nm, τ_R (major) = 12.0 min; τ_R (minor) = 13.0 min. ^{1}H NMR (600 MHz, cdcl$_3$) δ 7.83 (t, *J* = 8.5 Hz, 2H), 7.66 (d, *J* = 20.2 Hz, 2H), 7.48 (d, *J* = 8.4 Hz, 1H), 7.30 (d, *J* = 8.9 Hz, 1H), 7.13 (q, *J* = 8.7 Hz, 2H), 7.07 (d, *J* = 8.7 Hz, 1H), 4.86 (s, 1H), 3.39 (q, *J* = 6.5 Hz, 4H), 3.32 (d, *J* = 2.5 Hz, 6H), 2.84 – 2.73 (m, 4H), 2.11 (s, 3H), 1.93 (h, *J* = 7.4, 6.6 Hz, 4H). ^{13}C NMR (151 MHz, cdcl$_3$) δ 150.26, 139.14, 136.83, 136.15, 132.74, 131.82, 131.80, 129.28, 129.12, 128.97, 128.84, 128.39, 128.23, 128.08, 126.73, 126.66, 125.51, 124.58, 117.68, 117.40, 72.02, 71.92, 58.49, 32.22, 32.13, 31.07, 31.01, 19.98. HRMS (ESI) m/z [M-H]$^-$ calcd for $[C_{29}H_{31}O_3]^-$ 427.2279, found 427.2288.

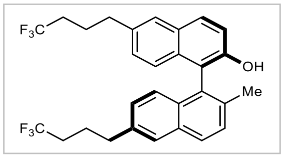

2'-methyl-6,6'-bis(4,4,4-trifluorobutyl)-[1,1'-binaphthalen]-2-ol (Compound **3k**). Prepared according to the general procedure by using Ni(cod)$_2$ (5.6 mg, 0.02 mmol), **L1** (9.8 mg, 0.024 mmol), C-O electrophile (97.7 mg, 0.20 mmol), Me-MgBr (3.0 M in THF, 0.2 mL, 0.60 mmol), 2.0 mL of PhMe. After 24 hours, the reaction afforded compound **3k** with 98% isolated yield as a white solid. Optical Rotation: $[\alpha]^{20}{}_D$ = -78.7 (c 0.89, CH_2Cl_2). 96% ee, determined by HPLC, HPLC conditions: CHIRALPAK® IA, 10% i-PrOH in hexane, 1.00 mL min^{-1}, λ = 254 nm, τ_R (minor) = 6.1 min; τ_R (major) = 6.8 min. ^{1}H NMR (600 MHz, cdcl$_3$) δ 7.85 (t, *J* = 9.8 Hz, 2H), 7.66 (d, *J* = 22.2 Hz, 2H), 7.51 (d, *J* = 8.4 Hz, 1H), 7.32 (d, *J* = 8.8 Hz, 1H), 7.20 – 7.08 (m, 2H), 7.05 (d, *J* = 8.6 Hz, 1H), 6.92 (d, *J* = 8.5 Hz, 1H), 4.73 (s, 1H), 2.80 (dt, *J* = 15.2, 7.6 Hz, 4H), 2.11 (d, *J* = 10.2 Hz, 7H), 1.95 (dt, *J* = 7.8, 3.8 Hz, 4H). ^{13}C NMR (151 MHz, cdcl$_3$) δ 150.47, 137.90, 136.63, 135.61, 132.75, 132.02, 132.00, 129.29, 129.24, 128.70, 128.56, 127.92, 127.77, 127.23 (q, *J* = 276.1 Hz), 127.10 (q, *J* = 276.1 Hz), 126.95, 126.86, 125.80, 124.86, 117.62, 34.55, 34.50, 33.21 (q, *J* = 28.5 Hz), 33.17 (q, *J* = 28.5 Hz), 23.34 (q, *J* = 11.7 Hz), 23.32 (q, *J* = 11.7 Hz), 20.00. HRMS (ESI) m/z [M-H]$^-$ calcd for $[C_{29}H_{25}F_6O]^-$ 503.1815, found 503.1818.

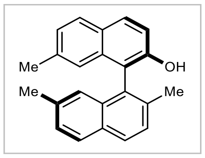

2',7,7'-trimethyl-[1,1'-binaphthalen]-2-ol (Compound **3l**). Prepared according to the general procedure by using Ni(cod)$_2$ (5.6 mg, 0.02 mmol), **L1** (9.8 mg, 0.024 mmol), C-O electrophile (59.3 mg, 0.20 mmol), Me-MgBr (3.0 M in THF, 0.2 mL, 0.60 mmol), 2.0 mL of PhMe. After 24 hours,

the reaction afforded compound **3l** with 96% isolated yield as a white solid. Optical Rotation: $[\alpha]^{20}_{D}$ = -77.8 (c 0.54, CH_2Cl_2). 96% ee, determined by HPLC, HPLC conditions: CHIRALPAK® IA, 10% i-PrOH in hexane, 1.00 mL min^{-1}, λ = 254 nm, τ_R (minor) = 5.0 min; τ_R (major) = 5.8 min. ^{1}H NMR (600 MHz, cdcl3) δ 7.88 (t, *J* = 10.0 Hz, 2H), 7.82 (d, *J* = 8.2 Hz, 1H), 7.78 (d, *J* = 8.2 Hz, 1H), 7.48 (d, *J* = 8.4 Hz, 1H), 7.29 (d, *J* = 8.8 Hz, 2H), 7.18 (d, *J* = 7.9 Hz, 1H), 7.03 (s, 1H), 6.77 (s, 1H), 4.76 (s, 1H), 2.29 (s, 3H), 2.26 (s, 3H), 2.11 (s, 3H). ^{13}C NMR (151 MHz, cdcl3) δ 150.82, 137.20, 136.61, 136.39, 133.59, 133.46, 130.85, 129.37, 128.61, 128.28, 128.09, 128.00, 127.96, 127.81, 127.45, 125.66, 124.30, 123.50, 117.31, 116.40, 21.86, 21.82, 20.16. HRMS (ESI) m/z $[M-H]^-$ calcd for $[C_{23}H_{20}O]^-$ 311.1441, found 311.1449.

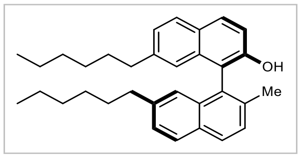

7,7'-dihexyl-2'-methyl-[1,1'-binaphthalen]-2-ol (Compound **3m**). Prepared according to the general procedure by using Ni(cod)2 (5.6 mg, 0.02 mmol), **L1** (9.8 mg, 0.024 mmol), C-O electrophile (87.3 mg, 0.20 mmol), Me-MgBr (3.0 M in THF, 0.2 mL, 0.60 mmol), 2.0 mL of PhMe. After 24 hours, the reaction afforded compound **3m** with 93% isolated yield as a colorless oil. Optical Rotation: $[\alpha]^{20}_{D}$ = -129.1 (c 0.79, CH_2Cl_2). 98% ee, determined by HPLC, HPLC conditions: CHIRALPAK® IA, 5% i-PrOH in hexane, 1.00 mL min^{-1}, λ = 254 nm, τ_R (minor) = 4.1 min; τ_R (major) = 4.8 min. ^{1}H NMR (600 MHz, cdcl3) δ 7.86 (dd, *J* = 11.9, 8.6 Hz, 2H), 7.82 (d, *J* = 8.4 Hz, 1H), 7.78 (d, *J* = 8.3 Hz, 1H), 7.46 (d, *J* = 8.3 Hz, 1H), 7.28 (dd, *J* = 18.6, 7.8 Hz, 1H), 7.17 (d, *J* = 6.7 Hz, 1H), 7.00 (s, 1H), 6.75 (s, 1H), 4.72 (s, 1H), 2.48 (dt, *J* = 16.1, 7.7 Hz, 4H), 2.09 (s, 3H), 1.48 – 1.40 (m, 4H), 1.20 – 1.11 (m, 12H), 0.83 – 0.75 (m, 6H). ^{13}C NMR (151 MHz, cdcl3) δ 150.80, 141.57, 141.34, 137.08, 133.53, 133.40, 131.07, 129.33, 128.58, 128.46, 128.13, 128.00, 127.98, 127.64, 127.00, 124.86, 123.94, 123.19, 117.43, 116.42, 36.10, 31.59, 31.58, 31.33, 31.27, 28.79, 28.75, 22.53, 22.51, 20.15, 13.98. HRMS (ESI) m/z $[M-H]^-$ calcd for $[C_{33}H_{40}O]^-$ 451.3006, found 451.3009.

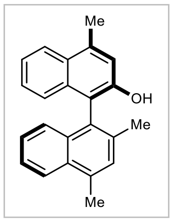

2',4,4'-trimethyl-[1,1'-binaphthalen]-2-ol (Compound **3n**). Prepared according to the general procedure by using Ni(cod)2 (5.6 mg, 0.02 mmol), **L1** (9.8 mg, 0.024 mmol), C-O electrophile (59.3 mg, 0.20 mmol), Me-MgBr (3.0 M in THF, 0.2 mL, 0.60 mmol), 2.0 mL of PhMe. After 24 hours, the reaction afforded compound **3n** with 81% isolated yield as a white solid. Optical Rotation: $[\alpha]^{20}_{D}$ = -40 (c 0.1, CH_2Cl_2). 94% ee, determined by HPLC, HPLC conditions: CHIRALPAK® IA, 3% i-PrOH in hexane, 1.00 mL min^{-1}, λ = 254 nm, τ_R (minor) = 7.0 min; τ_R (major) = 8.6 min. ^{1}H NMR (600 MHz, cdcl3) δ 8.06 (d, *J* = 8.4 Hz, 1H), 8.01 (d, *J* = 8.4 Hz, 1H), 7.47 (dd, *J* = 8.2, 5.8 Hz, 1H), 7.41 (s, 1H), 7.35 (t, *J* = 7.7 Hz, 1H), 7.26 (d, *J* = 6.6 Hz, 2H), 7.21 (dd, *J* = 7.6, 4.7 Hz, 2H), 7.00 (d, *J* = 8.6 Hz, 1H), 4.71 (s, 1H), 2.79 (s, 6H), 2.11 (s, 3H). ^{13}C NMR (151 MHz, cdcl3) δ 150.36, 136.92, 136.40, 135.25, 133.77, 133.53, 131.82, 129.88, 128.51, 127.04, 126.42, 126.35, 126.15, 125.29, 125.21,

124.32, 124.27, 123.13, 118.06, 115.92, 20.05, 19.47, 19.41. HRMS (ESI) m/z $[M-H]^-$ calcd for $[C_{23}H_{19}O]^-$ 311.1441, found 311.1450.

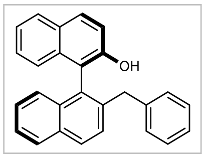

2'-benzyl-[1,1'-binaphthalen]-2-ol (Compound **3o**). Prepared according to the general procedure by using Ni(cod)$_2$ (5.6 mg, 0.02 mmol), **L1** (9.8 mg, 0.024 mmol), C-O electrophile (53.7 mg, 0.20 mmol), Bn-MgCl (0.60 mmol), 2.0 mL of PhMe. After 36 hours, the reaction afforded compound **3o** with 91% isolated yield as a white solid. Optical Rotation: $[\alpha]^{20}{}_D$ = 19.4 (c 0.62, CH_2Cl_2). 99% ee, determined by HPLC, HPLC conditions: CHIRALPAK® IA, 10% i-PrOH in hexane, 1.00 mL min^{-1}, λ = 254 nm, τ_R (major) = 5.7 min; τ_R (minor) = 6.9 min. ^{1}H NMR (600 MHz, cdcl$_3$) δ 7.92 (dd, *J* = 8.8, 4.8 Hz, 2H), 7.88 (dd, *J* = 15.2, 8.2 Hz, 2H), 7.51 (d, *J* = 8.5 Hz, 1H), 7.44 (t, *J* = 7.4 Hz, 1H), 7.31 (q, *J* = 8.0 Hz, 2H), 7.27 (d, *J* = 7.7 Hz, 1H), 7.25 – 7.23 (m, 1H), 7.20 (t, *J* = 7.6 Hz, 1H), 7.10 (dq, *J* = 14.6, 7.2 Hz, 3H), 6.99 (d, *J* = 8.4 Hz, 1H), 6.90 (d, *J* = 6.9 Hz, 2H), 4.70 (s, 1H), 3.77 (q, *J* = 12 Hz, 2H). ^{13}C NMR (151 MHz, cdcl$_3$) δ 151.18, 140.36, 140.30, 133.67, 133.24, 132.76, 130.01, 129.26, 129.16, 129.05, 128.98, 128.30, 128.27, 128.12, 126.96, 126.69, 126.00, 125.94, 125.79, 124.64, 123.46, 117.49, 117.34, 39.74. HRMS (ESI) m/z $[M-H]^-$ calcd for $[C_{27}H_{20}O]^-$ 359.1441, found 359.1450.

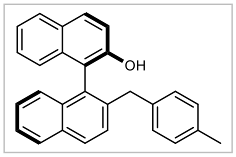

2'-(4-methylbenzyl)-[1,1'-binaphthalen]-2-ol (Compound **3p**). Prepared according to the general procedure by using Ni(cod)$_2$ (5.6 mg, 0.02 mmol), **L1** (9.8 mg, 0.024 mmol), C-O electrophile (53.7 mg, 0.20 mmol), 4-Me-$C_6H_4CH_2$-MgCl (0.60 mmol), 2.0 mL of PhMe. After 36 hours, the reaction afforded compound **3p** with 94% isolated yield as a white solid. Optical Rotation: $[\alpha]^{20}{}_D$ = 10.8 (c 0.65, CH_2Cl_2). 91% ee, determined by HPLC, HPLC conditions: CHIRALPAK® IA, 10% i-PrOH in hexane, 1.00 mL min^{-1}, λ = 254 nm, τ_R (major) = 5.3 min; τ_R (minor) = 5.9 min. ^{1}H NMR (600 MHz, cdcl$_3$) δ 7.91 (d, *J* = 7.8 Hz, 2H), 7.87 (t, *J* = 9.3 Hz, 2H), 7.50 (d, *J* = 8.6 Hz, 1H), 7.43 (t, *J* = 7.4 Hz, 1H), 7.35 – 7.29 (m, 2H), 7.27 (t, *J* = 7.6 Hz, 1H), 7.23 (d, *J* = 4.8 Hz, 1H), 7.20 (t, *J* = 7.6 Hz, 1H), 6.99 (d, *J* = 8.5 Hz, 1H), 6.93 (d, *J* = 7.6 Hz, 2H), 6.81 (d, *J* = 7.6 Hz, 2H), 4.72 (s, 1H), 3.72 (d, *J* = 2.9 Hz, 2H), 2.23 (s, 3H). ^{13}C NMR (151 MHz, cdcl$_3$) δ 151.18, 140.63, 137.25, 135.46, 133.71, 133.24, 132.73, 129.97, 129.23, 129.17, 128.99, 128.94, 128.83, 128.24, 128.11, 126.92, 126.66, 125.88, 125.80, 124.70, 123.42, 117.49, 117.41, 39.23, 20.92. HRMS (ESI) m/z $[M-H]^-$ calcd for $[C_{28}H_{22}O]^-$ 373.1598, found 373.1608.

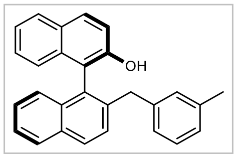

2'-(3-methylbenzyl)-[1,1'-binaphthalen]-2-ol (Compound **3q**). Prepared according to the general procedure by using Ni(cod)$_2$ (5.6 mg, 0.02 mmol), **L1** (9.8 mg, 0.024 mmol), C-O electrophile (53.7

mg, 0.20 mmol), 3-Me-$C_6H_4CH_2$-MgCl (0.60 mmol), 2.0 mL of PhMe. After 24 hours, the reaction afforded compound **3q** with 97% isolated yield as a white solid. Optical Rotation: $[\alpha]^{20}{}_D$ = 4.5 (c 0.67, CH_2Cl_2). 95% ee, determined by HPLC, HPLC conditions: CHIRALPAK® IA, 5% i-PrOH in hexane, 1.00 mL min^{-1}, λ = 254 nm, τ_R (major) = 7.0 min; τ_R (minor) = 7.6 min. ^1H NMR (600 MHz, $cdcl_3$) δ 7.96 – 7.83 (m, 4H), 7.53 (d, *J* = 8.5 Hz, 1H), 7.44 (t, *J* = 7.3 Hz, 1H), 7.36 – 7.15 (m, 5H), 7.06 – 6.94 (m, 2H), 6.89 (d, *J* = 7.5 Hz, 1H), 6.73 (d, *J* = 7.7 Hz, 1H), 6.62 (s, 1H), 4.69 (s, 1H), 3.81 – 3.64 (m, 2H), 2.16 (s, 3H). ^{13}C NMR (151 MHz, $cdcl_3$) δ 151.20, 140.52, 140.15, 137.79, 133.71, 133.29, 132.77, 129.97, 129.93, 129.26, 129.18, 128.94, 128.38, 128.12, 128.09, 126.92, 126.72, 126.62, 126.02, 125.90, 125.81, 124.72, 123.44, 117.49, 117.40, 39.76, 21.22. HRMS (ESI) m/z $[M-H]^-$ calcd for $[C_{28}H_{22}O]^-$ 373.1598, found 373.1606.

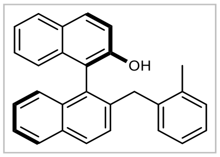

2'-(2-methylbenzyl)-[1,1'-binaphthalen]-2-ol (Compound **3r**). Prepared according to the general procedure by using $Ni(cod)_2$ (5.6 mg, 0.02 mmol), **L1** (9.8 mg, 0.024 mmol), C-O electrophile (53.7 mg, 0.20 mmol), 2-Me-$C_6H_4CH_2$-MgCl (0.60 mmol), 2.0 mL of PhMe. After 36 hours, the reaction afforded compound **3r** with 97% isolated yield as a white solid. Optical Rotation: $[\alpha]^{20}{}_D$ = 13.4 (c 0.67, CH_2Cl_2). 99% ee, determined by HPLC, HPLC conditions: CHIRALPAK® IA, 10% i-PrOH in hexane, 1.00 mL min^{-1}, λ = 254 nm, τ_R (major) = 5.1 min; τ_R (minor) = 6.2 min. ^1H NMR (600 MHz, $cdcl_3$) δ 7.90 (dd, *J* = 8.7, 4.5 Hz, 3H), 7.86 (d, *J* = 8.2 Hz, 1H), 7.45 (t, *J* = 7.4 Hz, 1H), 7.35 – 7.20 (m, 6H), 7.11 – 7.00 (m, 4H), 6.93 (d, *J* = 6.4 Hz, 1H), 4.73 (s, 1H), 3.83 – 3.68 (m, 2H), 1.95 (s, 3H). ^{13}C NMR (151 MHz, $cdcl_3$) δ 151.02, 139.78, 138.16, 136.60, 133.45, 133.26, 132.73, 130.17, 130.12, 129.99, 129.26, 129.20, 129.15, 128.18, 128.15, 127.52, 126.95, 126.71, 126.38, 125.95, 125.87, 125.65, 124.60, 123.49, 117.53, 117.35, 36.96, 19.55. HRMS (ESI) m/z $[M-H]^-$ calcd for $[C_{28}H_{22}O]^-$ 373.1598, found 373.1609.

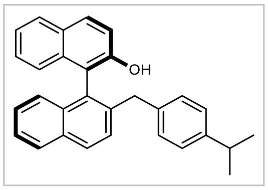

2'-(4-isopropylbenzyl)-[1,1'-binaphthalen]-2-ol (Compound **3s**). Prepared according to the general procedure by using $Ni(cod)_2$ (5.6 mg, 0.02 mmol), **L1** (9.8 mg, 0.024 mmol), C-O electrophile (53.7 mg, 0.20 mmol), 4-C_3H_7-$C_6H_4CH_2$-MgCl (0.60 mmol), 2.0 mL of PhMe. After 36 hours, the reaction afforded compound **3s** with 97% isolated yield as a white solid. Optical Rotation: $[\alpha]^{20}{}_D$ = 14.3 (c 0.63, CH_2Cl_2). 99% ee, determined by HPLC, HPLC conditions: CHIRALPAK® OD, 3% i-PrOH in hexane, 1.00 mL min^{-1}, λ = 254 nm, τ_R (major) = 7.0 min; τ_R (minor) = 7.9 min. ^1H NMR (600 MHz, $cdcl_3$) δ 7.91 (dd, *J* = 8.7, 4.0 Hz, 2H), 7.86 (dd, *J* = 17.2, 8.2 Hz, 2H), 7.52 (d, *J* = 8.6 Hz, 1H), 7.43 (t, *J* = 6.7 Hz, 1H), 7.33 – 7.20 (m, 3H), 7.19 – 7.13 (m, 2H), 6.95 (dd, *J* = 8.2, 4.6 Hz, 3H), 6.81 (d, *J* = 7.8 Hz, 2H), 4.74 (s, 1H), 3.73 (s, 2H), 2.81 – 2.69 (m, 1H), 1.16 (d, *J* = 6.9 Hz, 6H). ^{13}C NMR (151 MHz, $cdcl_3$) δ 151.16, 146.47, 140.62, 137.57, 133.75, 133.28, 132.74, 130.61, 129.95, 129.19, 129.15, 128.93, 128.38, 128.10, 128.06, 126.89, 126.60, 126.27, 126.18,

125.86, 125.82, 124.72, 123.40, 117.49, 117.44, 39.33, 33.61, 23.96. HRMS (ESI) m/z [M-H]- calcd for [$C_{30}H_{26}O$]- 401.1911, found 401.1916.

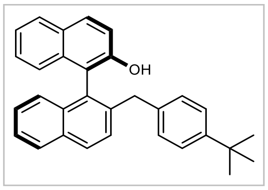

2'-(4-(tert-butyl)benzyl)-[1,1'-binaphthalen]-2-ol (Compound **3t**). Prepared according to the general procedure by using Ni(cod)$_2$ (5.6 mg, 0.02 mmol), **L1** (9.8 mg, 0.024 mmol), C-O electrophile (53.7 mg, 0.20 mmol), 4-tBu-$C_6H_4CH_2$-MgCl (0.60 mmol), 2.0 mL of PhMe. After 36 hours, the reaction afforded compound **3t** with 81% isolated yield as a white solid. Optical Rotation: $[\alpha]^{20}{}_D$ = 10 (c 0.60, CH_2Cl_2). 90% ee, determined by HPLC, HPLC conditions: CHIRALPAK® OD, 5% i-PrOH in hexane, 1.00 mL min^{-1}, λ = 254 nm, τ_R (major) = 5.6 min; τ_R (minor) = 6.2 min. ^{1}H NMR (600 MHz, cdcl$_3$) δ 7.95 – 7.82 (m, 4H), 7.54 (d, *J* = 8.6 Hz, 1H), 7.43 (t, *J* = 7.4 Hz, 1H), 7.34 – 7.21 (m, 4H), 7.16 (t, *J* = 7.6 Hz, 1H), 7.11 (d, *J* = 7.9 Hz, 2H), 6.94 (d, *J* = 8.5 Hz, 1H), 6.81 (d, *J* = 7.9 Hz, 2H), 4.74 (s, 1H), 3.74 (s, 2H), 1.23 (s, 9H). ^{13}C NMR (151 MHz, cdcl$_3$) δ 151.14, 148.75, 140.61, 137.18, 133.76, 133.29, 132.76, 129.96, 129.21, 129.15, 128.93, 128.66, 128.43, 128.10, 128.05, 126.90, 126.59, 125.87, 125.82, 125.11, 124.74, 123.40, 117.47, 117.45, 39.25, 34.27, 31.33. HRMS (ESI) m/z [M-H]- calcd for [$C_{31}H_{27}O$]- 415.2067, found 415.2082.

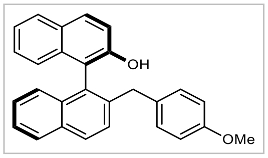

2'-(4-methoxybenzyl)-[1,1'-binaphthalen]-2-ol (Compound **3u**). Prepared according to the general procedure by using Ni(cod)$_2$ (5.6 mg, 0.02 mmol), **L1** (9.8 mg, 0.024 mmol), C-O electrophile (53.7 mg, 0.20 mmol), 4-OMe-$C_6H_4CH_2$-MgCl (0.60 mmol), 2.0 mL of PhMe. After 36 hours, the reaction afforded compound **3u** with 79% isolated yield as a white solid. Optical Rotation: $[\alpha]^{20}{}_D$ = 11.9 (c 0.59, CH_2Cl_2). 85% ee, determined by HPLC, HPLC conditions: CHIRALPAK® OD, 5% i-PrOH in hexane, 1.00 mL min^{-1}, λ = 254 nm, τ_R (major) = 6.7 min; τ_R (minor) = 7.5 min. ^{1}H NMR (600 MHz, cdcl$_3$) δ 7.91 (d, *J* = 8.7 Hz, 2H), 7.87 (dd, *J* = 11.8, 8.1 Hz, 2H), 7.50 (d, *J* = 8.5 Hz, 1H), 7.44 (t, *J* = 7.4 Hz, 1H), 7.35 – 7.16 (m, 5H), 6.97 (d, *J* = 8.4 Hz, 1H), 6.81 (d, *J* = 8.5 Hz, 2H), 6.66 (d, *J* = 8.6 Hz, 2H), 4.72 (s, 1H), 3.70 (s, 5H). ^{13}C NMR (151 MHz, cdcl$_3$) δ 157.93, 151.15, 140.74, 133.71, 133.25, 132.73, 132.45, 129.97, 129.24, 129.16, 128.78, 128.23, 128.11, 126.92, 126.66, 125.88, 125.78, 124.68, 123.43, 117.49, 117.40, 113.77, 55.18, 38.83. HRMS (ESI) m/z [M-H]- calcd for [$C_{28}H_{22}O_2$]- 389.1547, found 389.1556.

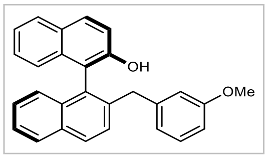

2'-(3-methoxybenzyl)-[1,1'-binaphthalen]-2-ol (Compound **3v**). Prepared according to the general procedure by using Ni(cod)$_2$ (5.6 mg, 0.02 mmol), **L9** (9.8 mg, 0.024 mmol), C-O electrophile (53.7

mg, 0.20 mmol), 3-OMe-$C_6H_4CH_2$-MgCl (0.60 mmol), 2.0 mL of PhMe. After 36 hours, the reaction afforded compound **3v** with 98% isolated yield as a white solid. Optical Rotation: $[\alpha]^{20}{}_D$ = 14.1 (c 0.64, CH_2Cl_2). 97% ee, determined by HPLC, HPLC conditions: CHIRALPAK® OD, 5% i-PrOH in hexane, 1.00 mL min^{-1}, λ = 254 nm, τ_R (major) = 8.5 min; τ_R (minor) = 9.7 min. ^1H NMR (600 MHz, cdcl3) δ 7.98 – 7.80 (m, 4H), 7.53 (d, *J* = 8.5 Hz, 1H), 7.44 (t, *J* = 7.4 Hz, 1H), 7.36 – 7.15 (m, 5H), 7.04 (t, *J* = 7.9 Hz, 1H), 6.99 (d, *J* = 8.5 Hz, 1H), 6.63 (dd, *J* = 8.3, 2.6 Hz, 1H), 6.55 (d, *J* = 7.6 Hz, 1H), 6.40 (t, *J* = 2.0 Hz, 1H), 4.73 (s, 1H), 3.78 – 3.70 (m, 2H), 3.62 (s, 3H). ^{13}C NMR (151 MHz, cdcl3) δ 159.59, 151.23, 141.89, 140.22, 133.71, 133.24, 132.79, 130.02, 129.29, 129.20, 129.16, 128.95, 128.30, 128.29, 128.13, 126.95, 126.69, 125.94, 125.81, 124.66, 123.45, 121.45, 117.50, 117.37, 114.59, 111.79, 55.00, 39.81. HRMS (ESI) m/z $[M-H]^-$ calcd for $[C_{28}H_{21}O_2]^-$ 389.1547, found 389.1549.

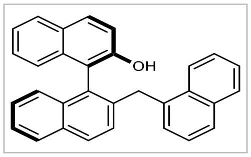

2'-(naphthalen-1-ylmethyl)-[1,1'-binaphthalen]-2-ol (Compound **3w**). Prepared according to the general procedure by using $Ni(cod)_2$ (5.6 mg, 0.02 mmol), **L1** (9.8 mg, 0.024 mmol), C-O electrophile (53.7 mg, 0.20 mmol), 1-NpCH_2-MgCl (0.60 mmol), 2.0 mL of PhMe. After 36 hours, the reaction afforded compound **3w** with 78% isolated yield as a white solid. Optical Rotation: $[\alpha]^{20}{}_D$ = 1.5 (c 0.57, CH_2Cl_2). 96% ee, determined by HPLC, HPLC conditions: CHIRALPAK® IA, 10% i-PrOH in hexane, 1.00 mL min^{-1}, λ = 254 nm, τ_R (major) = 8.1 min; τ_R (minor) = 9.7 min. ^1H NMR (600 MHz, cdcl3) δ 7.91 – 7.82 (m, 3H), 7.77 (t, *J* = 8.3 Hz, 2H), 7.68 (t, *J* = 7.7 Hz, 2H), 7.44 – 7.41 (m, 1H), 7.38 – 7.19 (m, 9H), 7.15 (t, *J* = 8.6 Hz, 2H), 4.90 (s, 1H), 4.20 (q, *J* = 16.5 Hz, 2H). ^{13}C NMR (151 MHz, cdcl3) δ 151.16, 139.89, 135.99, 133.90, 133.54, 133.22, 132.73, 132.19, 130.11, 129.35, 129.19, 128.96, 128.55, 128.31, 128.15, 127.85, 127.54, 127.13, 126.96, 126.76, 125.90, 125.78, 125.74, 125.44, 125.40, 124.69, 124.24, 123.55, 117.58, 117.35, 36.63. HRMS (ESI) m/z $[M-H]^-$ calcd for $[C_{31}H_{22}O]^-$ 409.1598, found 409.1598.

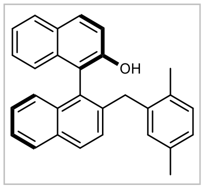

2'-(2,5-dimethylbenzyl)-[1,1'-binaphthalen]-2-ol (Compound **3x**). Prepared according to the general procedure by using $Ni(cod)_2$ (5.6 mg, 0.02 mmol), **L1** (9.8 mg, 0.024 mmol), C-O electrophile (53.7 mg, 0.20 mmol), 2,5-Me_2-$C_6H_3CH_2$-MgCl (0.60 mmol), 2.0 mL of PhMe. After 36 hours, the reaction afforded compound **3x** with 97% isolated yield as a white solid. Optical Rotation: $[\alpha]^{20}{}_D$ = 2.9 (c 0.7, CH_2Cl_2). 99% ee, determined by HPLC, HPLC conditions: CHIRALPAK® IA, 5% i-PrOH in hexane, 1.00 mL min^{-1}, λ = 254 nm, τ_R (major) = 6.5 min; τ_R (minor) = 7.8 min. ^1H NMR (600 MHz, cdcl3) δ 7.92 – 7.83 (m, 4H), 7.43 (t, *J* = 7.3 Hz, 1H), 7.34 – 7.17 (m, 6H), 7.02 (d, *J* = 8.4 Hz, 1H), 6.92 (d, *J* = 7.6 Hz, 1H), 6.86 (d, *J* = 7.5 Hz, 1H), 6.69 (s, 1H), 4.74 (s, 1H), 3.83 – 3.61 (m, 2H), 2.17 (s, 3H), 1.93 (s, 3H). ^{13}C NMR (151 MHz, cdcl3) δ 151.00, 139.92, 137.85, 135.22, 133.46, 133.36, 133.28, 132.70, 130.96, 130.04, 129.95, 129.24, 129.15, 129.10, 128.15, 128.12,

127.65, 127.03, 126.90, 126.63, 125.82, 125.65, 124.63, 123.45, 117.51, 117.39, 37.10, 20.83, 19.12. HRMS (ESI) m/z [M-H]$^-$ calcd for [$C_{29}H_{24}O$]$^-$ 387.1754, found 387.1762.

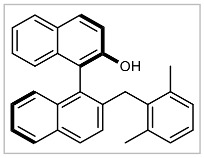

2'-(2,6-dimethylbenzyl)-[1,1'-binaphthalen]-2-ol (Compound **3y**). Prepared according to the general procedure by using Ni(cod)$_2$ (5.6 mg, 0.02 mmol), **L1** (9.8 mg, 0.024 mmol), C-O electrophile (53.7 mg, 0.20 mmol), 2,6-Me$_2$-$C_6H_3CH_2$-MgCl (0.60 mmol), 2.0 mL of PhMe. After 36 hours, the reaction afforded compound **3y** with 98% isolated yield as a white solid. Optical Rotation: $[\alpha]^{20}_{D}$ = 28.6 (c 0.7, CH_2Cl_2). 98% ee, determined by HPLC, HPLC conditions: CHIRALPAK® IA, 5% i-PrOH in hexane, 1.00 mL min^{-1}, λ = 254 nm, τ_R (major) = 5.7 min; τ_R (minor) = 6.4 min. ^{1}H NMR (600 MHz, cdcl$_3$) δ 7.94 (d, *J* = 8.9 Hz, 1H), 7.89 (dd, *J* = 15.3, 8.2 Hz, 2H), 7.81 (d, *J* = 8.6 Hz, 1H), 7.44 (dt, *J* = 8.1, 3.9 Hz, 1H), 7.40 (d, *J* = 9.0 Hz, 1H), 7.35 (t, *J* = 6.9 Hz, 1H), 7.28 (dd, *J* = 17.0, 5.4 Hz, 3H), 7.08 (dd, *J* = 15.6, 8.5 Hz, 2H), 7.02 (d, *J* = 7.7 Hz, 2H), 6.95 (d, *J* = 8.6 Hz, 1H), 4.87 (s, 1H), 3.83 – 3.63 (m, 2H), 2.08 (s, 6H). ^{13}C NMR (151 MHz, cdcl$_3$) δ 150.83, 138.55, 137.37, 135.96, 133.24, 133.21, 132.72, 130.02, 129.40, 129.37, 129.20, 128.33, 128.12, 128.08, 126.94, 126.80, 126.41, 125.75, 125.37, 125.30, 124.44, 123.59, 117.58, 117.38, 33.03, 20.06. HRMS (ESI) m/z [M-H]$^-$ calcd for [$C_{29}H_{24}O$]$^-$ 387.1754, found 387.1759.

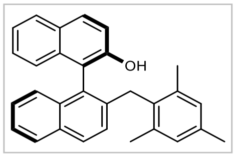

2'-(2,4,6-trimethylbenzyl)-[1,1'-binaphthalen]-2-ol (Compound **3z**). Prepared according to the general procedure by using Ni(cod)$_2$ (5.6 mg, 0.02 mmol), **L1** (9.8 mg, 0.024 mmol), C-O electrophile (53.7 mg, 0.20 mmol), 2,4,6-Me$_3$-$C_6H_2CH_2$-MgCl (0.60 mmol), 2.0 mL of PhMe. After 36 hours, the reaction afforded compound **3z** with 99% isolated yield as a white solid. Optical Rotation: $[\alpha]^{20}_{D}$ = 17.9 (c 0.67, CH_2Cl_2). 99.5% ee, determined by HPLC, HPLC conditions: CHIRALPAK® IA, 10% i-PrOH in hexane, 1.00 mL min^{-1}, λ = 254 nm, τ_R (major) = 5.3 min; τ_R (minor) = 5.5 min. ^{1}H NMR (600 MHz, cdcl$_3$) δ 7.94 (d, *J* = 8.8 Hz, 1H), 7.89 (dd, *J* = 14.9, 8.2 Hz, 2H), 7.81 (d, *J* = 8.6 Hz, 1H), 7.47 – 7.41 (m, 1H), 7.40 (d, *J* = 8.9 Hz, 1H), 7.35 (t, *J* = 7.5 Hz, 1H), 7.31 – 7.25 (m, 3H), 7.08 (d, *J* = 8.5 Hz, 1H), 6.97 (d, *J* = 8.6 Hz, 1H), 6.85 (s, 2H), 4.87 (s, 1H), 3.79 – 3.58 (m, 2H), 2.27 (s, 3H), 2.04 (s, 6H). ^{13}C NMR (151 MHz, cdcl$_3$) δ 150.82, 138.86, 137.20, 135.73, 133.25, 133.21, 132.82, 132.70, 129.99, 129.38, 129.34, 129.07, 128.87, 128.32, 128.11, 126.89, 126.78, 125.70, 125.39, 125.36, 124.47, 123.56, 117.57, 117.41, 32.65, 20.89, 19.98. HRMS (ESI) m/z [M-H]$^-$ calcd for [$C_{30}H_{26}O$]$^-$ 401.1911, found 401.1919.

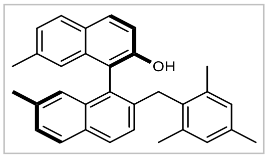

7,7'-dimethyl-2'-(2,4,6-trimethylbenzyl)-[1,1'-binaphthalen]-2-ol (Compound **3aa**). Prepared according to the general procedure by using Ni(cod)$_2$ (5.6 mg, 0.02 mmol), **L1** (9.8 mg, 0.024 mmol), C-O electrophile (59.5 mg, 0.20 mmol), 2,4,6-Me$_3$-C$_6$H$_2$CH$_2$-MgCl (0.60 mmol), 2.0 mL of PhMe. After 24 hours, the reaction afforded compound **3aa** with 99% isolated yield as a white solid. Optical Rotation: $[\alpha]^{20}{}_D$ = 8.2 (c 0.61, CH$_2$Cl$_2$). 99.5% ee, determined by HPLC, HPLC conditions: CHIRALPAK® IA, 5% i-PrOH in hexane, 1.00 mL min^{-1}, λ = 254 nm, τ_R (major) = 4.0 min; τ_R (minor) = 5.1 min. ^{1}H NMR (600 MHz, cdcl$_3$) δ 7.89 (d, *J* = 8.8 Hz, 1H), 7.83 – 7.71 (m, 3H), 7.32 (d, *J* = 8.9 Hz, 1H), 7.28 (d, *J* = 8.3 Hz, 1H), 7.19 (d, *J* = 8.3 Hz, 1H), 7.08 (s, 1H), 6.88 (d, *J* = 9.8 Hz, 2H), 6.84 (s, 2H), 4.79 (s, 1H), 3.62 (q, *J* = 17.8 Hz, 2H), 2.32 – 2.21 (m, 9H), 2.03 (s, 6H). ^{13}C NMR (151 MHz, cdcl$_3$) δ 150.83, 138.84, 137.17, 136.71, 136.48, 135.61, 133.40, 133.36, 132.94, 130.96, 129.57, 128.95, 128.82, 128.48, 128.15, 128.04, 127.96, 127.61, 125.78, 124.46, 124.09, 123.56, 117.01, 116.58, 32.71, 21.91, 21.71, 20.89, 19.87. HRMS (ESI) m/z [M-H]$^-$ calcd for [C$_{32}$H$_{29}$O]$^-$ 429.2224, found 429.2235.

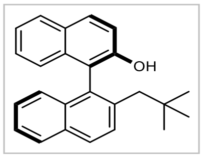

2'-neopentyl-[1,1'-binaphthalen]-2-ol (Compound **3ab**). Prepared according to the general procedure by using Ni(cod)$_2$ (5.6 mg, 0.02 mmol), **L1** (9.8 mg, 0.024 mmol), C-O electrophile (53.7 mg, 0.20 mmol), ^{t}BuCH$_2$MgBr (0.60 mmol), 2.0 mL of PhMe. After 24 hours, the reaction afforded compound **3ab** with 95% isolated yield as a white solid. Optical Rotation: $[\alpha]^{20}{}_D$ = 5.7 (c 0.53, CH$_2$Cl$_2$). 99% ee, determined by HPLC, HPLC conditions: CHIRALPAK® IA, 5% i-PrOH in hexane, 1.00 mL min^{-1}, λ = 254 nm, τ_R (minor) = 7.8 min; τ_R (major) = 8.4 min. ^{1}H NMR (600 MHz, cdcl$_3$) δ 7.91 (dd, *J* = 11.7, 8.6 Hz, 3H), 7.85 (d, *J* = 8.1 Hz, 1H), 7.62 (d, *J* = 8.5 Hz, 1H), 7.44 (t, *J* = 7.3 Hz, 1H), 7.35 – 7.17 (m, 5H), 7.02 (d, *J* = 8.5 Hz, 1H), 4.84 (s, 1H), 2.57 – 2.36 (m, 2H), 0.74 (s, 9H). ^{13}C NMR (151 MHz, cdcl$_3$) δ 151.07, 139.62, 133.78, 132.95, 132.73, 130.12, 129.93, 129.85, 129.07, 128.12, 128.08, 126.73, 126.40, 126.13, 125.72, 125.37, 123.28, 117.89, 117.37, 46.85, 32.85, 30.22. HRMS (ESI) m/z [M-H]$^-$ calcd for [C$_{25}$H$_{23}$O]$^-$ 339.1754, found 339.1760.

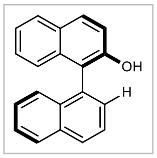

[1,1'-binaphthalen]-2-ol (Compound **3ac**). Prepared according to the general procedure by using Ni(cod)$_2$ (5.6 mg, 0.02 mmol), **L1** (9.8 mg, 0.024 mmol), C-O electrophile (53.7 mg, 0.20 mmol), cyclohexyl-MgBr (0.60 mmol), 2.0 mL of PhMe. After 24 hours, the reaction afforded compound **3ac** with 91% isolated yield as a white solid. Optical Rotation: $[\alpha]^{20}{}_D$ = -55.3 (c 0.47, CH$_2$Cl$_2$). 94% ee, determined by HPLC, HPLC conditions: CHIRALPAK® IA, 20% i-PrOH in hexane, 1.00 mL min^{-1}, λ = 254 nm, τ_R (minor) = 5.0 min; τ_R (major) = 5.7 min. ^{1}H NMR (600 MHz, cdcl$_3$) δ 7.95 (dd, *J* = 21.1, 8.3 Hz, 2H), 7.87 (d, *J* = 8.9 Hz, 1H), 7.83 (d, *J* = 8.2 Hz, 1H), 7.59 (t, *J* = 7.6 Hz, 1H), 7.49 (dd, *J* = 12.1, 7.2 Hz, 2H), 7.37 (d, *J* = 8.4 Hz, 1H), 7.34 – 7.26 (m, 3H), 7.22 – 7.17 (m, 1H), 7.08 (d, *J* = 8.5 Hz, 1H), 4.91 (s, 1H). ^{13}C NMR (101 MHz, CDCl$_3$) δ 150.94, 134.20, 133.89, 132.80, 131.42, 129.86, 129.63, 129.24, 128.92, 128.46, 127.99, 126.85, 126.54, 126.52, 126.01, 125.77, 124.95, 123.35, 118.74, 117.42. HRMS (ESI) m/z [M-H]$^-$ calcd for [C$_{20}$H$_{13}$O]$^-$ 269.0972, found 269.0974.

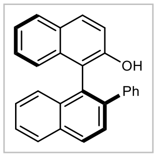

2'-phenyl-[1,1'-binaphthalen]-2-ol (Compound **3ad**). Prepared according to the general procedure by using Ni(cod)$_2$ (5.6 mg, 0.02 mmol), **L1** (9.8 mg, 0.024 mmol), C-O electrophile (53.7 mg, 0.20 mmol), Ph-MgCl (0.60 mmol), 2.0 mL of PhMe. After 24 hours, the reaction afforded compound **3ad** with 88% isolated yield as a white solid. Optical Rotation: $[\alpha]^{20}{}_D$ = -9.0 (c 0.63, CH_2Cl_2). 88% ee, determined by HPLC, HPLC conditions: CHIRALPAK® IA, 20% i-PrOH in hexane, 1.00 mL min^{-1}, λ = 254 nm, τ_R (major) = 4.8 min; τ_R (minor) = 5.6 min. ^{1}H NMR (600 MHz, cdcl$_3$) δ 8.03 (d, *J* = 8.3 Hz, 1H), 7.94 (d, *J* = 8.2 Hz, 1H), 7.73 (d, *J* = 8.5 Hz, 2H), 7.66 (d, *J* = 8.6 Hz, 1H), 7.52 – 7.44 (m, 1H), 7.32 – 7.26 (m, 2H), 7.23 (t, *J* = 7.4 Hz, 1H), 7.21 – 7.15 (m, 1H), 7.15 – 7.06 (m, 4H), 7.05 – 6.99 (m, 3H), 4.86 (s, 1H); ^{13}C NMR (150 MHz, cdcl$_3$) δ 150.99, 141.59, 140.82, 134.16, 133.21, 133.17, 129.81, 129.32, 128.72, 128.62, 128.57, 128.54, 128.16, 128.03, 127.62, 127.15, 126.92, 126.53, 126.37, 126.33, 125.00, 123.16, 117.72, 117.17. The spectral data match those previously reported in the literature.

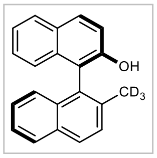

2'-(methyl-d^3)-[1,1'-binaphthalen]-2-ol (Compound **3ae**). Prepared according to the general procedure by using Ni(cod)$_2$ (5.6 mg, 0.02 mmol), **L1** (9.8 mg, 0.024 mmol), C-O electrophile (53.7 mg, 0.20 mmol), CD_3-MgBr (0.80 mmol), $MgBr_2$ (5.0 equiv), 2.0 mL of PhMe. After 24 hours, the reaction afforded compound **3ae** with 55% isolated yield as a white solid. Optical Rotation: $[\alpha]^{20}{}_D$ = -88 (c 0.25, CH_2Cl_2). 89% ee, determined by HPLC, HPLC conditions: CHIRALPAK® IA, 10% i-PrOH in hexane, 1.00 mL min^{-1}, λ = 254 nm, τ_R (minor) = 5.1 min; τ_R (major) = 5.5 min. ^{1}H NMR (600 MHz, cdcl$_3$) δ 7.92 – 7.87 (m, 3H), 7.85 (d, *J* = 8.1 Hz, 1H), 7.51 (d, *J* = 8.4 Hz, 1H), 7.45 – 7.39 (m, 1H), 7.35 – 7.27 (m, 2H), 7.27 – 7.23 (m, 1H), 7.23 – 7.16 (m, 2H), 6.96 (d, *J* = 8.5 Hz, 1H), 4.76 (s, 1H). ^{13}C NMR (151 MHz, cdcl$_3$) δ 150.76, 137.05, 133.37, 133.27, 132.60, 129.76, 129.20, 128.98, 128.94, 128.84, 128.15, 128.11, 126.82, 126.67, 125.52, 125.44, 124.51, 123.39, 117.63, 117.41. HRMS (ESI) m/z [M-H]$^-$ calcd for $[C_{21}H_{12}D_3O]^-$ 286.1317, found 286.1320.

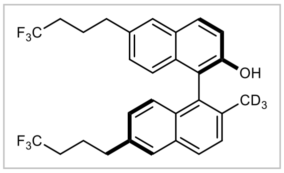

2'-(methyl-d3)-6,6'-bis(4,4,4-trifluorobutyl)-[1,1'-binaphthalen]-2-ol (Compound **3af**). Prepared according to the general procedure by using Ni(cod)$_2$ (5.6 mg, 0.02 mmol), **L1** (9.8 mg, 0.024 mmol), C-O electrophile (97.7 mg, 0.20 mmol), CD_3-MgBr (0.80 mmol), $MgBr_2$ (5.0 equiv), PhMe (2.0 mL). After 24 hours, the reaction afforded compound **3af** with 49% isolated yield as a white solid. Optical Rotation: $[\alpha]^{20}{}_D$ = -80 (c 0.1, CH_2Cl_2). 85% ee, determined by HPLC, HPLC conditions: CHIRALPAK® IA, 10% i-PrOH in hexane, 1.00 mL min^{-1}, λ = 254 nm, τ_R (minor) = 6.1 min; τ_R

(major) = 6.8 min. ^{1}H NMR (600 MHz, cdcl$_3$) δ 7.86 (dd, *J* = 16.9, 8.7 Hz, 2H), 7.68 (s, 1H), 7.64 (s, 1H), 7.53 (d, *J* = 8.4 Hz, 1H), 7.33 (d, *J* = 8.9 Hz, 1H), 7.16 (d, *J* = 8.7 Hz, 1H), 7.11 (d, *J* = 8.6 Hz, 1H), 7.06 (d, *J* = 8.6 Hz, 1H), 6.92 (d, *J* = 8.6 Hz, 1H), 4.71 (s, 1H), 2.80 (dt, *J* = 15.0, 7.6 Hz, 4H), 2.17 – 2.05 (m, 4H), 1.99 – 1.90 (m, 4H). ^{13}C NMR (151 MHz, cdcl$_3$) δ 150.48, 137.91, 136.54, 135.61, 132.79, 132.05, 132.00, 129.31, 129.29, 129.25, 128.58, 127.92, 127.77, 127.23 (q, *J* = 276.1 Hz), 127.10 (q, *J* = 276.1 Hz), 126.95, 126.87, 125.81, 124.88, 117.64, 117.62, 34.57, 34.52, 33.20 (q, *J* = 28.5 Hz), 33.17 (q, *J* = 28.5 Hz), 23.35 (q, *J* = 11.7 Hz), 23.32 (q, *J* = 11.7 Hz),. ^{13}C NMR (151 MHz, cdcl$_3$) δ 150.47, 137.90, 136.63, 135.61, 132.75, 132.02, 132.00, 129.29, 129.24, 128.70, 128.56, 127.92, 127.77, 127.23 (q, *J* = 276.1 Hz), 127.10 (q, *J* = 276.1 Hz), 126.95, 126.86, 125.80, 124.86, 117.62, 34.55, 34.50, 33.21 (q, *J* = 28.5 Hz), 33.17 (q, *J* = 28.5 Hz), 23.34 (q, *J* = 11.7 Hz), 23.32 (q, *J* = 11.7 Hz), 20.00. HRMS (APCI) m/z [M-H]$^-$ calcd for $[C_{29}H_{22}D_3F_6O]^-$ 506.2003, found 506.1937.

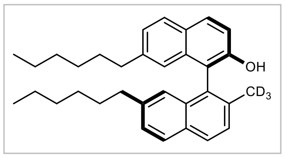

7,7'-dihexyl-2'-(methyl-d3)-[1,1'-binaphthalen]-2-ol (Compound **3ag**). Prepared according to the general procedure by using Ni(cod)$_2$ (5.6 mg, 0.02 mmol), **L1** (9.8 mg, 0.024 mmol), C-O electrophile (87.3 mg, 0.20 mmol), CD$_3$-MgBr (0.80 mmol), MgBr$_2$ (5.0 equiv), PhMe (2.0 mL). After 24 hours, the reaction afforded compound **3ag** with 55% isolated yield as a colorless oil. Optical Rotation: $[\alpha]^{20}_{D}$ = -178.3 (c 0.23, CH$_2$Cl$_2$). 96% ee, determined by HPLC, HPLC conditions: CHIRALPAK® IA, 5% i-PrOH in hexane, 1.00 mL min^{-1}, λ = 254 nm, τ_R (minor) = 4.1 min; τ_R (major) = 4.8 min. ^{1}H NMR (600 MHz, cdcl$_3$) δ 7.91 – 7.81 (m, 3H), 7.79 (d, *J* = 8.3 Hz, 1H), 7.48 (d, *J* = 8.4 Hz, 1H), 7.28 (dd, *J* = 17.9, 8.6 Hz, 2H), 7.17 (d, *J* = 8.4 Hz, 1H), 7.00 (s, 1H), 6.75 (s, 1H), 4.70 (s, 1H), 2.49 (dt, *J* = 15.8, 7.7 Hz, 4H), 1.49 – 1.40 (m, 4H), 1.21 – 1.11 (m, 12H), 0.82 – 0.74 (m, 6H). ^{13}C NMR (151 MHz, cdcl$_3$) δ 150.80, 141.58, 141.35, 137.00, 133.54, 133.39, 131.09, 129.34, 128.60, 128.47, 128.13, 128.00, 127.99, 127.65, 127.01, 124.86, 123.94, 123.20, 117.45, 116.41, 36.10, 31.59, 31.33, 31.28, 28.80, 28.76, 22.53, 22.51, 13.98. HRMS (ESI) m/z [M-H]$^-$ calcd for $[C_{33}H_{36}D_3O]^-$ 454.3195, found 454.3196.

5. Supplementary figures of determination of absolute stereochemistry

The absolute stereochemistry of compound **3o** was determined by chiral HPLC comparison with authentic samples prepared from commercially available (*R*)-BINOL and (*S*)-BINOL respectively. The optical rotation of **3o** is consistent with the value reported in the literature. [5]

Chiral HPLC: (CHIRALPAK® IA, 10% ⁱPrOH in hexane, 1.00 mL/min, λ = 254 nm), τ_R = 6.1 min; τ_R = 7.8 min.

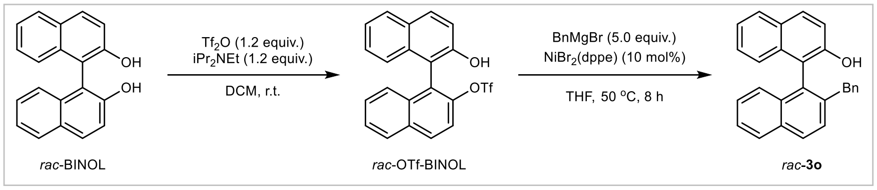

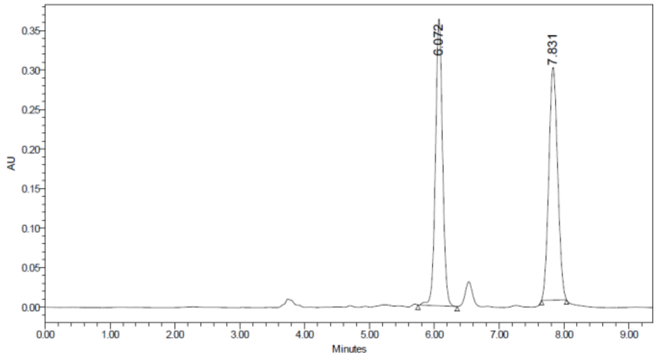

	RT (min)	Peak Type	Area (µV*sec)	% Area	Height (µV)	% Height	Integration Type	Points Across Peak	Start Time (min)	End Time (min)
1	6.072	Unknown	2762750	49.73	363265	55.24	bb	364	5.747	6.353
2	7.831	Unknown	2792461	50.27	294383	44.76	bb	231	7.653	8.038

Supplementary Figure 17. Racemic **3o** prepared from commercially available (rac)-BINOL

Chiral HPLC: (CHIRALPAK® IA, 10% ⁱPrOH in hexane, 1.00 mL/min, λ = 254 nm), τ_R (major) = 6.1 min; τ_R (minor) = 7.8 min.

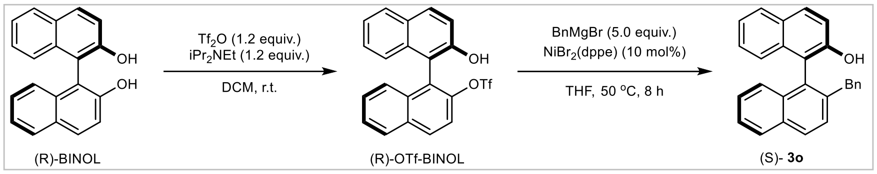

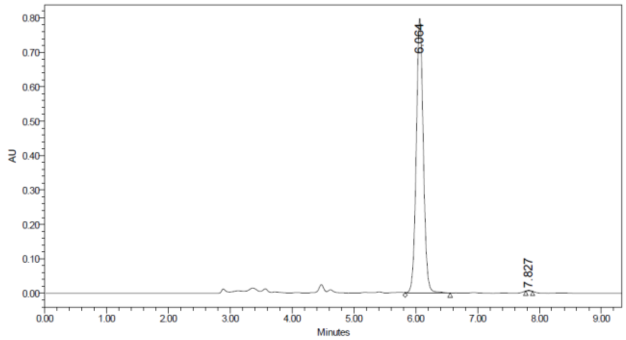

	RT (min)	Peak Type	Area (µV*sec)	% Area	Height (µV)	% Height	Integration Type	Points Across Peak	Start Time (min)	End Time (min)
1	6.064	Unknown	6304137	99.83	797390	99.69	VB	436	5.828	6.555
2	7.827	Unknown	10568	0.17	2509	0.31	bb	66	7.778	7.888

Supplementary Figure 18. Compound (S)-**3o** prepared from commercially available (R)-BINOL

Chiral HPLC: (CHIRALPAK® IA, 10% ⁱPrOH in hexane, 1.00 mL/min, λ = 254 nm), τ_R (minor) = 6.0 min; τ_R (major) = 7.7 min.

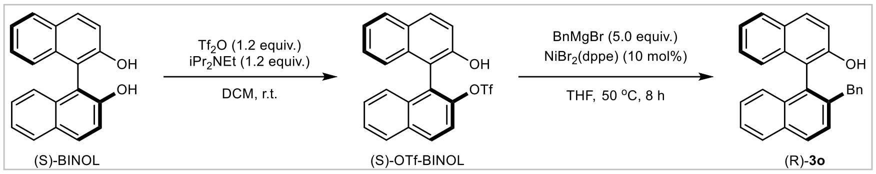

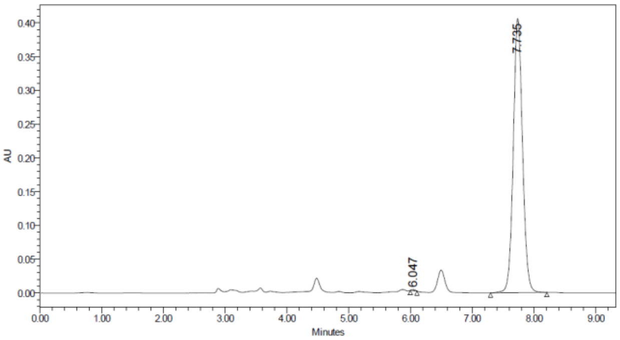

	RT (min)	Peak Type	Area (µV*sec)	% Area	Height (µV)	% Height	Integration Type	Peak Codes	Points Across Peak	Start Time (min)
1	6.047	Unknown	6532	0.15	1697	0.42	bb	I08	64	5.997
2	7.735	Unknown	4221176	99.85	405993	99.58	BB		548	7.295

Supplementary Figure 19. Compound (R)-**3o** prepared from commercially available (S)-BINOL

Chiral HPLC: (CHIRALPAK® IA, 10% ⁱPrOH in hexane, 1.00 mL/min, λ = 254 nm), τ_R (major) = 6.1 min; τ_R (minor) = 7.8 min.

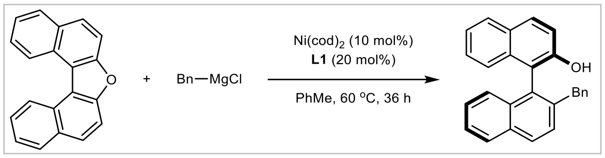

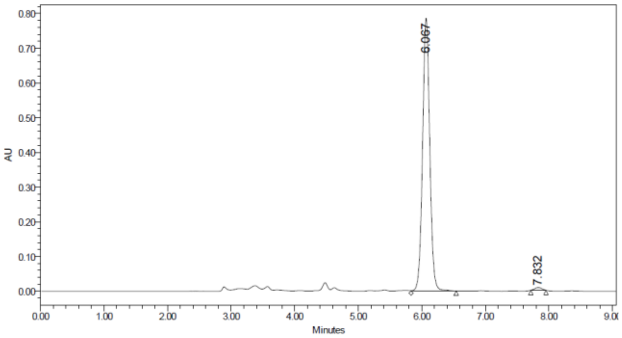

	RT (min)	Peak Type	Area (µV*sec)	% Area	Height (µV)	% Height	Integration Type	Points Across Peak	Start Time (min)	End Time (min)
1	6.067	Unknown	6181429	99.02	785717	99.02	VB	426	5.832	6.542
2	7.832	Unknown	61238	0.98	7812	0.98	bb	143	7.715	7.953

Supplementary Figure 20. Compound (S)-**3o** prepared from this work

6. Supplementary tables of mechanistic investigation

Supplementary Table 1. The effect of ligand loading in Ni/**L1** catalyzed enantioselective methylative cross-coupling

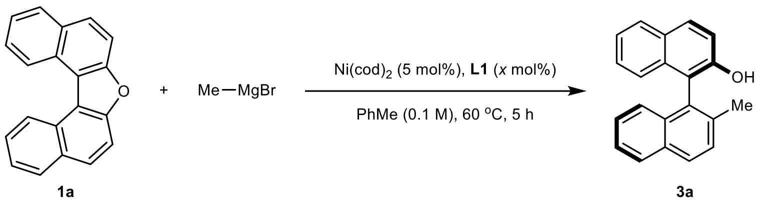

entries	**L1** (*x* mol%)	conversion	ee
1	0	< 10%	n.d.
2	6	76%	77%
3	10	96%	94%
4	15	97%	97%
5	20	99%	99%

• Higer conversion and ee were obtained in the presence of high loading of ligand (**L1**)

Supplementary Table 2. The effect of ligand loading in Ni/**L2** catalyzed enantioselective methylative cross-coupling

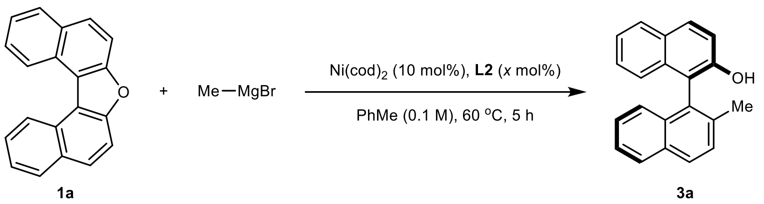

entries	**L2** (*x* mol%)	conversion	ee
1	0	< 5%	n.d.
2	12	< 10%	n.d.
3	20	< 10%	n.d.
4	30	< 10%	n.d.
5	40	< 10%	n.d.

• No conversion were observed in the presence of various loading of ligand (**L2**)

Supplementary Table 3. The effect of ligand loading in nickel catalyzed enantioselective arylative cross-coupling

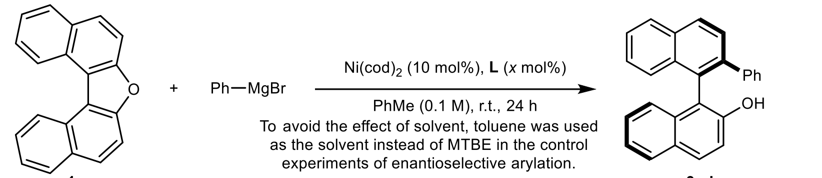

1a 3ad

entries	L (*x* mol%)	conversion	ee
1	**L2** (12 mol%)	91%	80%
2	**L2** (20 mol%)	77%	79%
3	**L2** (30 mol%)	66%	78%
4	**L2** (40 mol%)	59%	78%
5	**L1** (12 mol%)	95%	25%
6	**L1** (20 mol%)	67%	23%
7	**L1** (30 mol%)	57%	19%
8	**L1** (40 mol%)	51%	18%

From the above control experiments, we could find that lower conversion will be obtained for the enantioselective arylative cross-coupling when high loading of ligands, including **L1** and **L2**, were used. Thus, indicating that mono-ligated nickel species might serve as catalytically species in nickel catalyzed arylative cross-coupling. Moreover, combined theoretical and experimental studies of nickel-catalyzed arylative cross-coupling of arylmethyl ether via aromatic C-O bond activation were performed by the groups of Chatani and Mori in 2017, and they found that the oxidative addition of aromatic C-O bond was better mediated by bis-ligated nickel complex Ni(**L**)$_2$ than by mono-ligated nickel complex Ni(**L**). However, due to the steric effect, energy barrier for transmetallation of phenyl nucleophile with bis-ligated nickel species Ni(**L**)$_2$(Ar)(OMe) was much higher than that for mono-ligated nickel species Ni(**L**)(Ar)(OMe), and bis-ligated nickel species Ni(**L**)$_2$(Ar)(OMe) need occur one ligand dissociation in the transmetallation step. Thus, high concentration of ligand would inhibit the ligand dissociation and limit the transmetallation step.

7. Supplementary figures of computational details

All DFT calculations were performed by Gaussian 16 program.[6] The geometry optimizations were calculated using the B3LYP functional,[7,8] with LanL2DZ basis set[9-11] for nickel and 6-31G(d) basis set for the other atoms. The vibrational frequencies were computed at the same level of theory as for the geometry optimizations to confirm each optimized structure as an energy minimum (no imaginary frequency) or a transition state (one imaginary frequency) and obtain the thermodynamic corrections to Gibbs free energy. On the basis of the gas-phase optimized structures, the single-point energies and solvent effects were calculated with M06 functional,[12] SDD basis set[13] for Ni atom, and 6-311+G(d,p) basis set for the other atoms. The solvation energies (in toluene) were calculated using the self-consistent reaction field with the CPCM implicit solvent model.[14] The 3D diagrams of computed species were generated using CYLView.[15] The

theoretical strategy adopted here has been successfully applied in similar Ni-catalyzed reactions.[16,17] In this work, relative Gibbs free energies in toluene solution (including gas-phase corrections) were used to discuss reaction pathways.

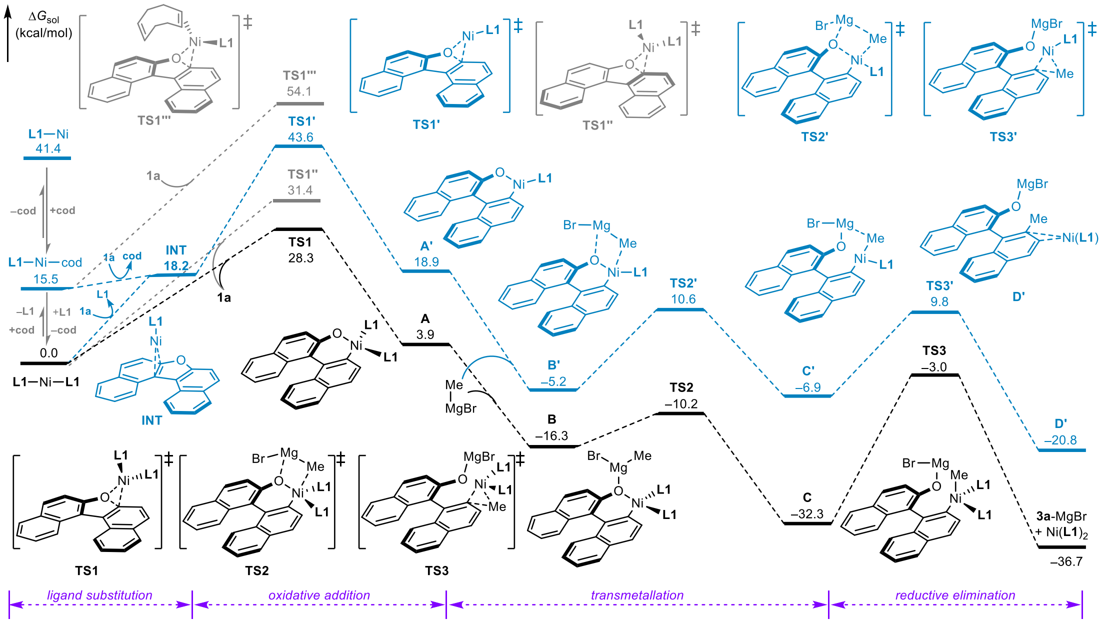

Supplementary Figure 21. Computational analysis of the mechanism for Ni(cod)$_2$/**L1** catalyzed enantioselective methylation of aromatic C–O bond. Relative Gibbs free energies (kcal/mol) in solution are calculated at the level of M06/6-311+G(d,p)-SDD (CPCM, toluene)//B3LYP/6-31G(d)-LanL2DZ.

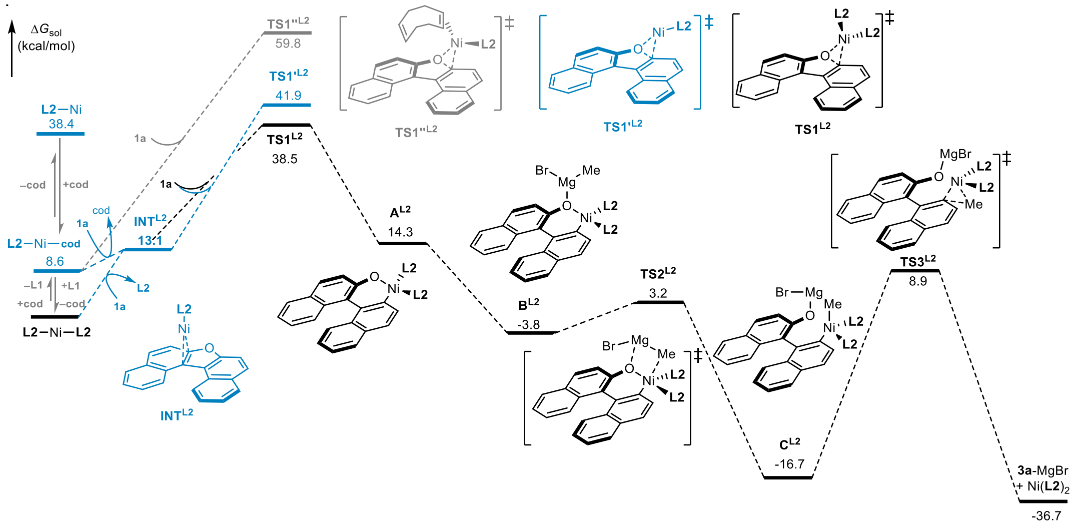

Supplementary Figure 22. Computational analysis of the mechanism for Ni(cod)$_2$/**L2** catalyzed enantioselective methylation of aromatic C–O bond. Relative Gibbs free energies (kcal/mol) in solution are calculated at the level of M06/6-311+G(d,p)-SDD (CPCM, toluene)//B3LYP/6-31G(d)-LanL2DZ.

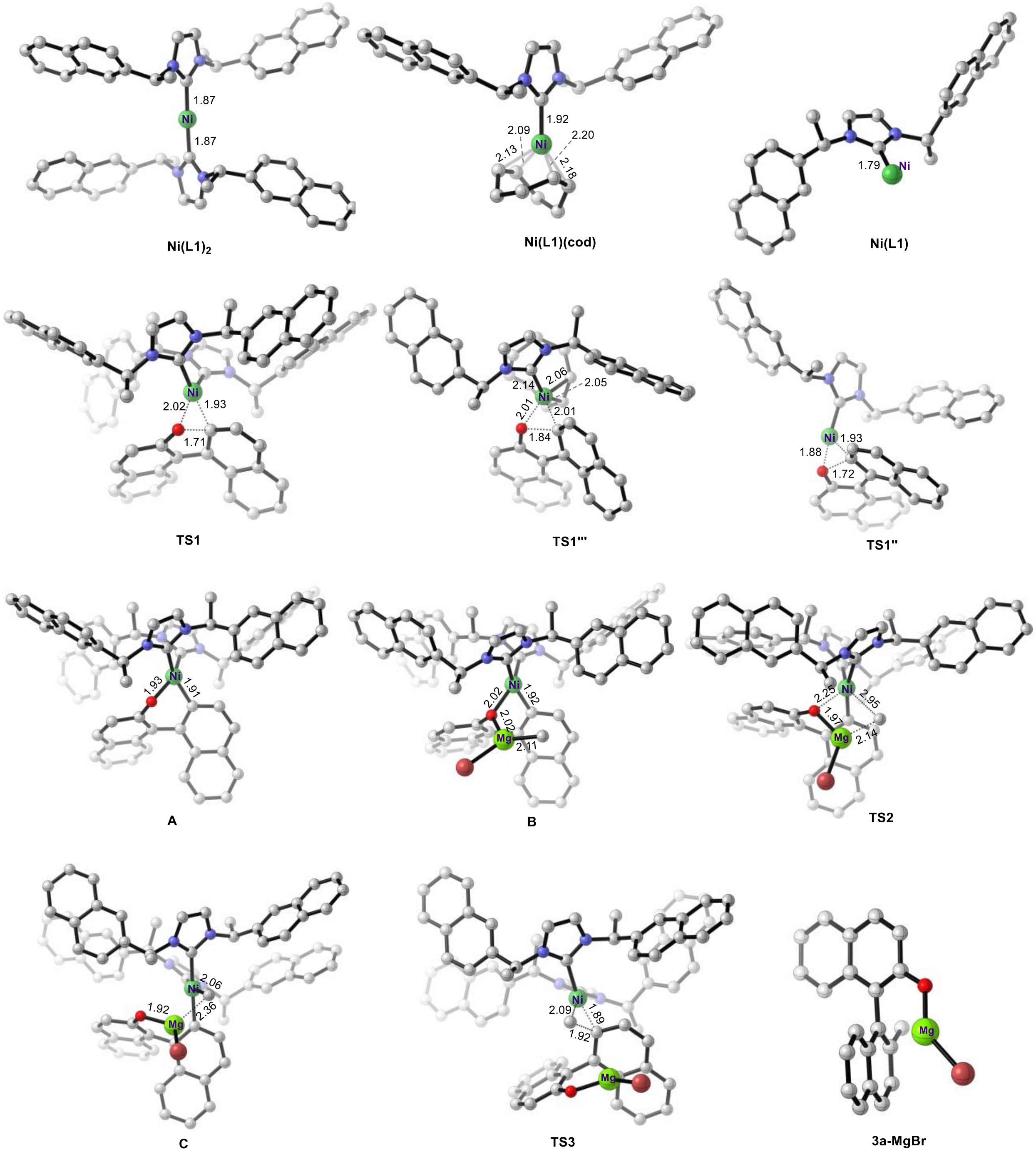

Supplementary Figure 23. Optimized structures along with the favorable pathway involved in Ni(cod)$_2$/**L1** catalyzed enantioselective methylation of substrate **1a**. All hydrogen atoms were omitted for clarity. Bond distances are given in Å.

8. Supplementary figures of NMR spectra of products

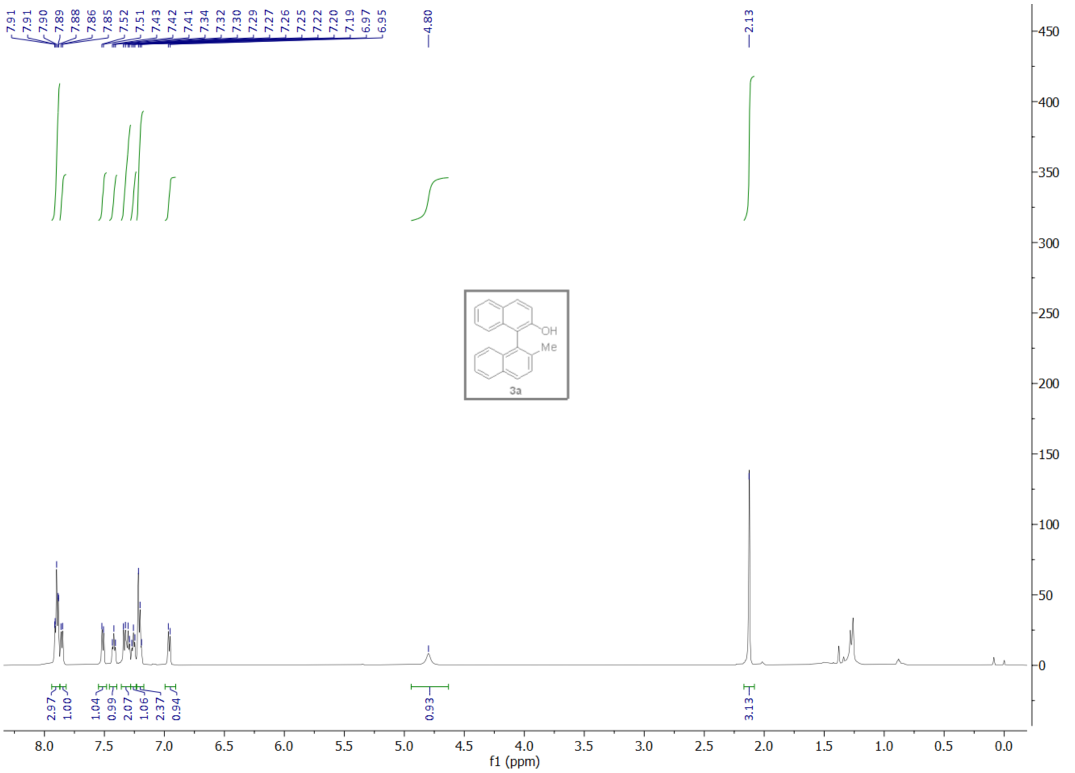

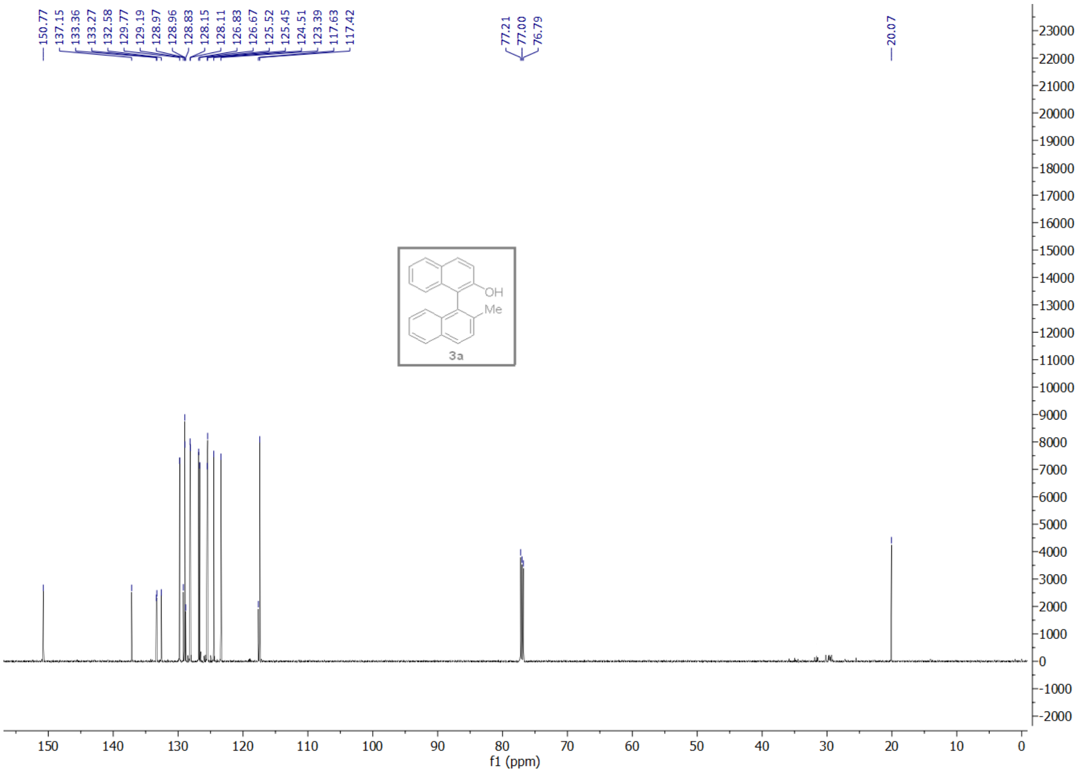

Supplementary Figure 24. ^{1}H NMR and ^{13}C NMR spectrum of **3a**.

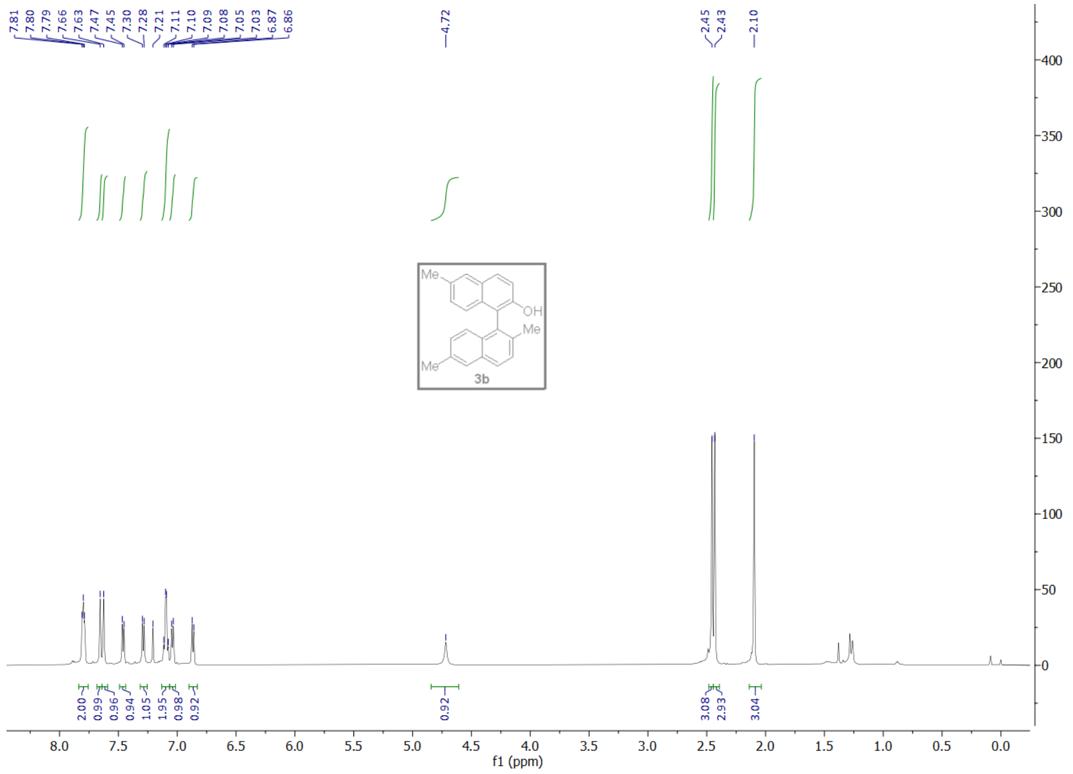

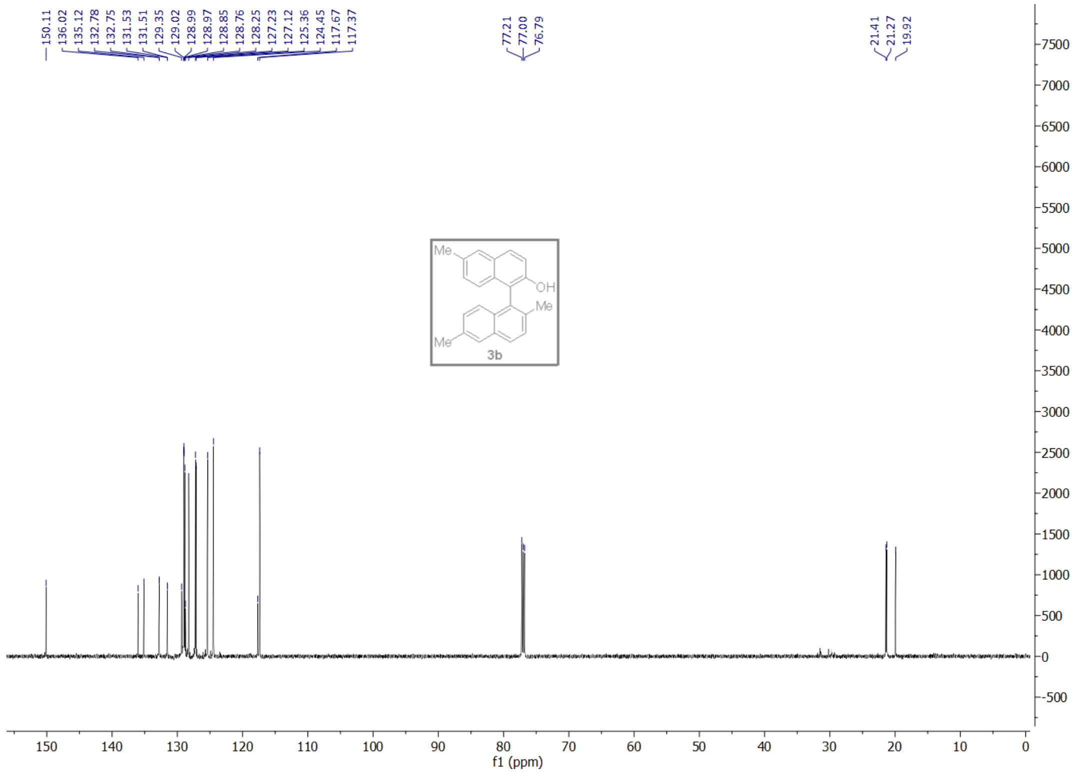

Supplementary Figure 25. ^{1}H NMR and ^{13}C NMR spectrum of **3b**.

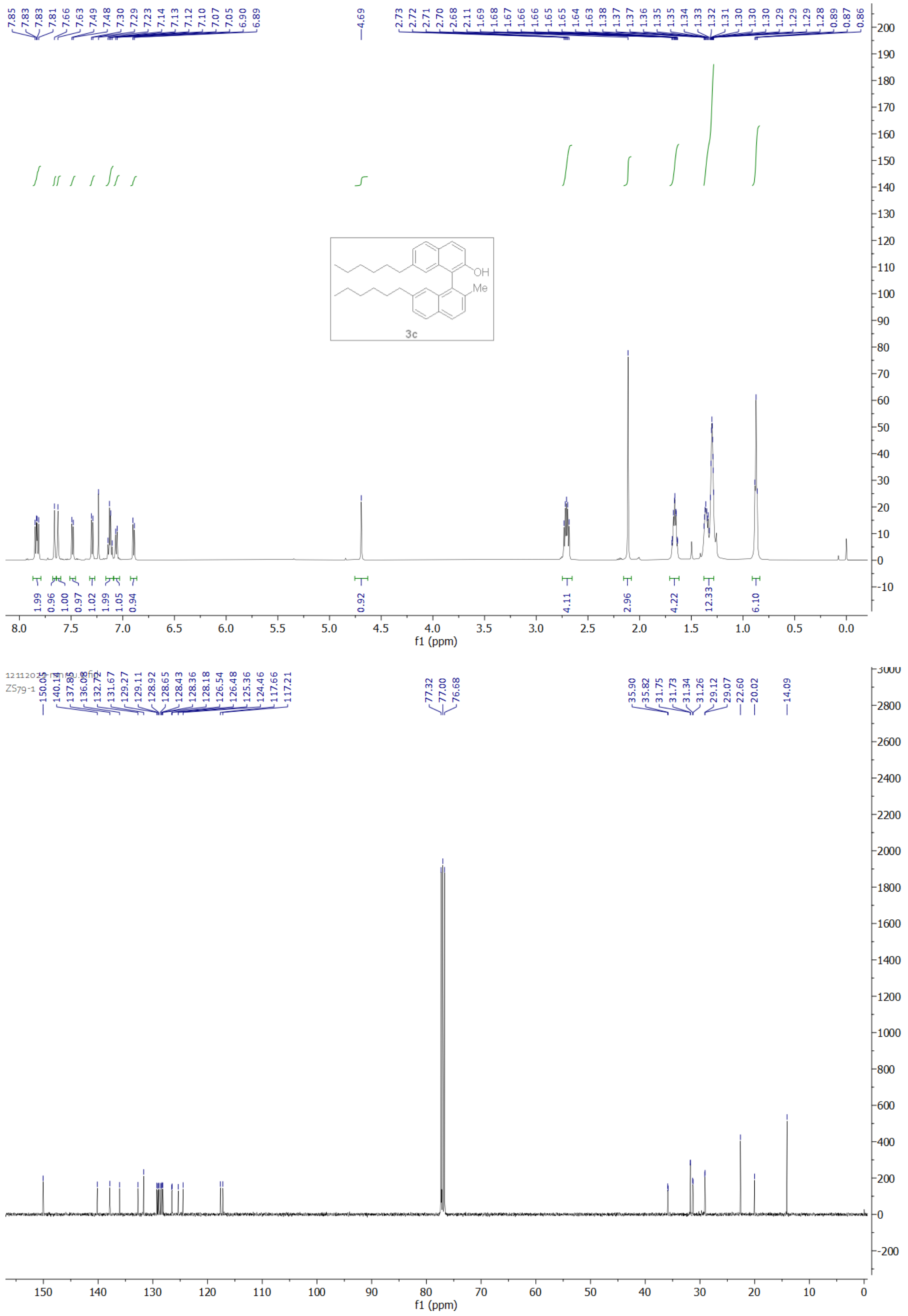

Supplementary Figure 26. ^{1}H NMR and ^{13}C NMR spectrum of **3c**.

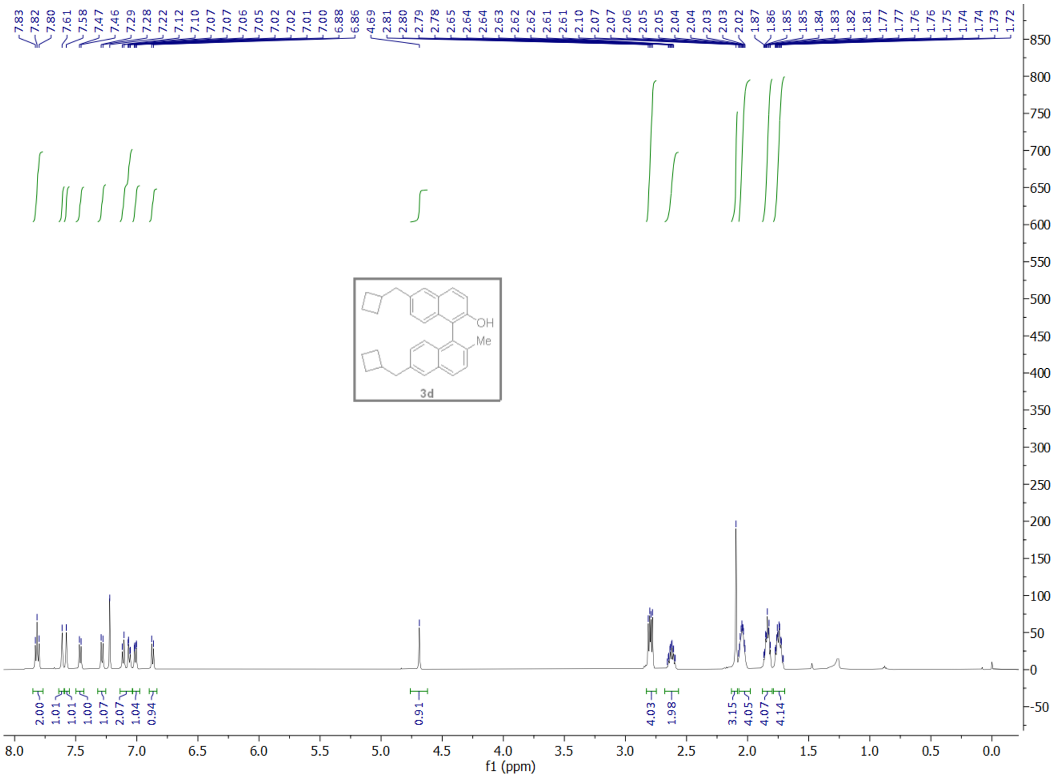

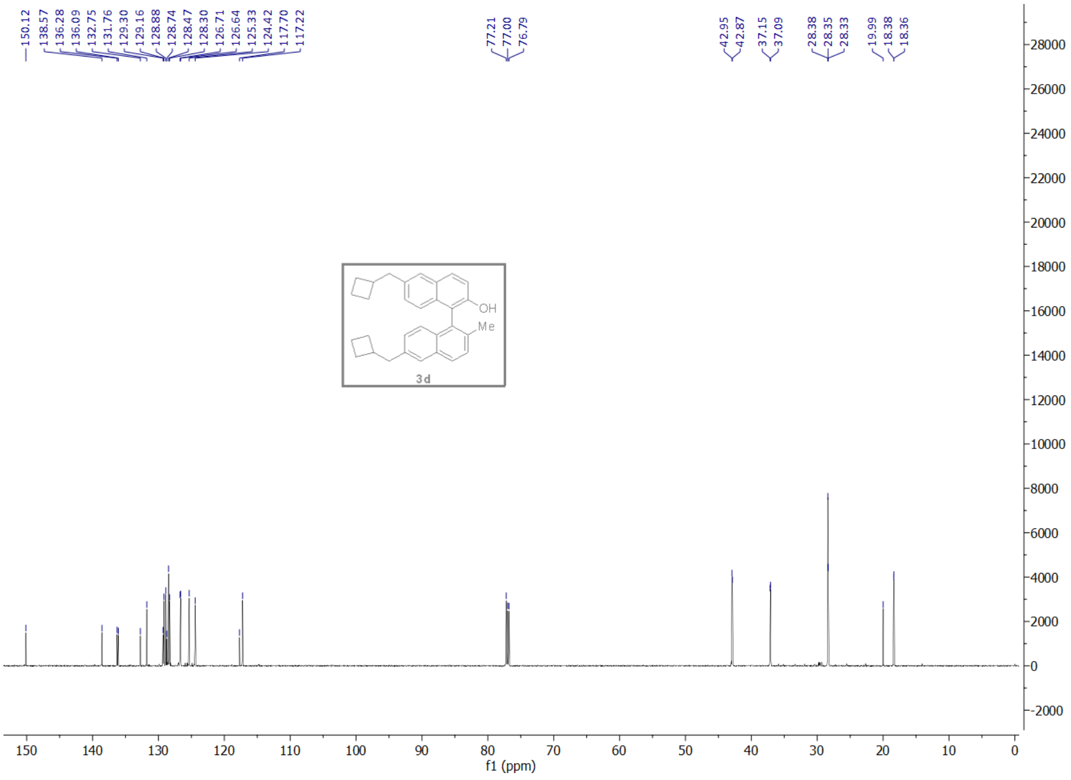

Supplementary Figure 27. ^{1}H NMR and ^{13}C NMR spectrum of **3d**.

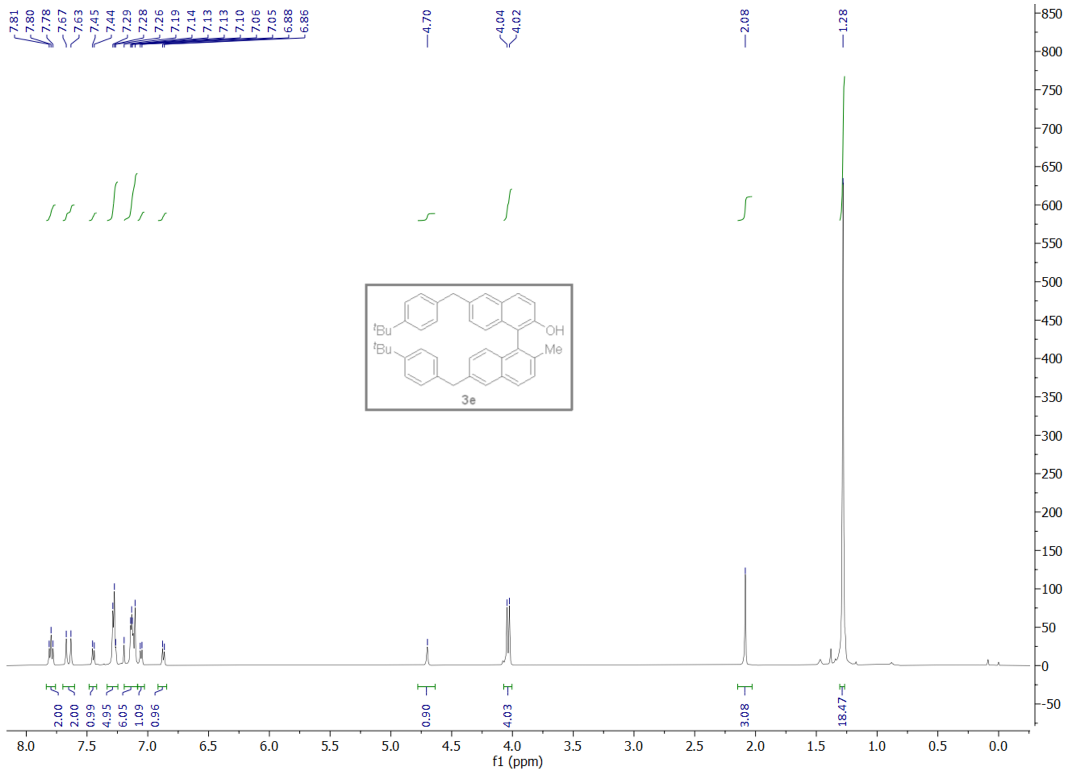

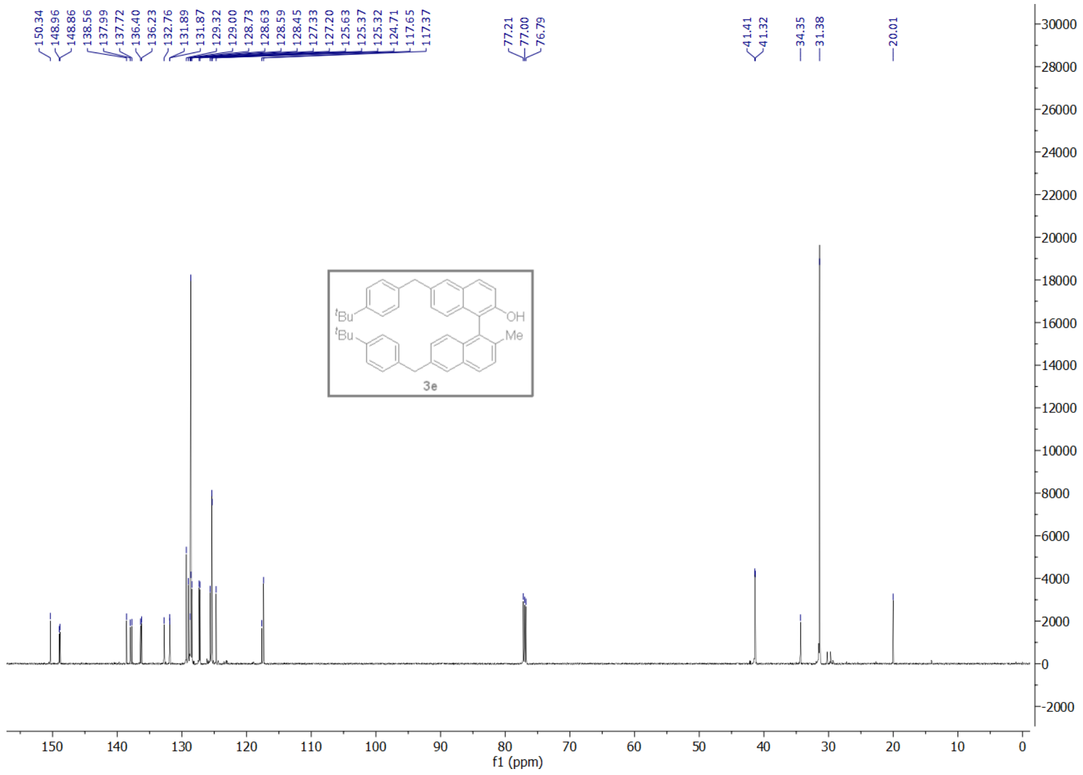

Supplementary Figure 28. ^{1}H NMR and ^{13}C NMR spectrum of **3e**.

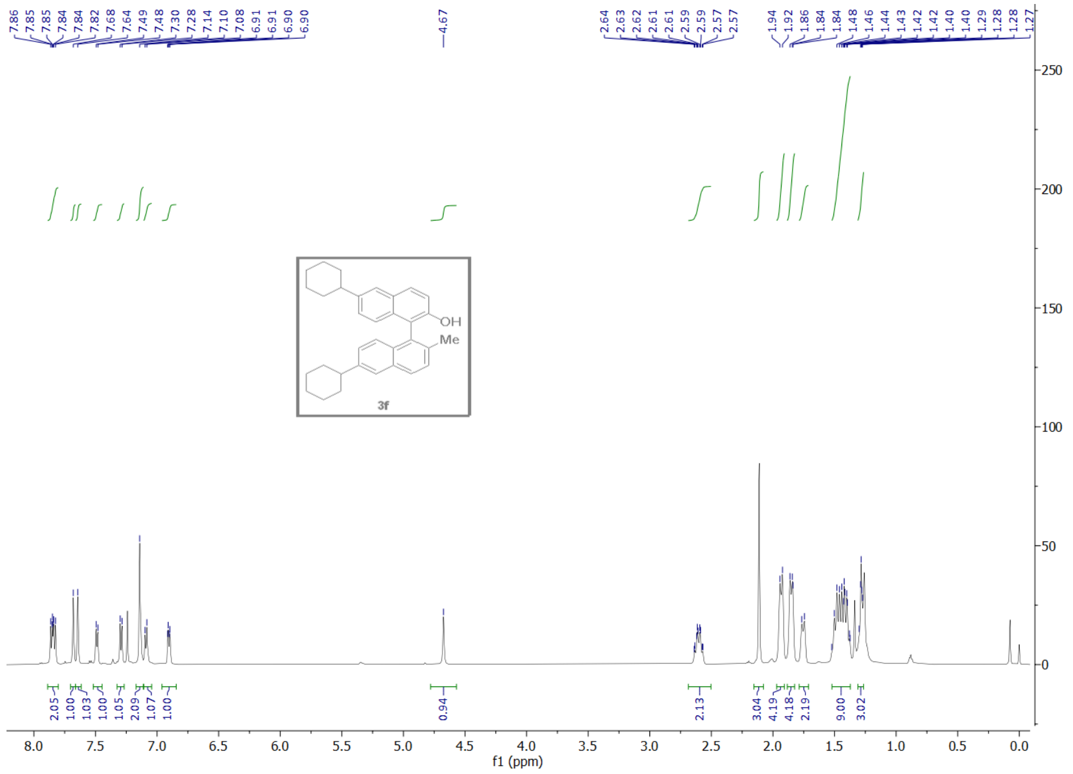

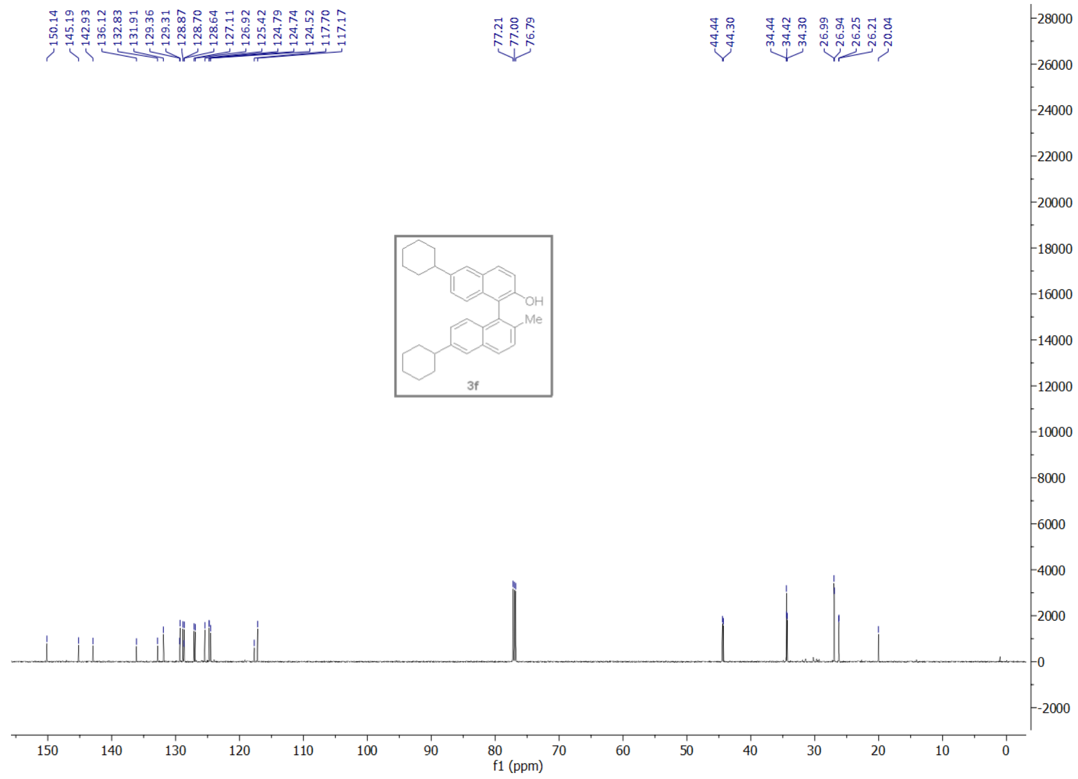

Supplementary Figure 29. ^{1}H NMR and ^{13}C NMR spectrum of **3f**.

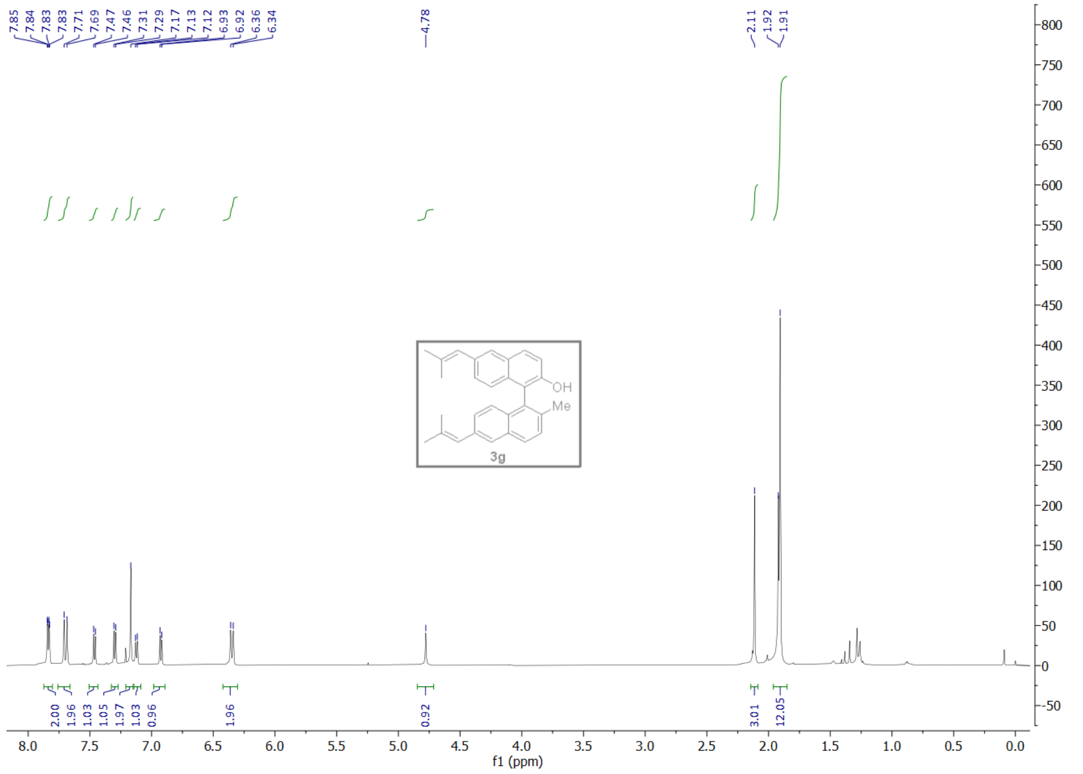

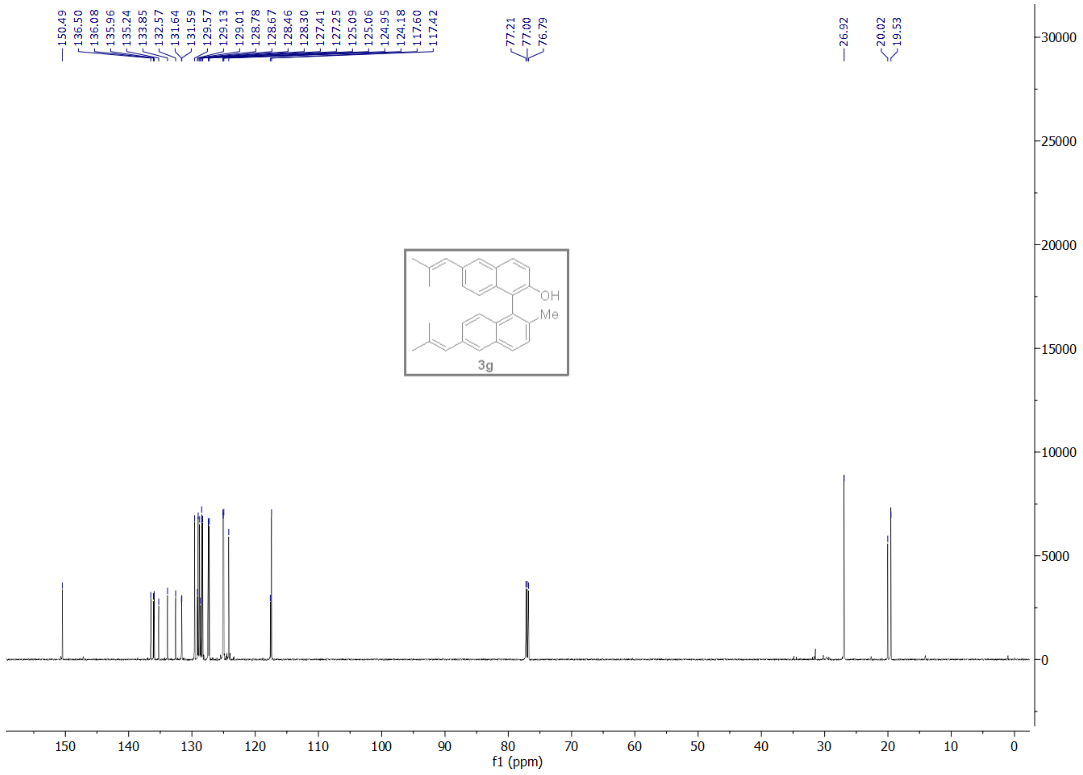

Supplementary Figure 30. ^{1}H NMR and ^{13}C NMR spectrum of **3g**.

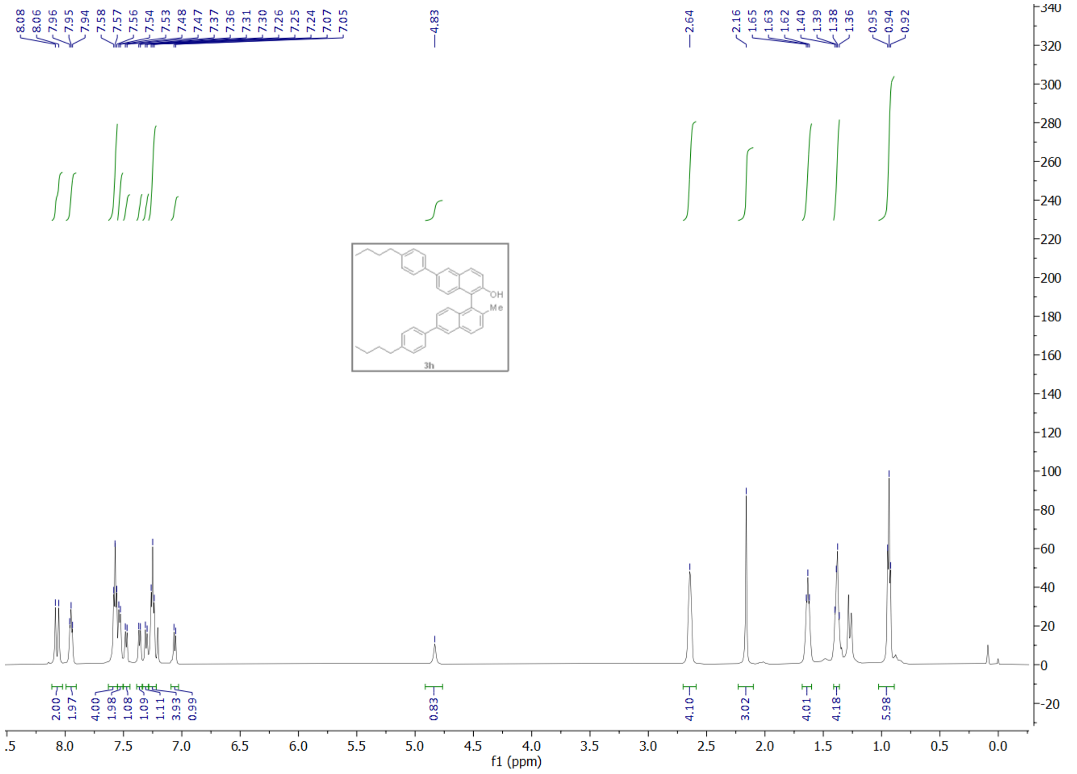

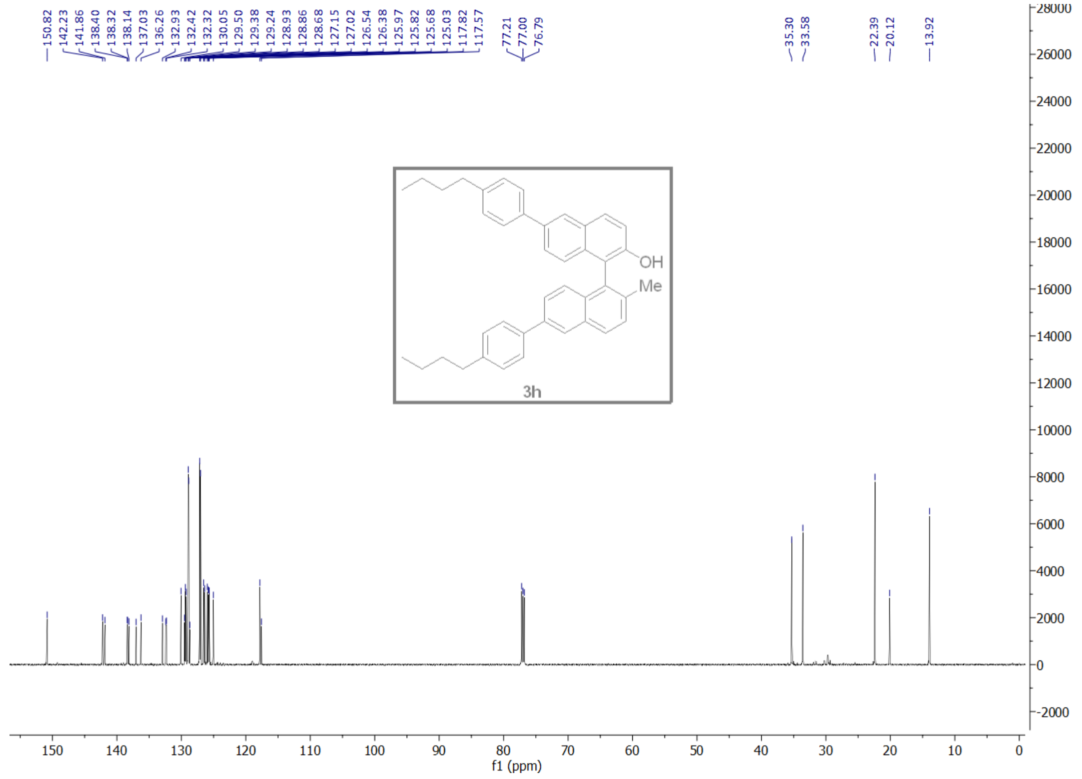

Supplementary Figure 31. ^{1}H NMR and ^{13}C NMR spectrum of **3h**.

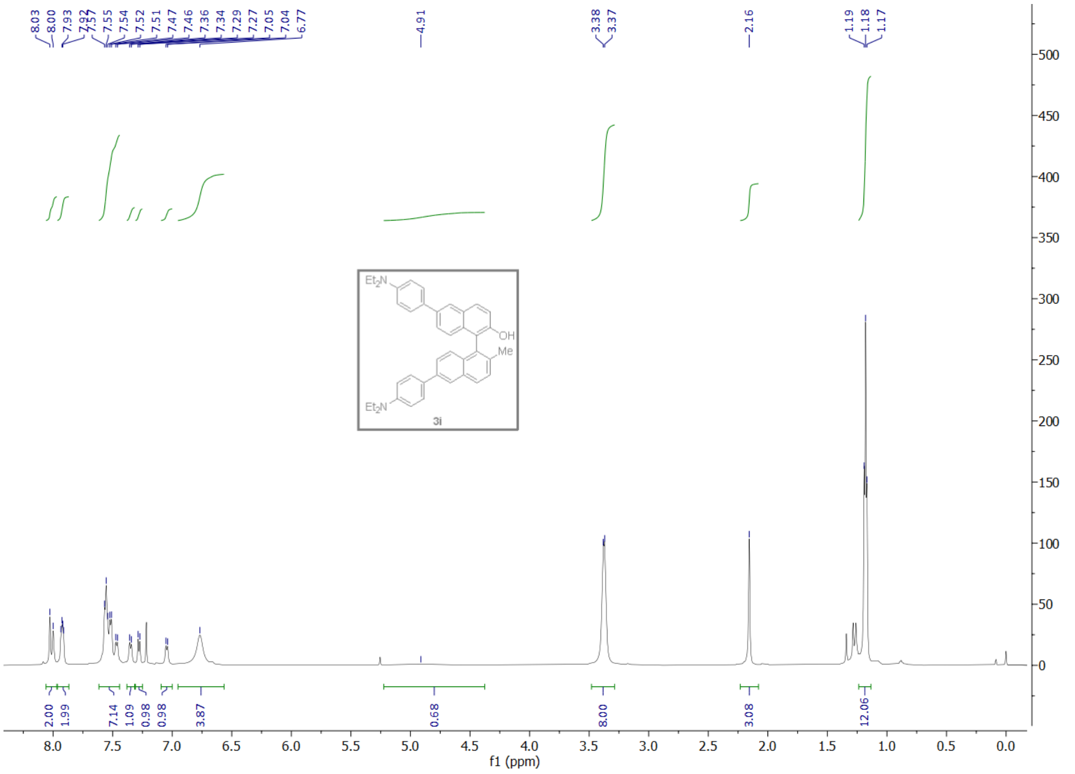

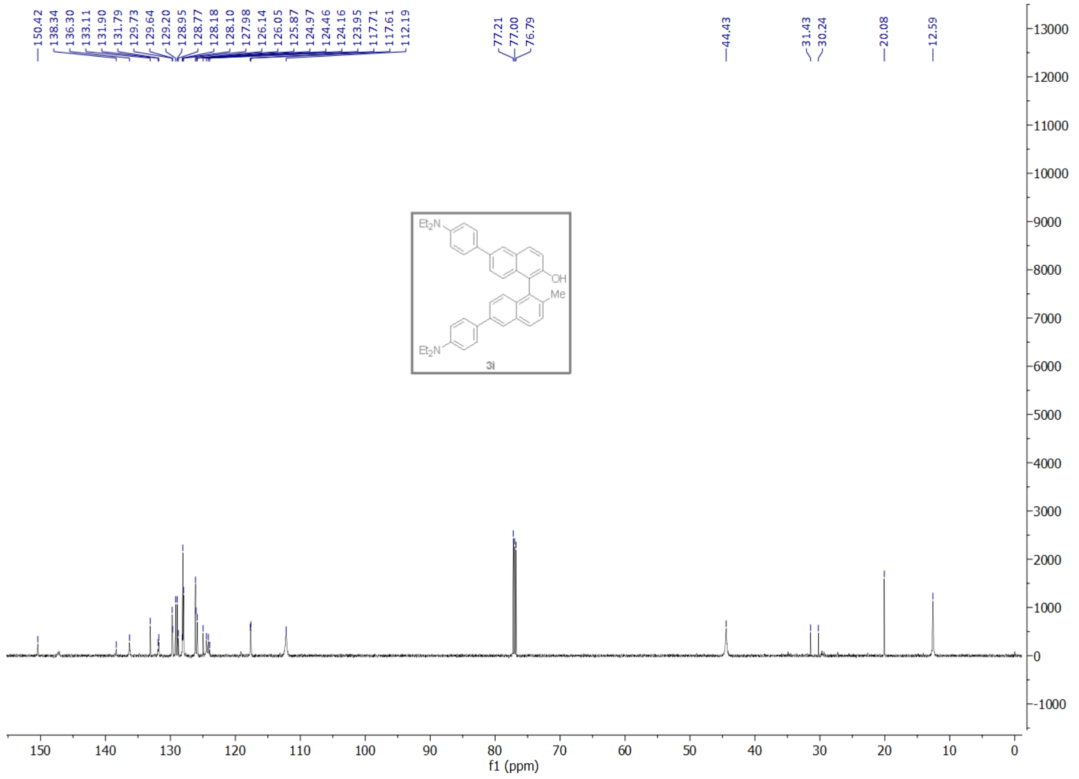

Supplementary Figure 32. ^{1}H NMR and ^{13}C NMR spectrum of **3i**.

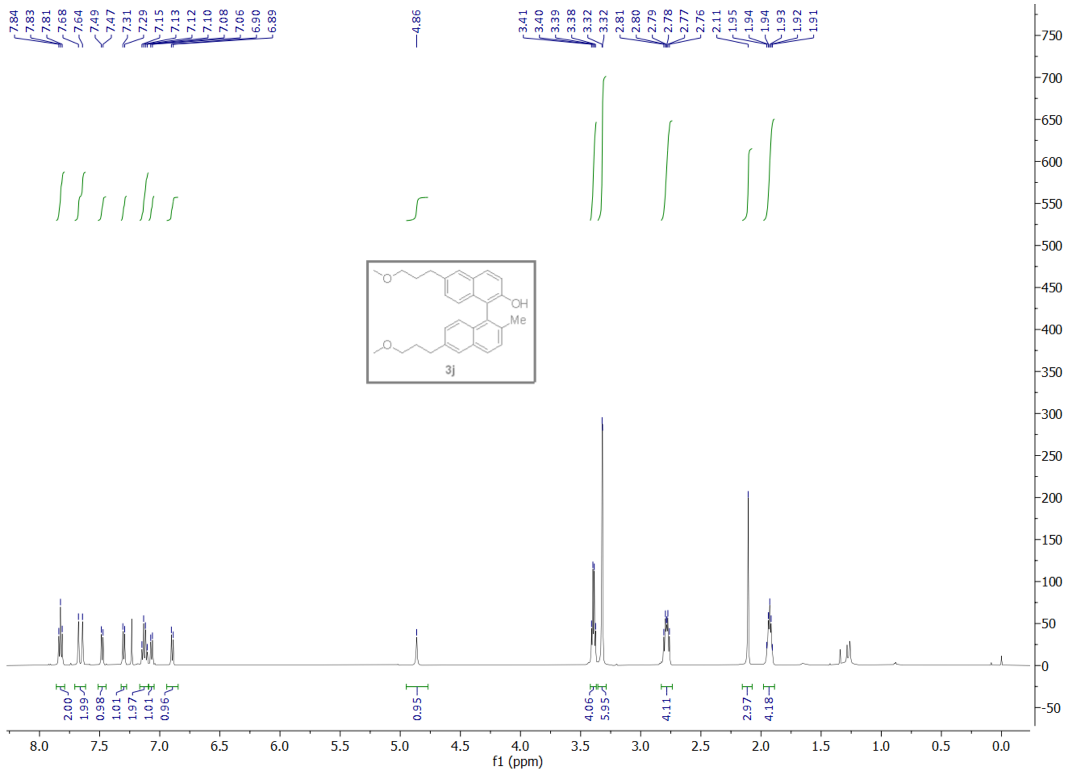

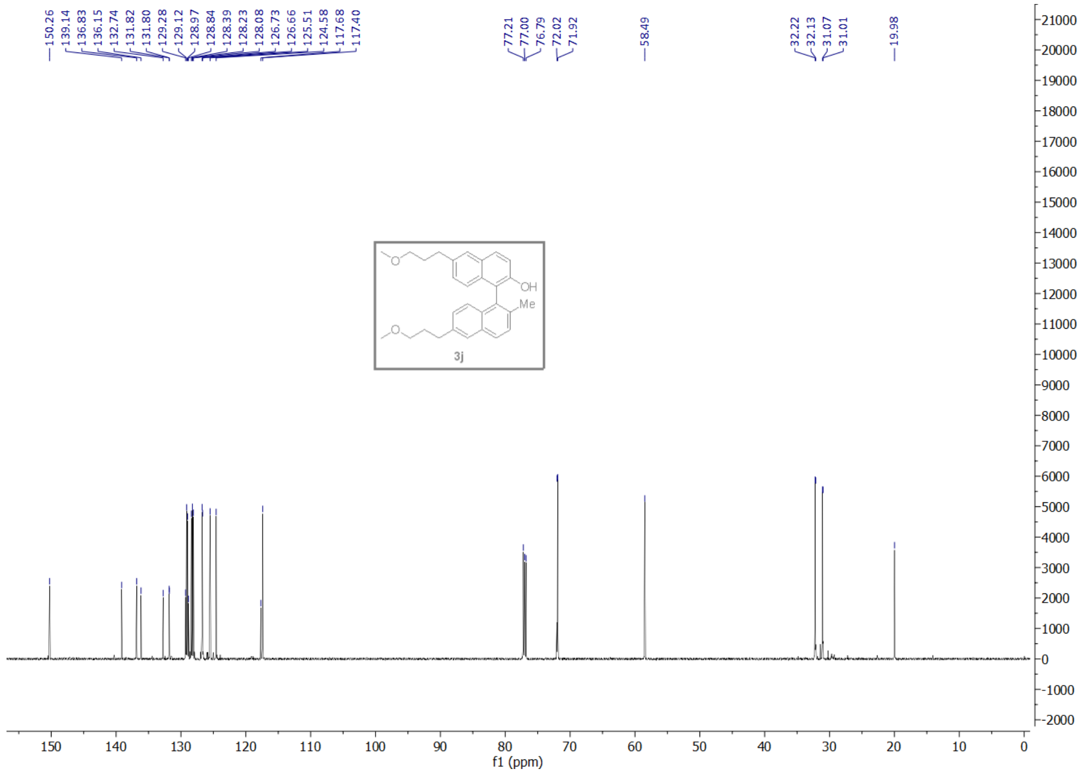

Supplementary Figure 33. ^{1}H NMR and ^{13}C NMR spectrum of **3j**.

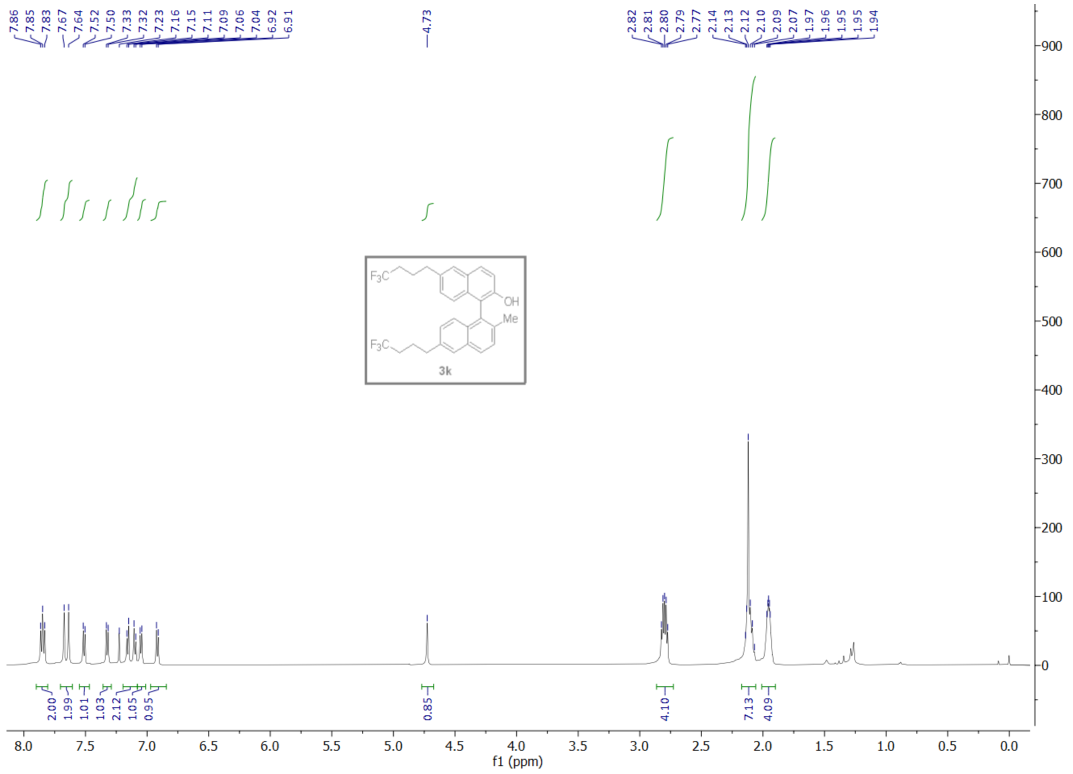

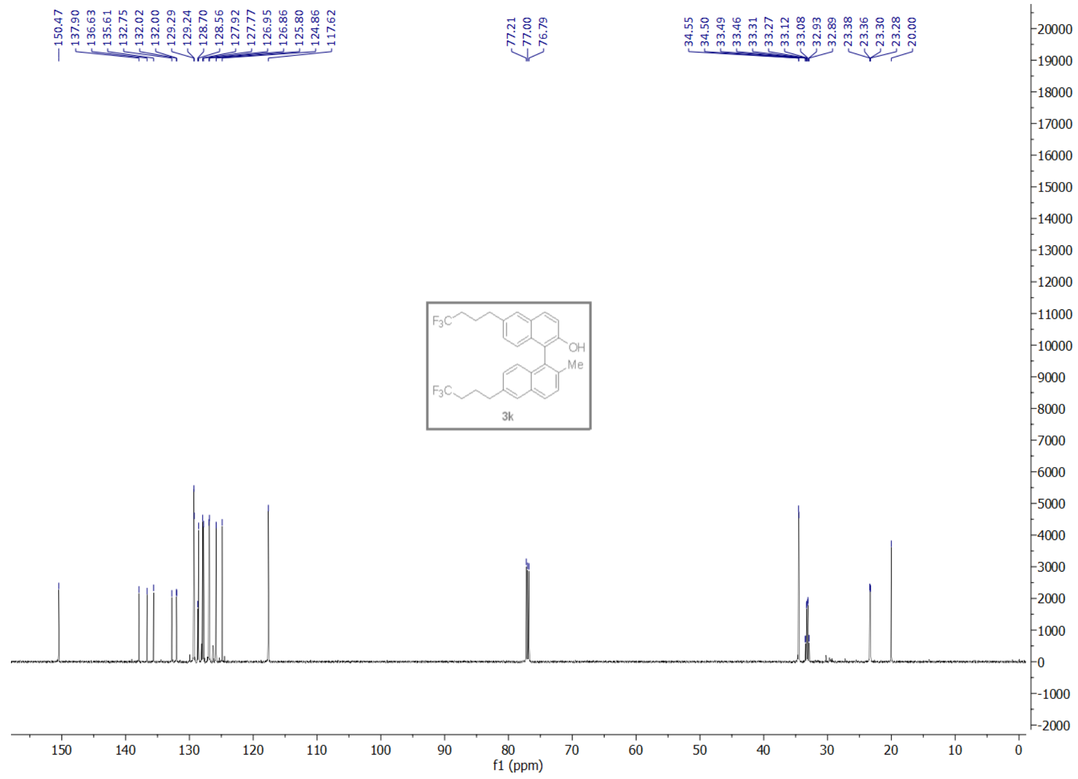

Supplementary Figure 34. ^{1}H NMR and ^{13}C NMR spectrum of **3k**.

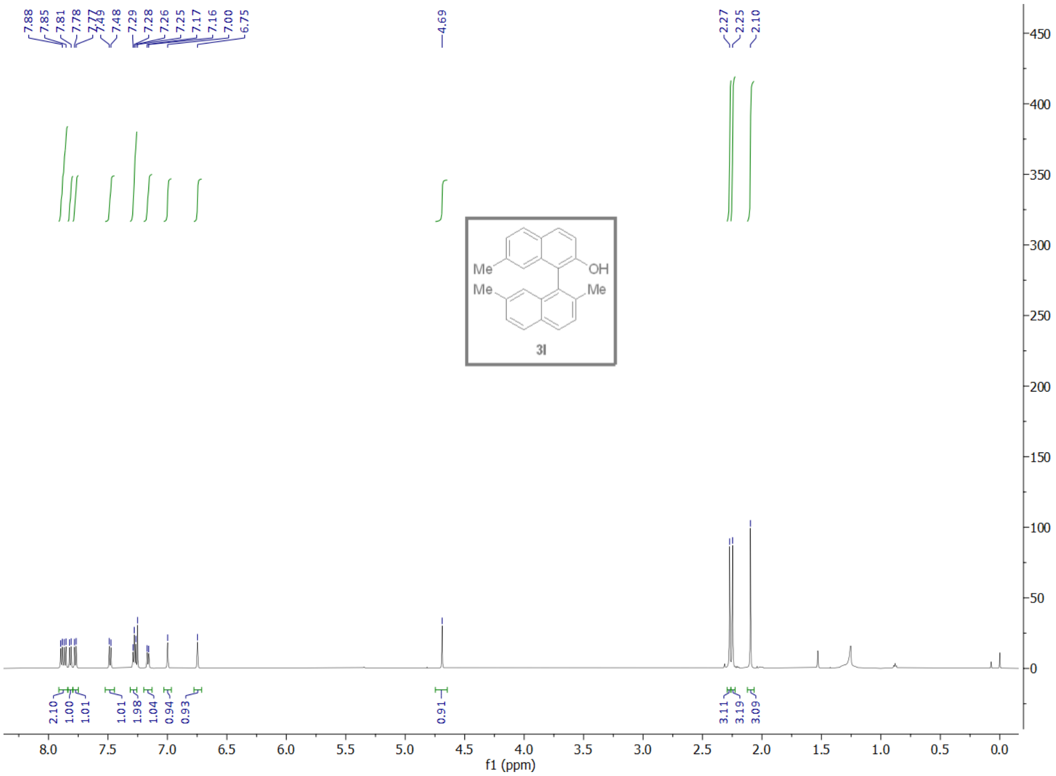

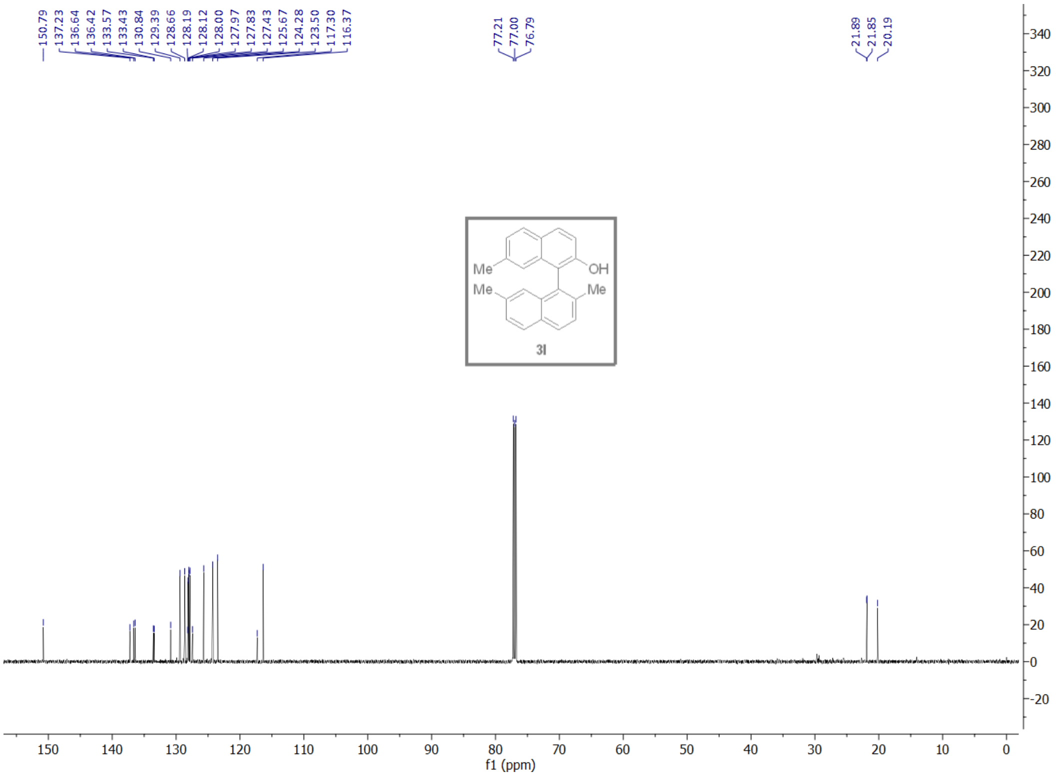

Supplementary Figure 35. ^{1}H NMR and ^{13}C NMR spectrum of **3l**.

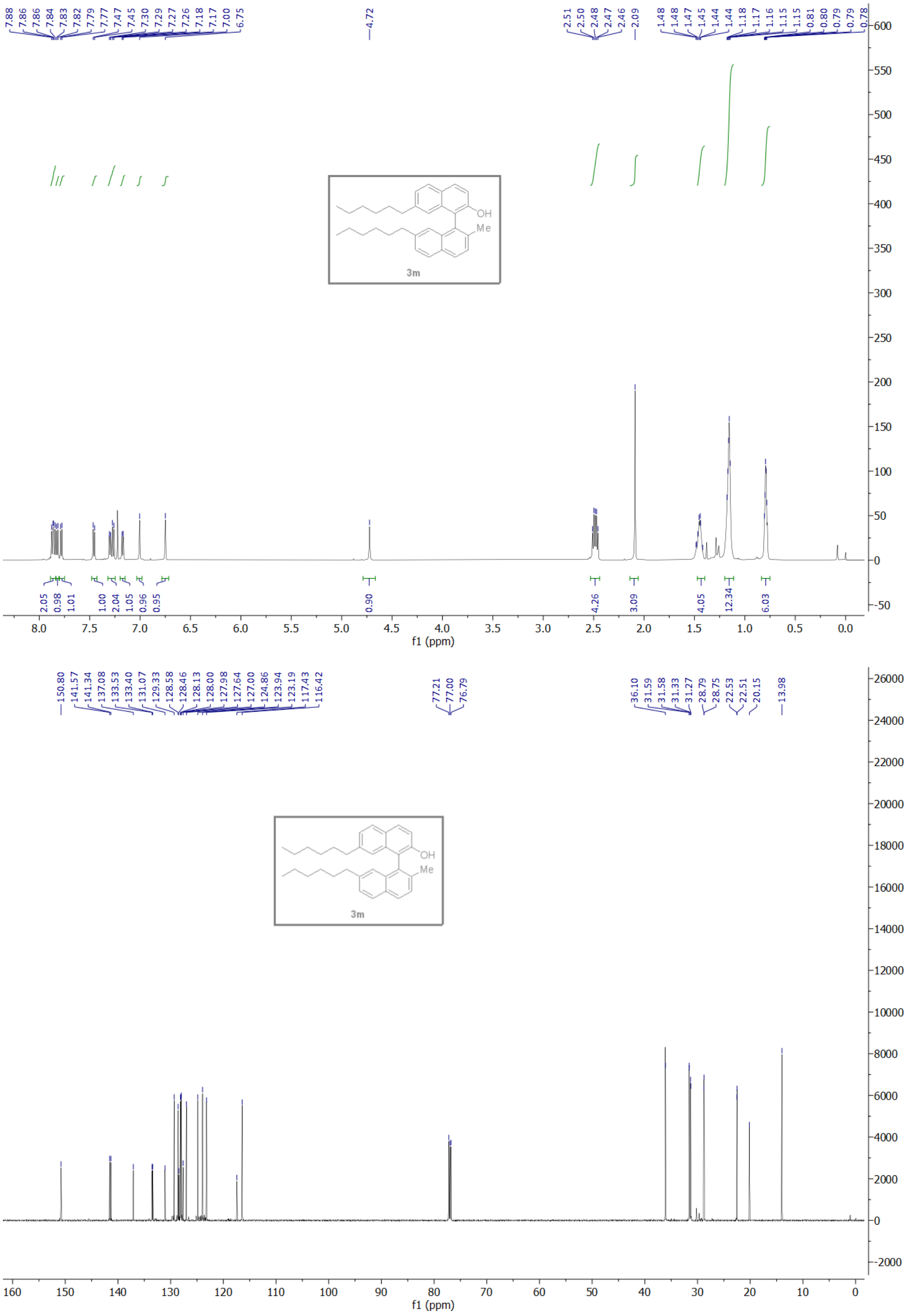

Supplementary Figure 36. ^{1}H NMR and ^{13}C NMR spectrum of **3m**.

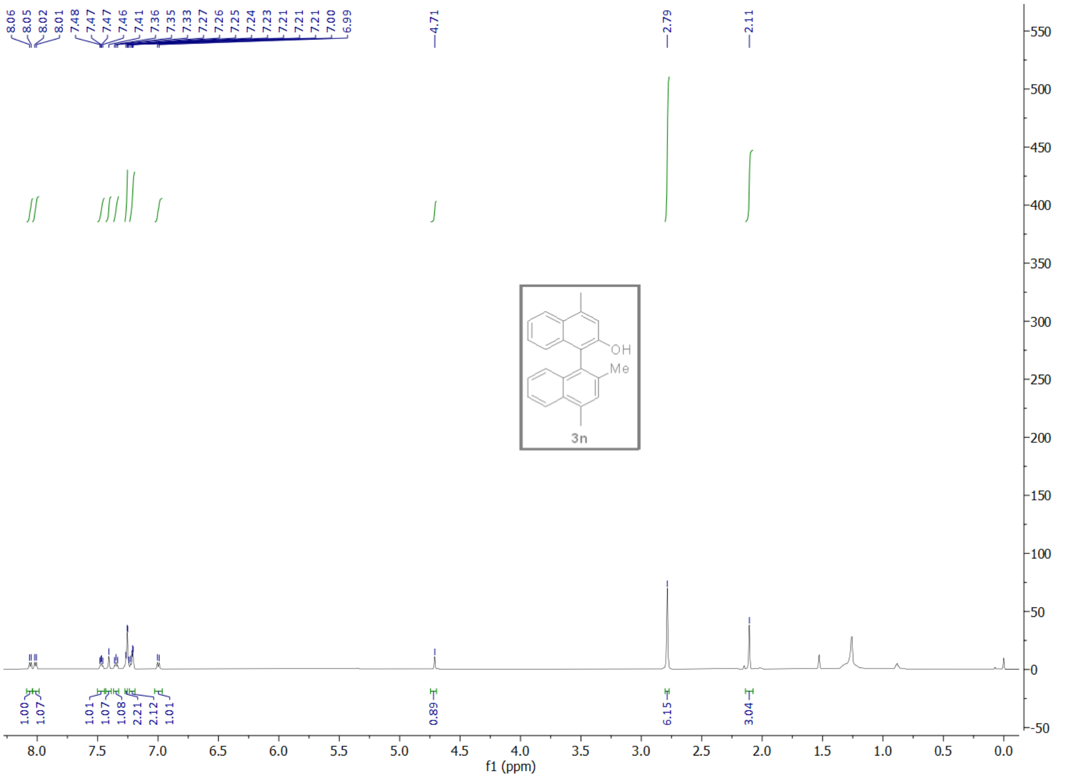

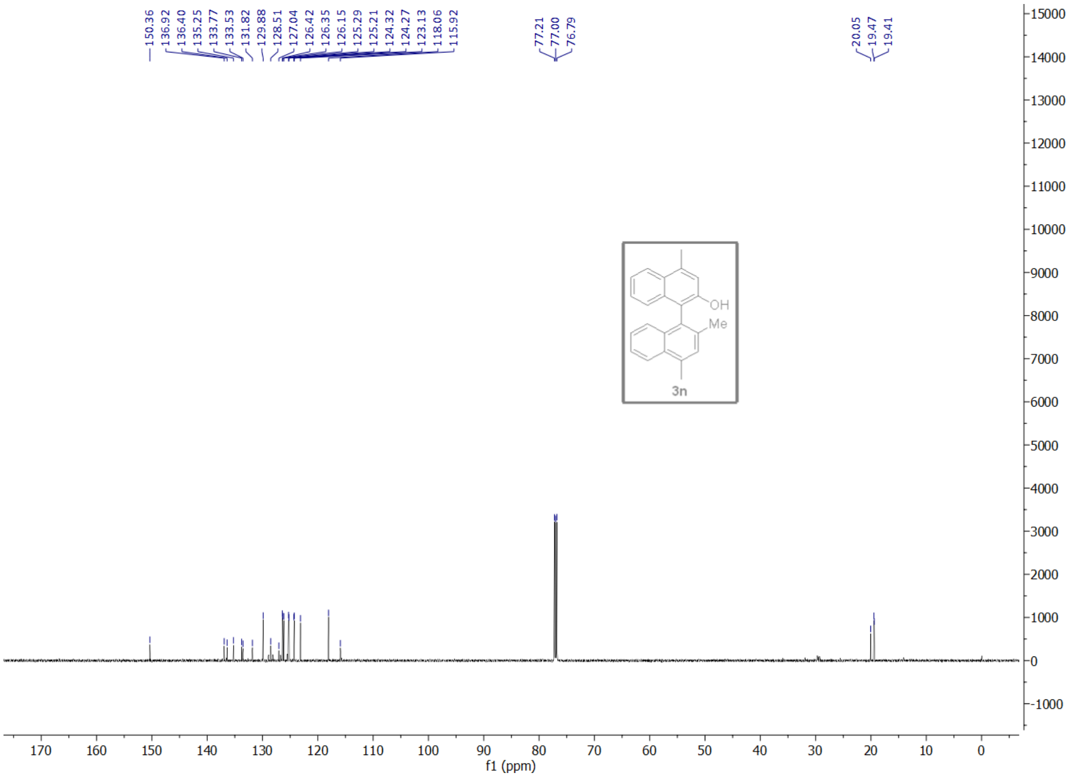

Supplementary Figure 37. ^{1}H NMR and ^{13}C NMR spectrum of **3n**.

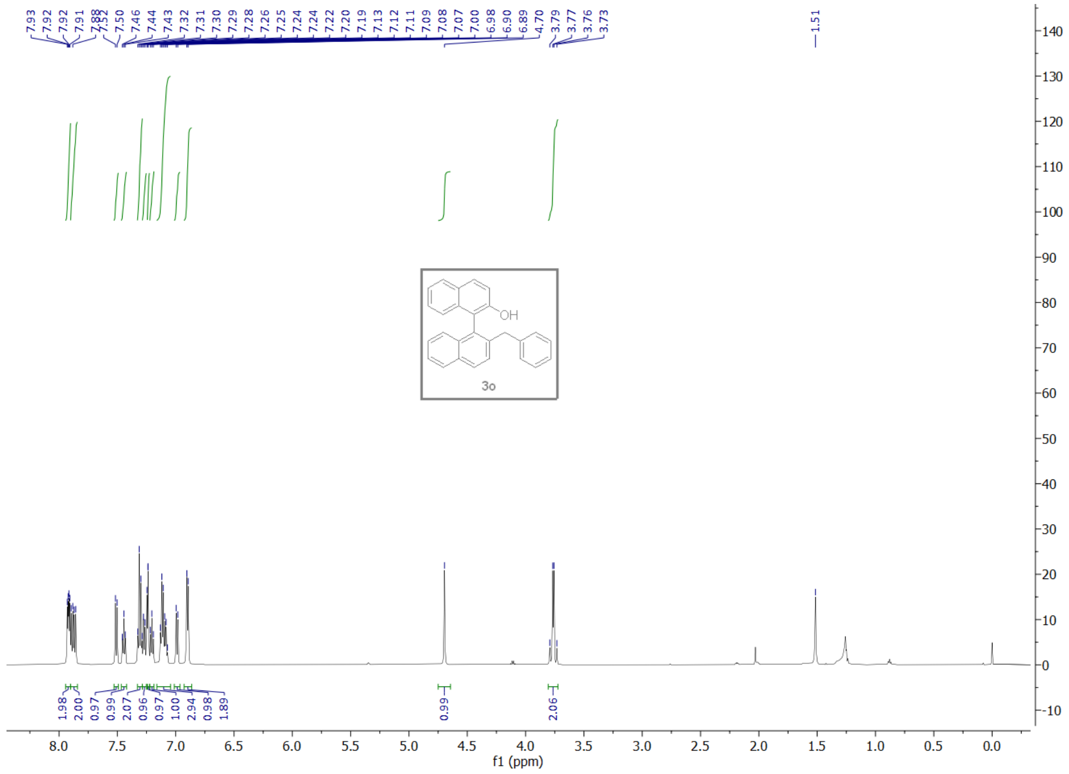

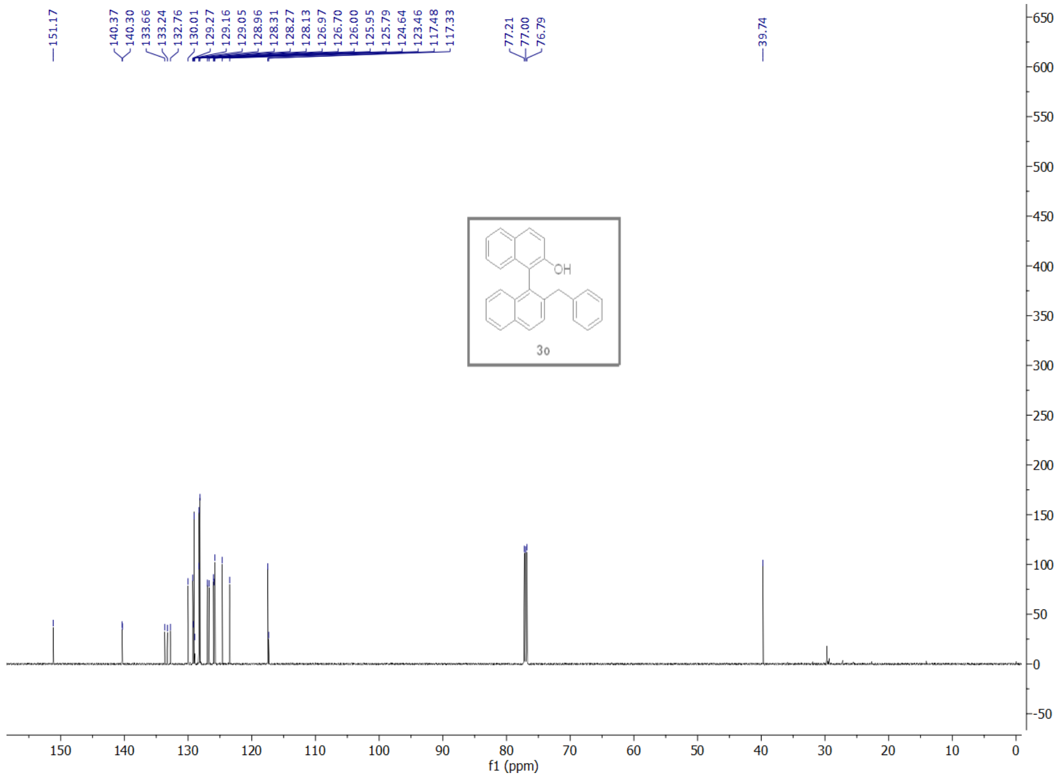

Supplementary Figure 38. ^{1}H NMR and ^{13}C NMR spectrum of **3o**.

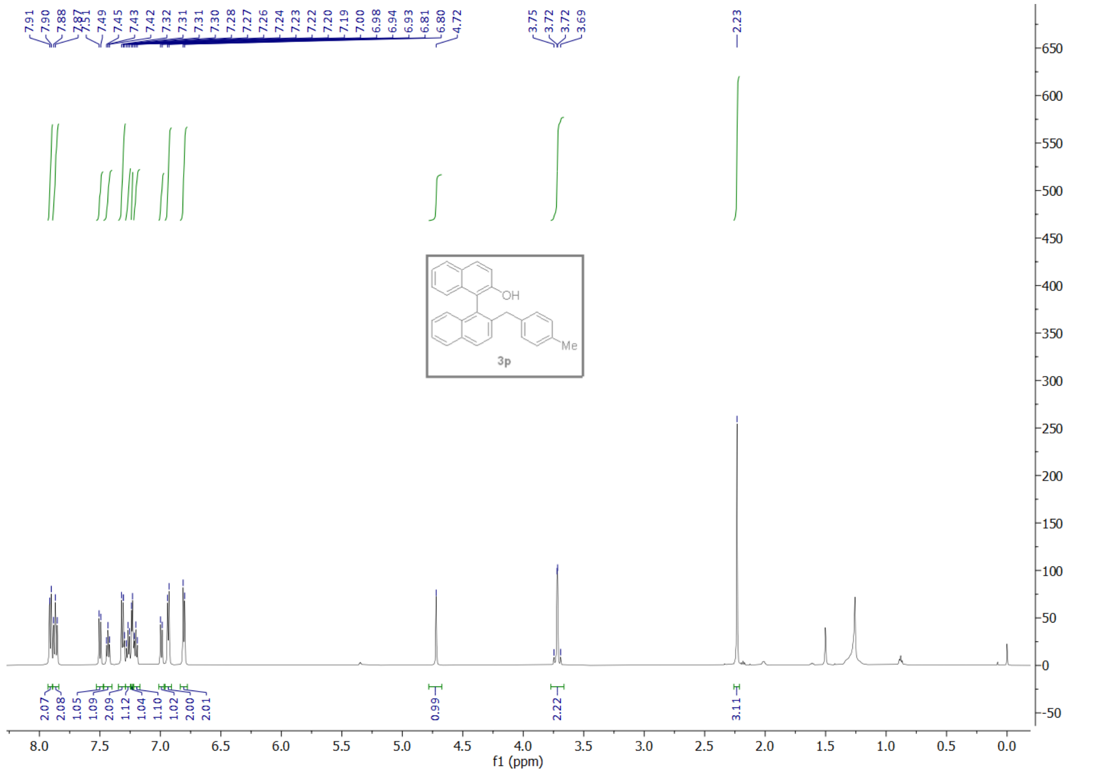

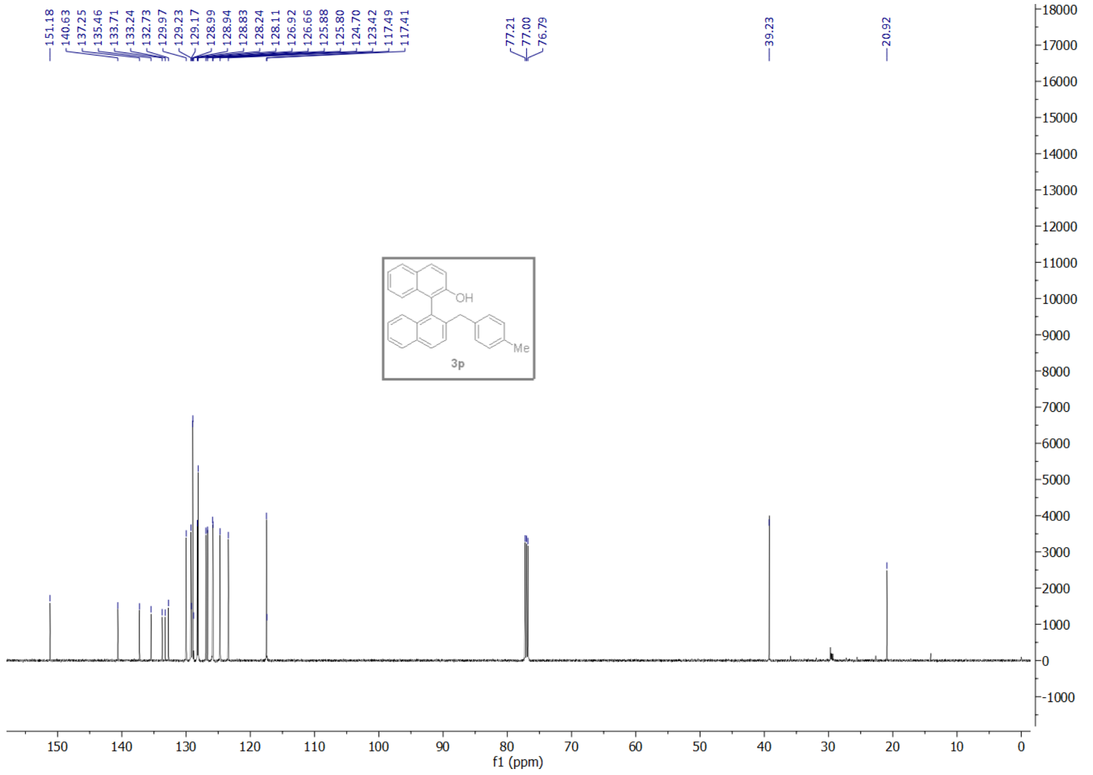

Supplementary Figure 39. ^{1}H NMR and ^{13}C NMR spectrum of **3p**.

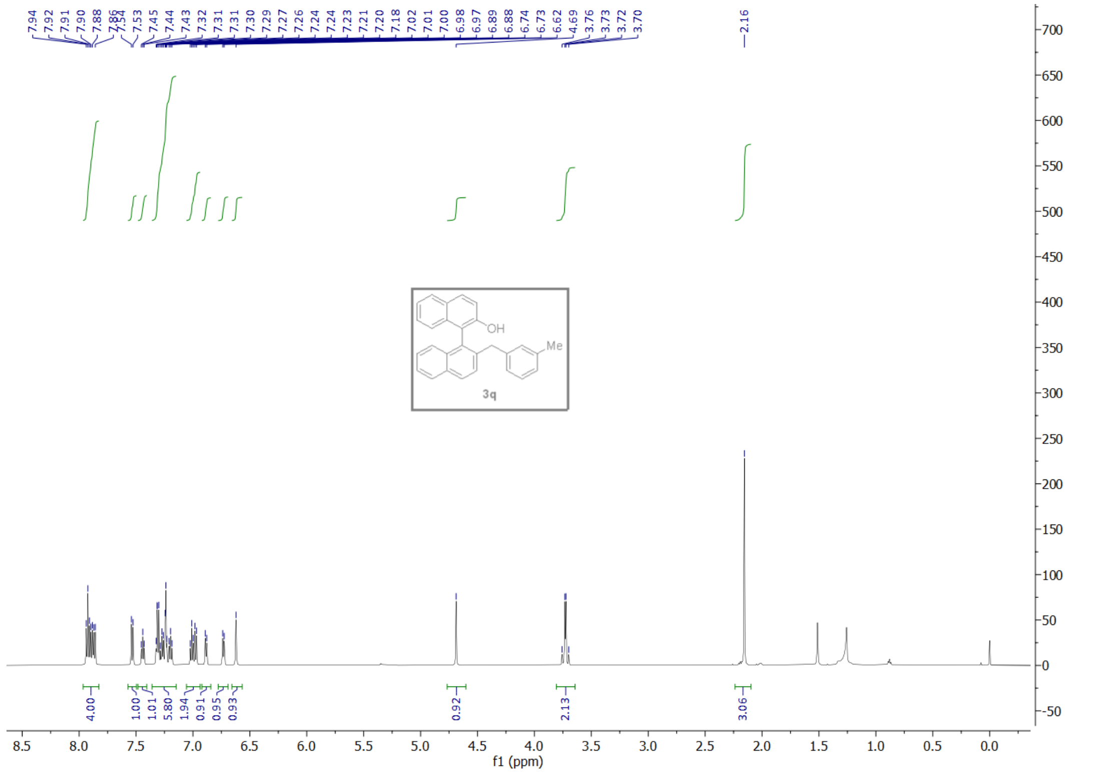

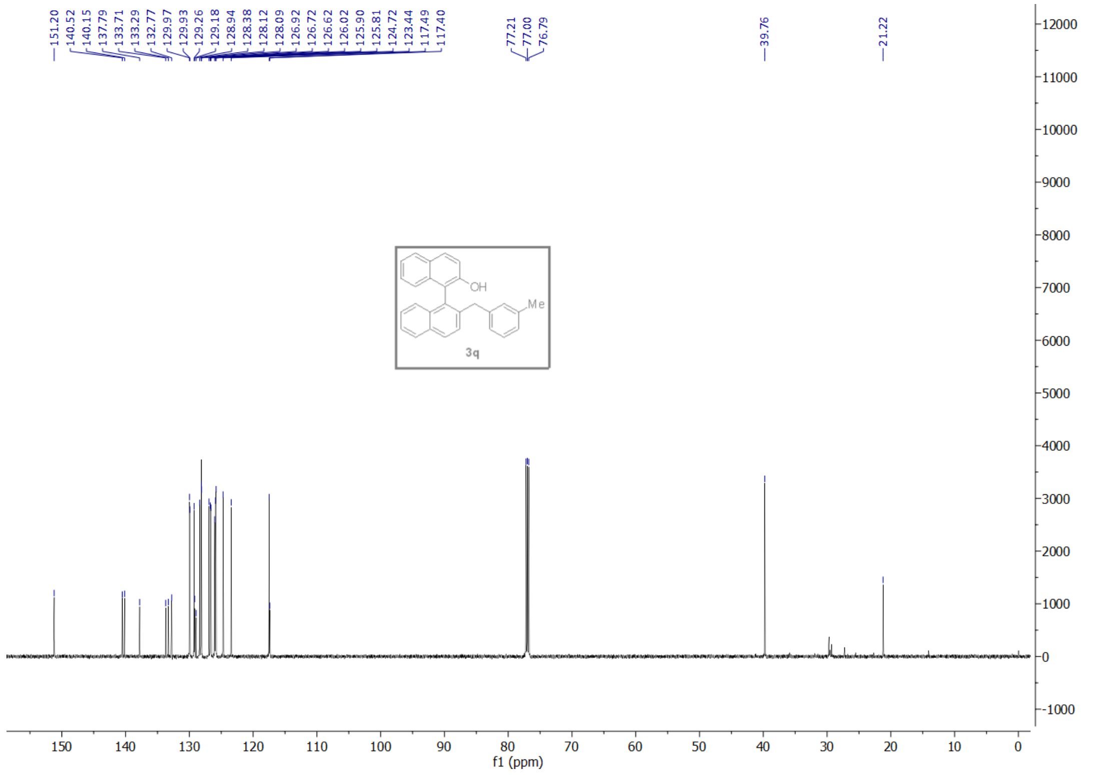

Supplementary Figure 40. ^{1}H NMR and ^{13}C NMR spectrum of **3q**.

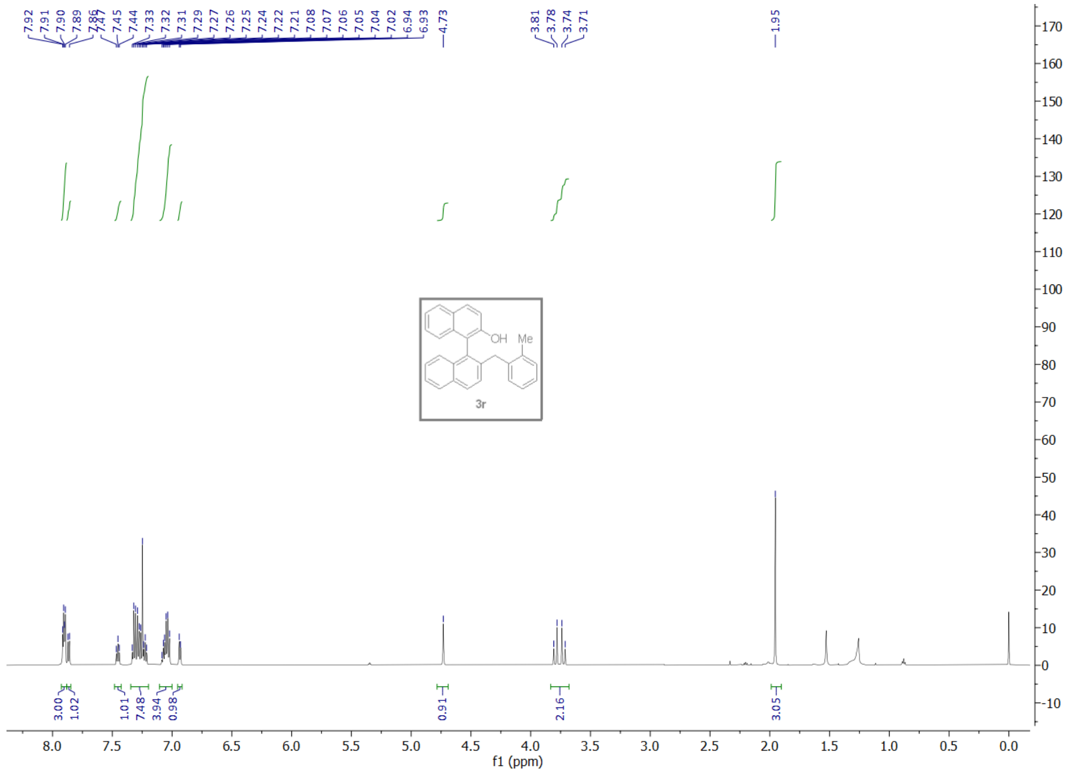

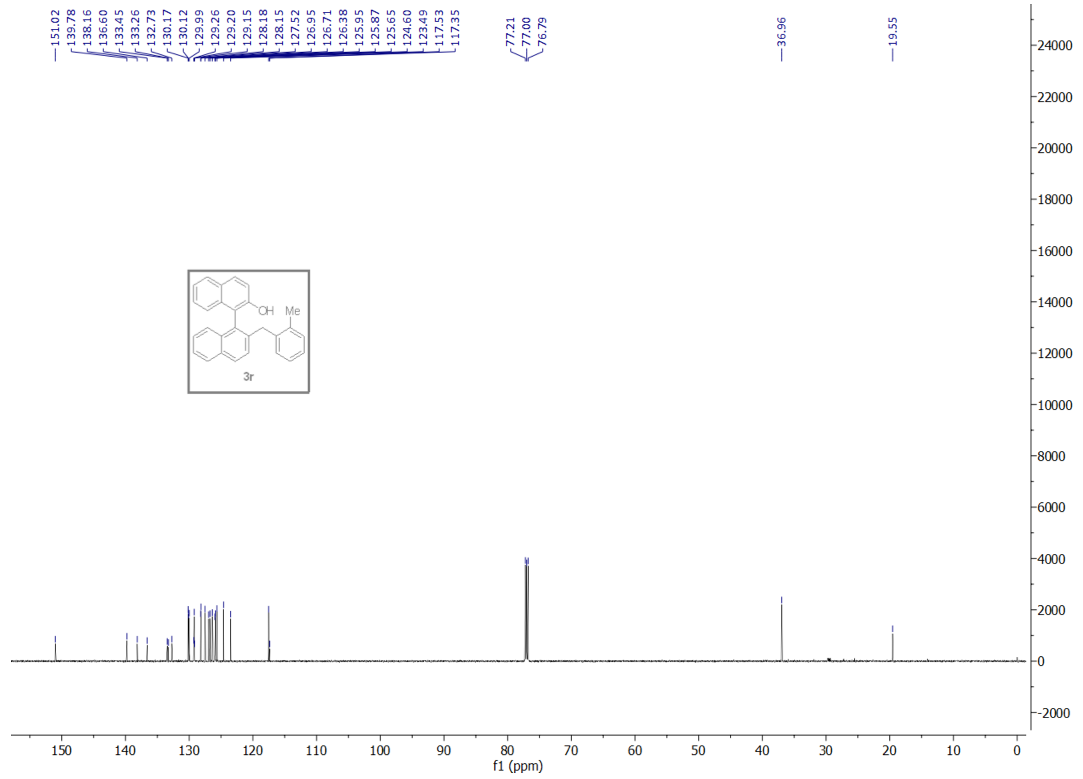

Supplementary Figure 41. ^{1}H NMR and ^{13}C NMR spectrum of **3r**.

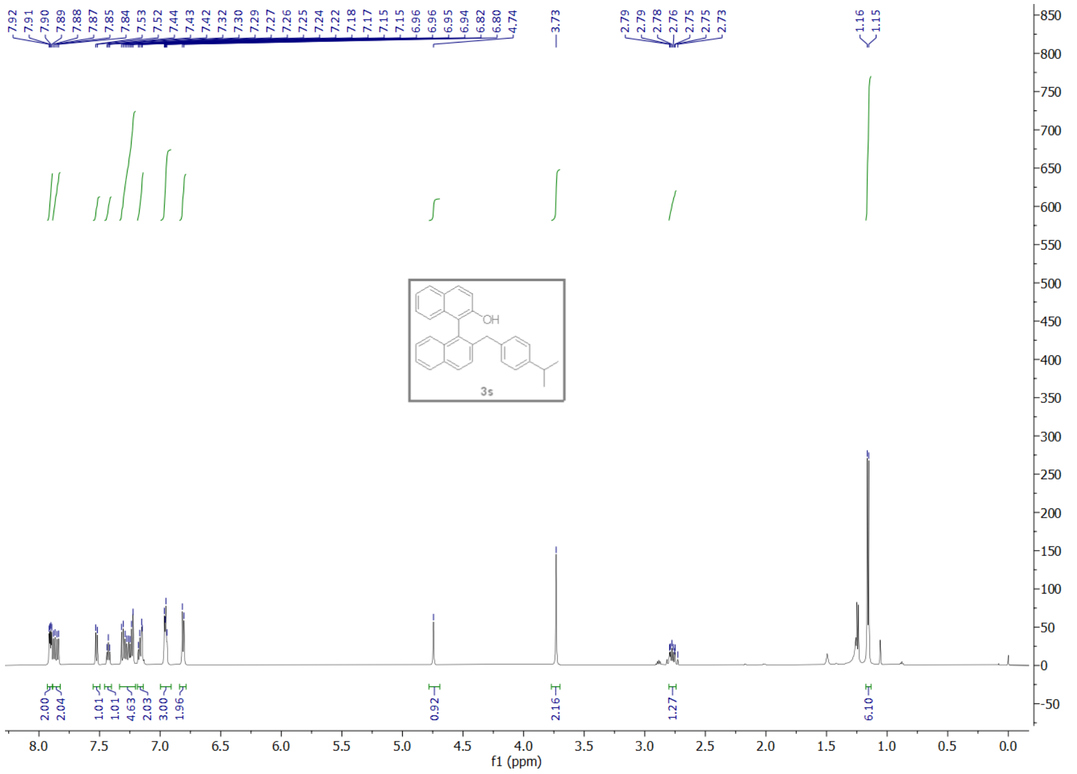

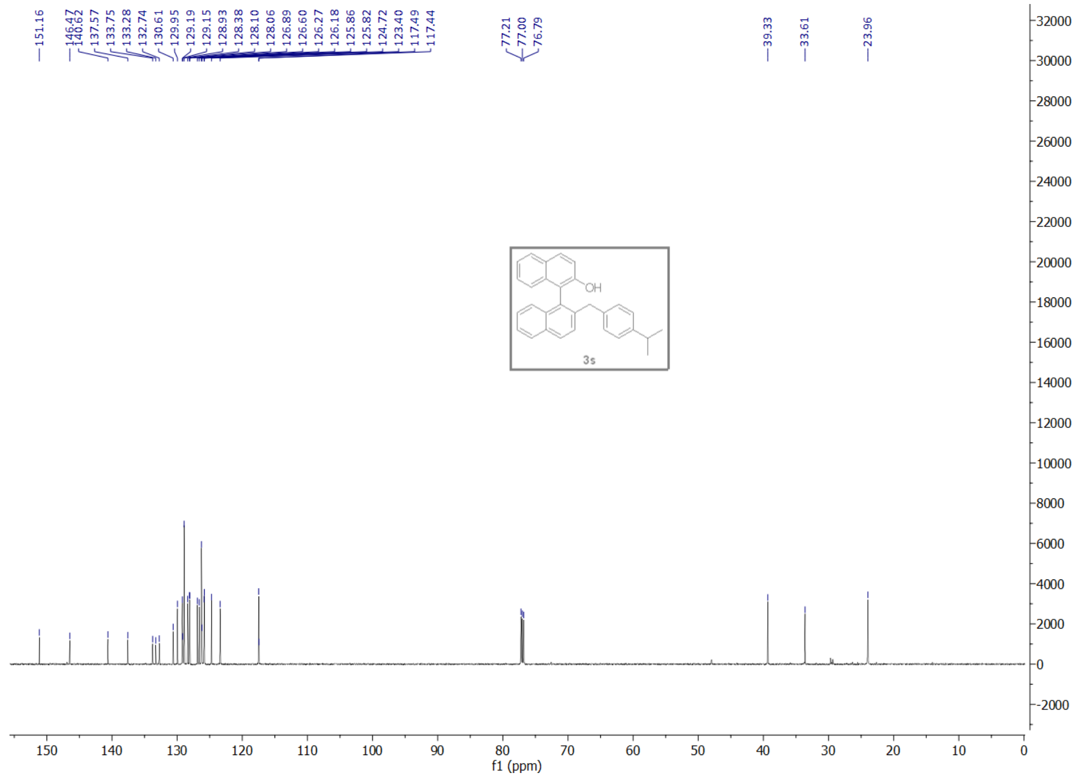

Supplementary Figure 42. ^{1}H NMR and ^{13}C NMR spectrum of **3s**.

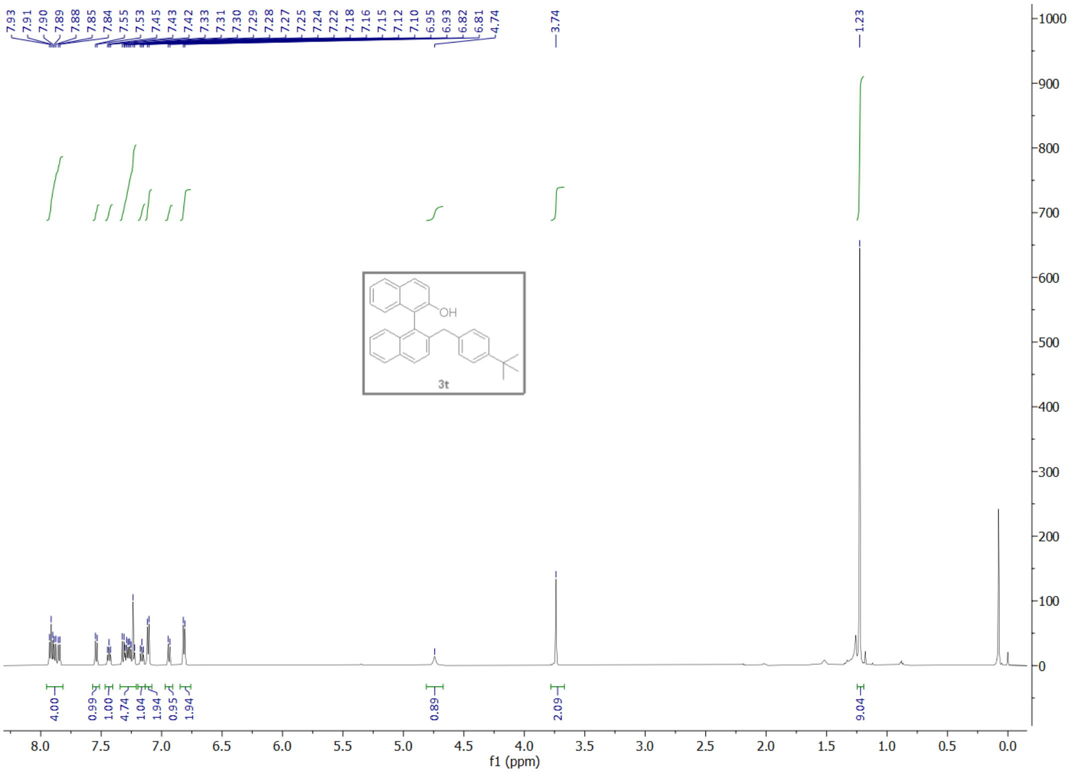

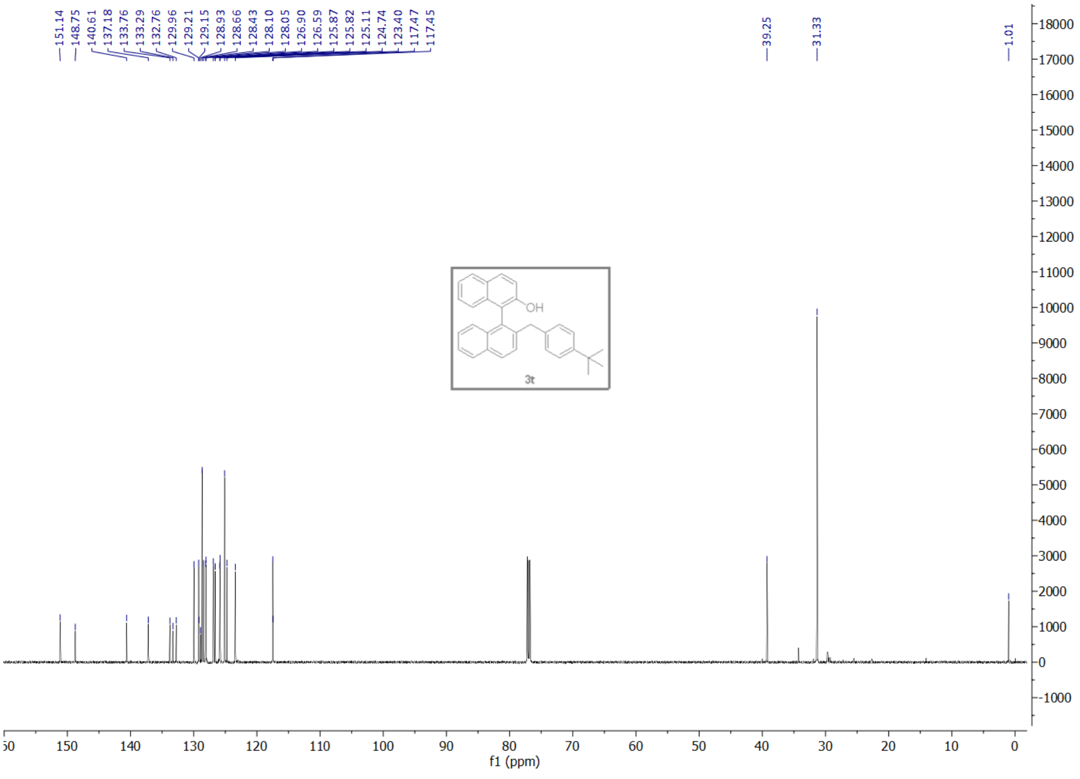

Supplementary Figure 43. ^{1}H NMR and ^{13}C NMR spectrum of **3t**.

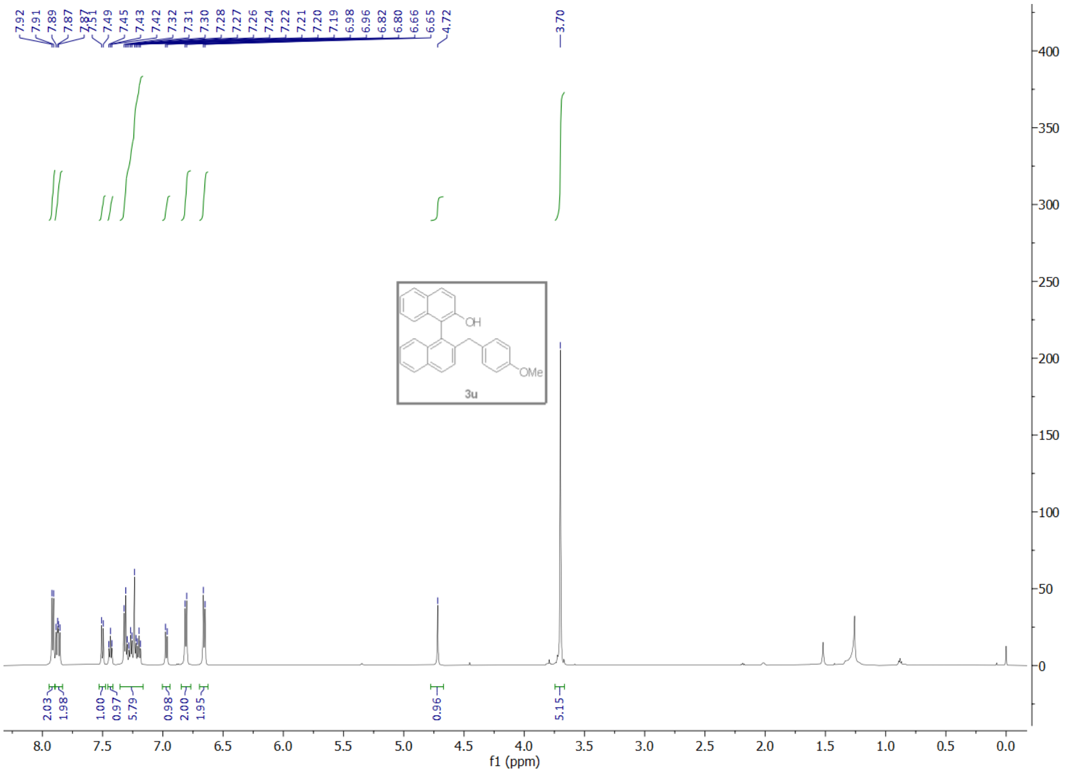

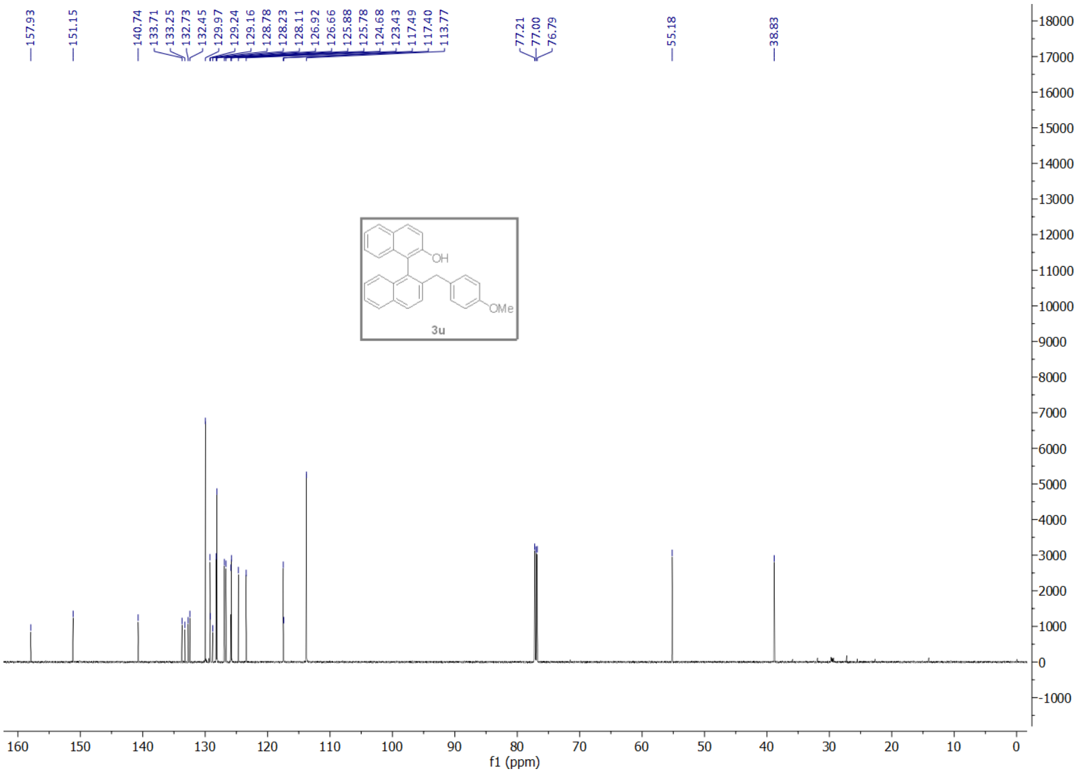

Supplementary Figure 44. ^{1}H NMR and ^{13}C NMR spectrum of **3u**.

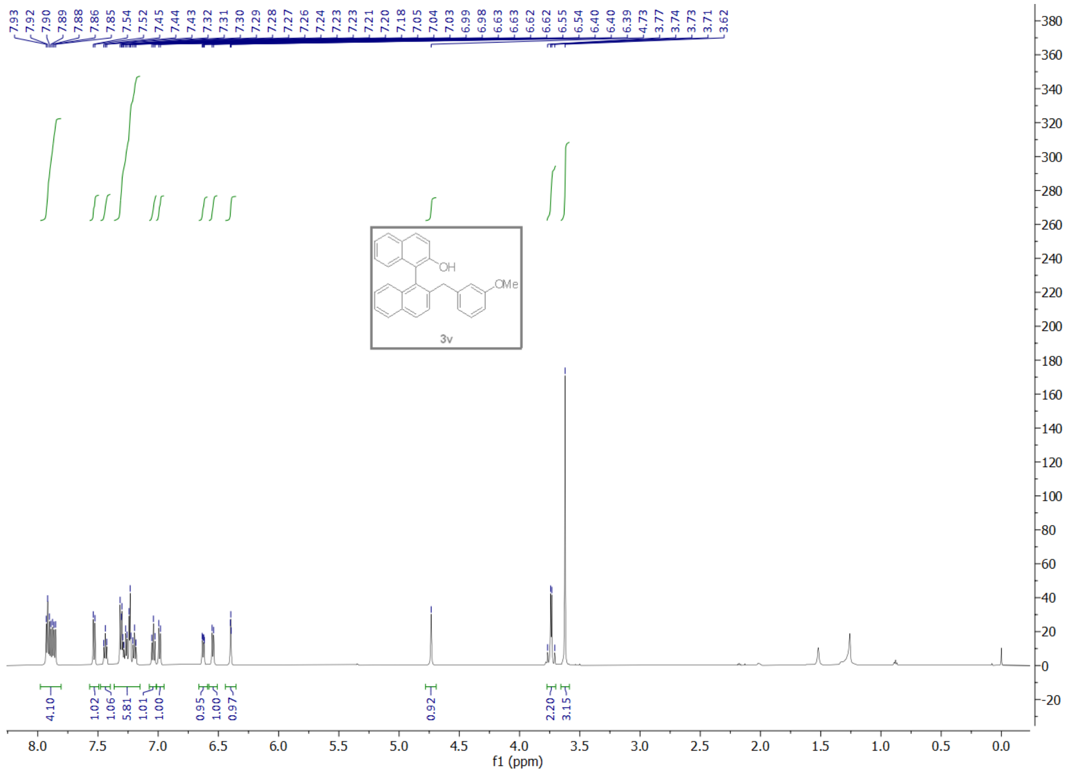

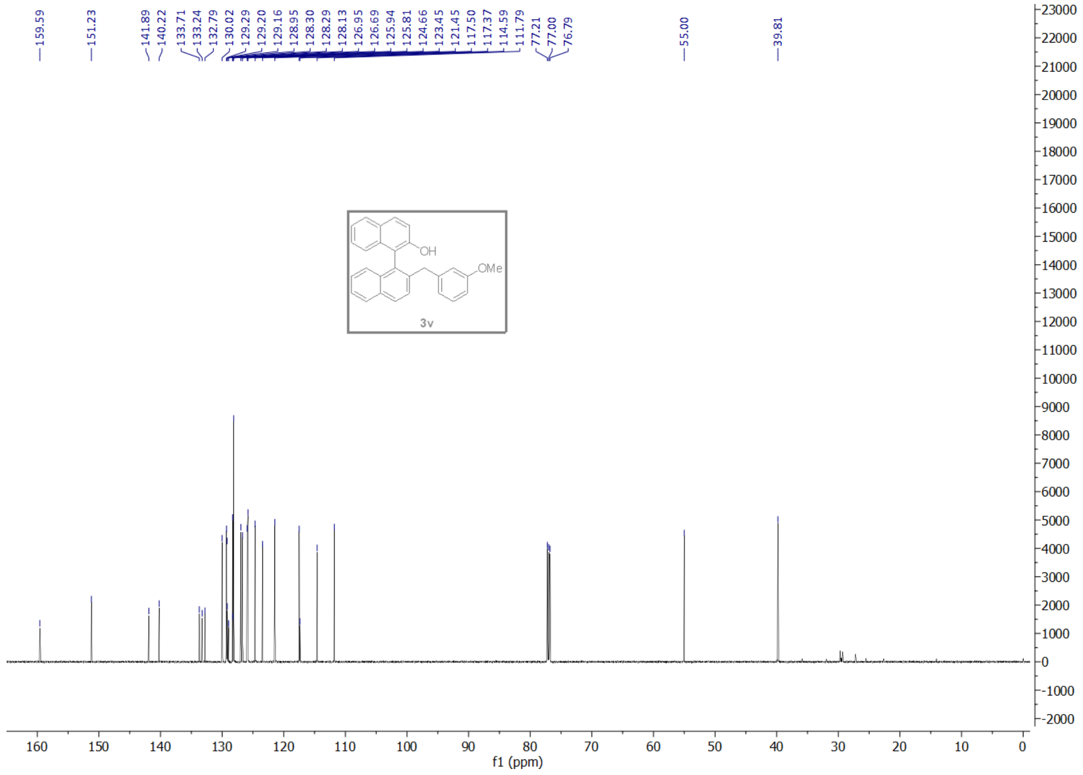

Supplementary Figure 45. ^{1}H NMR and ^{13}C NMR spectrum of **3v**.

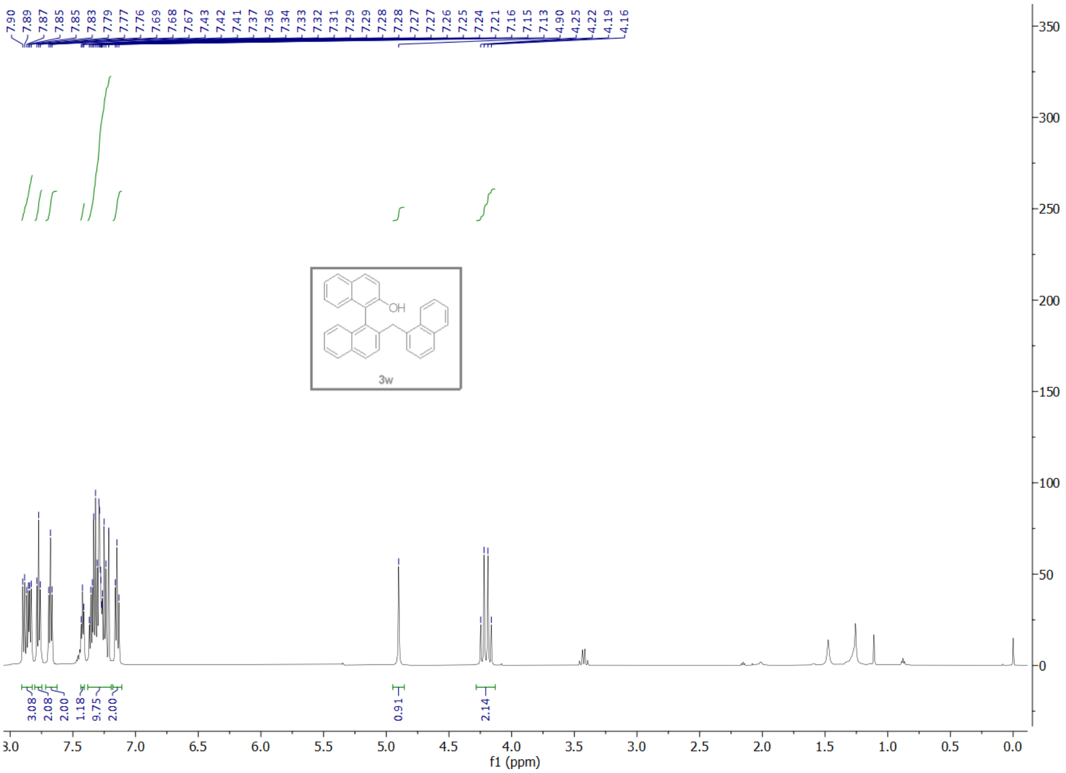

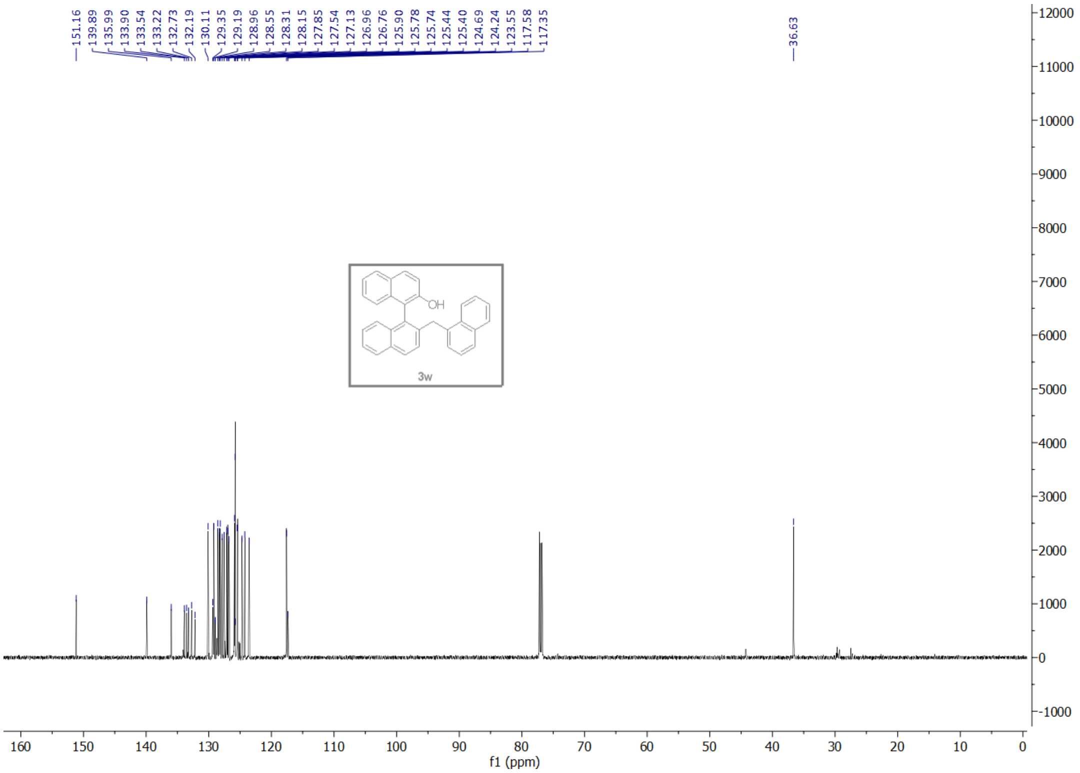

Supplementary Figure 46. ^{1}H NMR and ^{13}C NMR spectrum of **3w**.

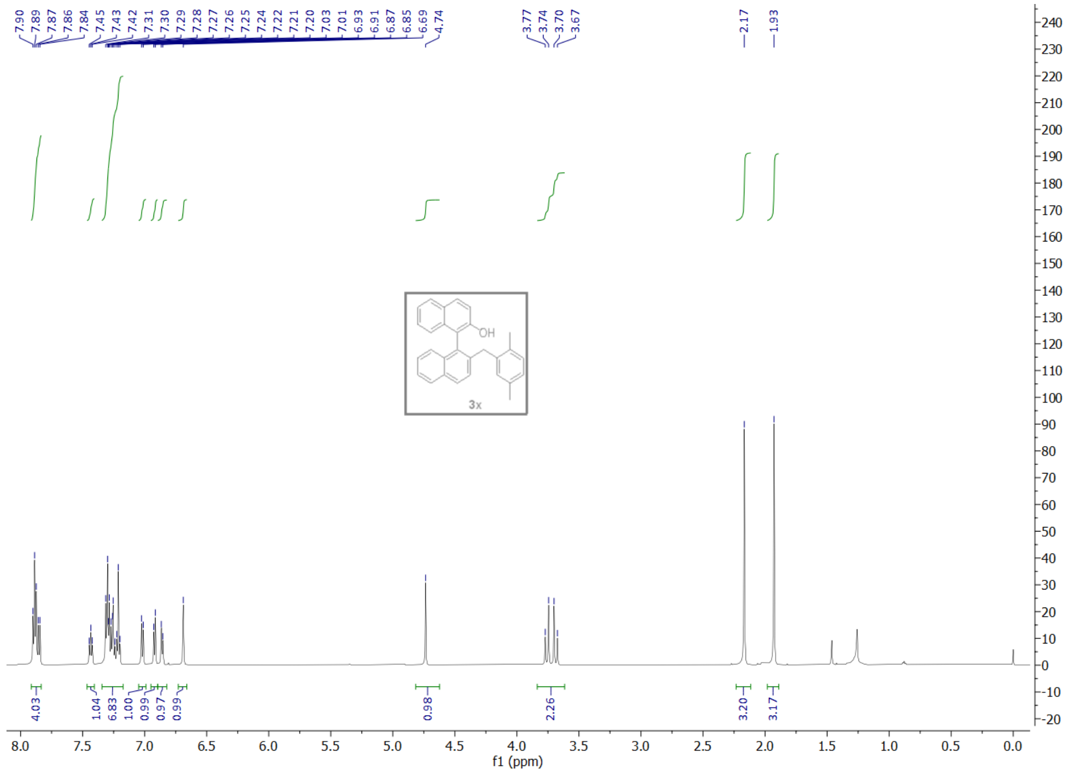

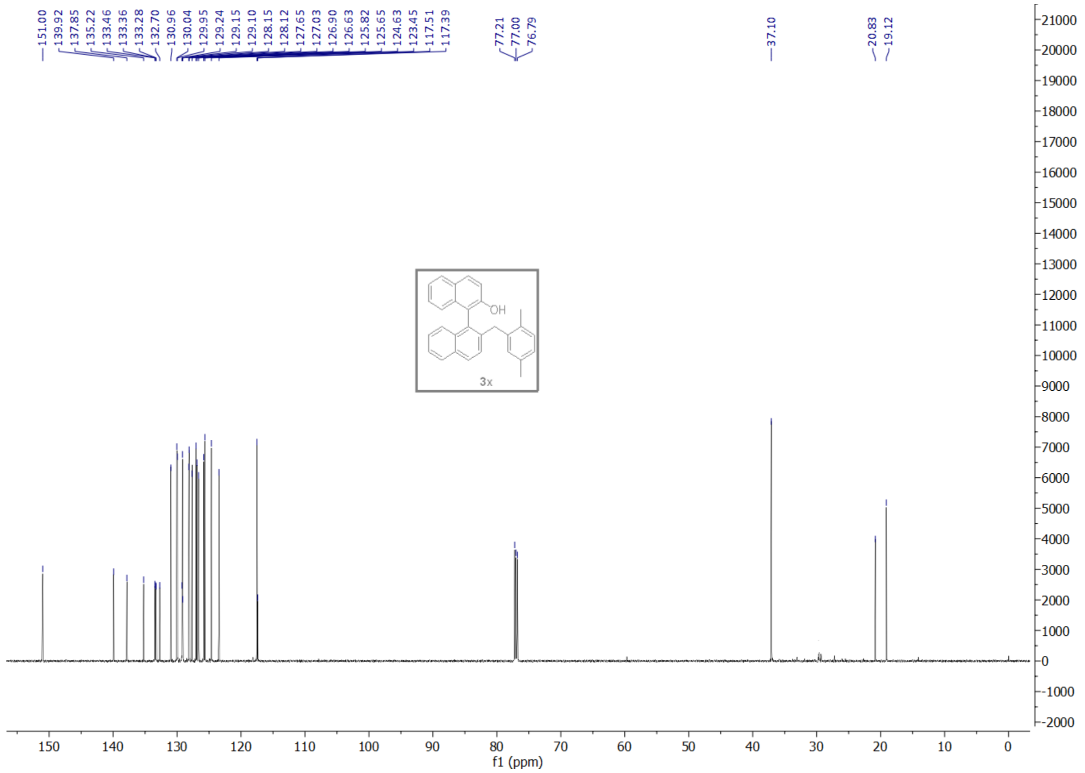

Supplementary Figure 47. ^{1}H NMR and ^{13}C NMR spectrum of **3x**.

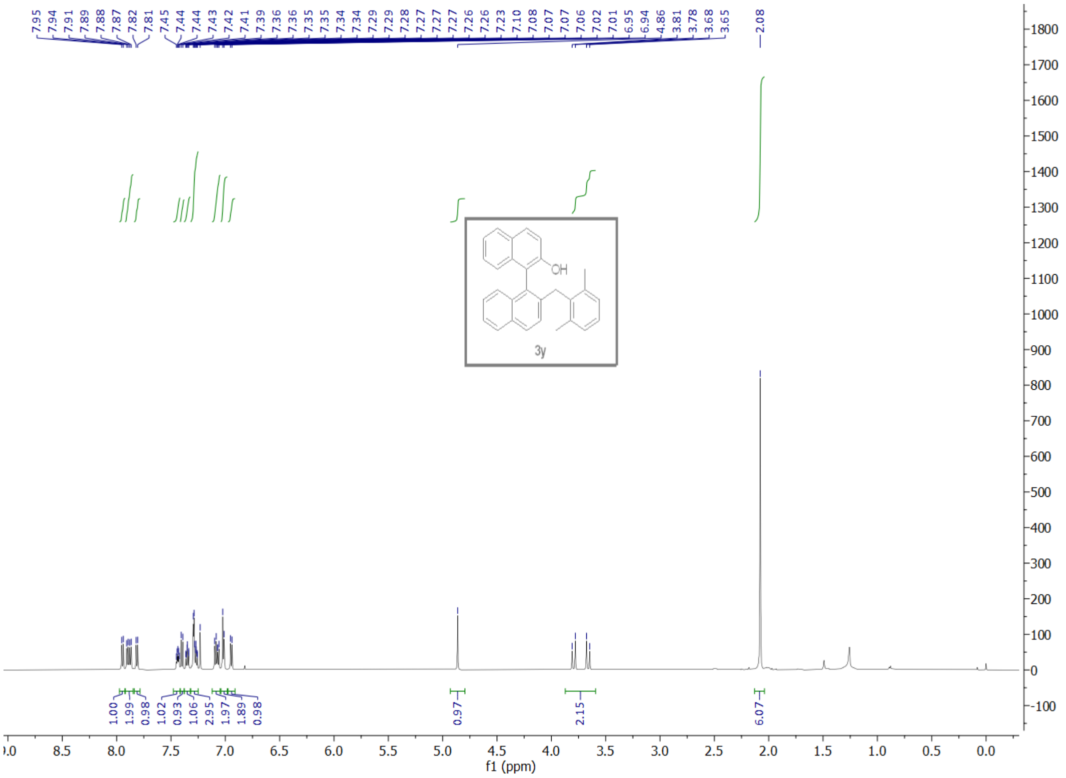

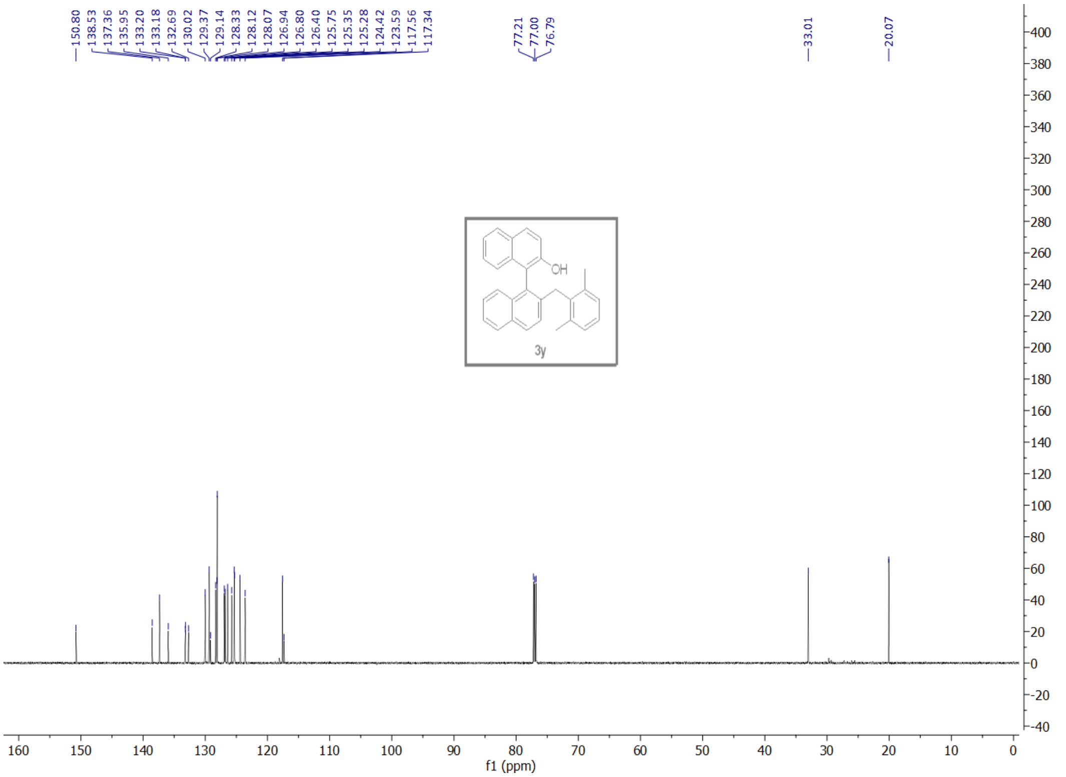

Supplementary Figure 48. ^{1}H NMR and ^{13}C NMR spectrum of **3y**.

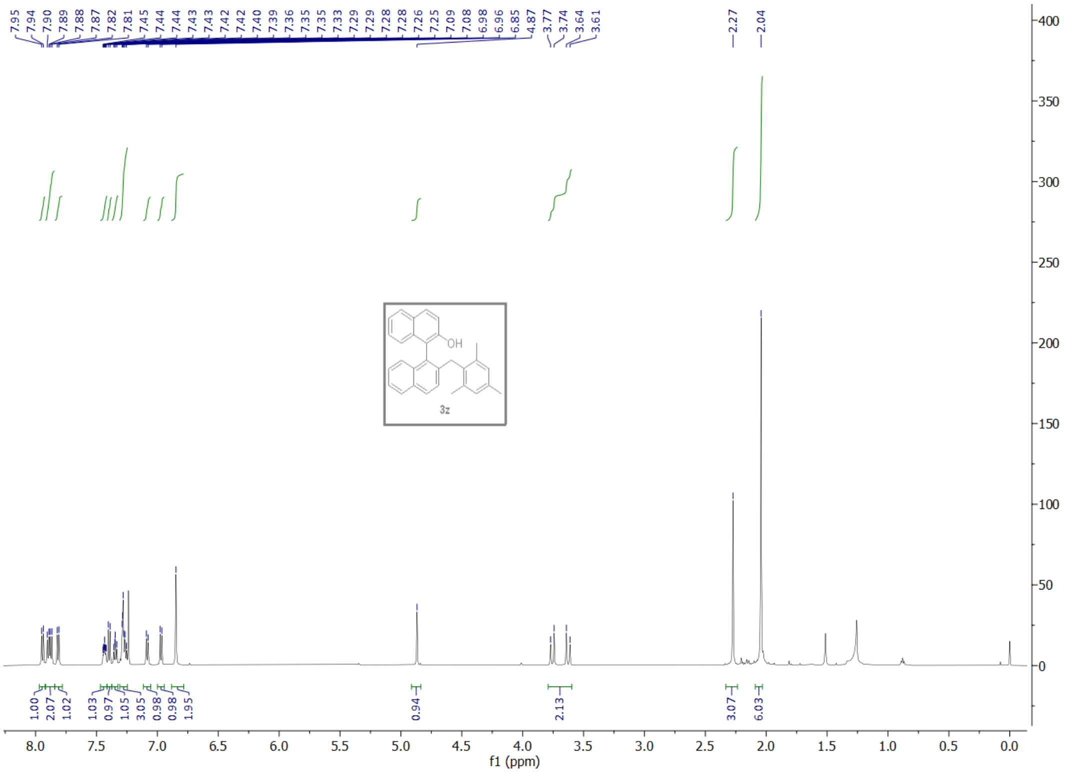

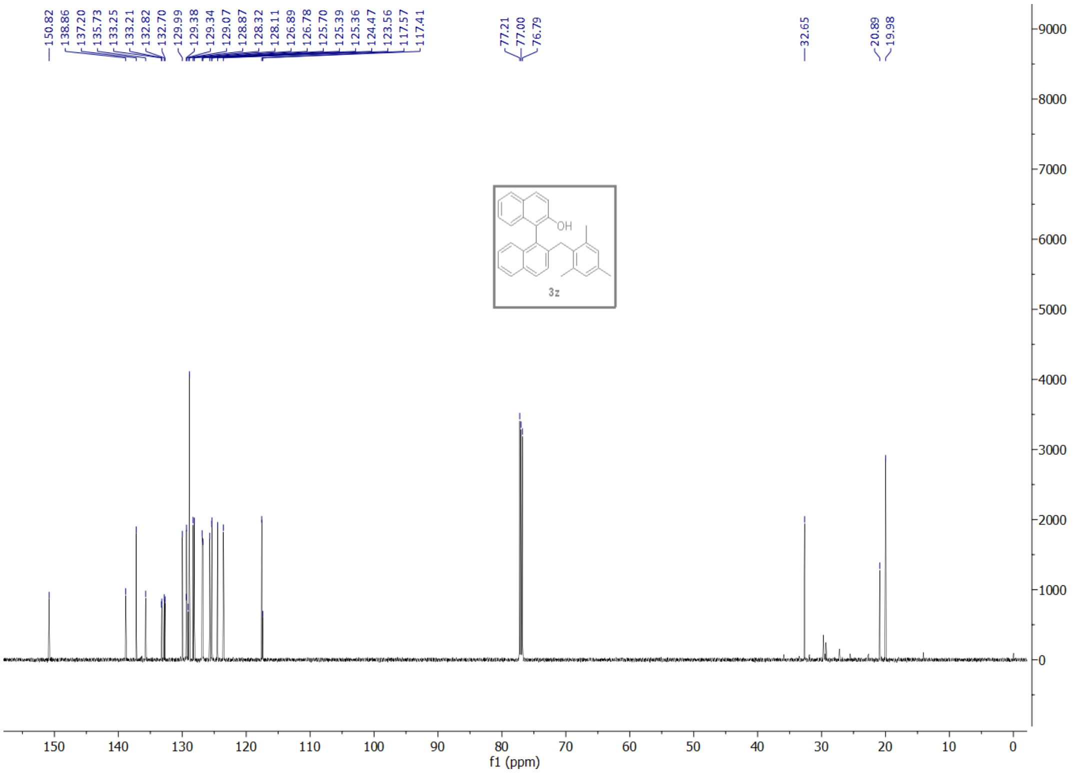

Supplementary Figure 49. ¹H NMR and ¹³C NMR spectrum of **3z**.

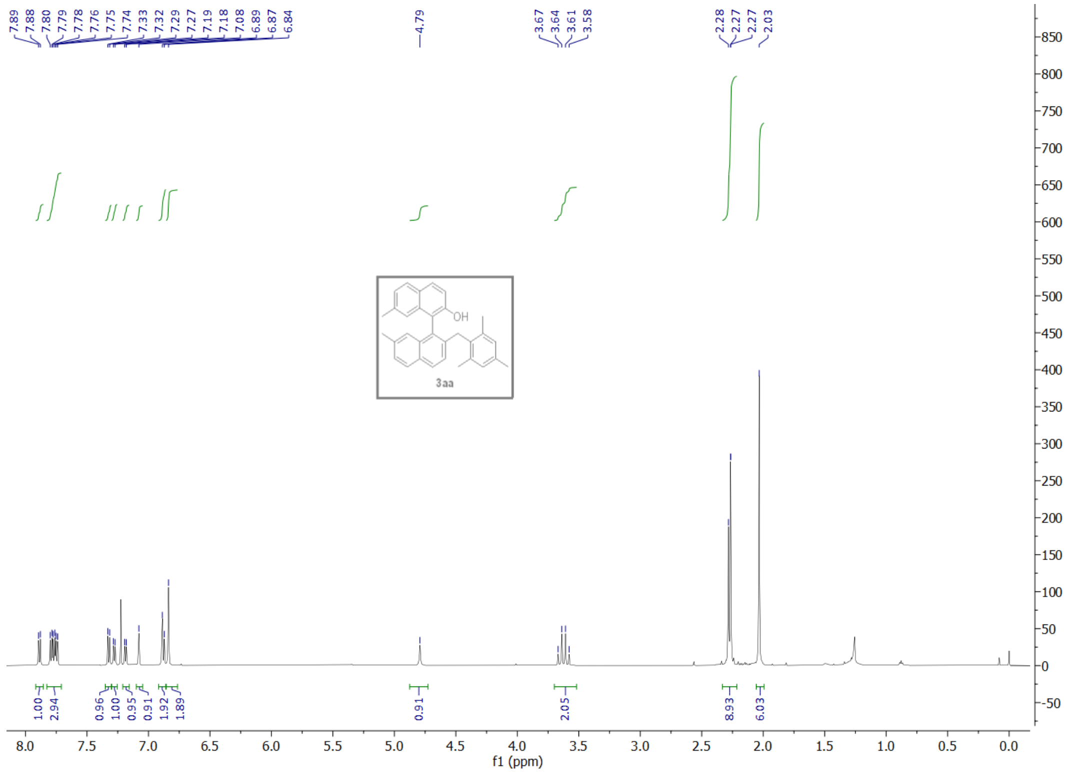

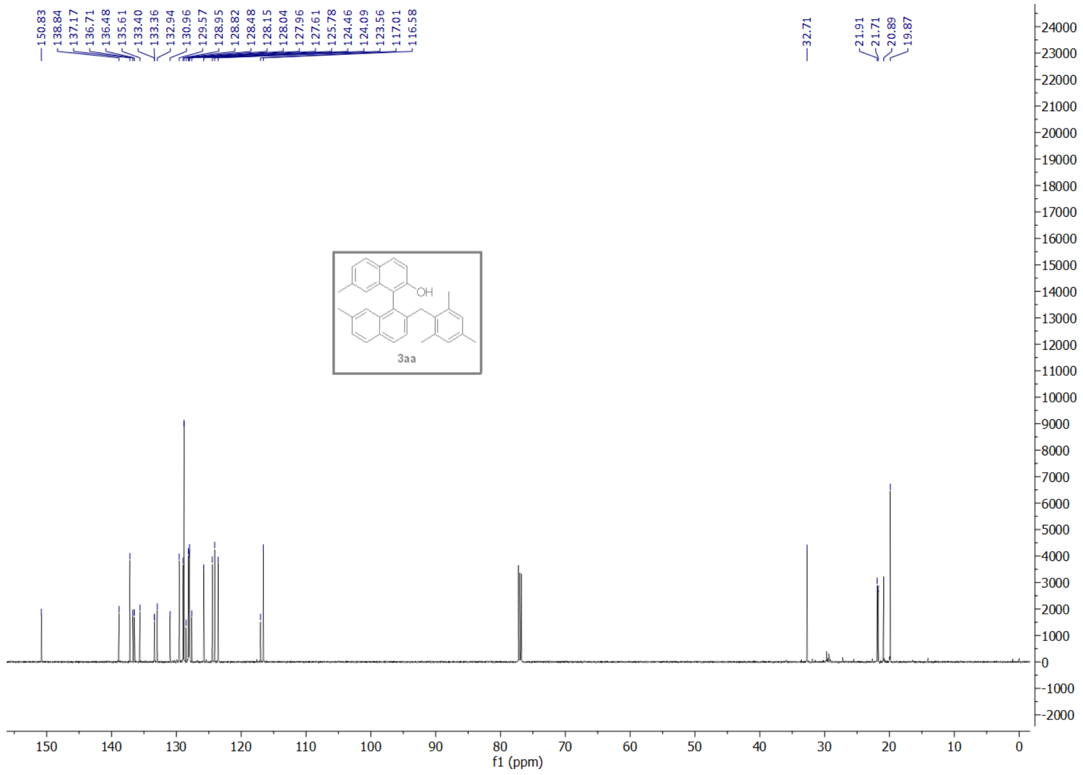

Supplementary Figure 50. ^{1}H NMR and ^{13}C NMR spectrum of **3aa**.

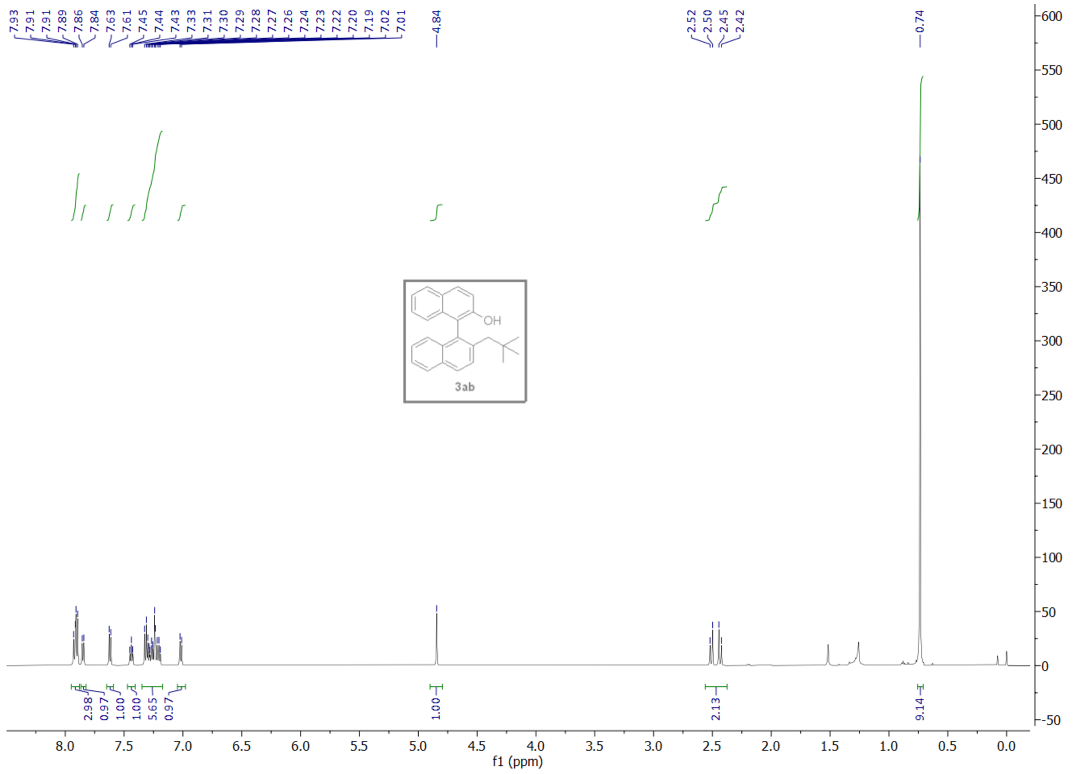

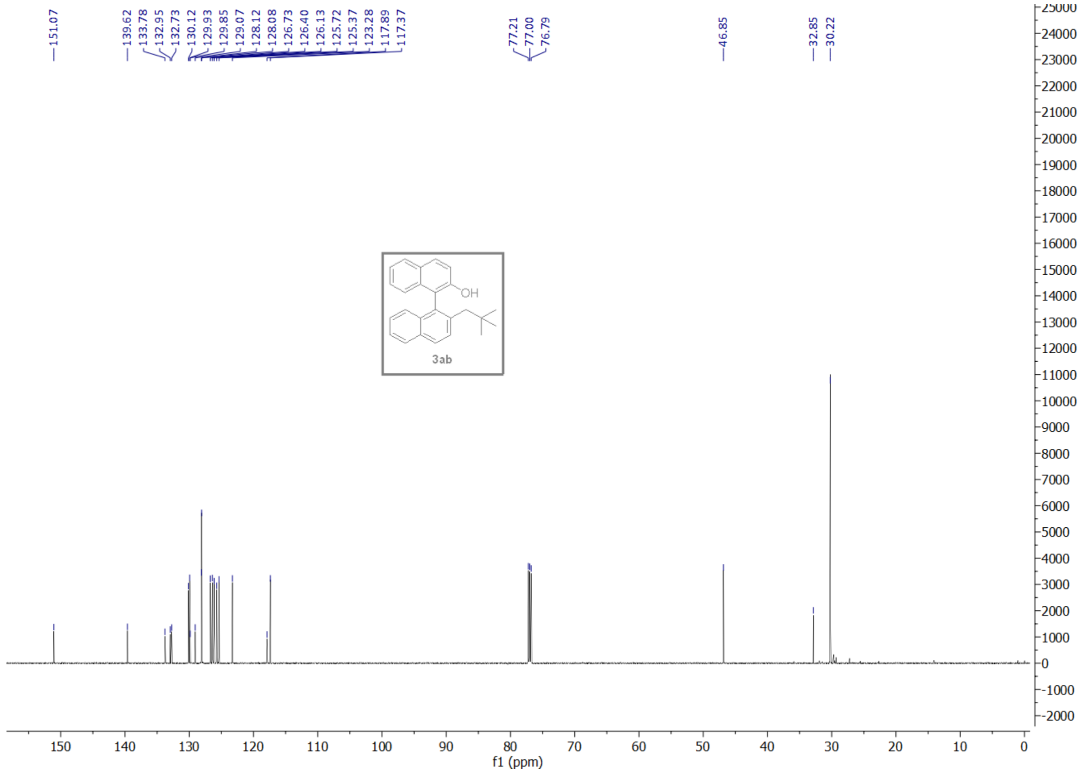

Supplementary Figure 51. ^{1}H NMR and ^{13}C NMR spectrum of **3ab**.

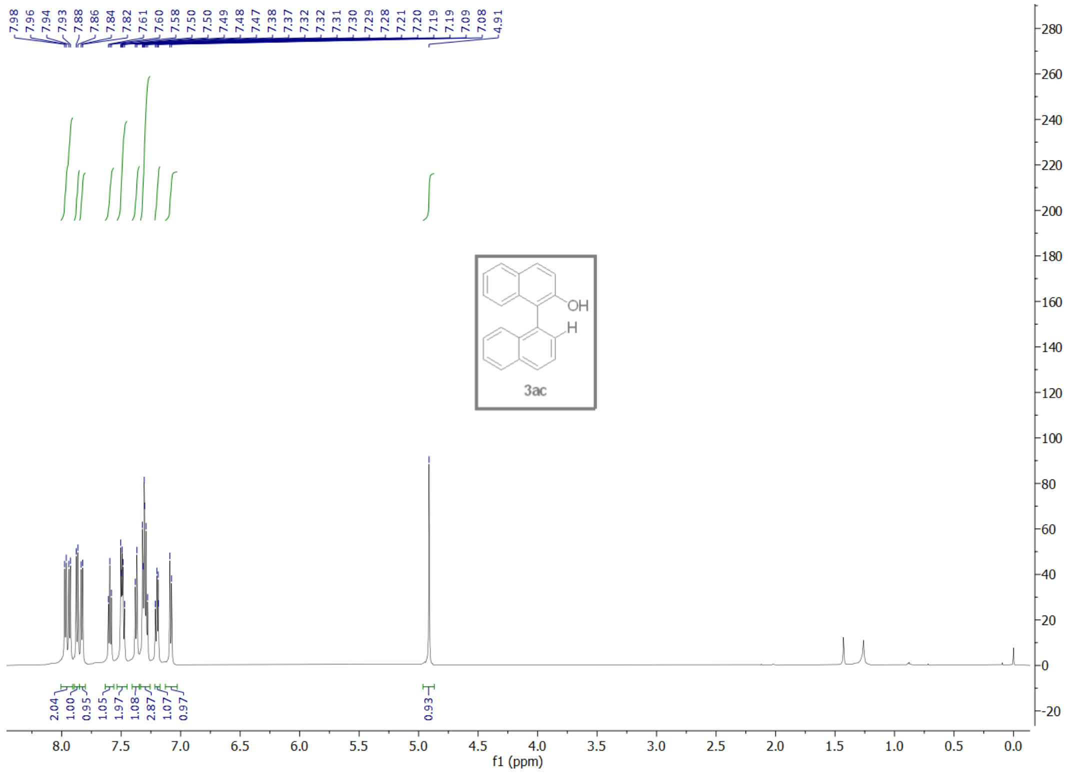

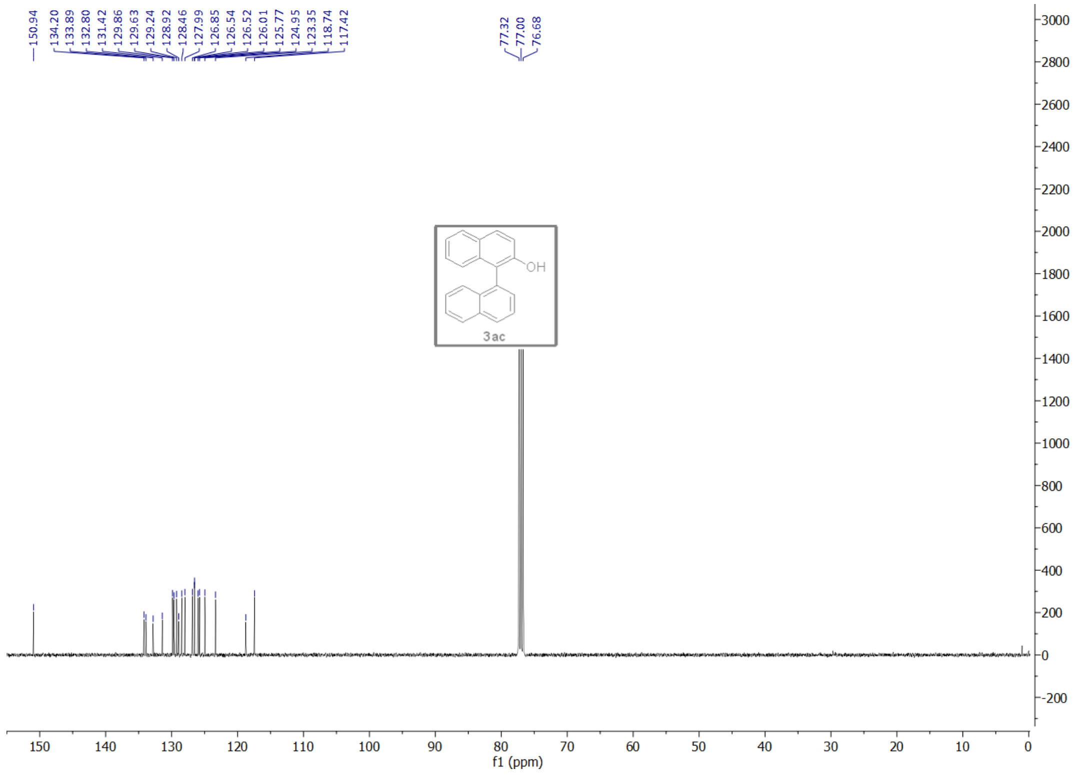

Supplementary Figure 52. ¹H NMR and ¹³C NMR spectrum of **3ac**.

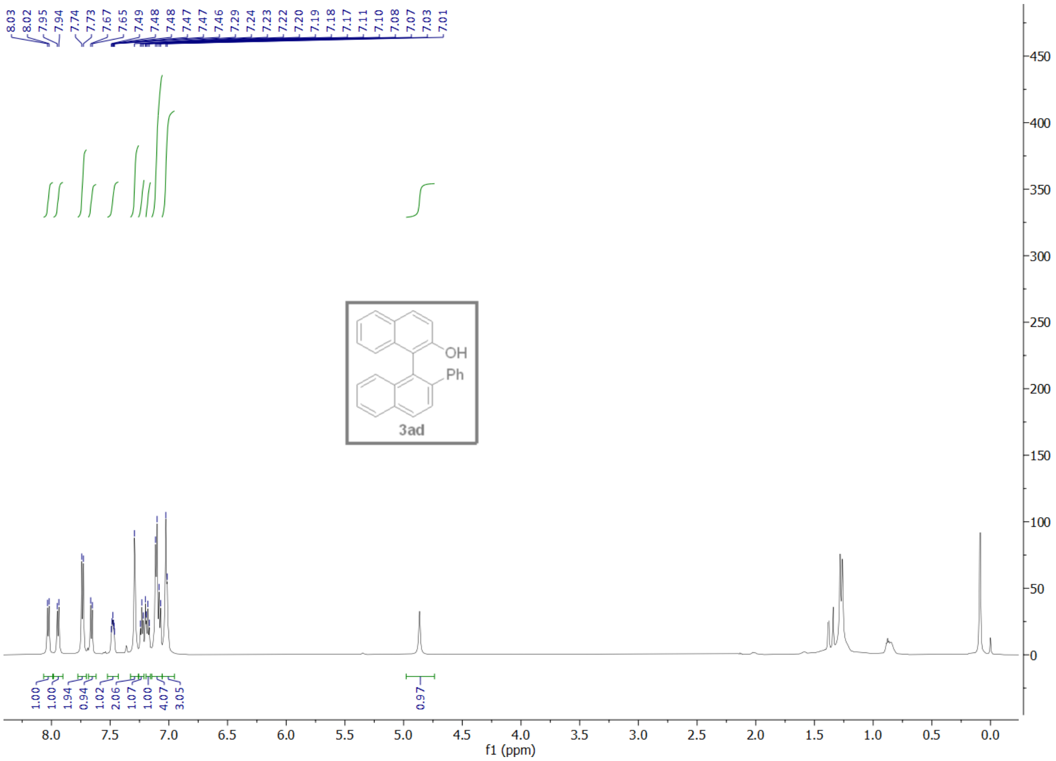

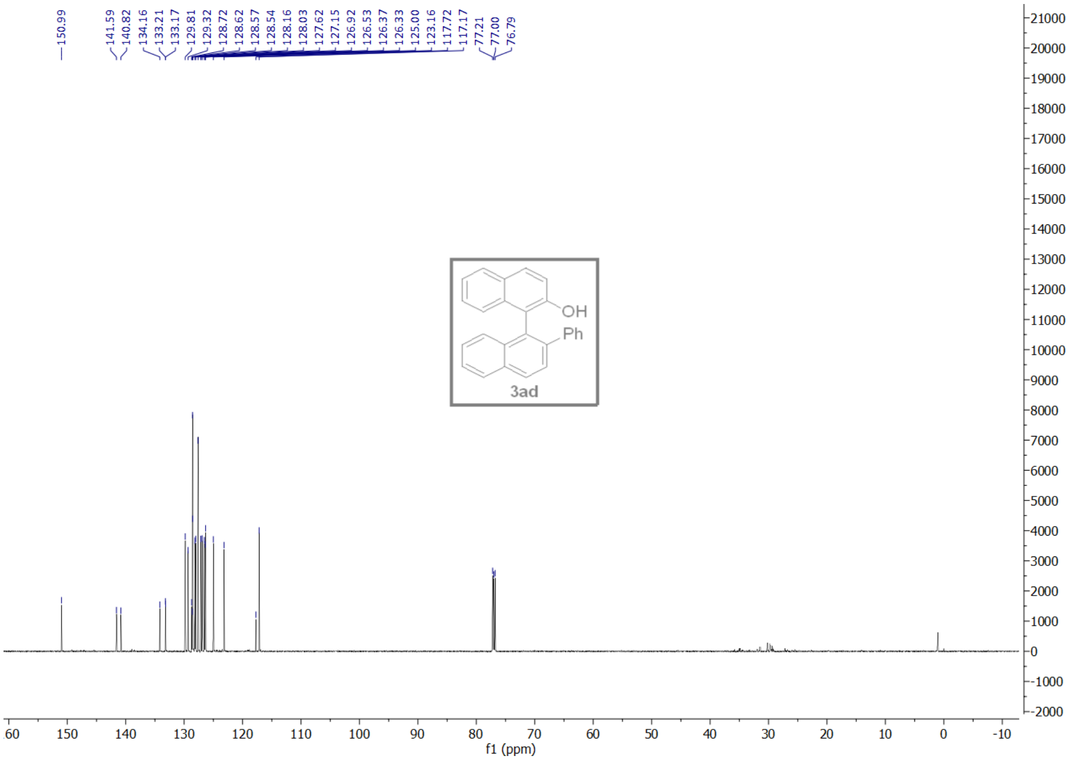

Supplementary Figure 53. ^{1}H NMR and ^{13}C NMR spectrum of **3ad**.

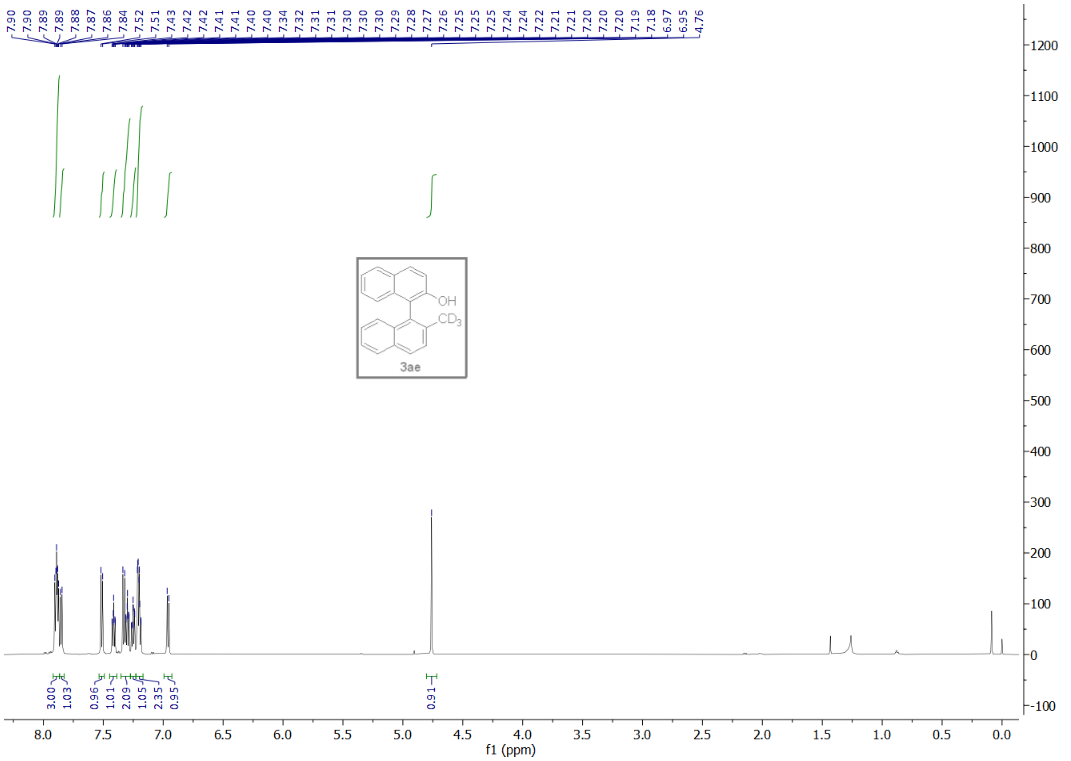

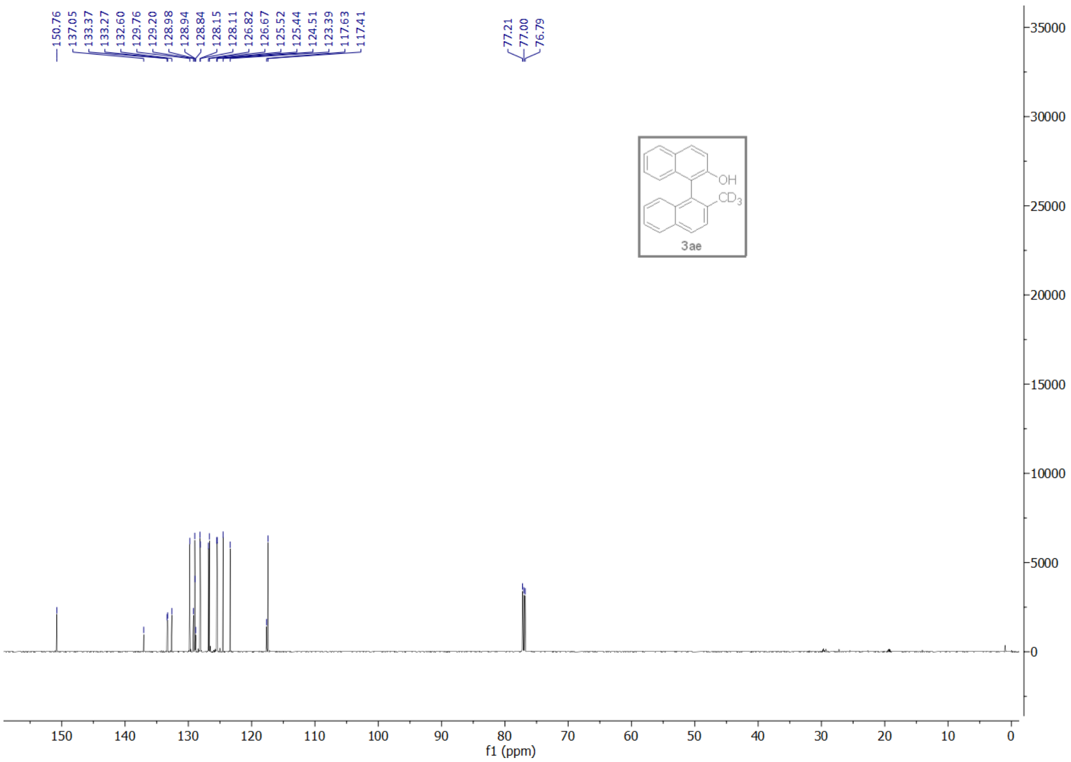

Supplementary Figure 54. ^{1}H NMR and ^{13}C NMR spectrum of **3ae**.

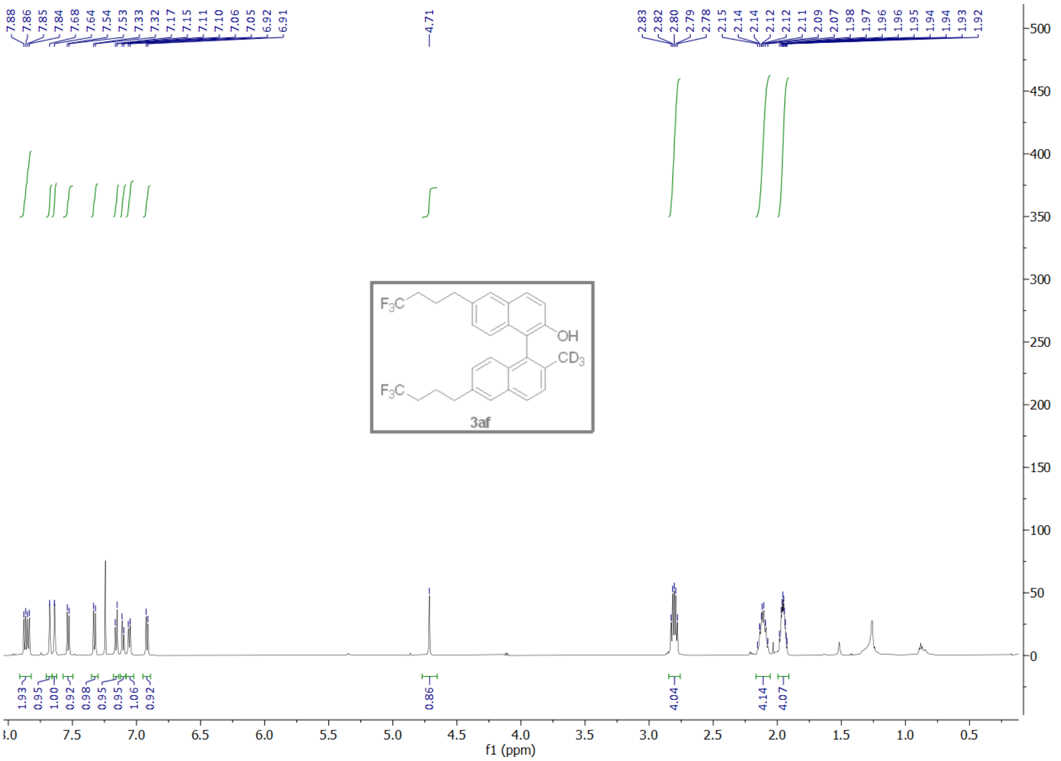

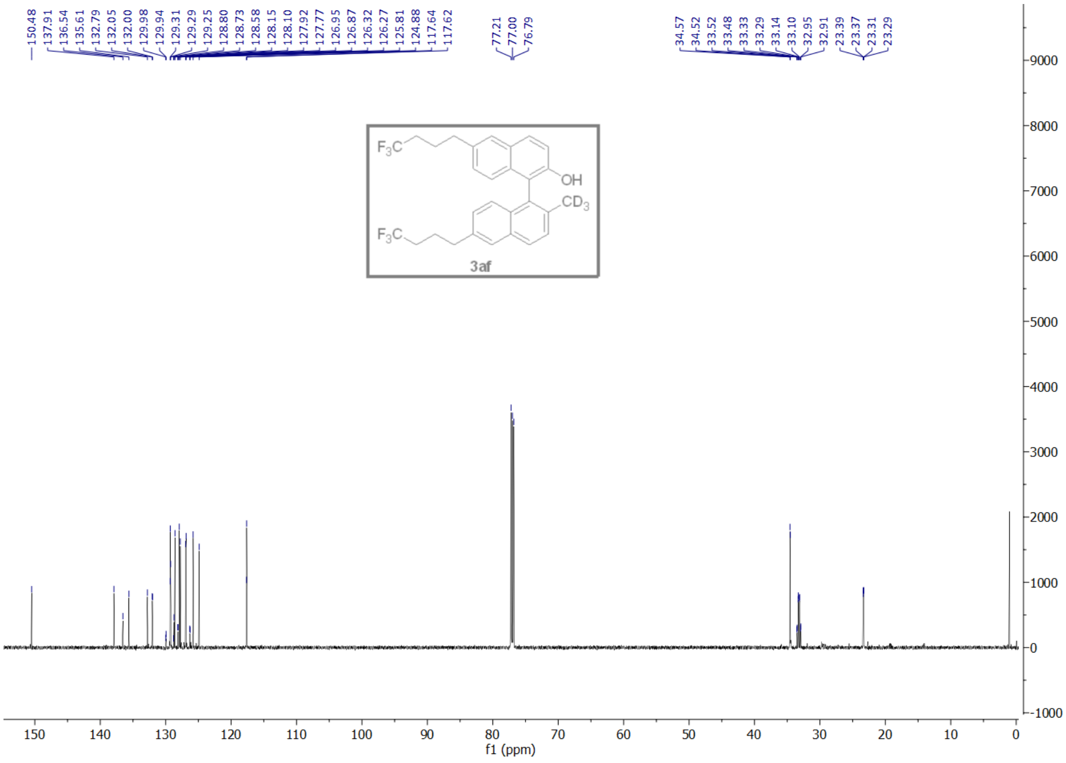

Supplementary Figure 55. ^{1}H NMR and ^{13}C NMR spectrum of **3af**.

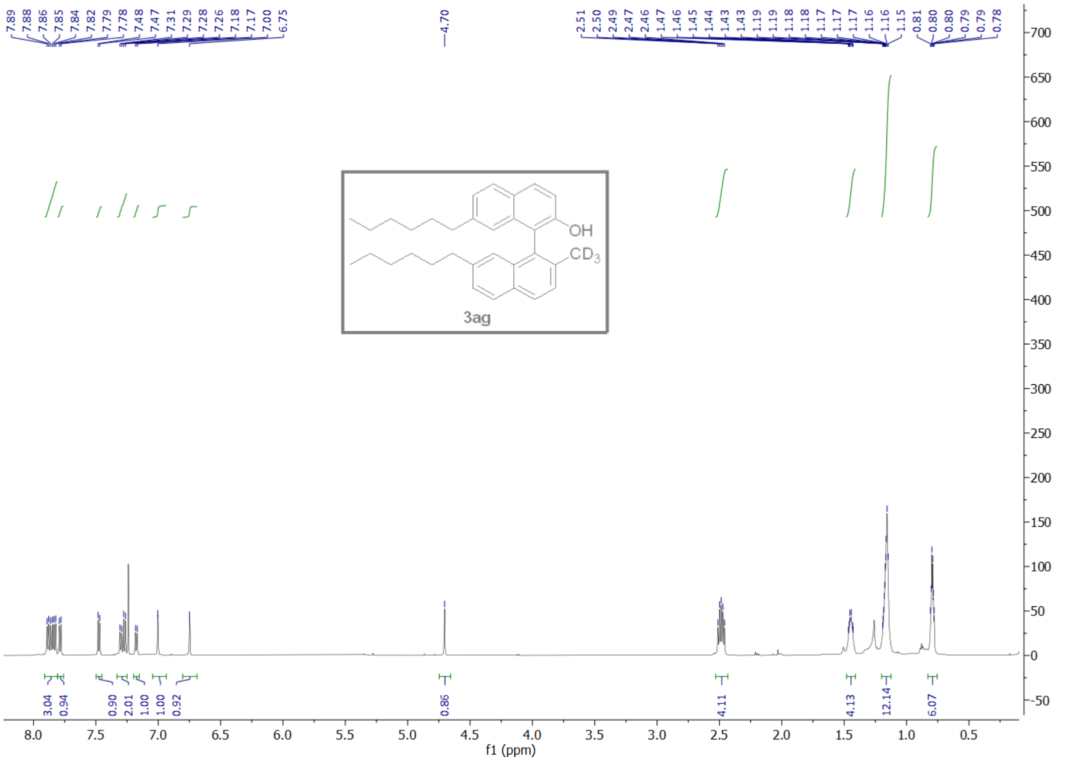

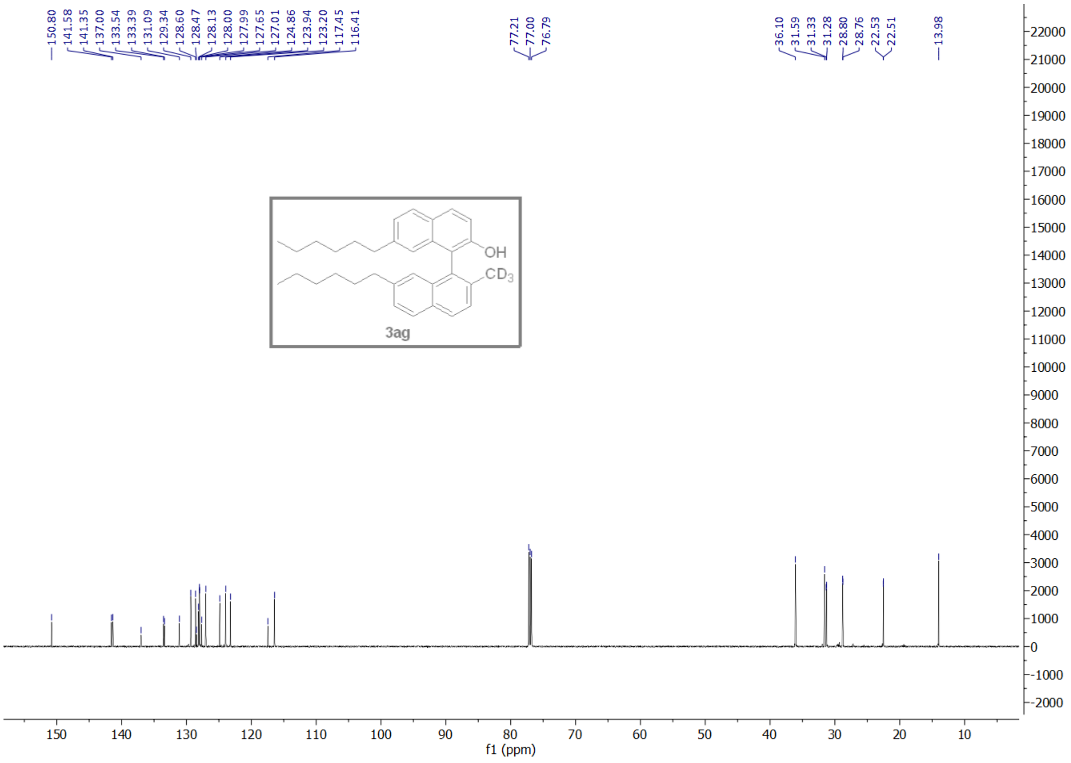

Supplementary Figure 56. ^{1}H NMR and ^{13}C NMR spectrum of **3ag**.

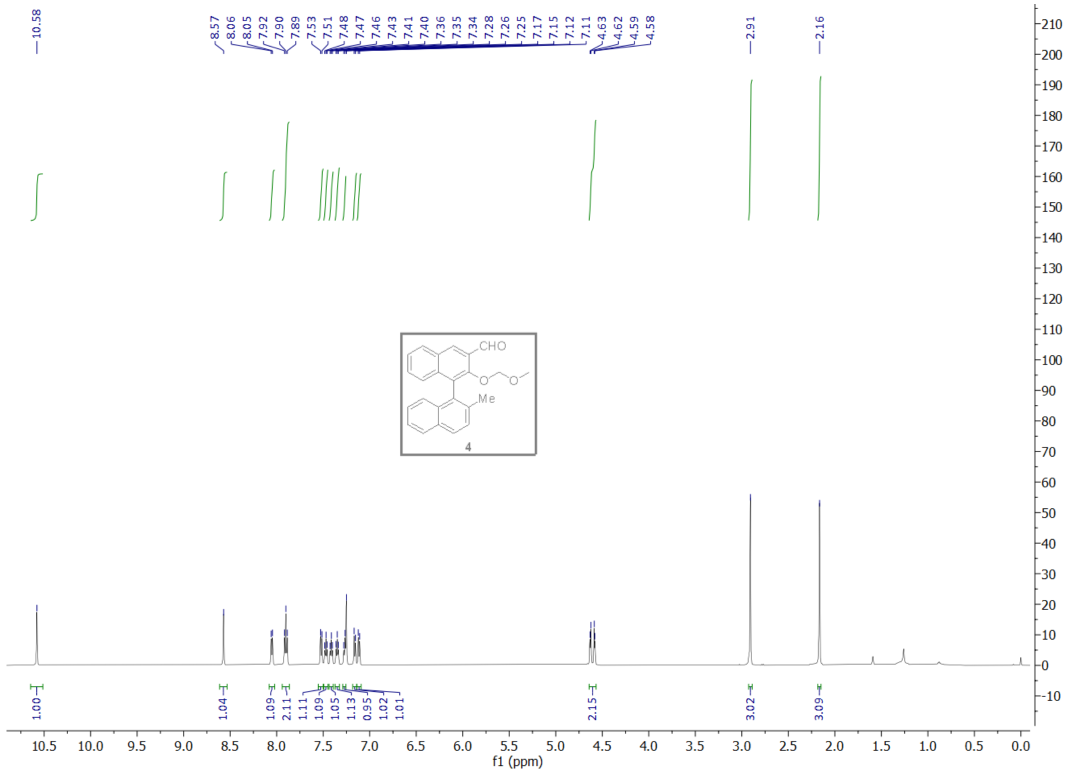

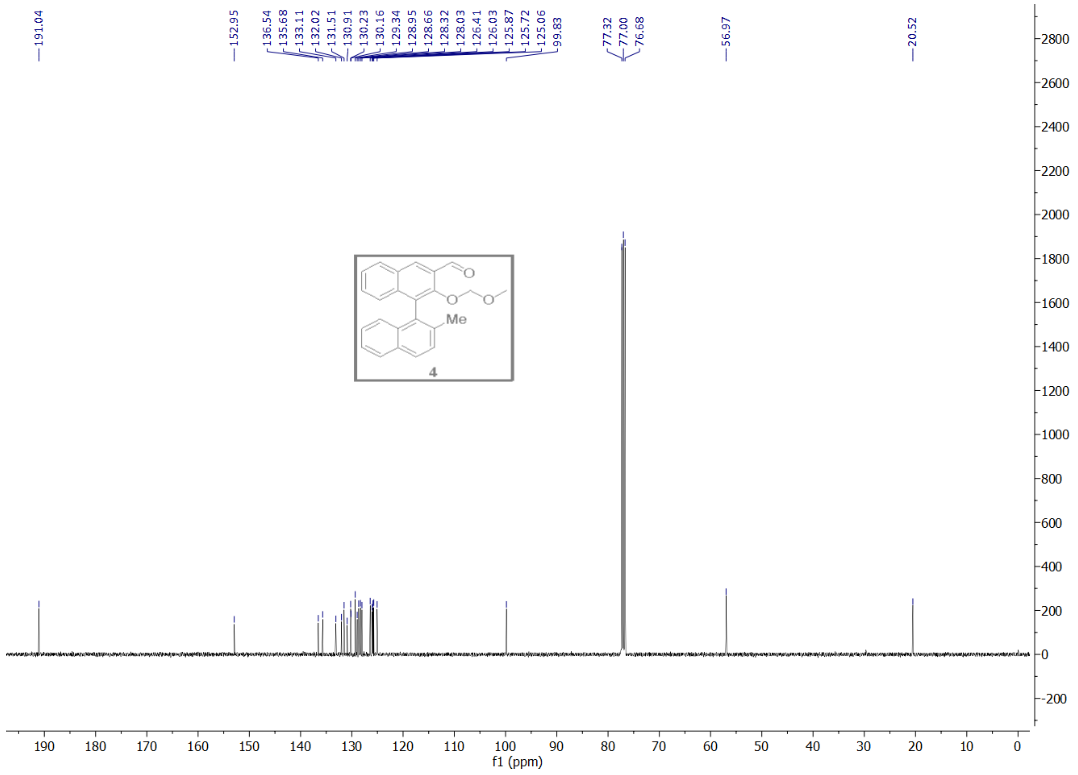

Supplementary Figure 57. ^{1}H NMR and ^{13}C NMR spectrum of **4**.

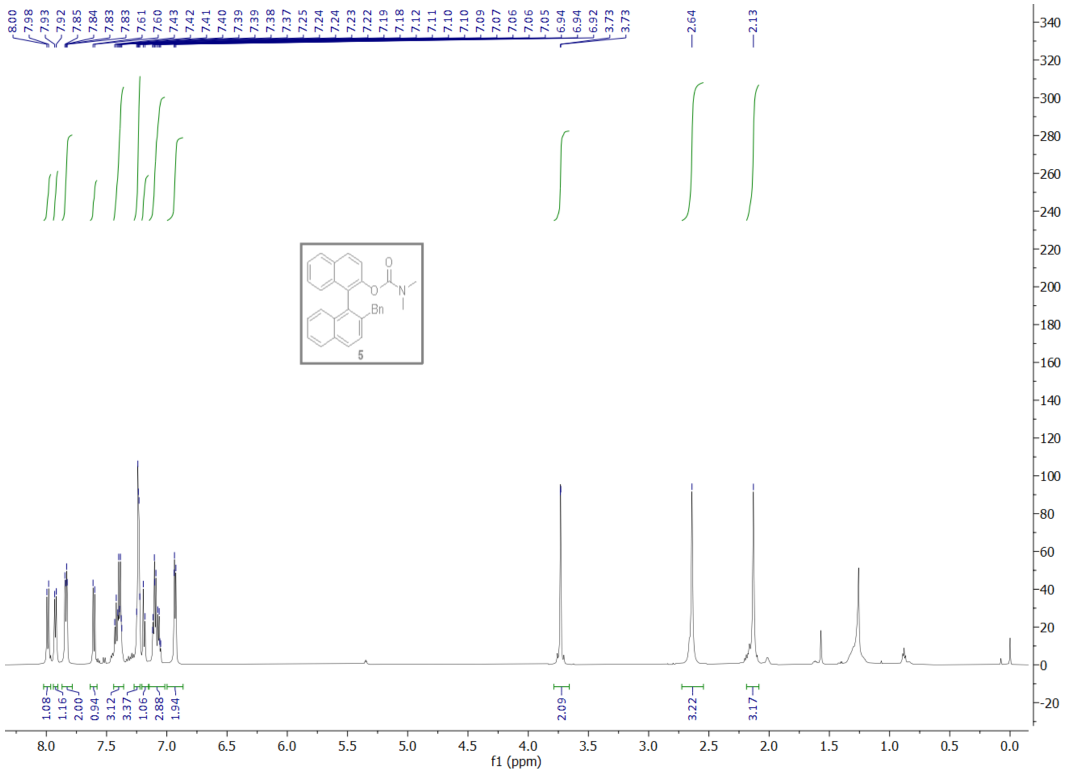

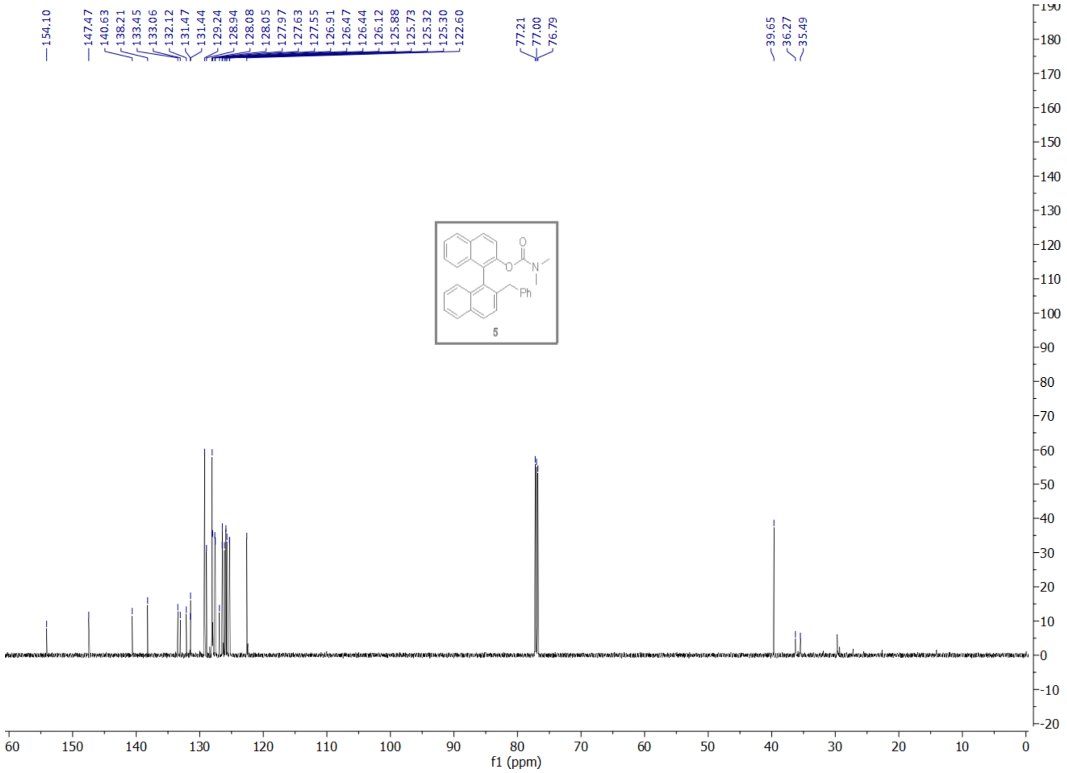

Supplementary Figure 58. ^{1}H NMR and ^{13}C NMR spectrum of **5**.

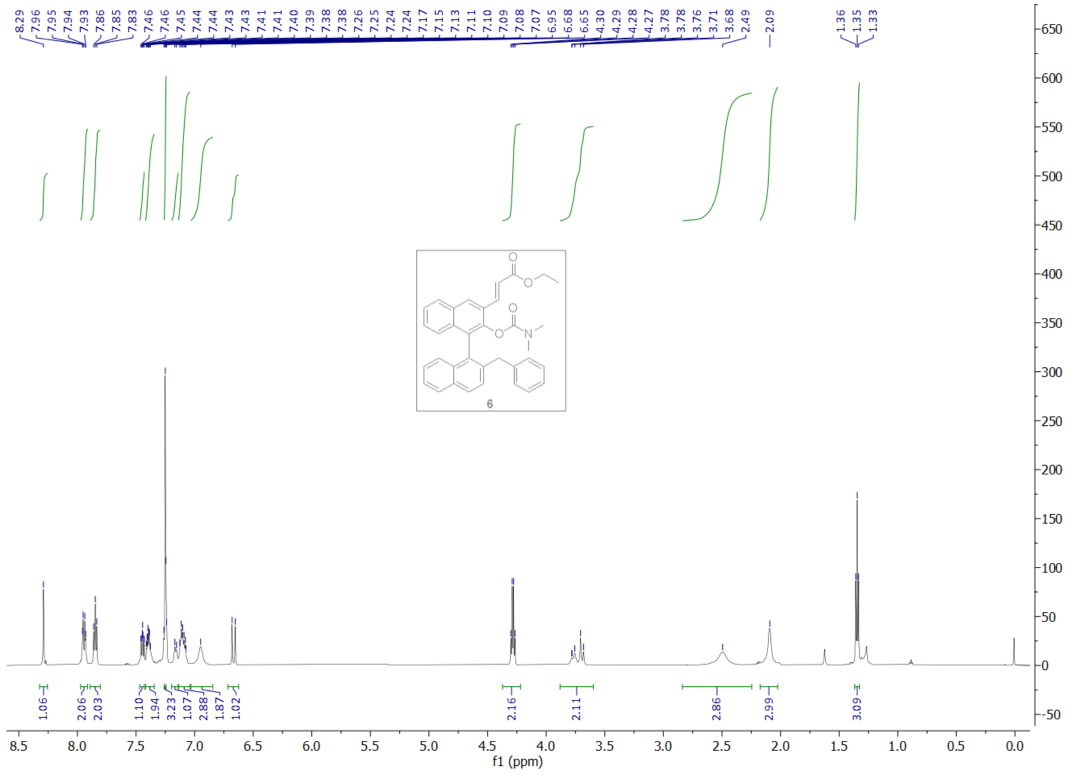

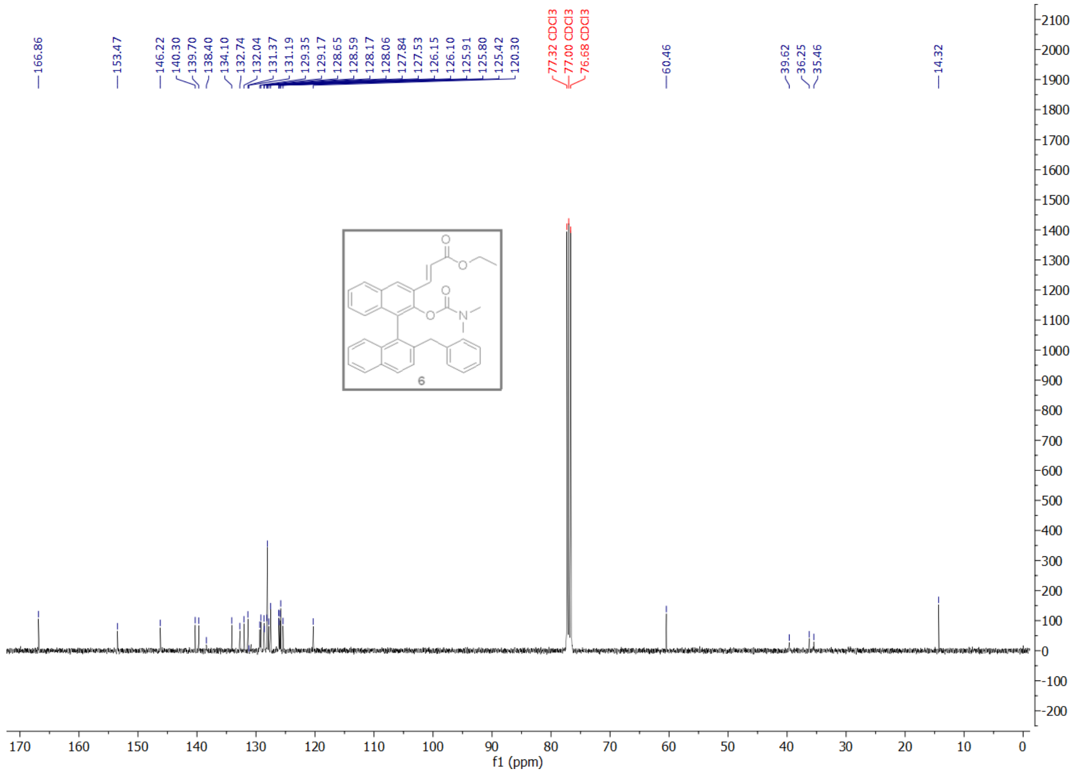

Supplementary Figure 59. ¹H NMR and ¹³C NMR spectrum of **6**.

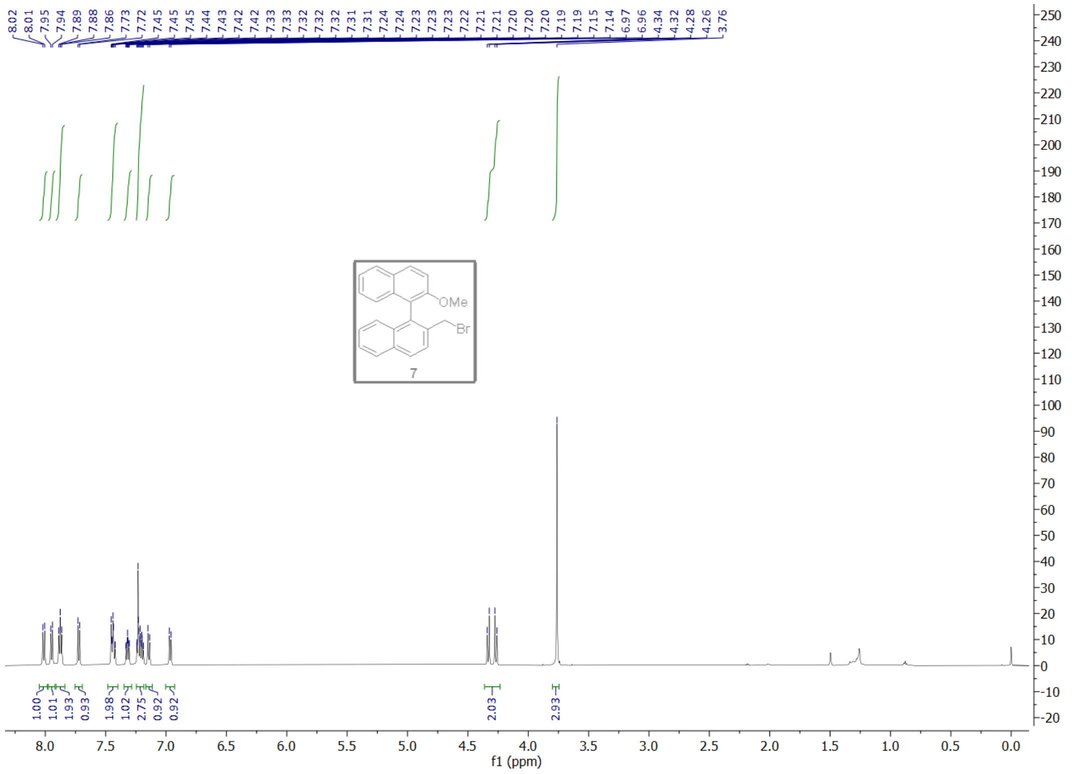

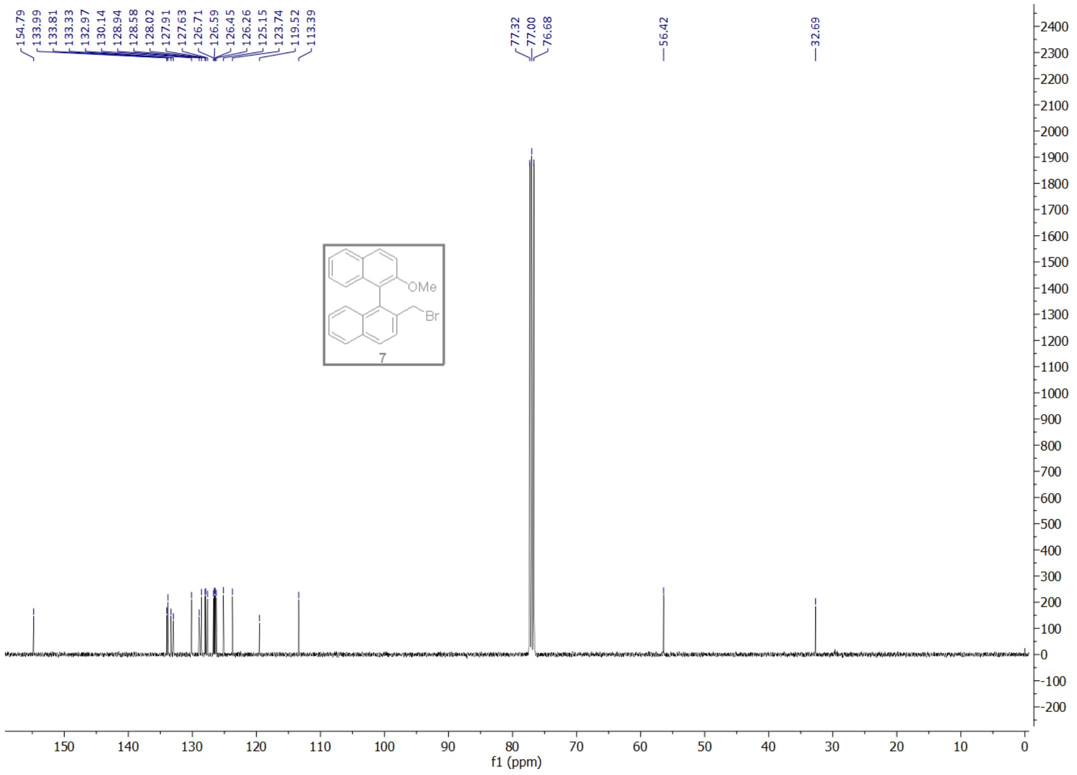

Supplementary Figure 60. ^{1}H NMR and ^{13}C NMR spectrum of **7**.

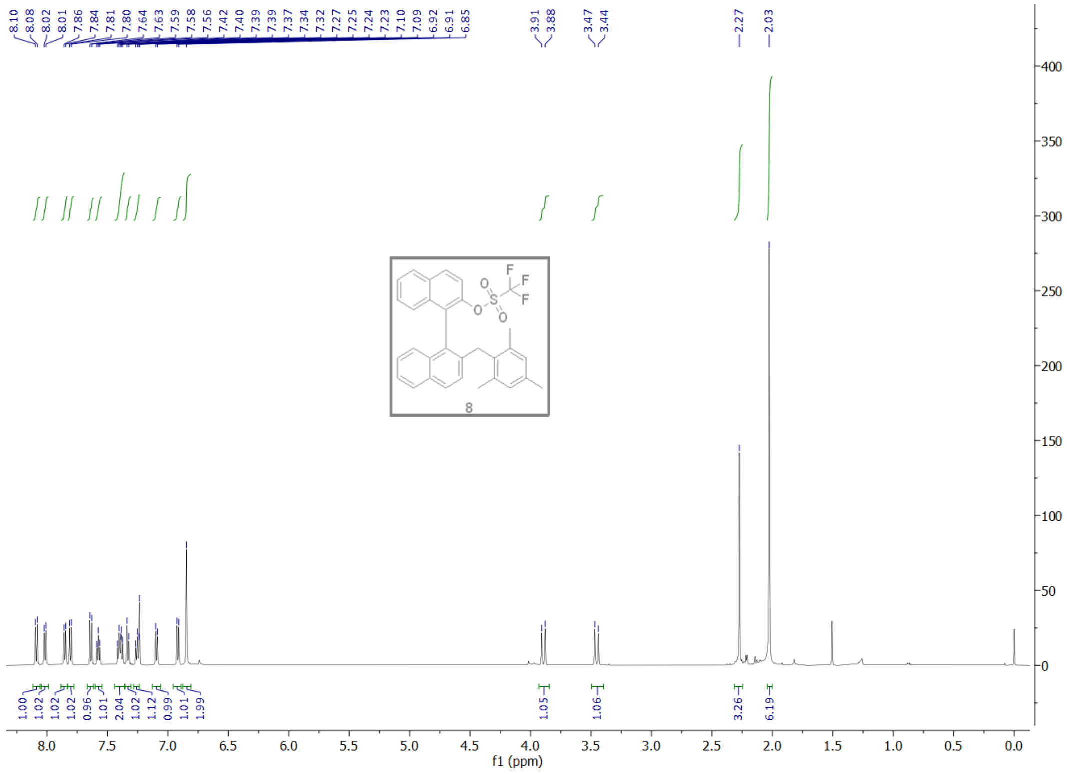

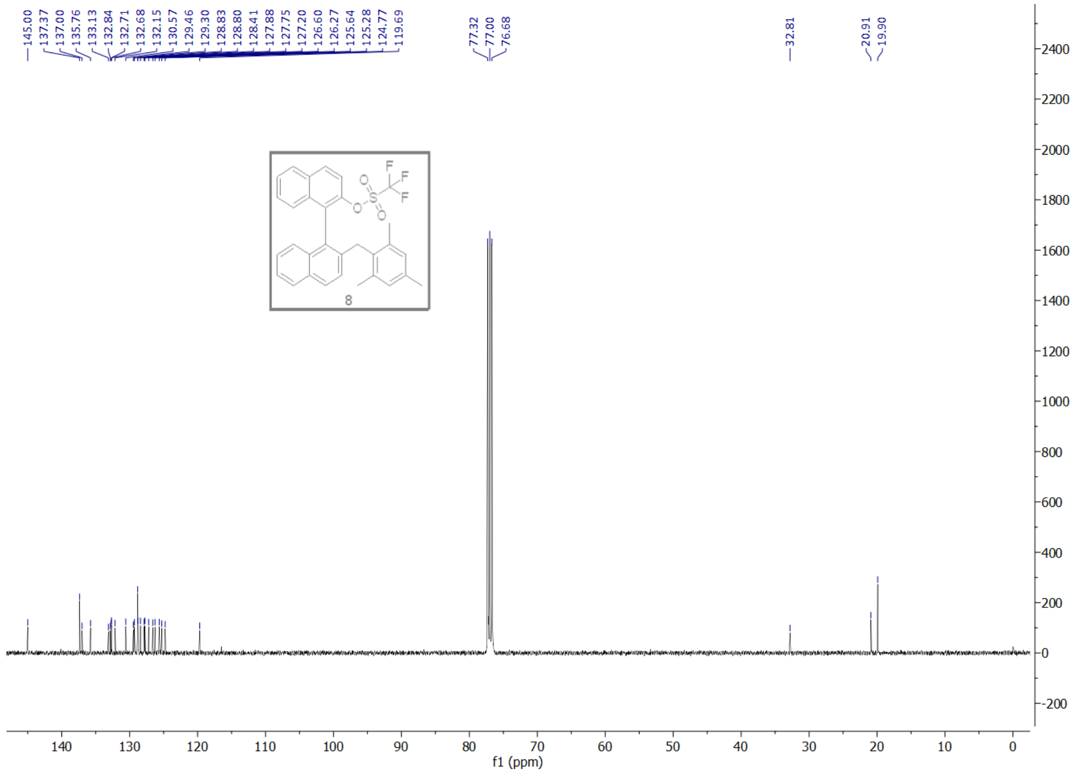

Supplementary Figure 61. ^{1}H NMR and ^{13}C NMR spectrum of **8**.

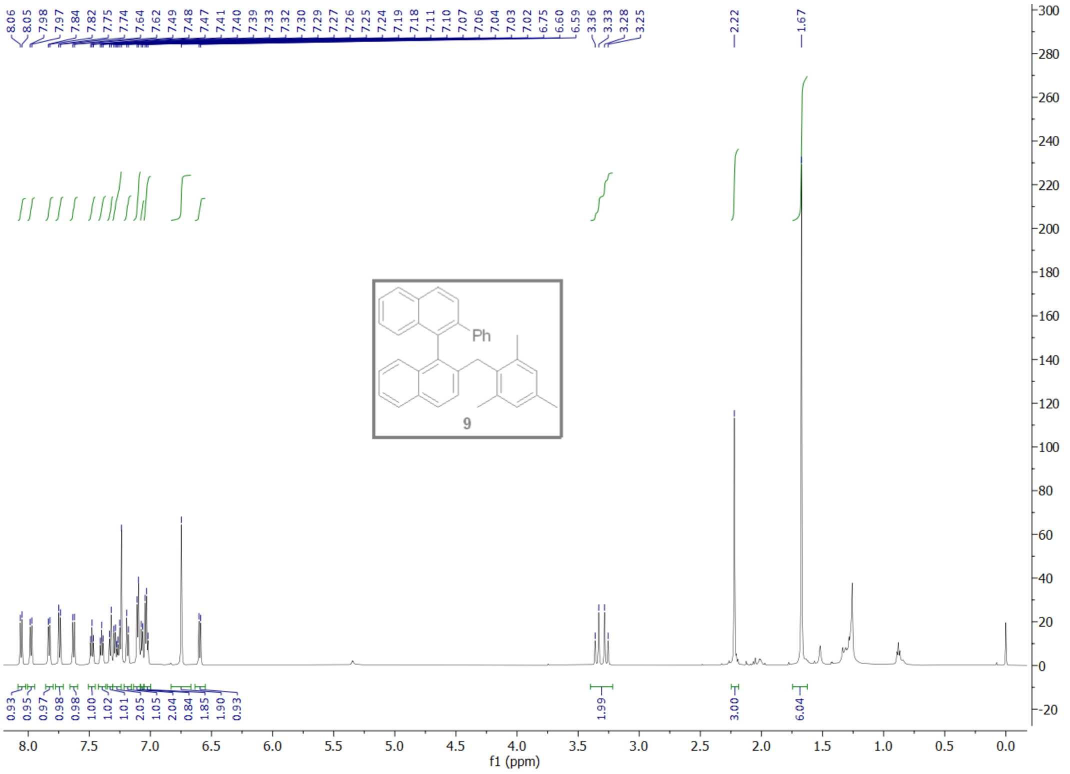

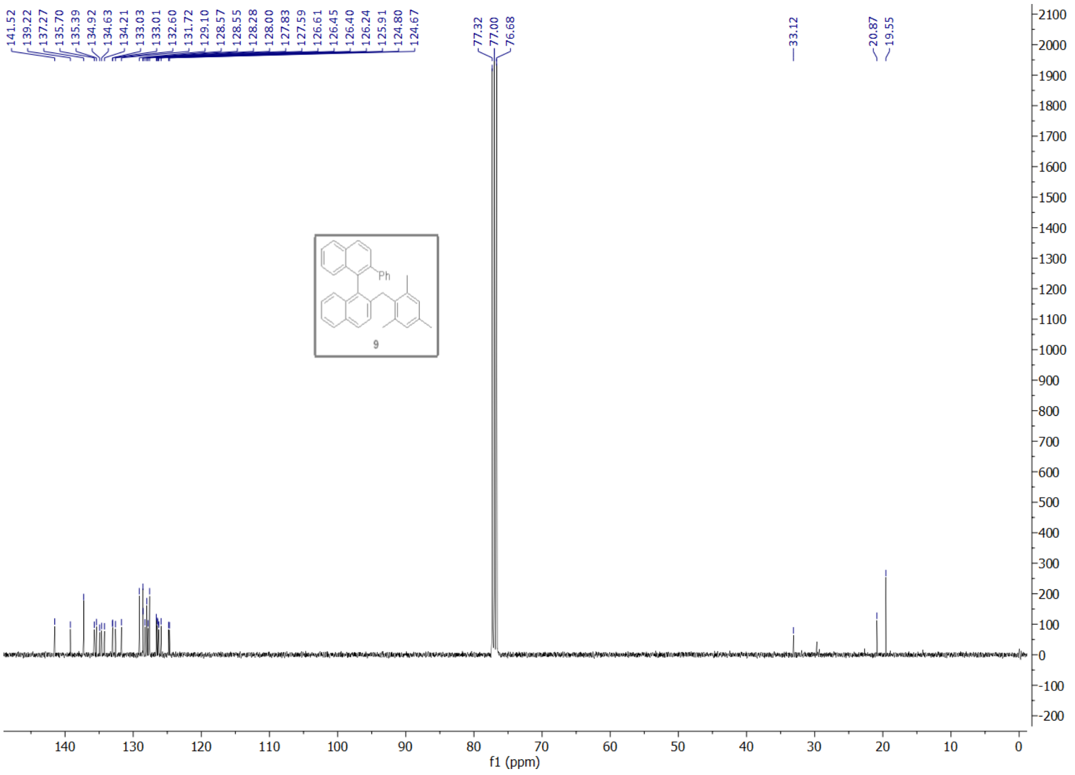

Supplementary Figure 62. ^{1}H NMR and ^{13}C NMR spectrum of **9**.

9. Supplementary figures of HPLC trace

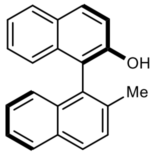

3a

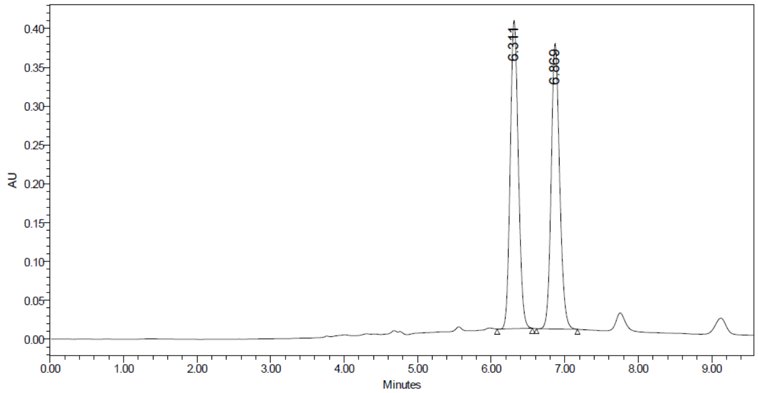

	RT (min)	Area (μV*sec)	% Area	Height (μV)	% Height
1	6.311	3034342	50.86	396970	51.92
2	6.869	2931211	49.14	367582	48.08

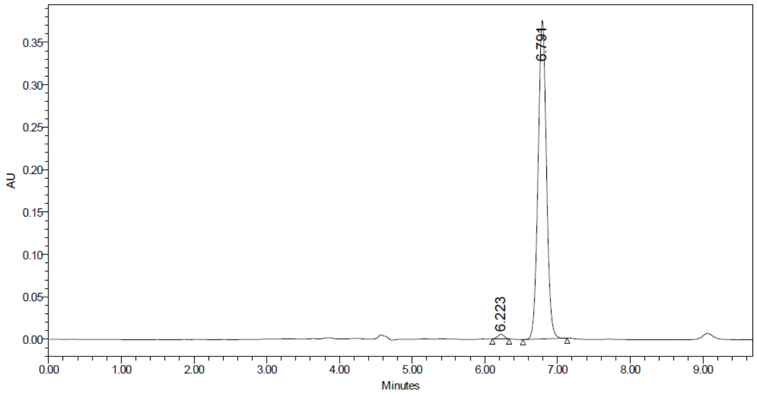

	RT (min)	Area (μV*sec)	% Area	Height (μV)	% Height
1	6.223	34342	1.13	5351	1.41
2	6.791	3003350	98.87	375357	98.59

Supplementary Figure 63. HPLC data of **3a**.

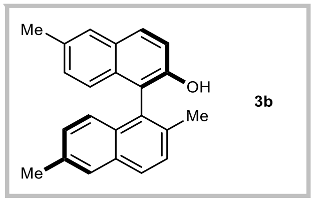

3b

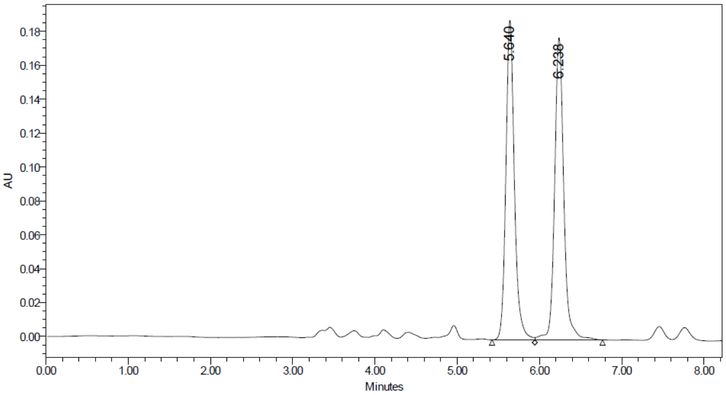

	RT (min)	Peak Type	Area (µV*sec)	% Area	Height (µV)	% Height	Integration Type	Points Across Peak	Start Time (min)	End Time (min)
1	5.640	Unknown	1356148	49.55	188579	51.39	BV	312	5.423	5.943
2	6.238	Unknown	1380995	50.45	178366	48.61	VB	494	5.943	6.767

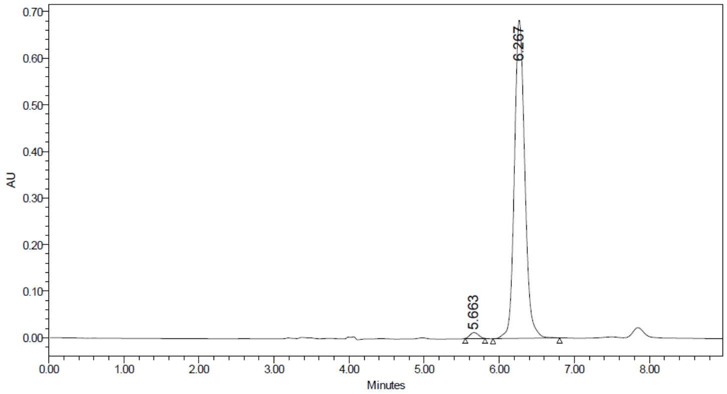

	RT (min)	Peak Type	Area (µV*sec)	% Area	Height (µV)	% Height	Integration Type	Points Across Peak	Start Time (min)	End Time (min)
1	5.663	Unknown	97027	1.45	12420	1.79	bb	157	5.548	5.810
2	6.267	Unknown	6577932	98.55	683133	98.21	bb	532	5.917	6.803

Supplementary Figure 64. HPLC data of **3b**.

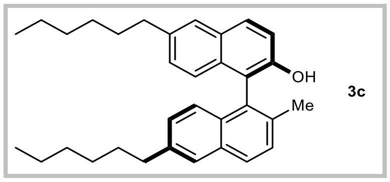

3c

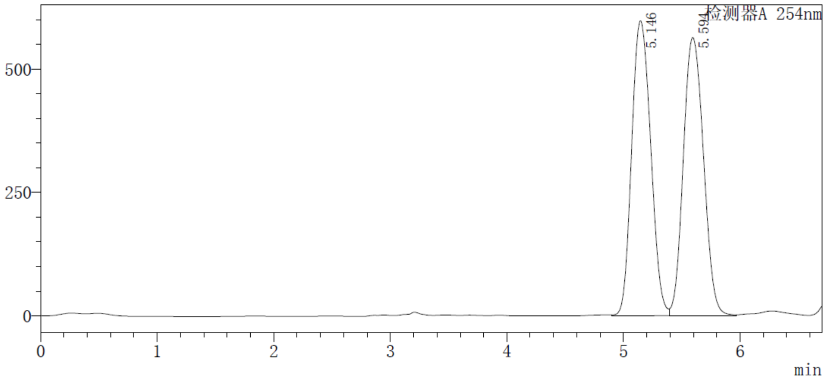

〈峰表〉
检测器A 254nm

峰号	保留时间	面积	高度	浓度	浓度单位	标记	化合物名
1	5.146	6803853	596712	50.089			
2	5.594	6779629	563353	49.911		V	
总计		13583481	1160065				

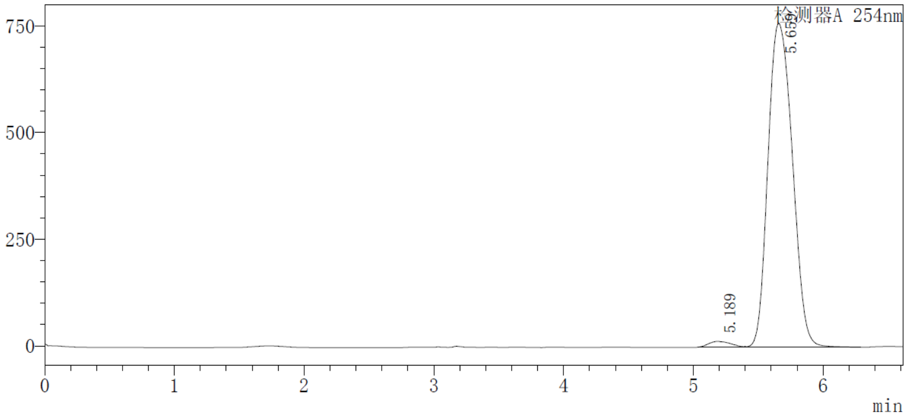

〈峰表〉
检测器A 254nm

峰号	保留时间	面积	高度	浓度	浓度单位	标记	化合物名
1	5.189	159745	13472	1.582		M	
2	5.659	9937833	759173	98.418		V	
总计		10097578	772645				

Supplementary Figure 65. HPLC data of **3c**.

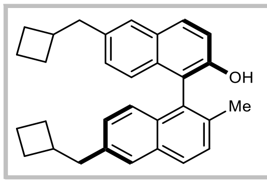

3d

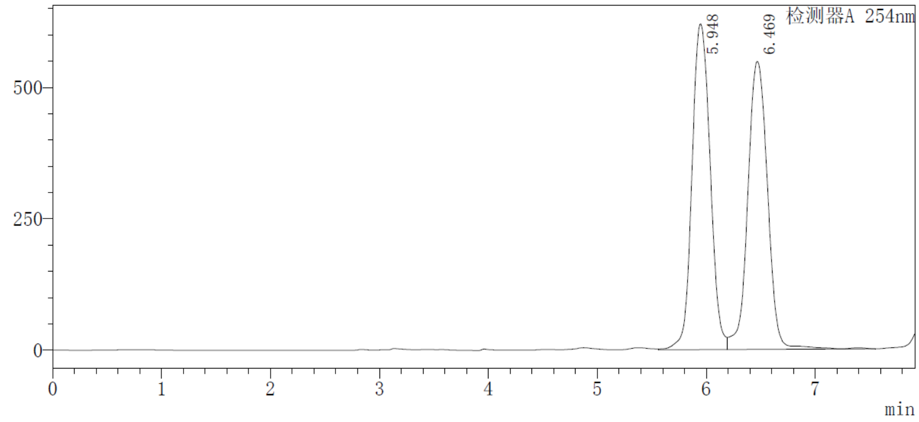

<峰表>
检测器A 254nm

峰号	保留时间	面积	高度	浓度	浓度单位	标记	化合物名
1	5.948	7201403	619791	50.892			
2	6.469	6948993	548096	49.108		V	
总计		14150396	1167888				

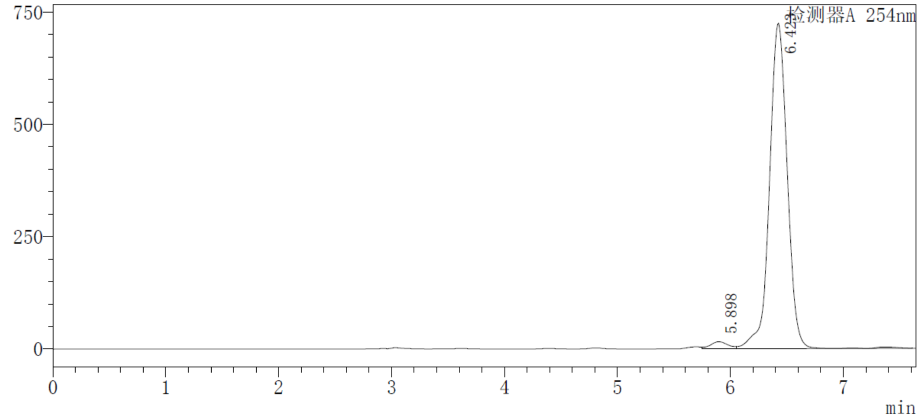

<峰表>
检测器A 254nm

峰号	保留时间	面积	高度	浓度	浓度单位	标记	化合物名
1	5.898	167527	15551	2.070			
2	6.423	7925224	724552	97.930		V	
总计		8092751	740103				

Supplementary Figure 66. HPLC data of **3d**.

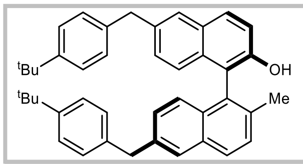

3e

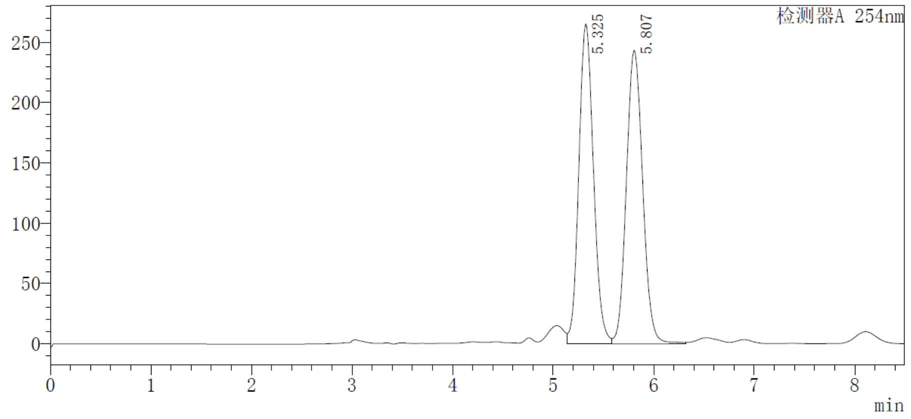

<峰表>

检测器A 254nm

峰号	保留时间	面积	高度	浓度	浓度单位	标记	化合物名
1	5.325	2682676	265252	49.335			
2	5.807	2755017	243266	50.665		V	
总计		5437693	508518				

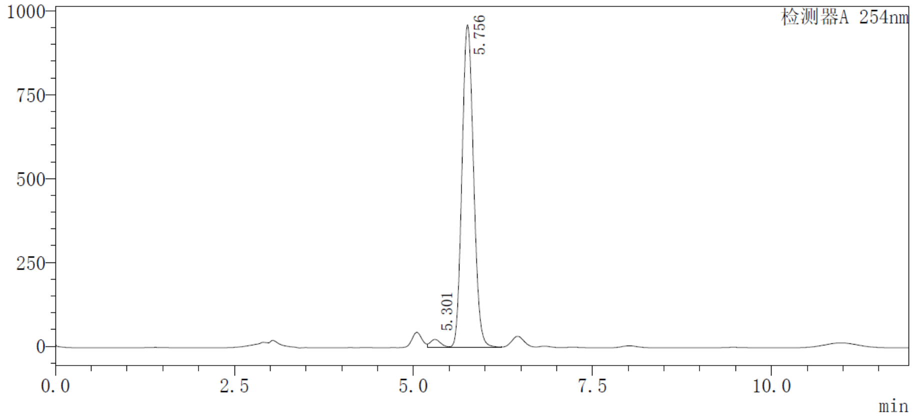

<峰表>

检测器A 254nm

峰号	保留时间	面积	高度	浓度	浓度单位	标记	化合物名
1	5.301	264957	25020	2.384			
2	5.756	10847578	963271	97.616		V	
总计		11112535	988291				

Supplementary Figure 67. HPLC data of **3e**.

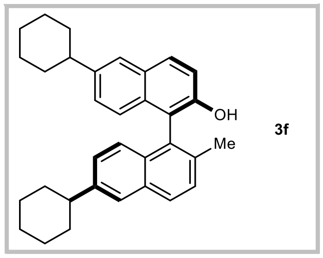

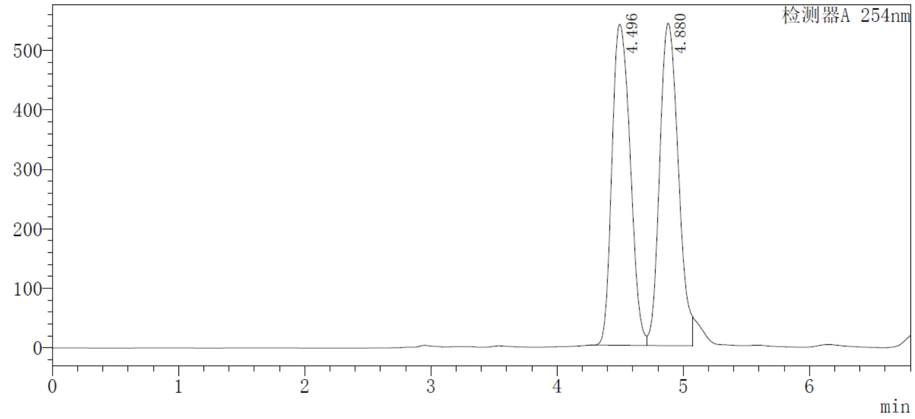

〈峰表〉
检测器A 254nm

峰号	保留时间	面积	高度	浓度	浓度单位	标记	化合物名
1	4. 496	5420692	539113	49. 345		M	
2	4. 880	5564647	541418	50. 655		V M	
总计		10985340	1080531				

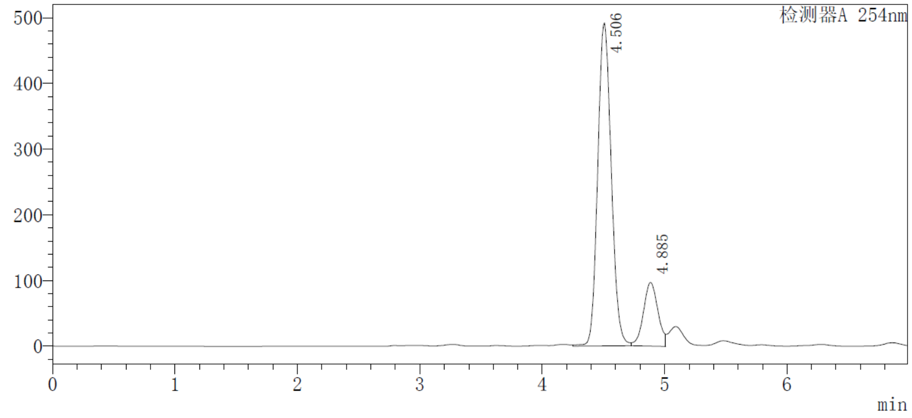

〈峰表〉
检测器A 254nm

峰号	保留时间	面积	高度	浓度	浓度单位	标记	化合物名
1	4. 506	3783677	492107	82. 581		M	
2	4. 885	798113	97079	17. 419		M	
总计		4581790	589186				

Supplementary Figure 68. HPLC data of **3f**.

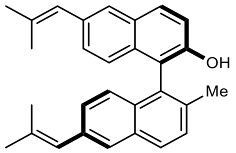

3g

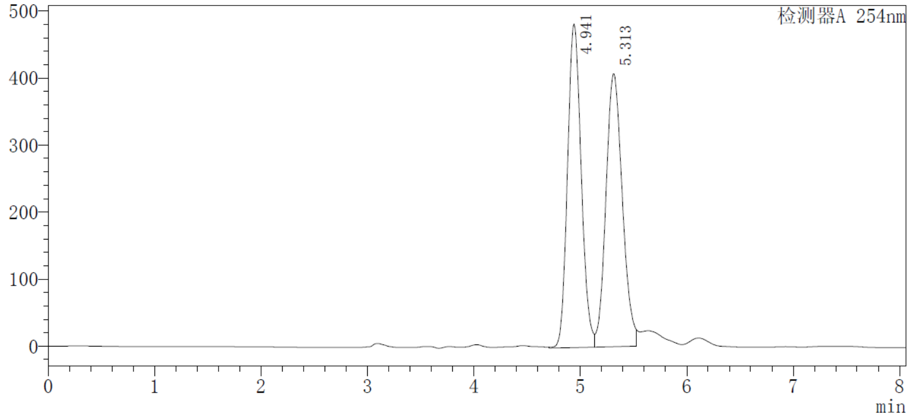

<峰表>
检测器A 254nm

峰号	保留时间	面积	高度	浓度	浓度单位	标记	化合物名
1	4.941	4328976	482940	49.737			
2	5.313	4374730	407251	50.263		V M	
总计		8703706	890190				

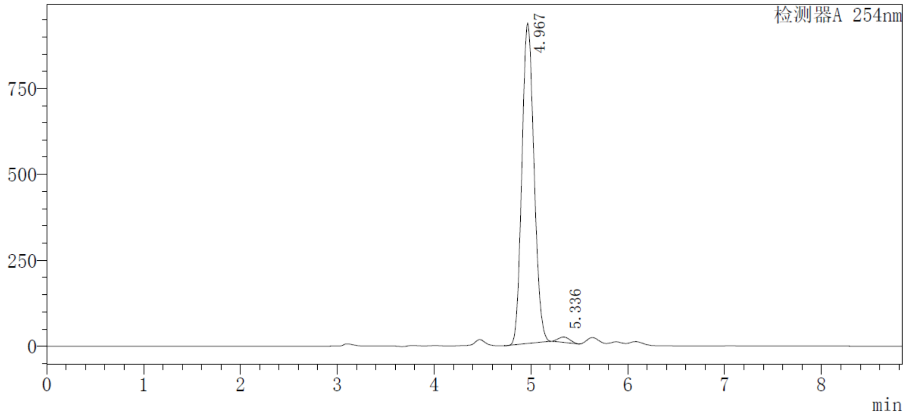

<峰表>
检测器A 254nm

峰号	保留时间	面积	高度	浓度	浓度单位	标记	化合物名
1	4.967	8125066	932916	98.500		M	
2	5.336	123691	15353	1.500		M	
总计		8248758	948269				

Supplementary Figure 69. HPLC data of **3g**.

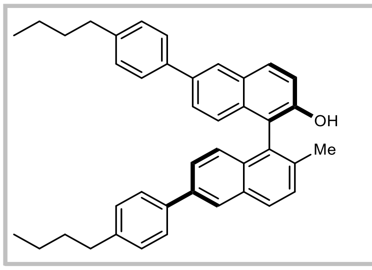

3h

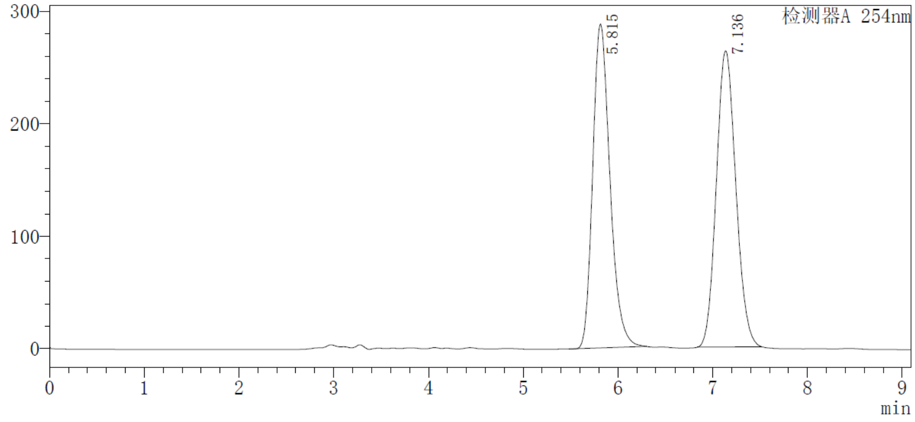

<峰表>
检测器A 254nm

峰号	保留时间	面积	高度	浓度	浓度单位	标记	化合物名
1	5.815	3640721	288064	49.129		M	
2	7.136	3769831	263359	50.871		M	
总计		7410553	551422				

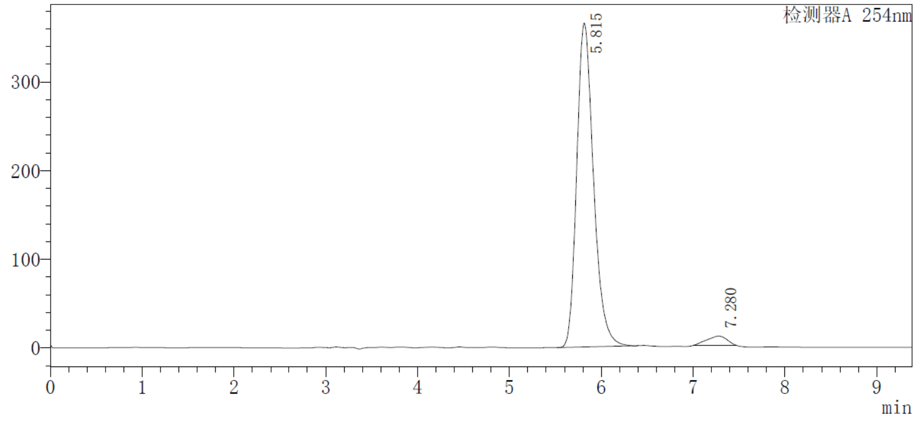

<峰表>
检测器A 254nm

峰号	保留时间	面积	高度	浓度	浓度单位	标记	化合物名
1	5.815	4649825	365514	96.596		M	
2	7.280	163850	10588	3.404		M	
总计		4813674	376102				

Supplementary Figure 70. HPLC data of **3h**.

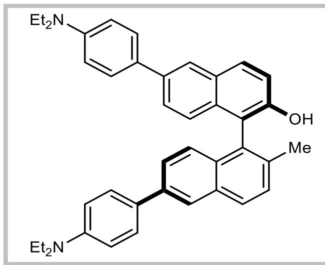

3i

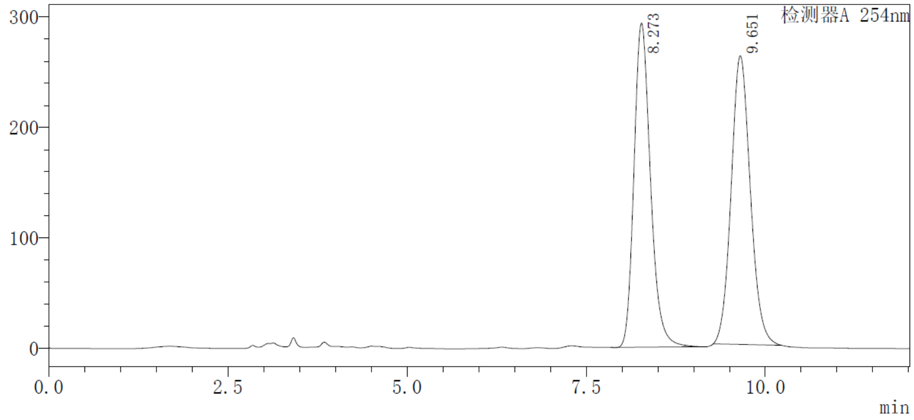

〈峰表〉
检测器A 254nm

峰号	保留时间	面积	高度	浓度	浓度单位	标记	化合物名
1	8.273	4705505	293474	49.125		M	
2	9.651	4873156	261490	50.875		M	
总计		9578661	554964				

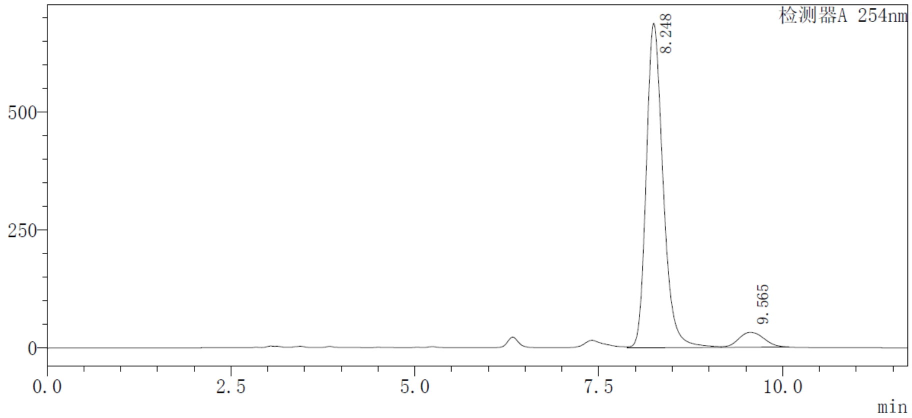

〈峰表〉
检测器A 254nm

峰号	保留时间	面积	高度	浓度	浓度单位	标记	化合物名
1	8.248	10964477	687831	93.576			
2	9.565	752704	31387	6.424		V M	
总计		11717181	719218				

Supplementary Figure 71. HPLC data of **3i**.

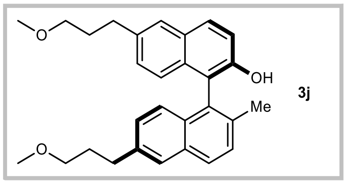

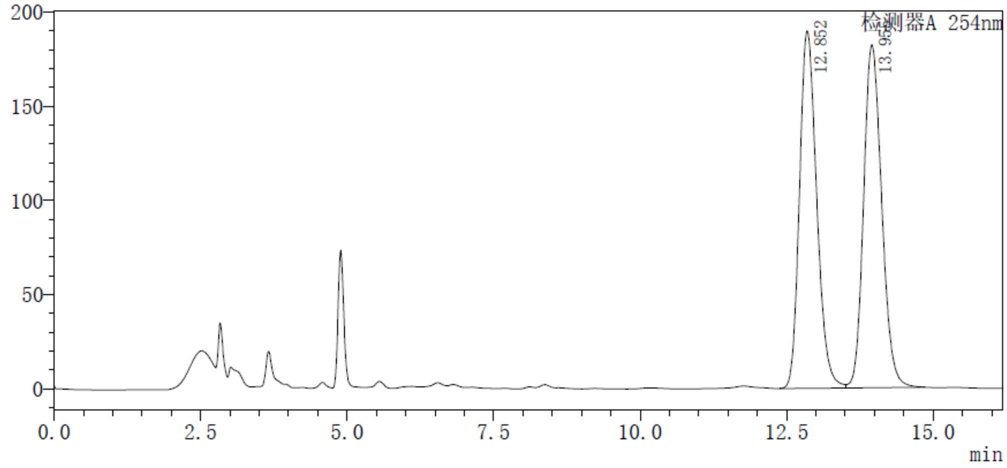

<峰表>
检测器A 254nm

峰号	保留时间	面积	高度	浓度	浓度单位	标记	化合物名
1	12.852	3878643	189648	49.777		M	
2	13.955	3913371	181965	50.223		V M	
总计		7792014	371613				

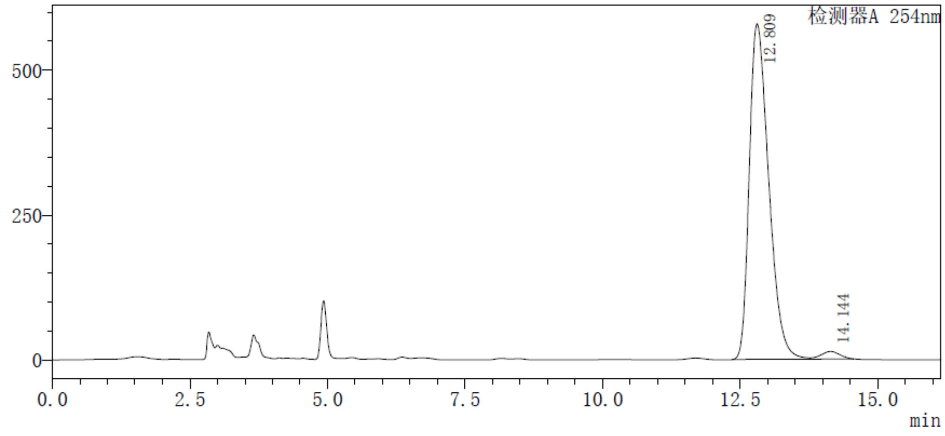

<峰表>
检测器A 254nm

峰号	保留时间	面积	高度	浓度	浓度单位	标记	化合物名
1	12.809	14216276	578961	97.894		M	
2	14.144	305842	12780	2.106		V M	
总计		14522118	591741				

Supplementary Figure 72. HPLC data of **3j**.

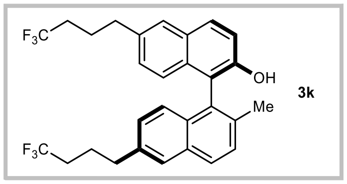

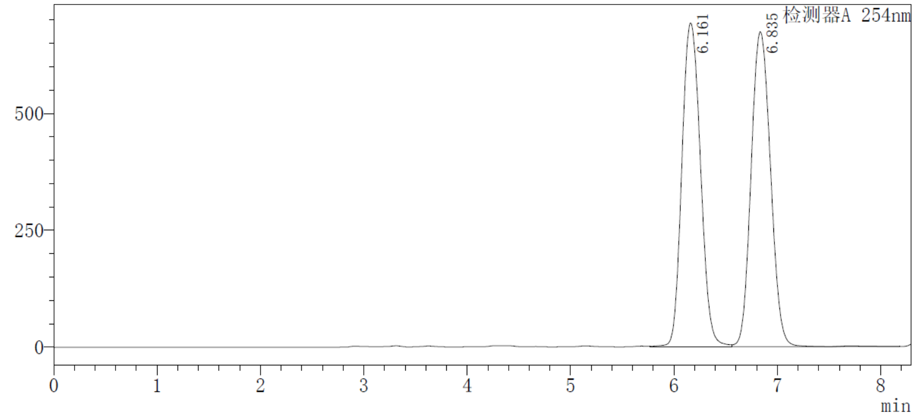

<峰表>
检测器A 254nm

峰号	保留时间	面积	高度	浓度	浓度单位	标记	化合物名
1	6.161	8670833	692796	49.630			
2	6.835	8800002	673802	50.370		V	
总计		17470836	1366598				

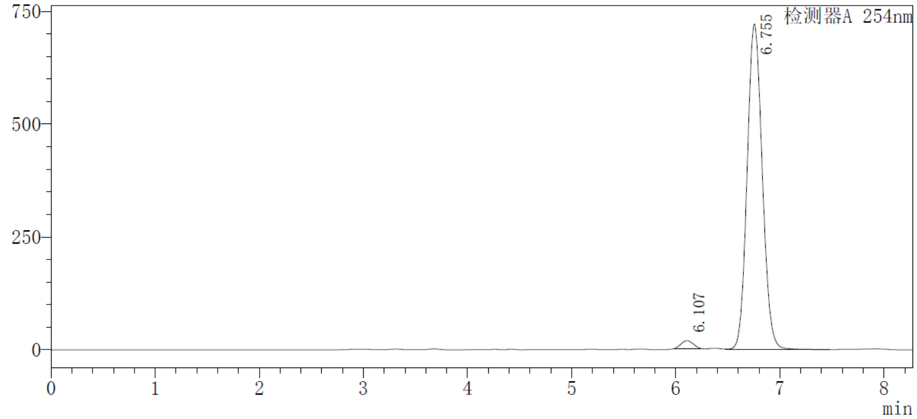

<峰表>
检测器A 254nm

峰号	保留时间	面积	高度	浓度	浓度单位	标记	化合物名
1	6.107	133623	17116	1.789		M	
2	6.755	7337017	721435	98.211			
总计		7470640	738551				

Supplementary Figure 73. HPLC data of **3k**.

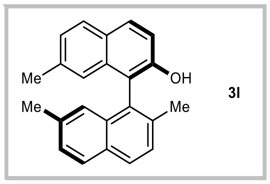

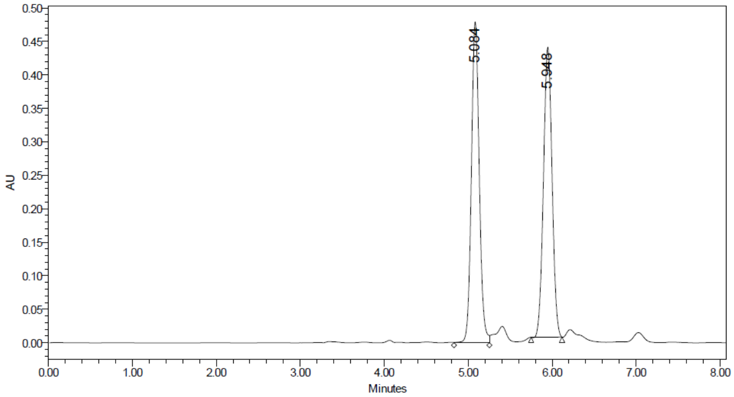

	RT (min)	Peak Type	Area (µV*sec)	% Area	Height (µV)	% Height	Integration Type	Points Across Peak	Start Time (min)	End Time (min)
1	5.084	Unknown	3106655	50.19	479293	52.55	VV	253	4.832	5.253
2	5.948	Unknown	3083488	49.81	432848	47.45	bb	220	5.750	6.117

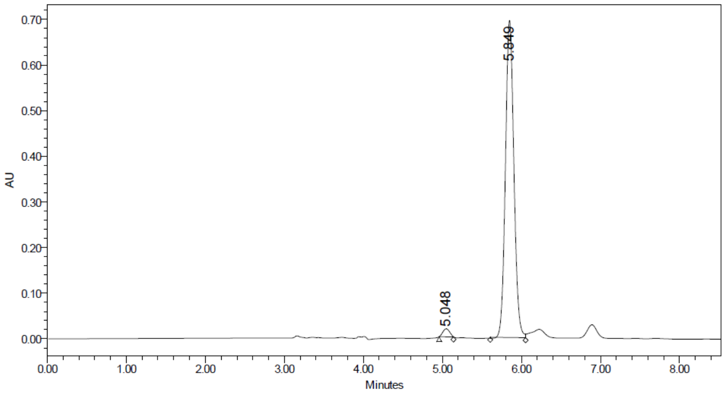

	RT (min)	Peak Type	Area (µV*sec)	% Area	Height (µV)	% Height	Integration Type	Points Across Peak	Start Time (min)	End Time (min)
1	5.048	Unknown	105519	2.01	17503	2.46	bv	109	4.958	5.140
2	5.849	Unknown	5149157	97.99	694861	97.54	VV	269	5.605	6.053

Supplementary Figure 74. HPLC data of **3l**.

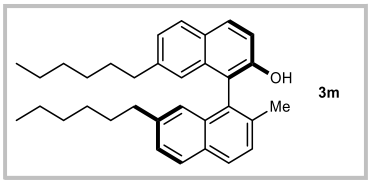

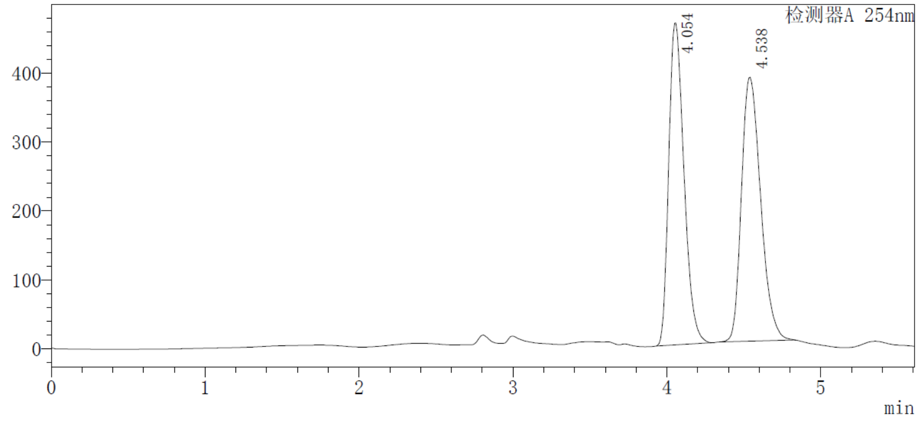

<峰表>
检测器A 254nm

峰号	保留时间	面积	高度	浓度	浓度单位	标记	化合物名
1	4. 054	3213695	466969	49. 179		M	
2	4. 538	3321028	382841	50. 821		M	
总计		6534723	849810				

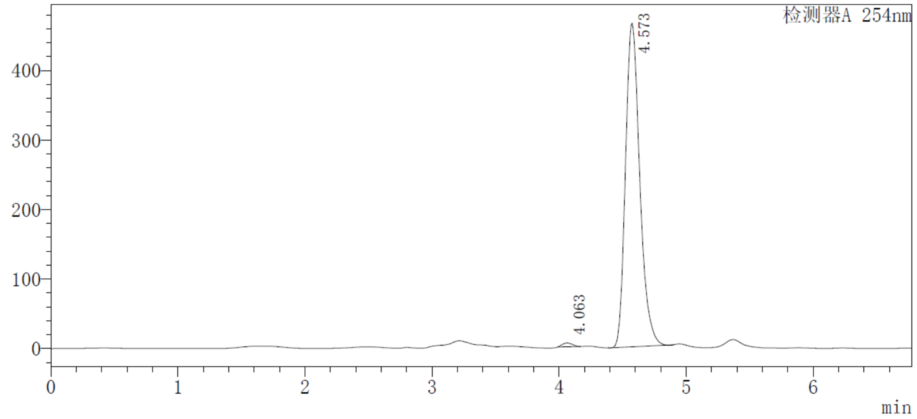

<峰表>
检测器A 254nm

峰号	保留时间	面积	高度	浓度	浓度单位	标记	化合物名
1	4. 063	29899	5381	0. 815		M	
2	4. 573	3640624	465692	99. 185		M	
总计		3670524	471073				

Supplementary Figure 75. HPLC data of **3m**.

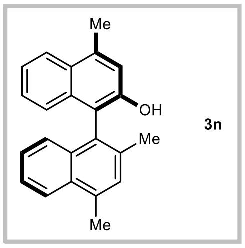

3n

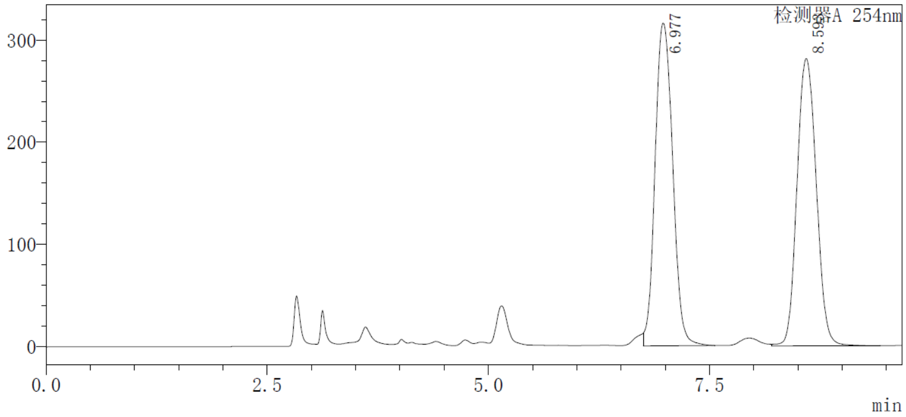

〈峰表〉
检测器A 254nm

峰号	保留时间	面积	高度	浓度	浓度单位	标记	化合物名
1	6.977	4373706	315996	50.574		M	
2	8.593	4274404	281596	49.426			
总计		8648110	597592				

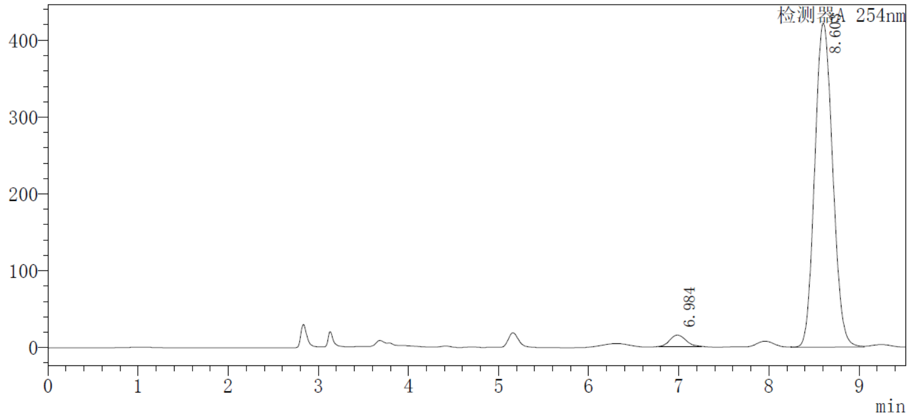

〈峰表〉
检测器A 254nm

峰号	保留时间	面积	高度	浓度	浓度单位	标记	化合物名
1	6.984	175492	14577	2.917		M	
2	8.603	5841535	421491	97.083			
总计		6017027	436067				

Supplementary Figure 76. HPLC data of **3n**.

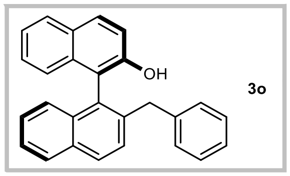

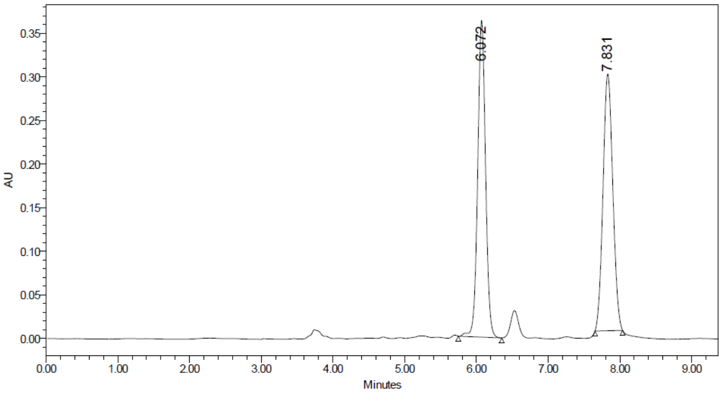

	RT (min)	Peak Type	Area (µV*sec)	% Area	Height (µV)	% Height	Integration Type	Points Across Peak	Start Time (min)	End Time (min)
1	6.072	Unknown	2762750	49.73	363265	55.24	bb	364	5.747	6.353
2	7.831	Unknown	2792461	50.27	294383	44.76	bb	231	7.653	8.038

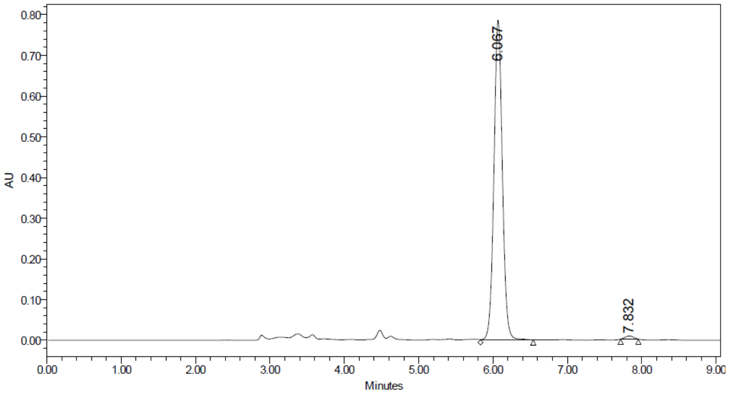

	RT (min)	Peak Type	Area (µV*sec)	% Area	Height (µV)	% Height	Integration Type	Points Across Peak	Start Time (min)	End Time (min)
1	6.067	Unknown	6181429	99.02	785717	99.02	VB	426	5.832	6.542
2	7.832	Unknown	61238	0.98	7812	0.98	bb	143	7.715	7.953

Supplementary Figure 77. HPLC data of **3o**.

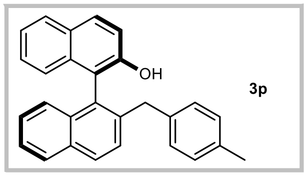

3p

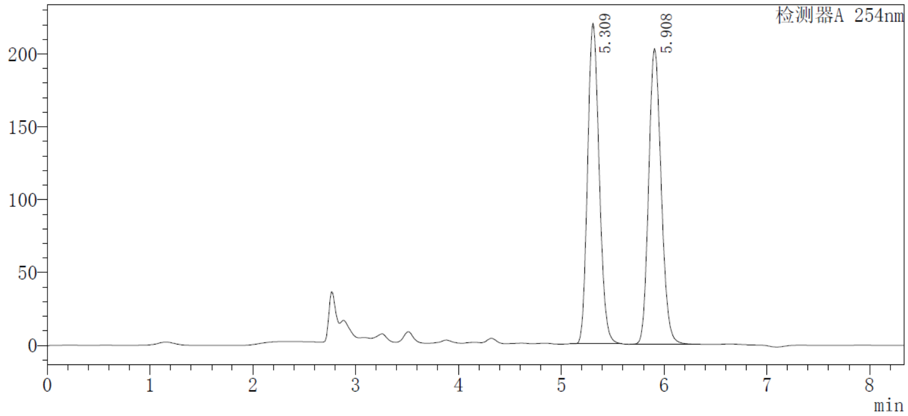

<峰表>
检测器A 254nm

峰号	保留时间	面积	高度	浓度	浓度单位	标记	化合物名
1	5.309	1720956	220095	49.982		M	
2	5.908	1722184	203032	50.018		M	
总计		3443140	423127				

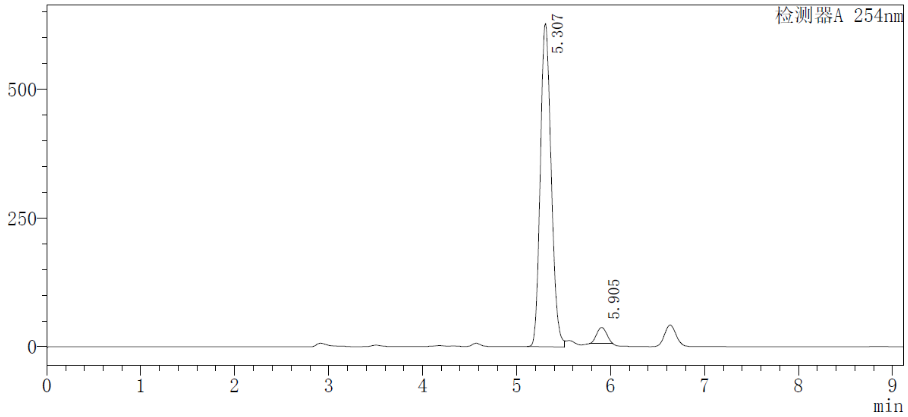

<峰表>
检测器A 254nm

峰号	保留时间	面积	高度	浓度	浓度单位	标记	化合物名
1	5.307	5038264	627628	95.556		M	
2	5.905	234322	31453	4.444		M	
总计		5272586	659081				

Supplementary Figure 78. HPLC data of **3p**.

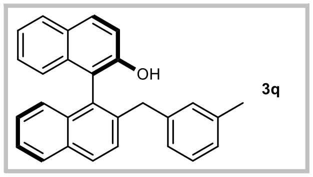

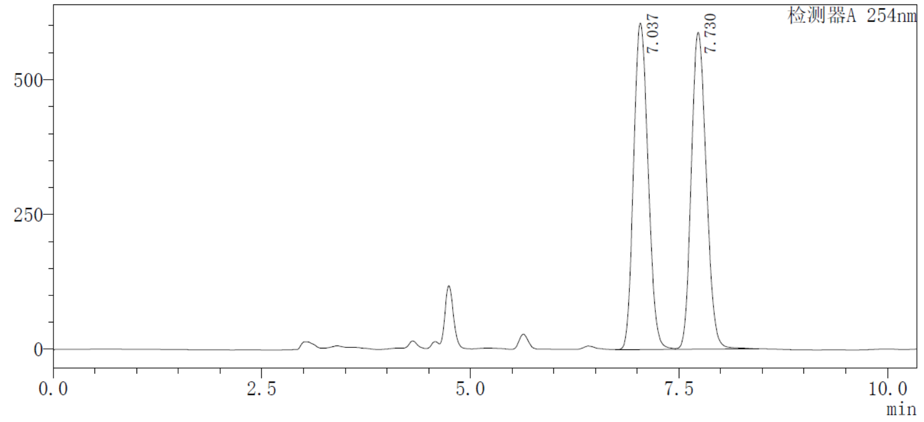

<峰表>
检测器A 254nm

峰号	保留时间	面积	高度	浓度	浓度单位	标记	化合物名
1	7.037	7329765	605149	49.910			
2	7.730	7356174	587562	50.090		V M	
总计		14685938	1192711				

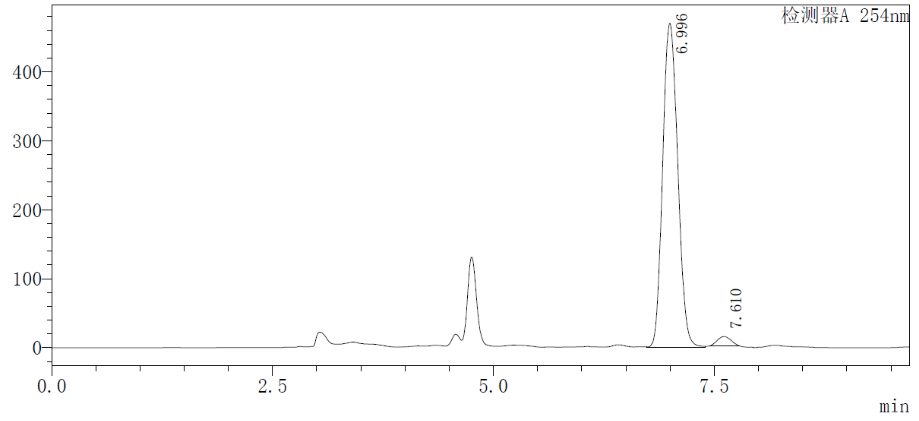

<峰表>
检测器A 254nm

峰号	保留时间	面积	高度	浓度	浓度单位	标记	化合物名
1	6.996	5604508	470263	97.497			
2	7.610	143884	13494	2.503		M	
总计		5748392	483757				

Supplementary Figure 79. HPLC data of **3q**.

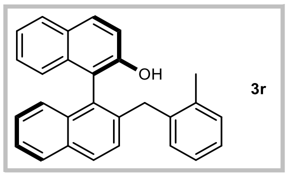

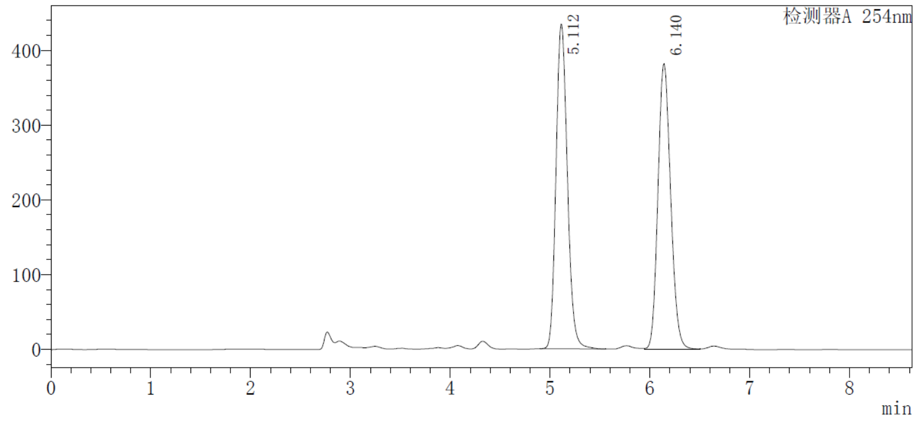

〈峰表〉
检测器A 254nm

峰号	保留时间	面积	高度	浓度	浓度单位	标记	化合物名
1	5.112	3431088	433989	50.167		M	
2	6.140	3408194	382611	49.833			
总计		6839282	816600				

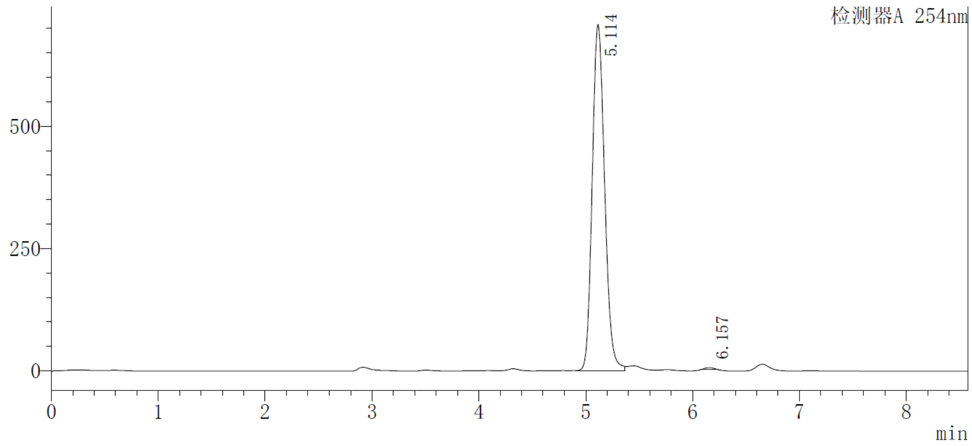

〈峰表〉
检测器A 254nm

峰号	保留时间	面积	高度	浓度	浓度单位	标记	化合物名
1	5.114	5705548	707450	99.539		M	
2	6.157	26420	4381	0.461		M	
总计		5731967	711831				

Supplementary Figure 80. HPLC data of **3r**.

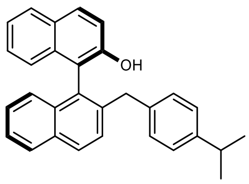

3s

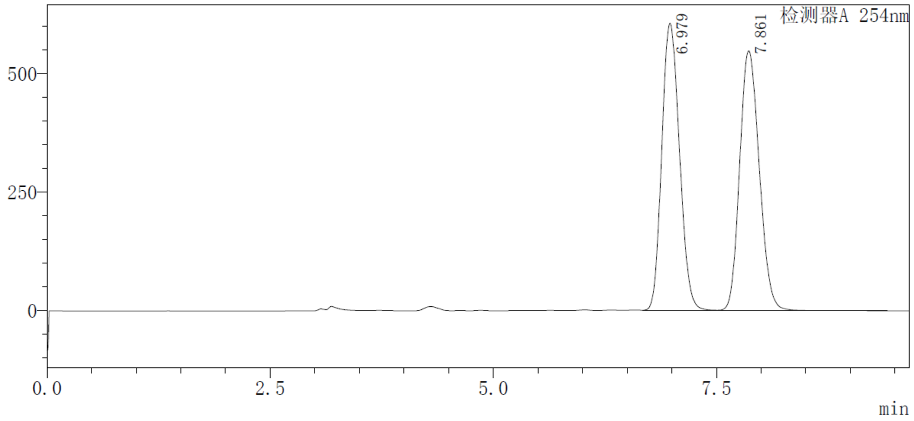

〈峰表〉
检测器A 254nm

峰号	保留时间	面积	高度	浓度	浓度单位	标记	化合物名
1	6.979	8409551	605863	49.948			
2	7.861	8427072	548127	50.052		SV	
总计		16836622	1153991				

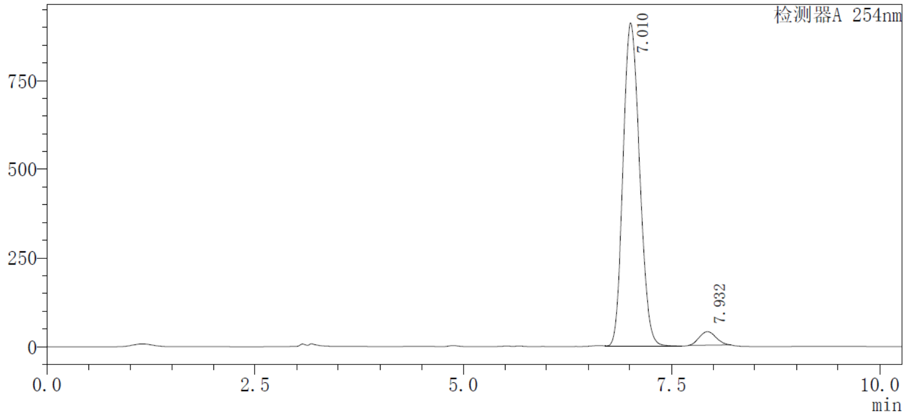

〈峰表〉
检测器A 254nm

峰号	保留时间	面积	高度	浓度	浓度单位	标记	化合物名
1	7.010	12667161	912562	95.942			
2	7.932	535820	38172	4.058		M	
总计		13202982	950734				

Supplementary Figure 81. HPLC data of **3s**.

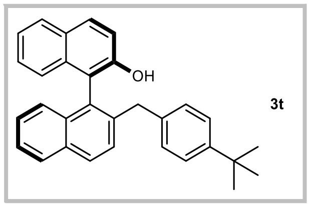

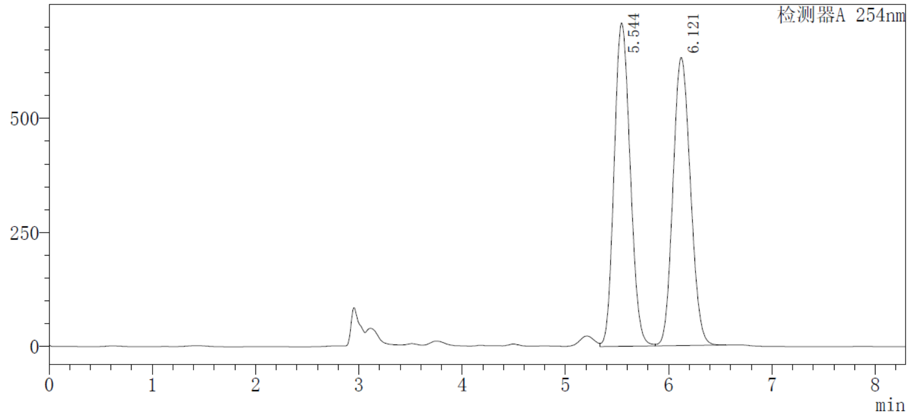

<峰表>
检测器A 254nm

峰号	保留时间	面积	高度	浓度	浓度单位	标记	化合物名
1	5.544	7685720	710139	50.740			
2	6.121	7461486	632353	49.260		V M	
总计		15147206	1342492				

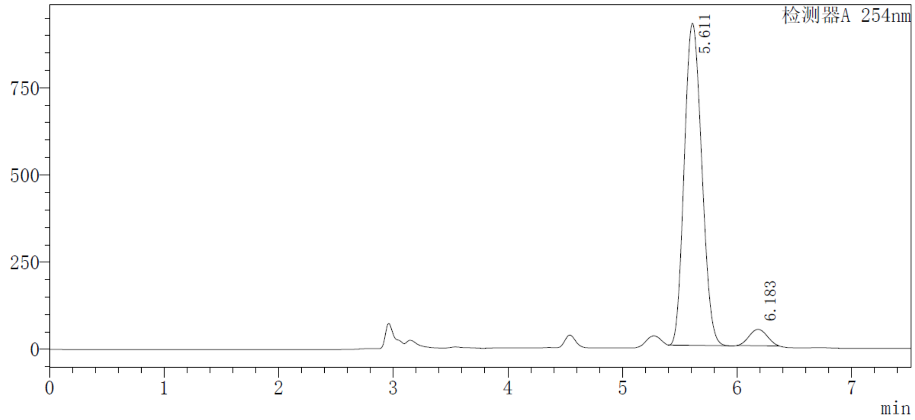

<峰表>
检测器A 254nm

峰号	保留时间	面积	高度	浓度	浓度单位	标记	化合物名
1	5.611	9801865	923605	95.019		M	
2	6.183	513864	47220	4.981		M	
总计		10315728	970825				

Supplementary Figure 82. HPLC data of **3t**.

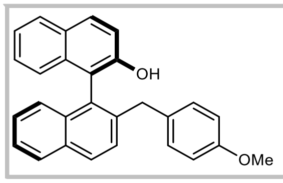

3u

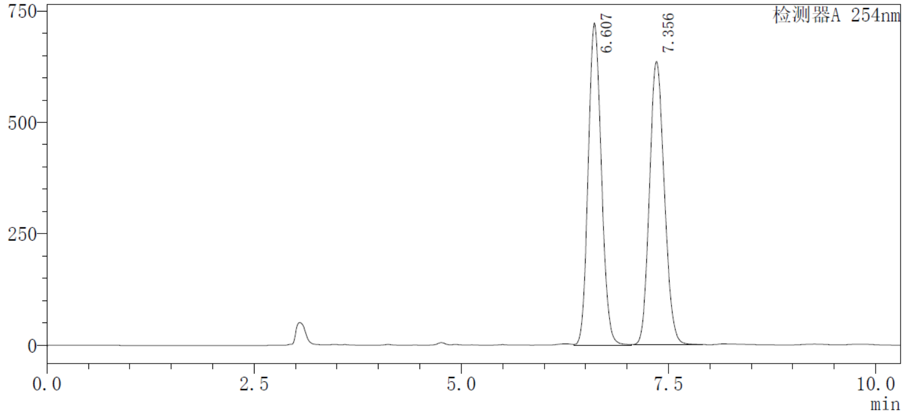

〈峰表〉
检测器A 254nm

峰号	保留时间	面积	高度	浓度	浓度单位	标记	化合物名
1	6.607	8003120	723663	50.242			
2	7.356	7926001	635163	49.758		M	
总计		15929121	1358826				

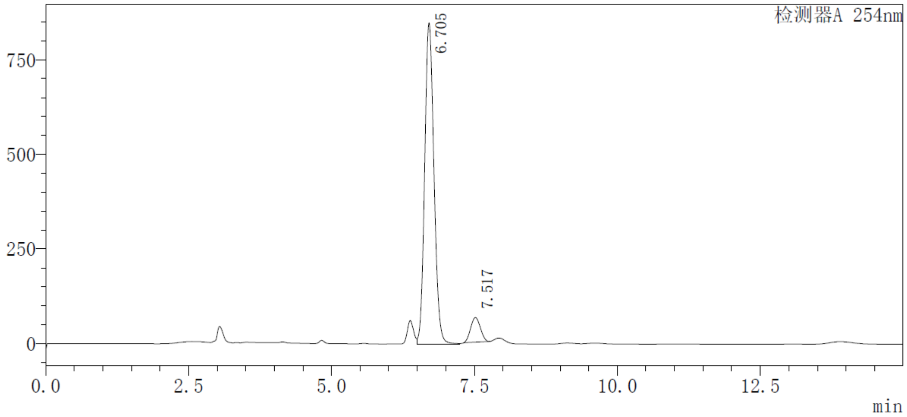

〈峰表〉
检测器A 254nm

峰号	保留时间	面积	高度	浓度	浓度单位	标记	化合物名
1	6.705	9581026	848416	92.532			
2	7.517	773260	65148	7.468		M	
总计		10354286	913564				

Supplementary Figure 83. HPLC data of **3u**.

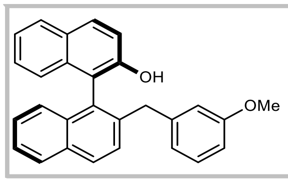

3v

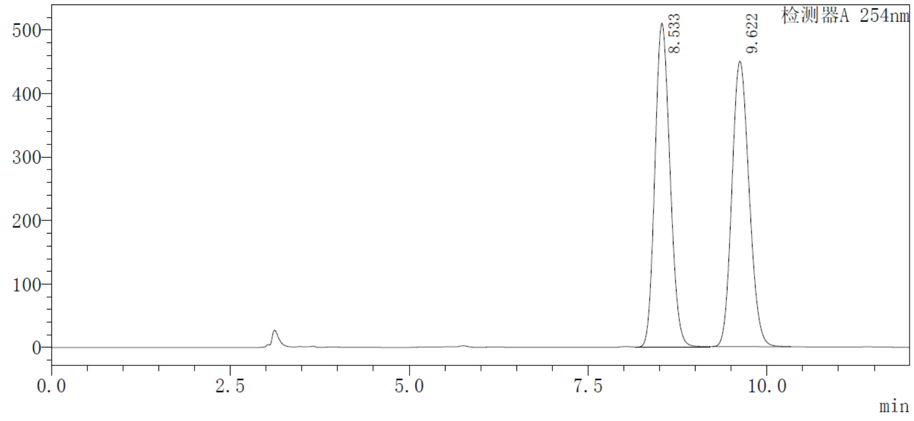

<峰表>
检测器A 254nm

峰号	保留时间	面积	高度	浓度	浓度单位	标记	化合物名
1	8.533	7526324	510364	50.092			
2	9.622	7498783	449464	49.908		M	
总计		15025107	959828				

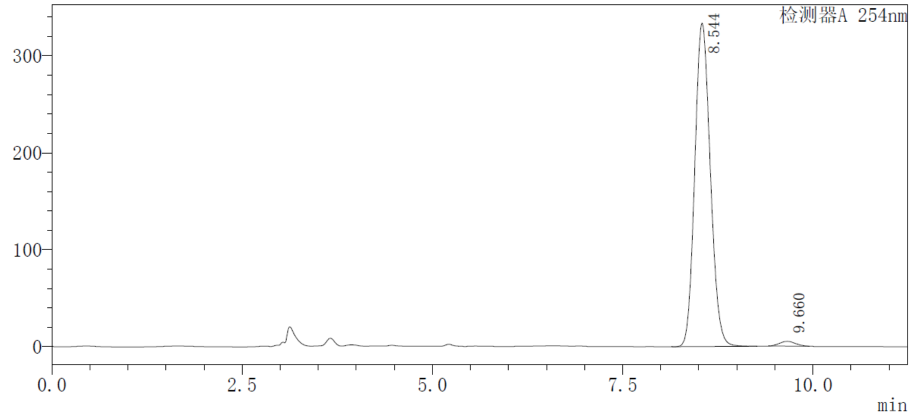

<峰表>
检测器A 254nm

峰号	保留时间	面积	高度	浓度	浓度单位	标记	化合物名
1	8.544	4750266	333518	98.477		M	
2	9.660	73454	4937	1.523		M	
总计		4823720	338455				

Supplementary Figure 84. HPLC data of **3v**.

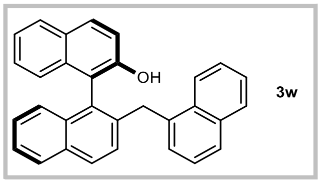

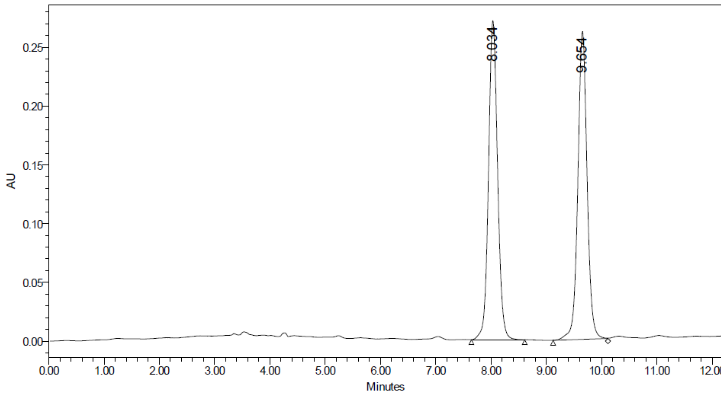

	RT (min)	Peak Type	Area (µV*sec)	% Area	Height (µV)	% Height	Integration Type	Points Across Peak	Start Time (min)	End Time (min)
1	8.034	Unknown	3097361	50.36	271370	50.90	BB	578	7.647	8.610
2	9.654	Unknown	3053660	49.64	261825	49.10	BV	596	9.123	10.117

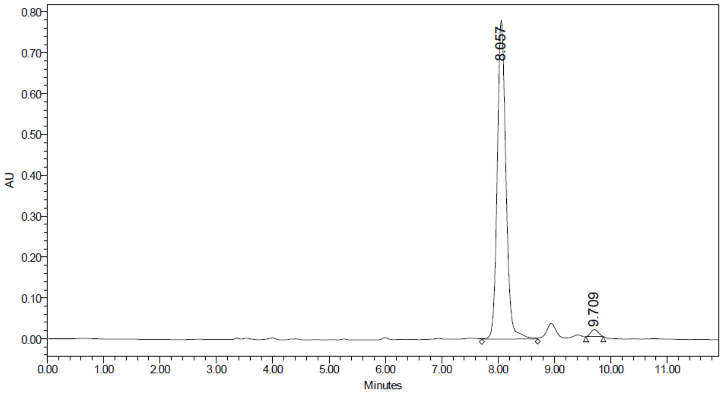

	RT (min)	Peak Type	Area (µV*sec)	% Area	Height (µV)	% Height	Integration Type	Points Across Peak	Start Time (min)	End Time (min)
1	8.057	Unknown	8364346	98.08	780069	97.84	VV	594	7.713	8.703
2	9.709	Unknown	163336	1.92	17220	2.16	bb	185	9.558	9.867

Supplementary Figure 85. HPLC data of **3w**.

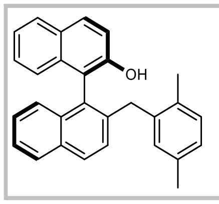

3x

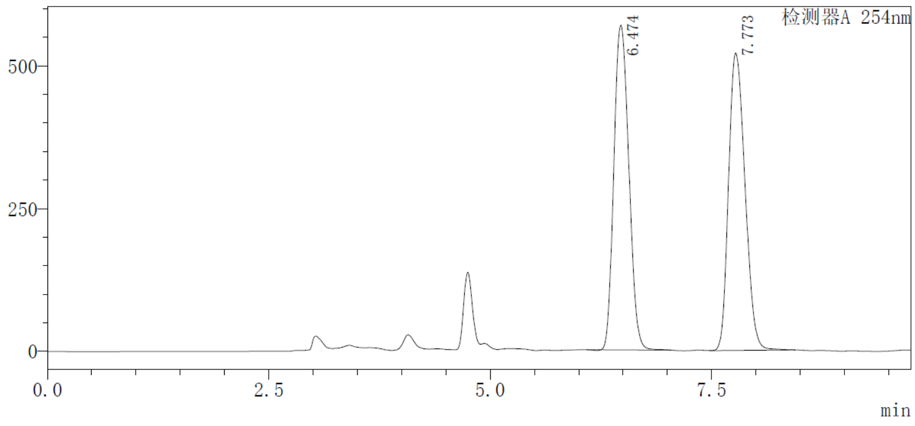

<峰表>
检测器A 254nm

峰号	保留时间	面积	高度	浓度	浓度单位	标记	化合物名
1	6.474	6691184	568652	49.859		M	
2	7.773	6729059	520838	50.141		M	
总计		13420243	1089490				

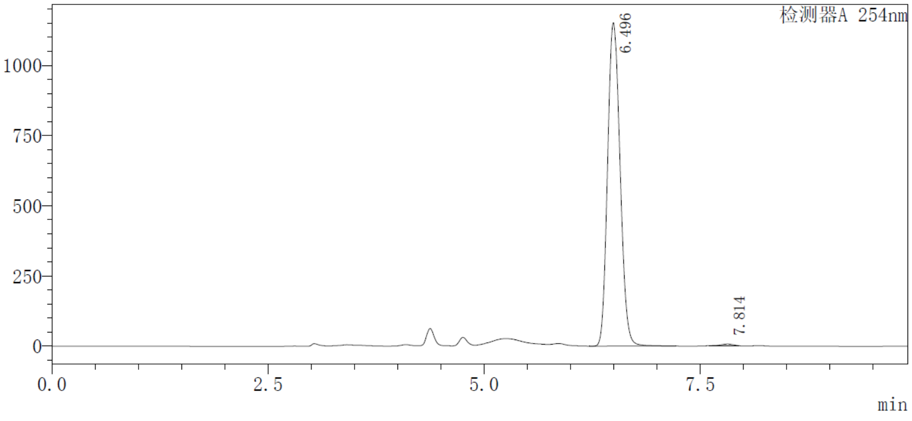

<峰表>
检测器A 254nm

峰号	保留时间	面积	高度	浓度	浓度单位	标记	化合物名
1	6.496	11564528	1150690	99.528		M	
2	7.814	54865	5608	0.472		M	
总计		11619393	1156298				

Supplementary Figure 86. HPLC data of **3x**.

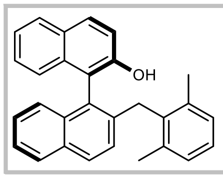

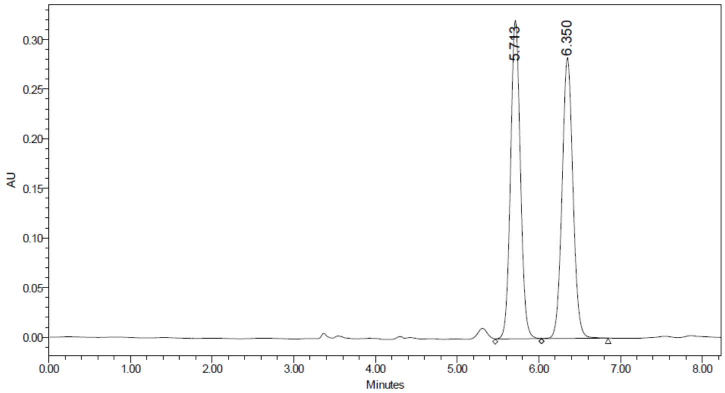

	RT (min)	Peak Type	Area (µV*sec)	% Area	Height (µV)	% Height	Integration Type	Points Across Peak	Start Time (min)	End Time (min)
1	5.713	Unknown	2669438	50.48	320986	53.18	VV	340	5.467	6.033
2	6.350	Unknown	2618318	49.52	282614	46.82	VB	489	6.033	6.848

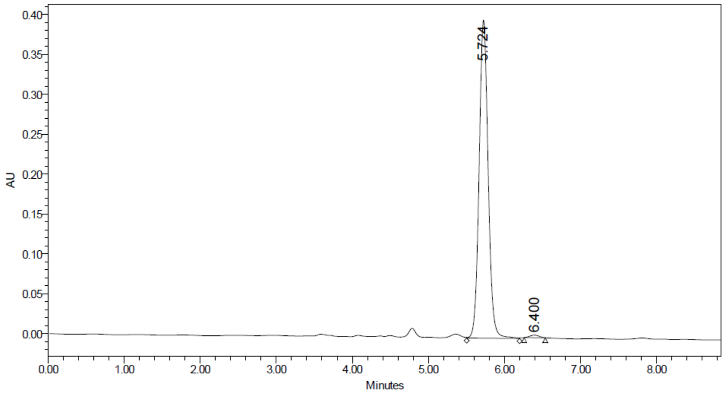

	RT (min)	Peak Type	Area (µV*sec)	% Area	Height (µV)	% Height	Integration Type	Points Across Peak	Start Time (min)	End Time (min)
1	5.724	Unknown	3286269	99.16	398723	99.20	VV	417	5.503	6.198
2	6.400	Unknown	27881	0.84	3224	0.80	bb	166	6.260	6.537

Supplementary Figure 87. HPLC data of **3y**.

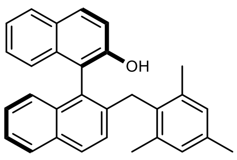

3z

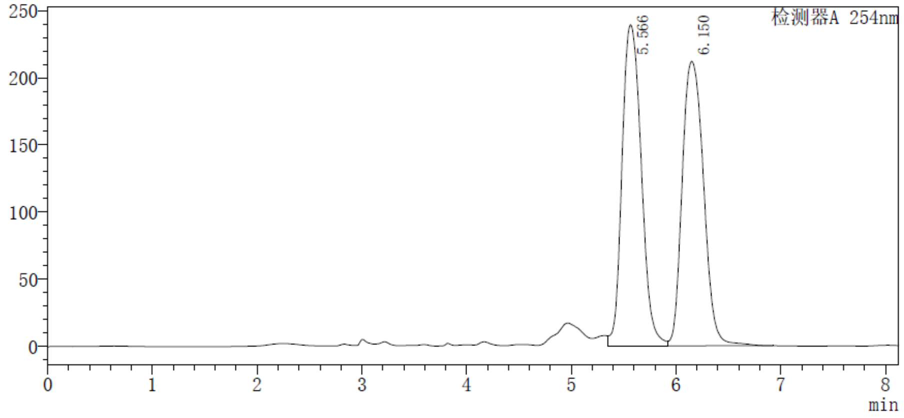

〈峰表〉

检测器A 254nm

峰号	保留时间	面积	高度	浓度	浓度单位	标记	化合物名
1	5.566	3050746	239192	50.302		M	
2	6.150	3014078	211876	49.698		V M	
总计		6064825	451069				

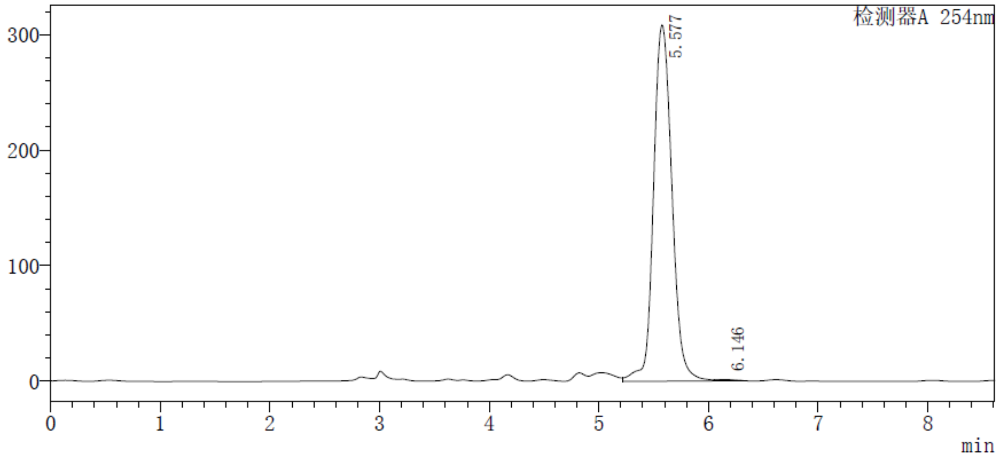

〈峰表〉

检测器A 254nm

峰号	保留时间	面积	高度	浓度	浓度单位	标记	化合物名
1	5.577	3612133	308649	99.878		S M	
2	6.146	4422	490	0.122		T	
总计		3616555	309139				

Supplementary Figure 88. HPLC data of **3z**.

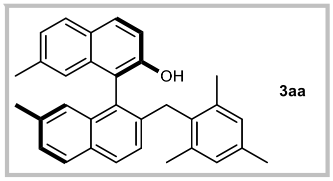

3aa

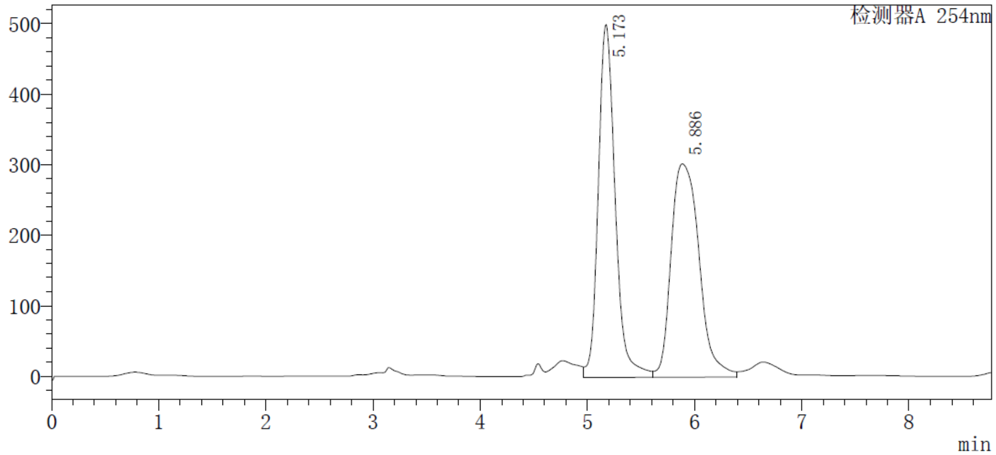

〈峰表〉
检测器A 254nm

峰号	保留时间	面积	高度	浓度	浓度单位	标记	化合物名
1	5.173	5497278	500557	49.917			
2	5.886	5515586	302745	50.083		V	
总计		11012864	803302				

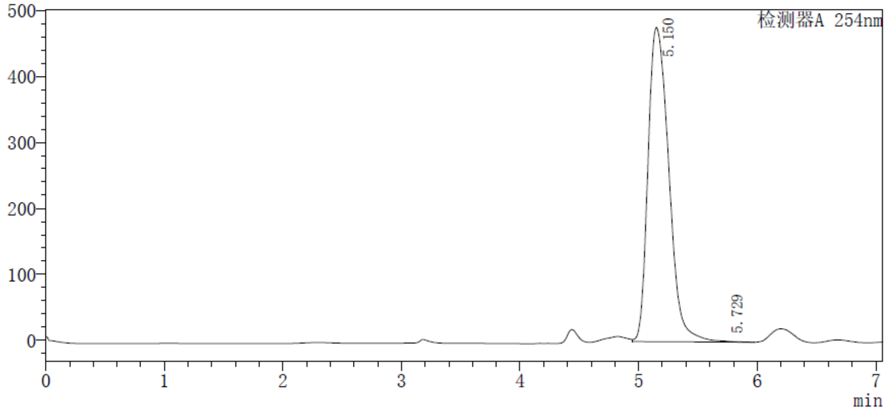

〈峰表〉
检测器A 254nm

峰号	保留时间	面积	高度	浓度	浓度单位	标记	化合物名
1	5.150	5819753	477104	99.862		M	
2	5.729	8058	1446	0.138		V M	
总计		5827811	478550				

Supplementary Figure 89. HPLC data of **3aa**.

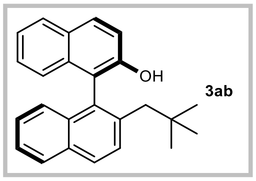

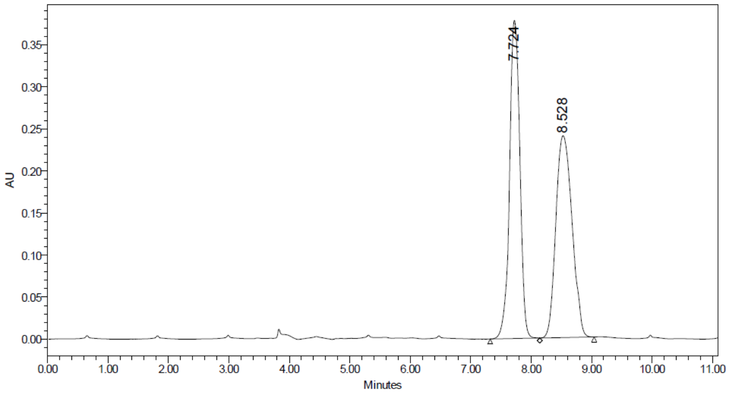

	RT (min)	Peak Type	Area (μV*sec)	% Area	Height (μV)	% Height	Integration Type	Points Across Peak	Start Time (min)	End Time (min)
1	7.724	Unknown	4470954	50.01	378154	61.18	BV	492	7.323	8.143
2	8.528	Unknown	4468899	49.99	239963	38.82	VB	540	8.143	9.043

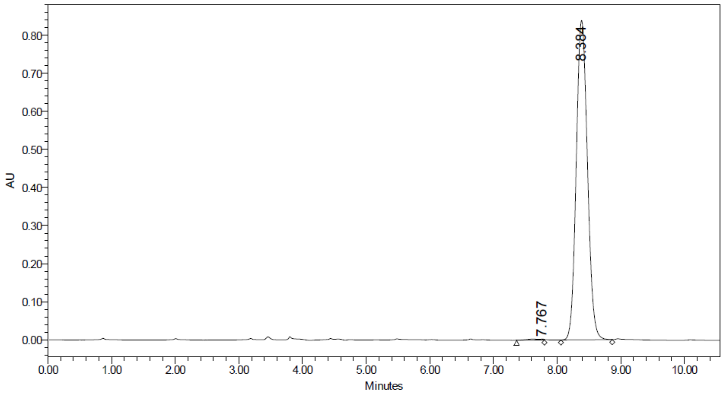

	RT (min)	Peak Type	Area (μV*sec)	% Area	Height (μV)	% Height	Integration Type	Points Across Peak	Start Time (min)	End Time (min)
1	7.767	Unknown	46786	0.45	2784	0.33	BV	263	7.363	7.802
2	8.384	Unknown	10249641	99.55	838867	99.67	VV	483	8.060	8.865

Supplementary Figure 90. HPLC data of **3ab**.

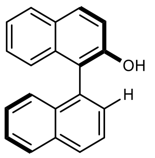

3ac

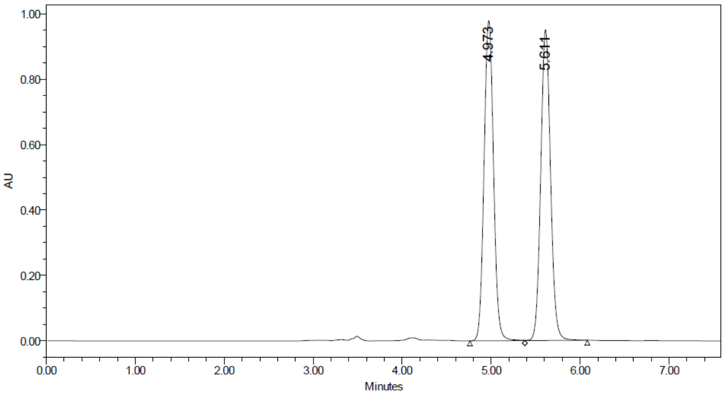

	RT (min)	Peak Type	Area (µV*sec)	% Area	Height (µV)	% Height	Integration Type	Points Across Peak	Start Time (min)	End Time (min)
1	4.973	Unknown	7243786	49.91	979767	50.74	BV	370	4.762	5.378
2	5.611	Unknown	7270493	50.09	951249	49.26	VB	422	5.378	6.082

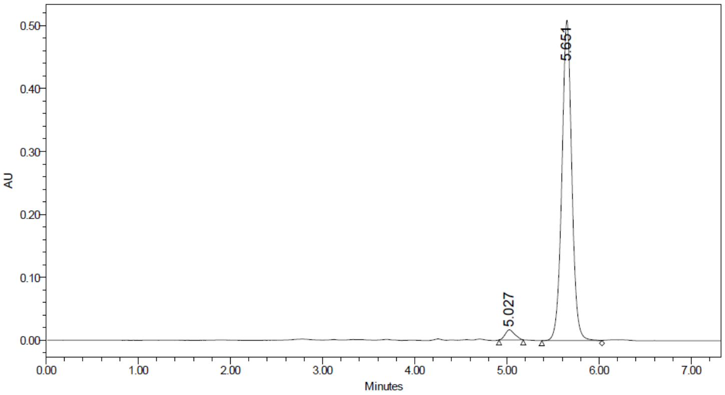

	RT (min)	Peak Type	Area (µV*sec)	% Area	Height (µV)	% Height	Integration Type	Points Across Peak	Start Time (min)	End Time (min)
1	5.027	Unknown	115124	2.93	15997	3.05	bb	158	4.915	5.178
2	5.651	Unknown	3815177	97.07	509300	96.95	bV	391	5.380	6.032

Supplementary Figure 91. HPLC data of **3ac**.

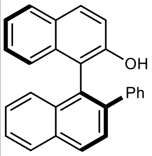

3ad

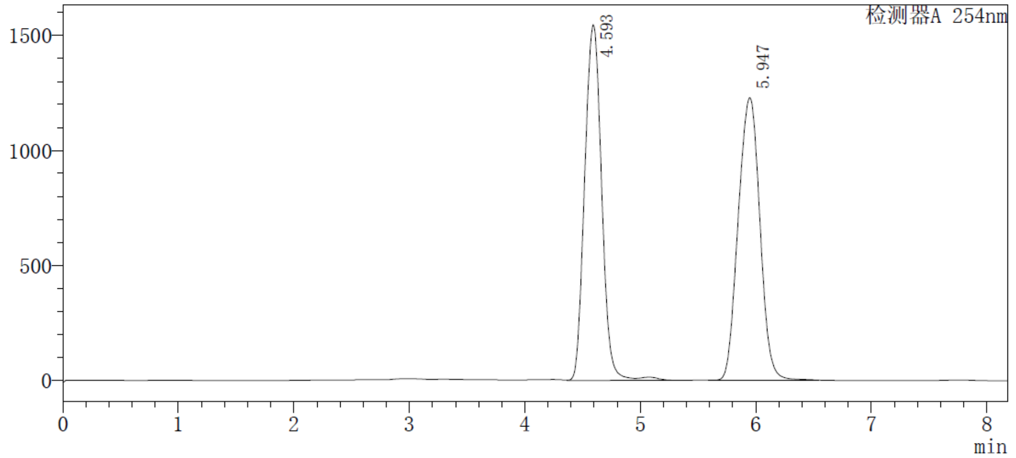

〈峰表〉
检测器A 254nm

峰号	保留时间	面积	高度	浓度	浓度单位	标记	化合物名
1	4.593	15909375	1549049	49.818		M	
2	5.947	16025343	1228689	50.182		M	
总计		31934717	2777737				

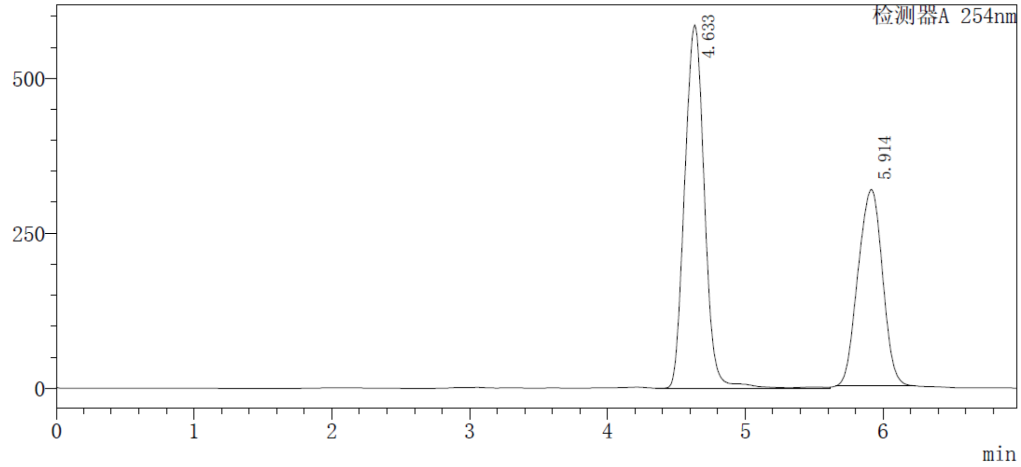

〈峰表〉
检测器A 254nm

峰号	保留时间	面积	高度	浓度	浓度单位	标记	化合物名
1	4.633	5850920	586397	59.833		M	
2	5.914	3927755	317068	40.167		M	
总计		9778674	903464				

Supplementary Figure 92. HPLC data of **3ad**.

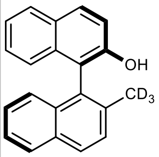

3ae

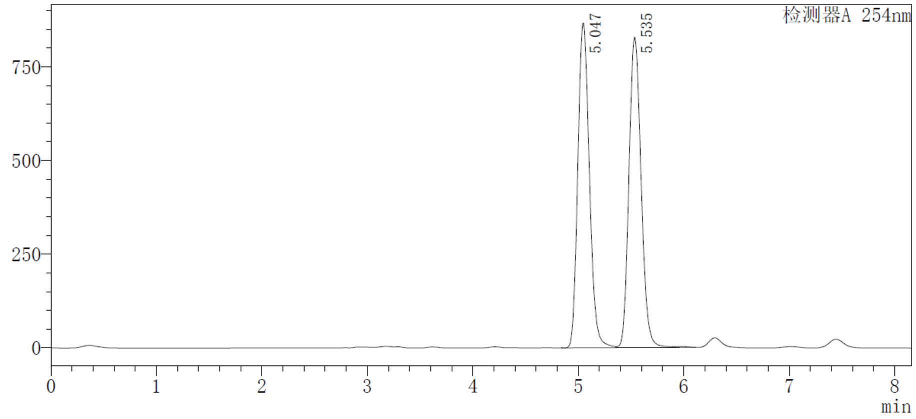

〈峰表〉
检测器A 254nm

峰号	保留时间	面积	高度	浓度	浓度单位	标记	化合物名
1	5.047	6633634	867578	49.946			
2	5.535	6648103	829652	50.054		V M	
总计		13281737	1697230				

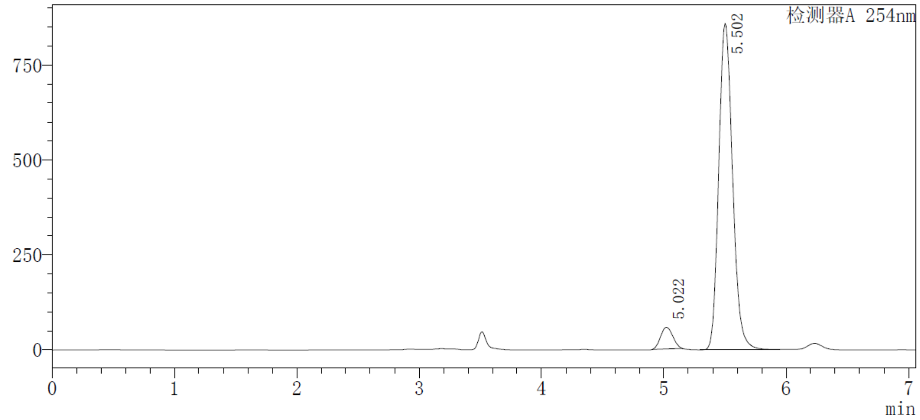

〈峰表〉
检测器A 254nm

峰号	保留时间	面积	高度	浓度	浓度单位	标记	化合物名
1	5.022	391360	56857	5.450		M	
2	5.502	6790090	860250	94.550			
总计		7181449	917107				

Supplementary Figure 93. HPLC data of **3ae**.

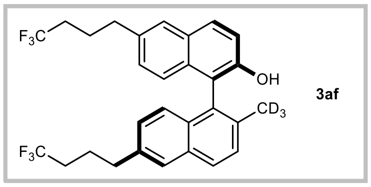

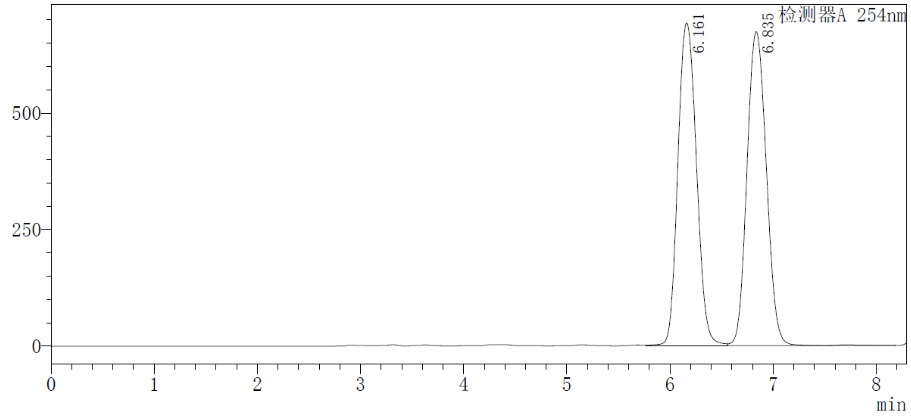

〈峰表〉
检测器A 254nm

峰号	保留时间	面积	高度	浓度	浓度单位	标记	化合物名
1	6. 161	8670833	692796	49. 630			
2	6. 835	8800002	673802	50. 370		V	
总计		17470836	1366598				

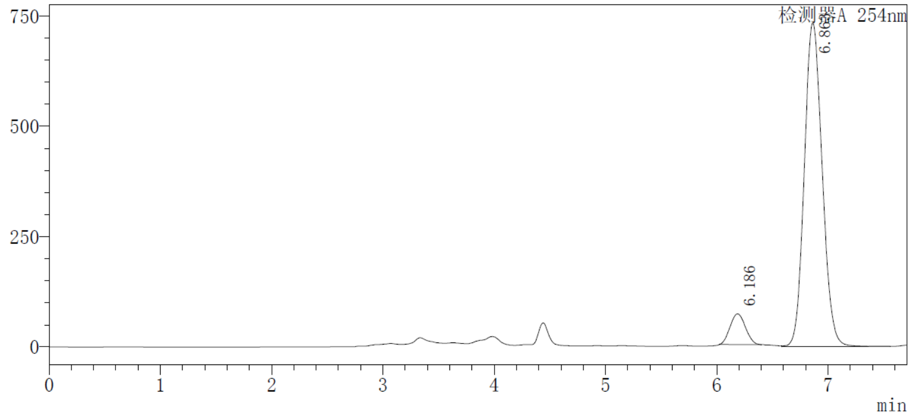

〈峰表〉
检测器A 254nm

峰号	保留时间	面积	高度	浓度	浓度单位	标记	化合物名
1	6. 186	672329	69838	7. 592		M	
2	6. 863	8183659	732716	92. 408			
总计		8855988	802554				

Supplementary Figure 94. HPLC data of **3af**.

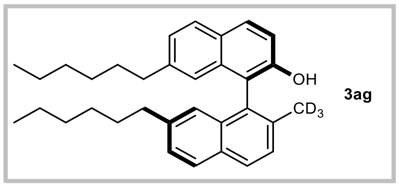

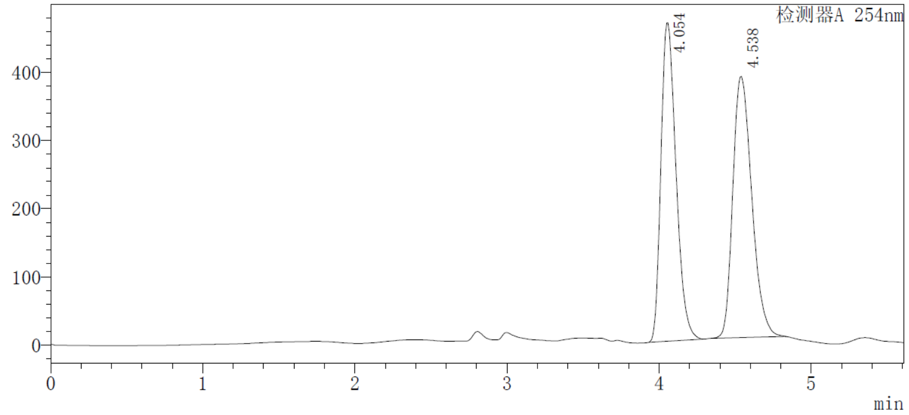

〈峰表〉
检测器A 254nm

峰号	保留时间	面积	高度	浓度	浓度单位	标记	化合物名
1	4.054	3213695	466969	49.179		M	
2	4.538	3321028	382841	50.821		M	
总计		6534723	849810				

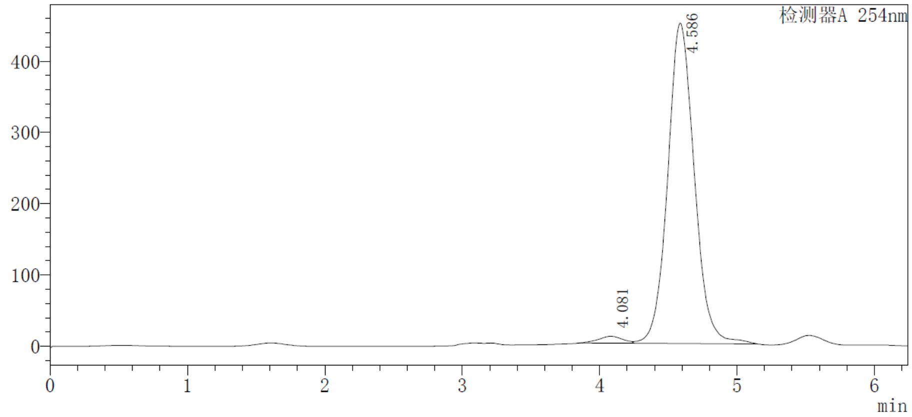

〈峰表〉
检测器A 254nm

峰号	保留时间	面积	高度	浓度	浓度单位	标记	化合物名
1	4.081	121440	9832	1.965		M	
2	4.586	6057456	449510	98.035		V M	
总计		6178896	459342				

Supplementary Figure 95. HPLC data of **3ag**.

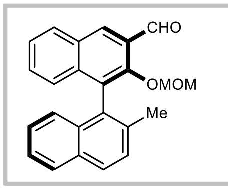

4

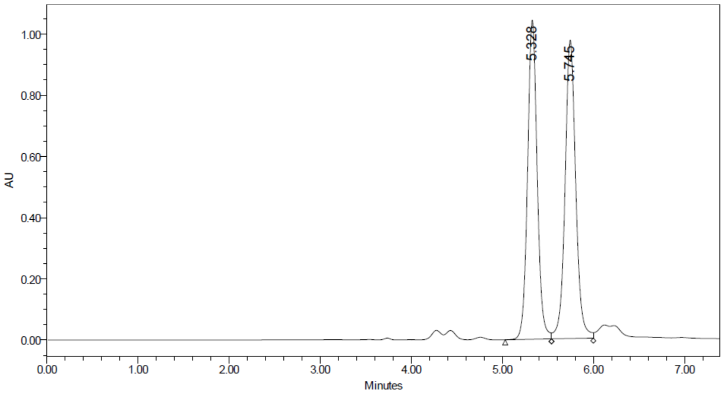

	RT (min)	Peak Type	Area (µV*sec)	% Area	Height (µV)	% Height	Integration Type	Points Across Peak	Start Time (min)	End Time (min)
1	5.328	Unknown	7640233	49.05	1042876	51.68	BV	305	5.032	5.540
2	5.745	Unknown	7937145	50.95	975003	48.32	VV	275	5.540	5.998

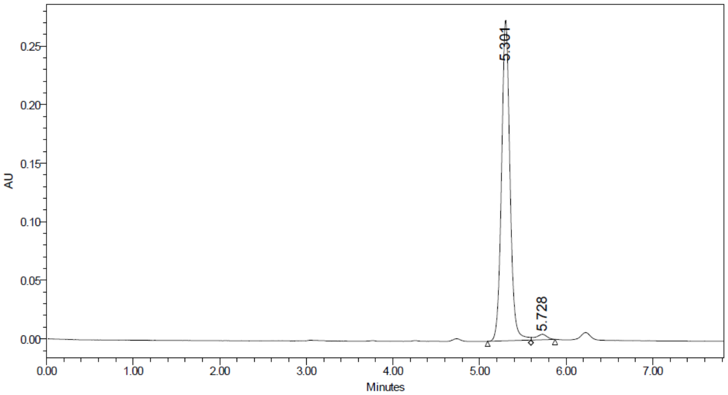

	RT (min)	Peak Type	Area (µV*sec)	% Area	Height (µV)	% Height	Integration Type	Points Across Peak	Start Time (min)	End Time (min)
1	5.301	Unknown	1854583	97.81	273791	98.33	Bv	300	5.093	5.593
2	5.728	Unknown	41600	2.19	4643	1.67	vb	167	5.593	5.872

Supplementary Figure 96. HPLC data of **4**.

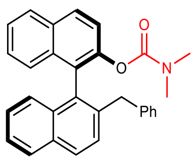

5

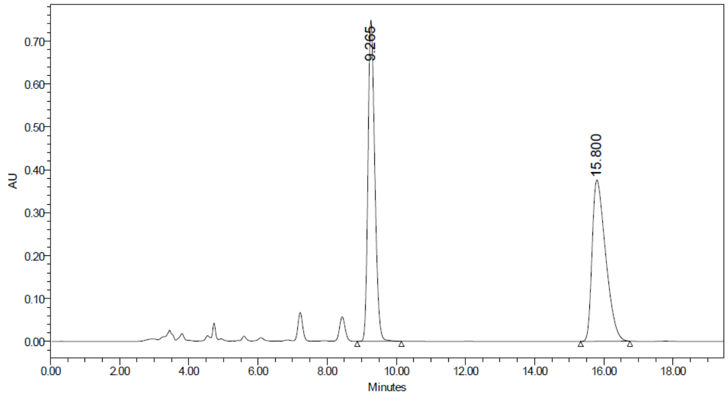

	RT (min)	Peak Type	Area (µV*sec)	% Area	Height (µV)	% Height	Integration Type	Points Across Peak	Start Time (min)	End Time (min)
1	9.265	Unknown	10218117	49.99	748135	66.57	BB	768	8.867	10.147
2	15.800	Unknown	10220169	50.01	375693	33.43	Bb	854	15.332	16.755

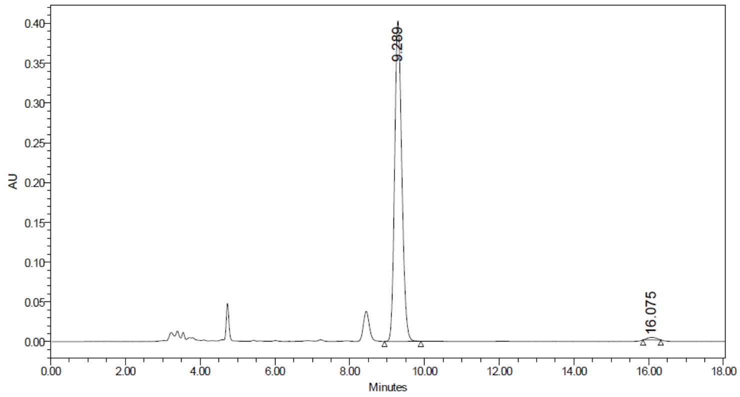

	RT (min)	Peak Type	Area (µV*sec)	% Area	Height (µV)	% Height	Integration Type	Points Across Peak	Start Time (min)	End Time (min)
1	9.289	Unknown	5352804	98.93	402680	99.18	BB	583	8.932	9.903
2	16.075	Unknown	57667	1.07	3343	0.82	bb	288	15.847	16.327

Supplementary Figure 97. HPLC data of **5**.

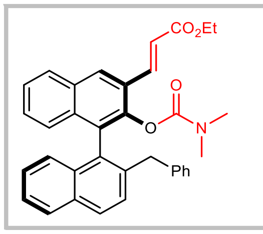

6

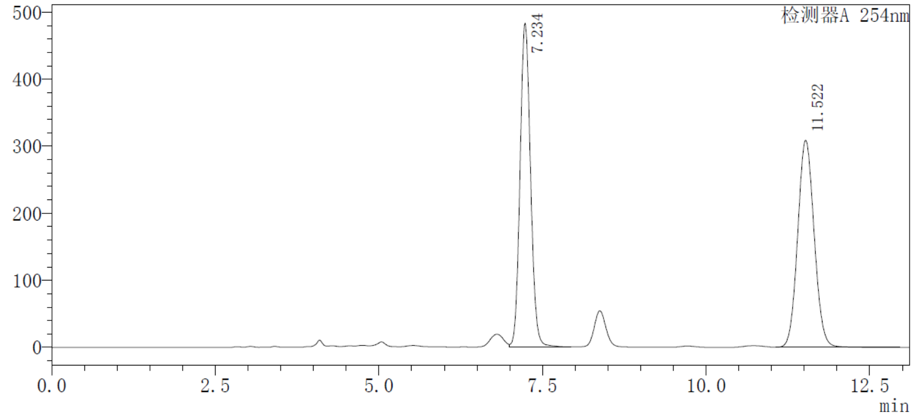

〈峰表〉
检测器A 254nm

峰号	保留时间	面积	高度	浓度	浓度单位	标记	化合物名
1	7.234	5420290	483684	50.027			
2	11.522	5414384	308963	49.973			
总计		10834673	792647				

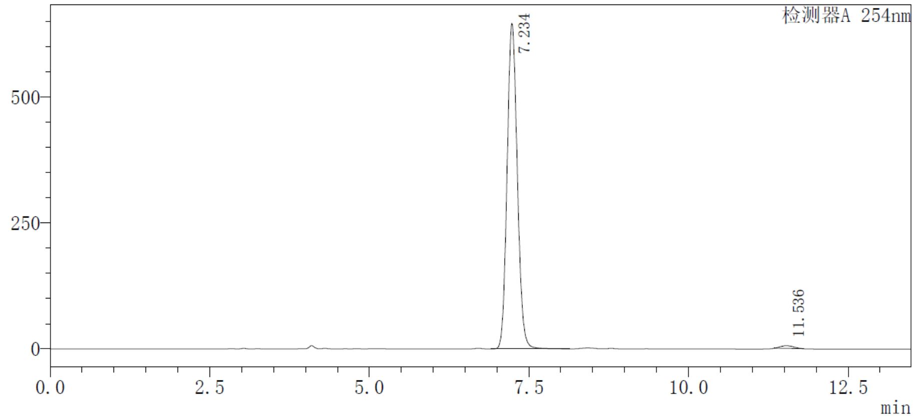

〈峰表〉
检测器A 254nm

峰号	保留时间	面积	高度	浓度	浓度单位	标记	化合物名
1	7.234	7198101	645966	98.925			
2	11.536	78245	5435	1.075		M	
总计		7276345	651401				

Supplementary Figure 98. HPLC data of **6**.

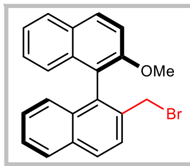

7

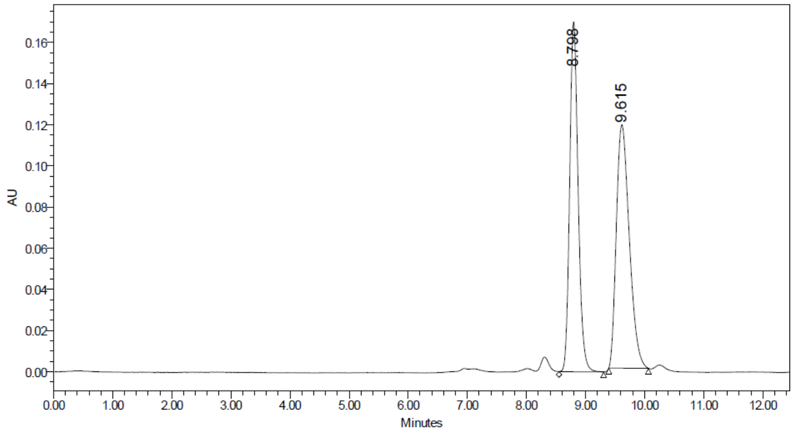

	RT (min)	Peak Type	Area (µV*sec)	% Area	Height (µV)	% Height	Integration Type	Points Across Peak	Start Time (min)	End Time (min)
1	8.798	Unknown	1689305	48.42	169963	59.00	VB	454	8.550	9.307
2	9.615	Unknown	1799769	51.58	118115	41.00	bb	402	9.393	10.063

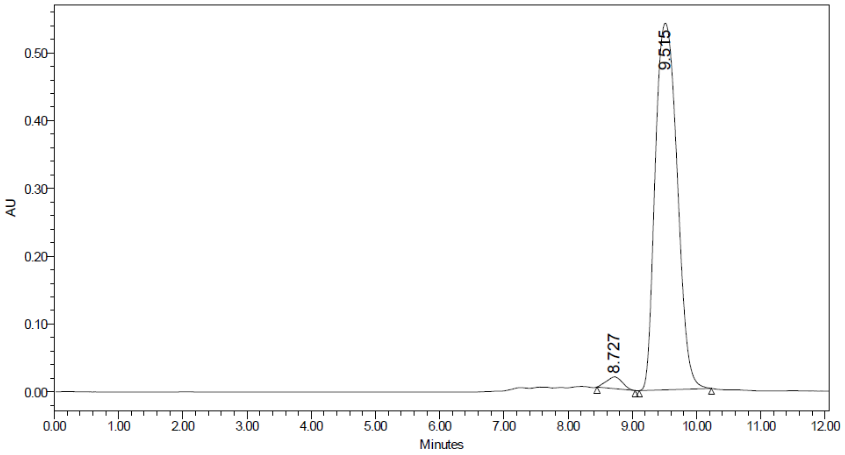

	RT (min)	Peak Type	Area (µV*sec)	% Area	Height (µV)	% Height	Integration Type	Points Across Peak	Start Time (min)	End Time (min)
1	8.727	Unknown	294952	2.24	17242	3.09	bb	356	8.450	9.043
2	9.515	Unknown	12850728	97.76	540986	96.91	bb	675	9.107	10.232

Supplementary Figure 99. HPLC data of **7**.

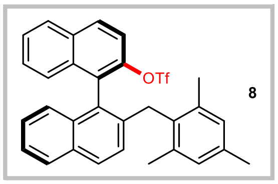

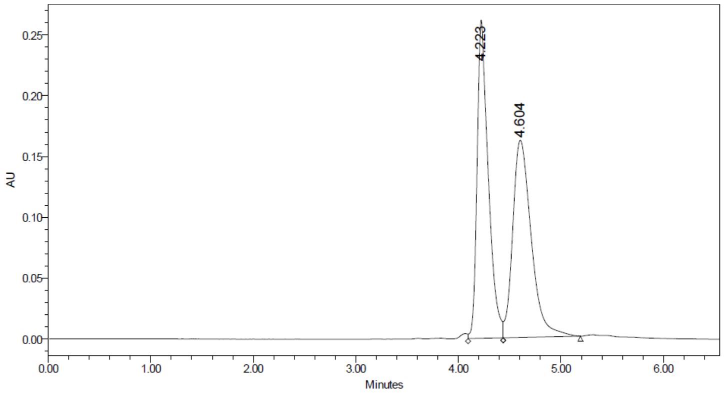

	RT (min)	Peak Type	Area (µV*sec)	% Area	Height (µV)	% Height	Integration Type	Points Across Peak	Start Time (min)	End Time (min)
1	4.223	Unknown	1978388	49.30	261740	61.73	VV	205	4.095	4.437
2	4.604	Unknown	2034849	50.70	162294	38.27	VB	452	4.437	5.190

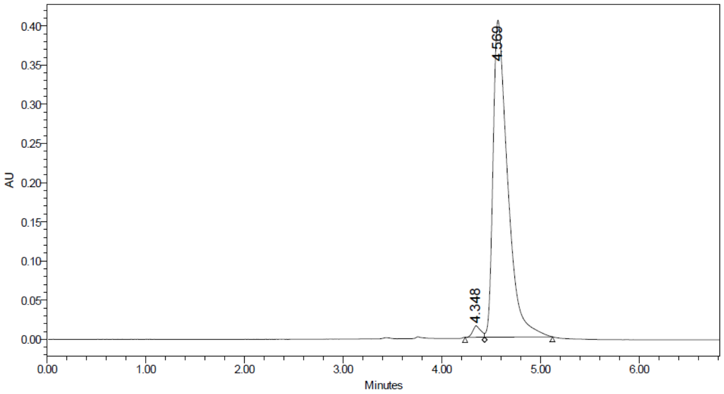

	RT (min)	Peak Type	Area (µV*sec)	% Area	Height (µV)	% Height	Integration Type	Points Across Peak	Start Time (min)	End Time (min)
1	4.348	Unknown	81299	1.92	14517	3.46	bV	118	4.237	4.433
2	4.569	Unknown	4160360	98.08	404466	96.54	Vb	413	4.433	5.122

Supplementary Figure 100. HPLC data of **8**.

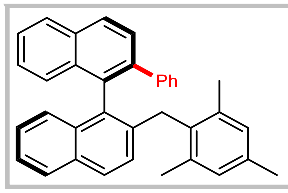

9

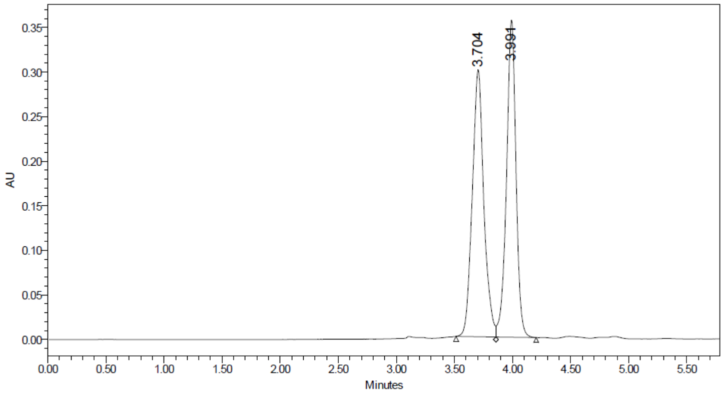

	RT (min)	Peak Type	Area (μV*sec)	% Area	Height (μV)	% Height	Integration Type	Points Across Peak	Start Time (min)	End Time (min)
1	3.704	Unknown	2068419	50.61	299422	45.71	bV	206	3.513	3.857
2	3.991	Unknown	2018883	49.39	355621	54.29	Vb	208	3.857	4.203

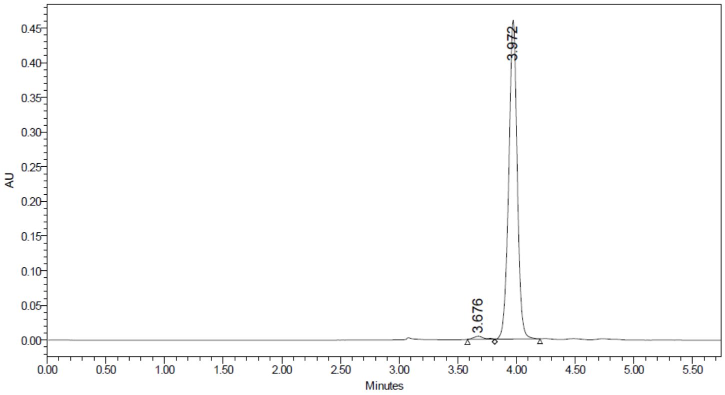

	RT (min)	Peak Type	Area (μV*sec)	% Area	Height (μV)	% Height	Integration Type	Points Across Peak	Start Time (min)	End Time (min)
1	3.676	Unknown	26190	1.13	4275	0.92	bV	139	3.583	3.815
2	3.972	Unknown	2296573	98.87	459646	99.08	VB	231	3.815	4.200

Supplementary Figure 101. HPLC data of **9**.

10. Supplementary References

1 Herrmann, W. A., Goossen, L. J., Köcher, C. & Artus, G. R. J. Chiral Heterocylic Carbenes in Asymmetric Homogeneous Catalysis. *Angew. Chem. Int. Ed.* **35**, 2805-2807 (1996).

2 Herrmann, W. A., Goossen, L. J., Artus, G. R. J. & Köcher, C. Metal Complexes of Chiral Imidazolin-2-ylidene Ligands. *Organometallics* **16**, 2472-2477 (1997).

3 Song, Y., Li, D., Yang, Y., Ji, H. & Liu, B. Synthesis and Cytotoxicity of Dinaphtho [2, 1-b: 1', 2'-d]furan Derivatives. *Chin. J. Org. Chem.* **38**, 1516-1524 (2018).

4 Yang, J. *et al.* Enantio- and Substrate-Selective Recognition of Chiral Neurotransmitters with C3-Symmetric Switchable Receptors. *Org. Lett.* **22**, 891-895 (2020).

5 Handa, S., Mathota Arachchige, Y. L. N. & Slaughter, L. M. Access to 2′-Substituted Binaphthyl Monoalcohols via Complementary Nickel-Catalyzed Kumada Coupling Reactions under Mild Conditions: Key Role of a P,O Ligand. *J. Org. Chem.* **78**, 5694-5699 (2013).

6 Frisch, M. J. *et al. Gaussian 16 Rev. B.01* (Wallingford, CT, 2016).

7 Becke, A. D. Density-functional thermochemistry. III. The role of exact exchange. *J. Chem. Phys.* **98**, 5648-5652 (1993).

8 Lee, C., Yang, W. & Parr, R. G. Development of the Colle-Salvetti correlation-energy formula into a functional of the electron density. *Phys. Rev. B* **37**, 785-789 (1988).

9 Hay, P. J. & Wadt, W. R. Ab initio effective core potentials for molecular calculations. Potentials for the transition metal atoms Sc to Hg. *J. Chem. Phys.* **82**, 270-283 (1985).

10 Wadt, W. R. & Hay, P. J. Ab initio effective core potentials for molecular calculations. Potentials for main group elements Na to Bi. *J. Chem. Phys.* **82**, 284-298 (1985).

11 Hay, P. J. & Wadt, W. R. Ab initio effective core potentials for molecular calculations. Potentials for K to Au including the outermost core orbitals. *J. Chem. Phys.* **82**, 299-310 (1985).

12 Zhao, Y. & Truhlar, D. G. The M06 suite of density functionals for main group thermochemistry, thermochemical kinetics, noncovalent interactions, excited states, and transition elements: two new functionals and systematic testing of four M06-class functionals and 12 other functionals. *Theor. Chem. Acc.* **120**, 215-241 (2008).

13 Dolg, M., Wedig, U., Stoll, H. & Preuss, H. Energy-adjusted ab initio pseudopotentials for the first row transition elements. *J. Chem. Phys.* **86**, 866-872 (1987).

14 Cossi, M., Rega, N., Scalmani, G. & Barone, V. Energies, structures, and electronic properties of molecules in solution with the C-PCM solvation model. *J. Comput. Chem.* **24**, 669-681 (2003).

15 Legault, C. J. U. d. S. CYLview, 1.0 b (http://www. cylview. org). (2009).

16 Ji, C.-L. & Hong, X. Factors Controlling the Reactivity and Chemoselectivity of Resonance Destabilized Amides in Ni-Catalyzed Decarbonylative and Nondecarbonylative Suzuki-Miyaura Coupling. *J. Am. Chem. Soc.* **139**, 15522-15529 (2017).

17 Zhang, Z.-B. *et al.* Nickel-Catalyzed Kumada Coupling of Boc-Activated Aromatic Amines via Nondirected Selective Aryl C–N Bond Cleavage. *Org. Lett.* **21**, 1226-1231 (2019).
