# Supplementary material for: Enantioselective alkylative cross-coupling of unactivated aromatic C–O electrophiles
Source: Nat Commun. 2022 May 26;13:2953. doi: 10.1038/s41467-022-30693-x (PMC9135759; doi:10.1038/s41467-022-30693-x)
Supplement: Supplementary file 3 — Description of Additional Supplementary file [file 41467_2022_30693_MOESM3_ESM.pdf]

Description of Additional Supplementary files

File name: Supplementary Data 1

Supplementary Data 1: Cartesian coordinates of the optimized structures.
