# Supplementary material for: Enantioselective alkylative cross-coupling of unactivated aromatic C–O electrophiles
Source: Nat Commun. 2022 May 26;13:2953. doi: 10.1038/s41467-022-30693-x (PMC9135759; doi:10.1038/s41467-022-30693-x)
Supplement: Supplementary file 4 — Supplementary Data 1 [file 41467_2022_30693_MOESM4_ESM.pdf]

Supplementary Data 1

Cartesian coordinates of optimized structures

Optimized Cartesian coordinates and their corresponding single-point energies ($E_{(SCRF)}$, a.u.) calculated at the M06/6-311+G(d,p)-SDD(CPCM, toluene)//B3LYP/6-31G(d)-LanL2DZ level as well as the imaginary frequency (IF, cm^{-1}) of transition states.

53 (the number of atoms involved in the molecule)

L1, $E_{(SCF\ Done)}$ = -1152.204988 a.u.

C	6.528683000	-2.484678000	-0.218311000
C	5.340270000	-1.923651000	-0.626186000
C	4.977434000	-0.609247000	-0.219213000
C	5.873793000	0.124381000	0.625680000
C	7.093653000	-0.484575000	1.029544000
C	7.414542000	-1.758131000	0.618292000
H	3.078663000	-0.563183000	-1.256265000
H	6.794325000	-3.489353000	-0.536130000
H	4.659189000	-2.478022000	-1.267548000
C	3.762238000	-0.001997000	-0.622228000
C	5.503841000	1.434074000	1.023761000
H	8.349779000	-2.213341000	0.933020000
C	4.313240000	1.994260000	0.612148000
C	3.417667000	1.276960000	-0.223694000
H	6.177937000	1.997958000	1.664621000
H	4.064960000	3.000655000	0.933446000
C	2.091625000	1.865970000	-0.714700000
H	2.083371000	1.816052000	-1.807904000
C	1.825864000	3.318694000	-0.304447000
H	0.873815000	3.646243000	-0.732297000
H	1.766971000	3.437716000	0.783213000
H	2.616206000	3.978304000	-0.677710000
C	0.180938000	0.375585000	-1.269020000
C	-0.504386000	-0.086867000	0.884365000
C	0.569736000	0.736428000	0.975402000
H	-1.125972000	-0.519555000	1.653946000
H	1.079716000	1.132026000	1.841029000
N	0.963164000	1.003917000	-0.333943000
N	-0.718085000	-0.292555000	-0.476792000
C	-7.619851000	-0.936813000	1.259977000
C	-6.393968000	-1.498479000	0.976284000

C	-5.394276000	-0.757377000	0.291424000
C	-5.680700000	0.588696000	-0.101941000
C	-6.953094000	1.139353000	0.204187000
C	-7.902419000	0.395008000	0.870212000
H	-3.926516000	-2.336534000	0.288139000
H	-8.375745000	-1.515294000	1.784300000
H	-6.175418000	-2.521539000	1.274274000
C	-4.118976000	-1.311131000	-0.014740000
C	-4.672791000	1.322358000	-0.787747000
H	-7.166694000	2.162389000	-0.097007000
H	-8.872719000	0.827662000	1.098707000
C	-3.452630000	0.757445000	-1.065514000
C	-3.156067000	-0.582162000	-0.679111000
H	-4.884895000	2.344847000	-1.092052000
H	-2.686419000	1.329042000	-1.582906000
C	-1.780627000	-1.137359000	-1.042581000
H	-1.643023000	-1.018724000	-2.122557000
C	-1.550225000	-2.610548000	-0.690560000
H	-0.548887000	-2.907554000	-1.015841000
H	-1.624549000	-2.792883000	0.387250000
H	-2.281589000	-3.248951000	-1.196838000
H	7.770872000	0.075317000	1.670559000

33

1a, $E_{\text{(SCF Done)}}$ = -844.1714946 a.u.

C	-2.713840000	2.615456000	0.453684000
C	-1.638026000	1.751430000	0.399626000
C	-1.799765000	0.383428000	0.059925000
C	-3.143594000	-0.098931000	-0.133690000
C	-4.222763000	0.822239000	-0.096795000
C	-4.018826000	2.157035000	0.176228000
H	-2.552429000	3.654757000	0.727118000
H	-0.657593000	2.117007000	0.672480000
C	-0.731303000	-0.578393000	-0.022060000
C	-3.403092000	-1.492738000	-0.303176000
H	-5.228249000	0.444629000	-0.267946000
H	-4.857924000	2.846342000	0.207603000
C	-2.392924000	-2.420689000	-0.243710000
C	-1.087339000	-1.929750000	-0.088004000
H	-4.432002000	-1.813716000	-0.441591000
H	-2.572208000	-3.487787000	-0.320627000
C	4.018750000	2.157102000	-0.176126000
C	4.222762000	0.822320000	0.096908000
C	3.143623000	-0.098906000	0.133698000

C	1.799829000	0.383421000	-0.059903000
C	1.638003000	1.751362000	-0.399743000
C	2.713770000	2.615455000	-0.453743000
H	4.432012000	-1.813732000	0.441579000
H	4.857804000	2.846473000	-0.207373000
H	5.228269000	0.444797000	0.268116000
C	3.403113000	-1.492758000	0.303092000
C	0.731388000	-0.578443000	0.022076000
H	0.657547000	2.116762000	-0.672749000
H	2.552375000	3.654727000	-0.727288000
C	1.087355000	-1.929808000	0.087992000
C	2.392980000	-2.420707000	0.243600000
H	2.572267000	-3.487818000	0.320427000
O	-0.000058000	-2.753898000	0.000123000

107

(L1)$_2$Ni, $E_{(SCF\ Done)}$ = -2475.467055 a.u.

C	-7.242095000	2.454587000	-0.139811000
C	-5.932869000	2.036763000	-0.064988000
C	-5.122785000	2.378415000	1.054242000
C	-5.695453000	3.164774000	2.107875000
C	-7.050534000	3.580801000	1.998462000
C	-7.806471000	3.235378000	0.901184000
H	-3.333001000	1.376340000	0.359300000
H	-7.850275000	2.185562000	-0.999147000
H	-5.499942000	1.434751000	-0.860059000
C	-3.771385000	1.963904000	1.164218000
C	-4.882294000	3.492875000	3.222796000
H	-7.480971000	4.177746000	2.799402000
H	-8.841330000	3.559159000	0.828437000
C	-3.572600000	3.069703000	3.298328000
C	-2.992954000	2.294595000	2.259192000
H	-5.310317000	4.086584000	4.027487000
H	-2.981695000	3.331625000	4.170535000
C	-1.544374000	1.810331000	2.300190000
H	-1.524704000	0.760962000	1.978551000
C	-0.856392000	1.902819000	3.666030000
H	0.137721000	1.450403000	3.598472000
H	-0.732624000	2.938413000	4.002024000
H	-1.433122000	1.363447000	4.425098000
C	-0.097099000	1.791838000	0.264037000
C	0.310429000	4.043194000	0.122568000
C	-0.498218000	3.847849000	1.194165000
H	0.741280000	4.948359000	-0.276763000

H	-0.928477000	4.554452000	1.887070000
N	-0.736391000	2.481526000	1.269004000
N	0.541512000	2.792004000	-0.434348000
C	6.913723000	5.392606000	-2.234575000
C	5.590302000	5.134143000	-2.518467000
C	4.872317000	4.138902000	-1.802954000
C	5.548273000	3.403668000	-0.777167000
C	6.912235000	3.692498000	-0.508609000
C	7.581565000	4.664809000	-1.219769000
H	3.014635000	4.415201000	-2.862639000
H	7.450654000	6.156995000	-2.789968000
H	5.076029000	5.691764000	-3.298164000
C	3.504830000	3.850805000	-2.074006000
C	4.821754000	2.406772000	-0.067650000
H	7.421653000	3.131402000	0.271577000
H	8.625437000	4.876697000	-1.004353000
C	3.501797000	2.156954000	-0.352498000
C	2.817906000	2.884269000	-1.370460000
H	5.330727000	1.837535000	0.707019000
H	2.954160000	1.393242000	0.194622000
C	1.358051000	2.529767000	-1.630748000
H	1.287195000	1.438120000	-1.736950000
C	0.727642000	3.175522000	-2.867510000
H	-0.297202000	2.809759000	-2.983298000
H	0.690722000	4.268279000	-2.793967000
H	1.293233000	2.913490000	-3.767786000
Ni	-0.053659000	-0.054003000	-0.051128000
C	-7.032405000	-2.307958000	0.142234000
C	-5.724378000	-1.905774000	-0.006193000
C	-4.925092000	-2.408493000	-1.070823000
C	-5.506308000	-3.341832000	-1.991272000
C	-6.859429000	-3.737501000	-1.808212000
C	-7.605374000	-3.233832000	-0.766535000
H	-3.127982000	-1.312499000	-0.551545000
H	-7.632594000	-1.914318000	0.958000000
H	-5.284706000	-1.193642000	0.687823000
C	-3.576182000	-2.012504000	-1.255139000
C	-4.703825000	-3.829843000	-3.054775000
H	-7.296765000	-4.446242000	-2.507945000
H	-8.639033000	-3.543470000	-0.636883000
C	-3.396558000	-3.420411000	-3.205980000
C	-2.809510000	-2.499596000	-2.297996000
H	-5.139190000	-4.535404000	-3.759005000

H	-2.814199000	-3.805525000	-4.037682000
C	-1.361479000	-2.027653000	-2.418885000
H	-1.331540000	-0.961810000	-2.160399000
C	-0.724798000	-2.204934000	-3.800890000
H	0.270162000	-1.748939000	-3.803511000
H	-0.610716000	-3.258418000	-4.079527000
H	-1.334725000	-1.715581000	-4.567701000
C	0.047025000	-1.900179000	-0.360868000
C	0.585917000	-4.120524000	-0.181128000
C	-0.204204000	-3.985775000	-1.275764000
H	1.061851000	-4.993322000	0.238554000
H	-0.569772000	-4.725477000	-1.971156000
N	-0.518560000	-2.636852000	-1.376277000
N	0.726588000	-2.851842000	0.365770000
C	7.202682000	-5.027993000	2.354544000
C	5.859063000	-4.849596000	2.602941000
C	5.098268000	-3.910590000	1.856325000
C	5.752160000	-3.147516000	0.836595000
C	7.137748000	-3.353481000	0.604920000
C	7.848684000	-4.272670000	1.345732000
H	3.235691000	-4.289456000	2.874992000
H	7.772414000	-5.749986000	2.933488000
H	5.361459000	-5.428461000	3.377955000
C	3.709181000	-3.705452000	2.090463000
C	4.982550000	-2.207432000	0.095668000
H	7.630309000	-2.771505000	-0.170780000
H	8.908781000	-4.421273000	1.158432000
C	3.642908000	-2.037322000	0.345280000
C	2.980837000	-2.792956000	1.357029000
H	5.473962000	-1.617617000	-0.674929000
H	3.062526000	-1.316661000	-0.225880000
C	1.495815000	-2.527208000	1.578033000
H	1.354863000	-1.441003000	1.669588000
C	0.876390000	-3.198131000	2.806914000
H	-0.172039000	-2.896820000	2.892990000
H	0.910339000	-4.291746000	2.745988000
H	1.401608000	-2.891213000	3.717416000

140

TS1, $E_{(\text{SCF Done})}$ = -3319.621085 a.u., IF = -360.25 cm^{-1}

Ni	-0.096067000	0.098623000	-0.764314000
O	0.584642000	-0.196313000	-2.640491000
C	-0.523728000	-1.325881000	-1.999541000
C	5.438635000	-1.278008000	3.191269000

C	6.640075000	-1.938883000	3.566160000
C	7.181667000	-2.917807000	2.763931000
H	3.116526000	-1.274044000	0.654024000
H	6.981896000	-4.054362000	0.923024000
H	4.897347000	-2.940118000	0.226673000
C	3.598921000	-0.984352000	1.585927000
C	4.845120000	-0.262419000	3.984384000
H	7.123760000	-1.657984000	4.499061000
H	8.100284000	-3.417606000	3.059982000
C	3.679615000	0.358188000	3.590190000
C	3.032852000	0.000805000	2.375349000
H	5.326301000	0.024934000	4.916697000
H	3.259737000	1.137455000	4.218124000
C	1.733095000	0.666658000	1.921375000
H	1.792774000	0.837726000	0.843797000
C	1.429261000	2.005154000	2.603833000
H	0.549003000	2.460049000	2.144680000
H	1.230194000	1.885682000	3.674403000
H	2.272080000	2.693424000	2.485166000
C	-0.239912000	-0.645448000	1.033359000
C	-0.814226000	-1.662526000	3.001885000
C	0.252817000	-0.872317000	3.260601000
H	-1.390643000	-2.298491000	3.653852000
H	0.809090000	-0.713082000	4.171023000
N	0.592551000	-0.261264000	2.061894000
N	-1.102740000	-1.520941000	1.649469000
C	-7.090631000	-2.982014000	4.753567000
C	-5.841015000	-3.268473000	4.250665000
C	-5.312566000	-2.537286000	3.152195000
C	-6.105200000	-1.495175000	2.571404000
C	-7.391207000	-1.225323000	3.111682000
C	-7.873669000	-1.950189000	4.178707000
H	-3.452598000	-3.619063000	3.056836000
H	-7.482575000	-3.548897000	5.593712000
H	-5.239952000	-4.061875000	4.689187000
C	-4.026099000	-2.809561000	2.611947000
C	-5.573878000	-0.779142000	1.465127000
H	-7.990860000	-0.435598000	2.664917000
H	-8.859055000	-1.735108000	4.583166000
C	-4.323589000	-1.070890000	0.970488000
C	-3.516500000	-2.091299000	1.547980000
H	-6.174872000	0.000025000	1.002025000
H	-3.947578000	-0.532511000	0.105276000

C	-2.128245000	-2.342497000	0.963302000
H	-2.126471000	-1.983165000	-0.063795000
C	-1.700516000	-3.815678000	0.953507000
H	-0.737142000	-3.909751000	0.446081000
H	-1.599899000	-4.227017000	1.963838000
H	-2.435079000	-4.413817000	0.406817000
C	6.546429000	-3.280537000	1.549136000
C	6.963039000	3.784733000	1.813198000
C	5.901964000	3.098762000	1.265560000
C	5.026327000	3.736848000	0.344552000
C	5.265200000	5.104818000	-0.006985000
C	6.369680000	5.782918000	0.575239000
C	7.199668000	5.138273000	1.465079000
H	3.763178000	2.016948000	0.022882000
H	7.625875000	3.287832000	2.516289000
H	5.717591000	2.061578000	1.533830000
C	3.921947000	3.060597000	-0.239363000
C	4.388724000	5.726385000	-0.935620000
H	6.549429000	6.820926000	0.305031000
H	8.041943000	5.666025000	1.904304000
C	3.328124000	5.039755000	-1.481791000
C	3.068293000	3.685511000	-1.127948000
H	4.573419000	6.759878000	-1.219682000
H	2.692302000	5.539391000	-2.207326000
C	1.890808000	2.912327000	-1.711375000
H	2.081801000	1.846544000	-1.574194000
C	1.648546000	3.164417000	-3.206695000
H	0.896023000	2.461698000	-3.574928000
H	1.296565000	4.183935000	-3.397477000
H	2.574844000	3.014285000	-3.771202000
C	-0.218392000	2.152974000	-0.583845000
C	-1.090463000	4.212132000	-0.097410000
C	0.123442000	4.412814000	-0.666772000
H	-1.826258000	4.915090000	0.261254000
H	0.659811000	5.325806000	-0.869129000
N	0.639437000	3.154870000	-0.954728000
N	-1.282475000	2.838051000	-0.054551000
C	-8.296256000	4.289156000	-0.760087000
C	-7.220005000	3.931556000	0.022337000
C	-6.013699000	3.468074000	-0.568103000
C	-5.932479000	3.379346000	-1.994294000
C	-7.058510000	3.754845000	-2.773511000
C	-8.215173000	4.199695000	-2.171325000

H	-4.974295000	3.164516000	1.294491000
H	-9.213381000	4.641813000	-0.296080000
H	-7.280464000	4.000113000	1.106225000
C	-4.886035000	3.091345000	0.214721000
C	-4.718746000	2.917406000	-2.573866000
H	-6.992594000	3.684534000	-3.856694000
H	-9.070885000	4.483633000	-2.777752000
C	-3.648558000	2.563209000	-1.788620000
C	-3.717857000	2.642319000	-0.366342000
H	-4.647355000	2.847487000	-3.656737000
H	-2.726588000	2.222896000	-2.251761000
C	-2.507601000	2.189103000	0.448637000
H	-2.330352000	1.127995000	0.245044000
C	-2.639440000	2.359363000	1.964662000
H	-1.734241000	1.990955000	2.453626000
H	-2.788337000	3.406336000	2.252120000
H	-3.483814000	1.772551000	2.338065000
C	-0.933264000	-6.047680000	-3.327520000
C	-0.118986000	-4.990968000	-2.969650000
C	-0.649113000	-3.713604000	-2.624380000
C	-2.074681000	-3.548747000	-2.787611000
C	-2.881713000	-4.656864000	-3.138257000
C	-2.335038000	-5.899939000	-3.386889000
H	-0.480817000	-7.005401000	-3.574755000
H	0.952185000	-5.139352000	-2.971169000
C	0.130806000	-2.580664000	-2.209385000
C	-2.645800000	-2.239837000	-2.709501000
H	-3.954939000	-4.500675000	-3.233384000
H	-2.967913000	-6.741662000	-3.654320000
C	-1.884775000	-1.141198000	-2.414468000
C	4.797017000	-1.645376000	1.962944000
H	-3.705533000	-2.126478000	-2.931614000
H	-2.318641000	-0.144792000	-2.424571000
C	5.114569000	-4.673099000	-2.066453000
C	5.150458000	-3.408929000	-2.615255000
C	3.988110000	-2.593519000	-2.673686000
C	2.734352000	-3.126788000	-2.205804000
C	2.753701000	-4.404002000	-1.580770000
C	3.906625000	-5.160589000	-1.515562000
H	5.002181000	-0.876753000	-3.523115000
H	6.013840000	-5.282761000	-2.032622000
H	6.083597000	-3.005122000	-3.002622000
C	4.051849000	-1.256499000	-3.156204000

C	1.555024000	-2.315367000	-2.320057000
H	1.842487000	-4.775004000	-1.124657000
H	3.887479000	-6.131686000	-1.027746000
C	1.717449000	-0.955051000	-2.659581000
C	2.943217000	-0.438592000	-3.127635000
H	2.992053000	0.585343000	-3.483811000
C	5.382284000	-2.659999000	1.156557000

140

A, $E_{\text{(SCF Done)}}$ = -3319.664149 a.u.

Ni	-0.051428000	0.101899000	-0.863838000
O	1.192697000	0.248583000	-2.329200000
C	-0.648027000	-1.482387000	-1.748684000
C	5.090503000	-1.548675000	3.276068000
C	6.185595000	-2.335213000	3.726375000
C	6.535062000	-3.492081000	3.067116000
H	2.715054000	-1.523680000	0.789922000
H	6.091185000	-4.827954000	1.411351000
H	4.184998000	-3.501875000	0.590480000
C	3.261603000	-1.192151000	1.670910000
C	4.692152000	-0.351410000	3.923607000
H	6.741895000	-2.007376000	4.601721000
H	7.373971000	-4.086707000	3.419333000
C	3.617887000	0.381775000	3.466925000
C	2.878199000	-0.035561000	2.326669000
H	5.247132000	-0.015973000	4.796984000
H	3.344653000	1.293481000	3.987802000
C	1.662909000	0.744134000	1.818167000
H	1.755278000	0.867081000	0.737577000
C	1.472399000	2.125133000	2.454663000
H	0.625673000	2.633852000	1.989263000
H	1.277678000	2.054658000	3.530321000
H	2.363536000	2.741309000	2.304978000
C	-0.450693000	-0.445716000	1.010423000
C	-1.035443000	-1.317753000	3.041726000
C	0.088502000	-0.592959000	3.229727000
H	-1.639967000	-1.877813000	3.736015000
H	0.682488000	-0.423882000	4.113591000
N	0.434622000	-0.068990000	1.993690000
N	-1.355985000	-1.224247000	1.693294000
C	-6.906683000	-3.410960000	5.296541000
C	-5.725633000	-3.588032000	4.610495000
C	-5.326100000	-2.670575000	3.601420000
C	-6.175323000	-1.556320000	3.305123000

C	-7.387975000	-1.402582000	4.028539000
C	-7.745944000	-2.307732000	5.003047000
H	-3.493365000	-3.690376000	3.104683000
H	-7.200833000	-4.119583000	6.065914000
H	-5.081592000	-4.435769000	4.832715000
C	-4.112571000	-2.826704000	2.876487000
C	-5.772042000	-0.652282000	2.285413000
H	-8.031411000	-0.556631000	3.798324000
H	-8.675937000	-2.180118000	5.550273000
C	-4.589789000	-0.833982000	1.607116000
C	-3.727649000	-1.928761000	1.900583000
H	-6.421702000	0.183196000	2.035101000
H	-4.316291000	-0.141981000	0.815005000
C	-2.430669000	-2.068738000	1.109378000
H	-2.602142000	-1.667217000	0.116059000
C	-1.922859000	-3.505202000	0.942510000
H	-1.059617000	-3.503782000	0.271962000
H	-1.619719000	-3.949711000	1.896545000
H	-2.704632000	-4.129293000	0.498744000
C	5.805645000	-3.915533000	1.927123000
C	6.926015000	3.780118000	2.106606000
C	5.912005000	3.103315000	1.465788000
C	5.042515000	3.782214000	0.568460000
C	5.238704000	5.182438000	0.339155000
C	6.294875000	5.850278000	1.015078000
C	7.120014000	5.165552000	1.879289000
H	3.862120000	2.048127000	0.054675000
H	7.584451000	3.251355000	2.790445000
H	5.759257000	2.041684000	1.642985000
C	3.985430000	3.116497000	-0.109244000
C	4.371509000	5.845386000	-0.569605000
H	6.442028000	6.913050000	0.836979000
H	7.925455000	5.685957000	2.390508000
C	3.358186000	5.167532000	-1.207794000
C	3.135129000	3.780684000	-0.972751000
H	4.527400000	6.903965000	-0.764901000
H	2.735240000	5.700656000	-1.920638000
C	2.023669000	3.007692000	-1.672456000
H	2.210585000	1.939891000	-1.581700000
C	1.894749000	3.310020000	-3.171238000
H	1.186690000	2.605131000	-3.613733000
H	1.553167000	4.332953000	-3.365910000
H	2.865638000	3.177109000	-3.659880000

C	-0.123068000	2.180669000	-0.608408000
C	-1.113179000	4.219836000	-0.305087000
C	0.114318000	4.438883000	-0.832606000
H	-1.901092000	4.905880000	-0.036806000
H	0.612515000	5.360954000	-1.082059000
N	0.704459000	3.195268000	-1.010678000
N	-1.242000000	2.845140000	-0.172979000
C	-8.126777000	4.313225000	-1.376714000
C	-7.084346000	4.055491000	-0.513406000
C	-5.896662000	3.427443000	-0.974715000
C	-5.797751000	3.065171000	-2.355904000
C	-6.889281000	3.343143000	-3.220462000
C	-8.028637000	3.953374000	-2.743039000
H	-4.902426000	3.429569000	0.937749000
H	-9.030036000	4.794346000	-1.011460000
H	-7.158001000	4.331483000	0.536207000
C	-4.803244000	3.148822000	-0.106935000
C	-4.603954000	2.437365000	-2.807775000
H	-6.810466000	3.065176000	-4.268766000
H	-8.857576000	4.160971000	-3.414122000
C	-3.565971000	2.183927000	-1.944428000
C	-3.653519000	2.540059000	-0.566173000
H	-4.522286000	2.155340000	-3.854726000
H	-2.661279000	1.704819000	-2.309379000
C	-2.470311000	2.205145000	0.339347000
H	-2.262862000	1.136540000	0.247837000
C	-2.664830000	2.526272000	1.824600000
H	-1.777555000	2.220035000	2.384980000
H	-2.826910000	3.595660000	1.998379000
H	-3.522393000	1.977525000	2.224961000
C	-0.077268000	-5.030353000	-5.086058000
C	0.478660000	-4.134533000	-4.197965000
C	-0.326999000	-3.311924000	-3.356774000
C	-1.749723000	-3.390767000	-3.546765000
C	-2.294514000	-4.342103000	-4.452020000
C	-1.480863000	-5.158767000	-5.201977000
H	0.573115000	-5.638669000	-5.709813000
H	1.555653000	-4.048348000	-4.146694000
C	0.210528000	-2.369454000	-2.395128000
C	-2.584913000	-2.457258000	-2.885190000
H	-3.376590000	-4.392971000	-4.556741000
H	-1.908452000	-5.877462000	-5.896479000
C	-2.036426000	-1.509290000	-2.050857000

C	4.354672000	-1.976336000	2.122243000
H	-3.656127000	-2.476052000	-3.079567000
H	-2.695717000	-0.749734000	-1.632358000
C	4.723113000	-5.088508000	-1.830427000
C	5.056361000	-3.789809000	-2.151088000
C	4.064038000	-2.783464000	-2.279359000
C	2.676153000	-3.123607000	-2.114311000
C	2.374668000	-4.470627000	-1.748593000
C	3.364086000	-5.420815000	-1.610083000
H	5.446170000	-1.173474000	-2.719359000
H	5.493561000	-5.849411000	-1.736289000
H	6.097346000	-3.509444000	-2.300253000
C	4.401276000	-1.432087000	-2.558813000
C	1.667893000	-2.120264000	-2.276338000
H	1.338094000	-4.743976000	-1.579803000
H	3.096923000	-6.436594000	-1.328357000
C	2.049906000	-0.762208000	-2.385261000
C	3.435866000	-0.458798000	-2.594228000
H	3.703840000	0.576619000	-2.786646000
C	4.740663000	-3.176741000	1.464253000

146

B, $E_{\text{(SCF Done)}}$ = -6133.786163 a.u.

Ni	-0.179422000	0.495600000	-0.285868000
O	1.043178000	1.827842000	-1.191527000
C	-0.642596000	0.070603000	-2.097854000
C	4.851680000	-3.895635000	1.753499000
C	5.935353000	-4.803625000	1.604425000
C	6.389803000	-5.154018000	0.353271000
H	2.700318000	-2.027718000	-0.170820000
H	6.148213000	-4.894878000	-1.789531000
H	4.265805000	-3.314927000	-1.586885000
C	3.156134000	-2.439277000	0.727807000
C	4.348427000	-3.508419000	3.020861000
H	6.398432000	-5.217716000	2.497217000
H	7.218626000	-5.849393000	0.249843000
C	3.289523000	-2.634044000	3.129274000
C	2.669940000	-2.085906000	1.973671000
H	4.812744000	-3.912545000	3.917407000
H	2.938692000	-2.356616000	4.117113000
C	1.467479000	-1.142075000	2.073428000
H	1.656924000	-0.261824000	1.457119000
C	1.133909000	-0.687779000	3.498150000
H	0.301715000	0.017922000	3.478284000

H	0.848111000	-1.532754000	4.134006000
H	1.993711000	-0.187840000	3.950684000
C	-0.538682000	-1.299261000	0.476473000
C	-1.184855000	-3.392909000	1.119203000
C	-0.107098000	-3.063509000	1.862061000
H	-1.796527000	-4.279754000	1.114900000
H	0.433750000	-3.618483000	2.611474000
N	0.278907000	-1.793412000	1.463829000
N	-1.440804000	-2.319314000	0.277507000
C	-6.969424000	-6.544752000	0.640994000
C	-5.752386000	-6.131312000	0.146506000
C	-5.382183000	-4.759625000	0.180291000
C	-6.298798000	-3.809557000	0.735291000
C	-7.547214000	-4.267171000	1.234985000
C	-7.875436000	-5.604020000	1.190578000
H	-3.462202000	-5.038955000	-0.756951000
H	-7.240940000	-7.596316000	0.609491000
H	-5.057166000	-6.851787000	-0.278216000
C	-4.132995000	-4.302378000	-0.322182000
C	-5.923611000	-2.439109000	0.751854000
H	-8.241722000	-3.542079000	1.652544000
H	-8.833191000	-5.943037000	1.575647000
C	-4.705399000	-2.033872000	0.258774000
C	-3.776980000	-2.968418000	-0.281619000
H	-6.621628000	-1.705173000	1.147614000
H	-4.455252000	-0.976508000	0.261253000
C	-2.444642000	-2.449451000	-0.814277000
H	-2.604985000	-1.441174000	-1.178906000
C	-1.845225000	-3.268791000	-1.962729000
H	-0.954534000	-2.760669000	-2.341057000
H	-1.558532000	-4.276537000	-1.643882000
H	-2.569244000	-3.353800000	-2.778750000
C	5.780502000	-4.612109000	-0.806991000
C	6.859253000	-0.130903000	3.503623000
C	5.878995000	0.303001000	2.639080000
C	4.829472000	1.144281000	3.097391000
C	4.802673000	1.530419000	4.475710000
C	5.827836000	1.065951000	5.342357000
C	6.834726000	0.255165000	4.866862000
H	3.840993000	1.313661000	1.185157000
H	7.656297000	-0.774399000	3.141781000
H	5.891200000	0.002770000	1.594550000
C	3.804244000	1.612032000	2.229972000

C	3.746201000	2.371453000	4.919277000
H	5.806287000	1.364347000	6.388014000
H	7.616166000	-0.092345000	5.537340000
C	2.775375000	2.812142000	4.050238000
C	2.786559000	2.431570000	2.677696000
H	3.719778000	2.673040000	5.963965000
H	1.990769000	3.460691000	4.427058000
C	1.757804000	2.966229000	1.684926000
H	1.908546000	2.478741000	0.723397000
C	1.896412000	4.484679000	1.487282000
H	1.113830000	4.869510000	0.825997000
H	1.850958000	5.030022000	2.436451000
H	2.860236000	4.703006000	1.020715000
C	-0.383847000	1.620912000	1.465710000
C	-1.588997000	2.709001000	3.072862000
C	-0.367802000	3.290721000	3.019122000
H	-2.461261000	2.945754000	3.661339000
H	0.021179000	4.137873000	3.558492000
N	0.358689000	2.617219000	2.046424000
N	-1.582528000	1.696077000	2.128579000
C	-8.605401000	3.083133000	2.198237000
C	-7.529882000	2.310837000	2.579188000
C	-6.299101000	2.379879000	1.872991000
C	-6.191607000	3.268113000	0.755022000
C	-7.317986000	4.050869000	0.387376000
C	-8.498621000	3.961196000	1.091929000
H	-5.276012000	0.929463000	3.096207000
H	-9.541377000	3.021680000	2.746864000
H	-7.609873000	1.637276000	3.429723000
C	-5.170524000	1.595766000	2.244779000
C	-4.954780000	3.331601000	0.055685000
H	-7.231575000	4.724145000	-0.461935000
H	-9.353719000	4.565273000	0.801282000
C	-3.885513000	2.559267000	0.438916000
C	-3.980525000	1.672331000	1.549815000
H	-4.852309000	4.004238000	-0.792084000
H	-2.950868000	2.636507000	-0.107558000
C	-2.758046000	0.827221000	1.897888000
H	-2.483207000	0.244510000	1.016192000
C	-2.940641000	-0.139764000	3.072210000
H	-2.023336000	-0.714067000	3.224503000
H	-3.173546000	0.387957000	4.003457000
H	-3.749832000	-0.845032000	2.861964000

C	0.428437000	0.134925000	-6.883564000
C	0.854139000	0.084765000	-5.573616000
C	-0.070595000	0.028330000	-4.489755000
C	-1.467179000	0.114638000	-4.818143000
C	-1.874664000	0.142277000	-6.179753000
C	-0.950405000	0.141020000	-7.197988000
H	1.163421000	0.179838000	-7.683032000
H	1.915993000	0.099430000	-5.366335000
C	0.319739000	-0.040291000	-3.096966000
C	-2.413229000	0.254118000	-3.772461000
H	-2.939161000	0.189857000	-6.399030000
H	-1.272686000	0.171616000	-8.235362000
C	-2.002158000	0.272070000	-2.458516000
C	4.238147000	-3.345626000	0.580422000
H	-3.462514000	0.391912000	-4.026572000
H	-2.744334000	0.476673000	-1.688553000
C	4.970687000	-2.326995000	-4.147208000
C	5.229427000	-1.206960000	-3.388678000
C	4.173568000	-0.413684000	-2.864864000
C	2.812111000	-0.764855000	-3.159263000
C	2.583010000	-1.953672000	-3.912284000
C	3.629607000	-2.711308000	-4.392745000
H	5.455124000	1.030003000	-1.881245000
H	5.788180000	-2.921267000	-4.546713000
H	6.253889000	-0.911399000	-3.173543000
C	4.425801000	0.731536000	-2.065802000
C	1.744357000	0.064392000	-2.682179000
H	1.560281000	-2.253596000	-4.115573000
H	3.423584000	-3.610290000	-4.968106000
C	2.038637000	1.108616000	-1.795089000
C	3.391031000	1.462755000	-1.533344000
H	3.585523000	2.359018000	-0.955356000
C	4.730250000	-3.729287000	-0.697635000
C	-1.610659000	4.205671000	-1.927086000
H	-1.940168000	4.597609000	-0.953240000
H	-2.316237000	3.416610000	-2.222028000
H	-1.736468000	5.028555000	-2.645677000
Mg	0.407320000	3.586956000	-1.945857000
Br	2.384471000	4.933361000	-2.462626000

146

TS2, $E_{\text{(SCF Done)}}$ = -6133.780120 a.u., IF = -84.35 cm^{-1}

Ni	-0.266833000	-0.224472000	0.748598000
C	-0.313931000	1.367960000	1.846616000

C	-1.171650000	-1.539085000	3.232758000
Mg	0.755192000	-0.646502000	3.522193000
C	6.289767000	1.641379000	-4.164936000
C	6.414199000	2.977173000	-4.478983000
H	1.935800000	2.214690000	-2.773209000
H	5.436477000	4.913753000	-4.524203000
H	3.302520000	4.073348000	-3.601974000
C	2.743138000	1.508919000	-2.937446000
C	4.897646000	-0.238277000	-3.302093000
H	7.123658000	0.959120000	-4.313636000
H	7.349789000	3.358398000	-4.879419000
C	3.695649000	-0.702533000	-2.827005000
C	2.578737000	0.168914000	-2.656190000
H	5.734287000	-0.922931000	-3.420026000
H	3.603616000	-1.751610000	-2.562884000
C	1.269791000	-0.467433000	-2.180470000
H	1.419547000	-0.868286000	-1.175517000
C	0.835709000	-1.609400000	-3.121070000
H	-0.074206000	-2.085439000	-2.747595000
H	0.647930000	-1.234323000	-4.132927000
H	1.613902000	-2.371018000	-3.185558000
C	-0.590346000	0.737823000	-0.920584000
C	-1.359578000	1.955737000	-2.686824000
C	-0.322022000	1.215645000	-3.134793000
H	-2.006508000	2.646512000	-3.201442000
H	0.125508000	1.149709000	-4.112762000
N	0.154932000	0.492188000	-2.049066000
N	-1.514909000	1.662234000	-1.339395000
C	-7.290916000	4.523884000	-3.905660000
C	-6.018047000	4.532673000	-3.380454000
C	-5.586355000	3.500433000	-2.503778000
C	-6.500239000	2.449231000	-2.170626000
C	-7.807082000	2.469571000	-2.727478000
C	-8.194016000	3.482431000	-3.576460000
H	-3.611960000	4.302704000	-2.196605000
H	-7.609194000	5.318688000	-4.574591000
H	-5.324998000	5.332819000	-3.629995000
C	-4.279011000	3.481776000	-1.946014000
C	-6.063020000	1.430711000	-1.282223000
H	-8.498597000	1.671104000	-2.469306000
H	-9.196068000	3.488556000	-3.996394000
C	-4.789217000	1.445888000	-0.762630000
C	-3.862688000	2.472844000	-1.098301000

H	-6.753403000	0.637549000	-1.005724000
H	-4.490078000	0.668036000	-0.066041000
C	-2.464054000	2.430368000	-0.487679000
H	-2.520127000	1.870669000	0.441679000
C	-1.867881000	3.808569000	-0.177333000
H	-0.908001000	3.684688000	0.328973000
H	-1.703290000	4.397117000	-1.086055000
H	-2.540806000	4.366786000	0.480281000
C	5.325560000	3.860947000	-4.278916000
C	7.324264000	-4.415426000	-1.099665000
C	6.308460000	-3.992436000	-0.273044000
C	4.947226000	-4.151345000	-0.655264000
C	4.650575000	-4.759303000	-1.918265000
C	5.722921000	-5.185180000	-2.749032000
C	7.029450000	-5.017683000	-2.349747000
H	4.118616000	-3.267124000	1.127595000
H	8.359964000	-4.289804000	-0.796187000
H	6.532868000	-3.530802000	0.685447000
C	3.876110000	-3.725626000	0.171318000
C	3.288764000	-4.905681000	-2.286488000
H	5.492707000	-5.646954000	-3.706442000
H	7.842123000	-5.347589000	-2.991154000
C	2.271878000	-4.479478000	-1.457491000
C	2.550755000	-3.877238000	-0.200635000
H	3.050493000	-5.365318000	-3.243199000
H	1.244715000	-4.601187000	-1.782708000
C	1.479818000	-3.407990000	0.787440000
H	1.719601000	-2.386716000	1.093529000
C	1.451128000	-4.289991000	2.051266000
H	0.690454000	-3.926386000	2.747809000
H	1.245414000	-5.339880000	1.814468000
H	2.421872000	-4.248035000	2.552531000
C	-0.607546000	-2.166048000	0.070806000
C	-1.842818000	-3.995160000	-0.522280000
C	-0.624580000	-4.438499000	-0.134068000
H	-2.721962000	-4.528560000	-0.846770000
H	-0.235984000	-5.440984000	-0.057046000
N	0.122495000	-3.318349000	0.209459000
N	-1.819271000	-2.615254000	-0.390708000
C	-8.857795000	-3.967412000	-0.504278000
C	-7.713451000	-3.476239000	-1.093528000
C	-6.570384000	-3.162136000	-0.310093000
C	-6.624846000	-3.364592000	1.105467000

C	-7.818809000	-3.871683000	1.683359000
C	-8.911106000	-4.166742000	0.897250000
H	-5.362526000	-2.507172000	-1.965888000
H	-9.725470000	-4.204655000	-1.113826000
H	-7.670243000	-3.323396000	-2.169641000
C	-5.374394000	-2.654282000	-0.890434000
C	-5.473340000	-3.047436000	1.876573000
H	-7.856191000	-4.023601000	2.759384000
H	-9.819416000	-4.554478000	1.350217000
C	-4.333018000	-2.557805000	1.286248000
C	-4.267123000	-2.349642000	-0.123261000
H	-5.503790000	-3.197085000	2.953171000
H	-3.467411000	-2.331256000	1.902020000
C	-2.987558000	-1.769115000	-0.728453000
H	-2.771524000	-0.816393000	-0.238261000
C	-3.051682000	-1.529385000	-2.240466000
H	-2.119765000	-1.079166000	-2.586000000
H	-3.205823000	-2.464602000	-2.789985000
H	-3.867727000	-0.842826000	-2.482548000
C	1.834618000	4.622644000	4.821182000
C	1.927552000	3.713397000	3.790014000
C	0.772555000	3.064766000	3.266261000
C	-0.483576000	3.341569000	3.901671000
C	-0.551004000	4.301367000	4.947252000
C	0.581859000	4.939626000	5.395637000
H	2.736804000	5.092279000	5.203831000
H	2.899603000	3.473636000	3.378740000
C	0.822589000	2.114863000	2.169855000
C	-1.627974000	2.600382000	3.511294000
H	-1.517659000	4.502587000	5.403757000
H	0.521840000	5.663807000	6.203708000
C	-1.529504000	1.622062000	2.549419000
C	3.970987000	2.020560000	-3.444002000
H	-2.572607000	2.781692000	4.020625000
H	-2.405381000	1.010568000	2.344589000
C	4.638752000	4.810537000	-0.267121000
C	5.063553000	3.504075000	-0.178546000
C	4.231994000	2.495490000	0.375307000
C	2.934935000	2.842701000	0.885146000
C	2.520257000	4.200796000	0.750472000
C	3.344753000	5.153352000	0.191625000
H	5.628980000	0.875054000	0.044238000
H	5.284068000	5.571197000	-0.698051000

H	6.044038000	3.217282000	-0.551896000
C	4.643308000	1.138061000	0.420608000
C	2.106735000	1.828449000	1.470846000
H	1.535366000	4.487194000	1.102561000
H	2.996784000	6.179979000	0.105066000
C	2.510651000	0.486747000	1.398235000
C	3.801888000	0.163292000	0.894191000
H	4.102944000	-0.879226000	0.899415000
C	4.133915000	3.393393000	-3.769684000
O	1.702506000	-0.516569000	1.796579000
C	5.071779000	1.129693000	-3.644213000
Br	2.038562000	-0.595424000	5.576128000
H	-1.741122000	-1.153975000	4.093872000
H	-1.772895000	-1.296054000	2.347370000
H	-1.152968000	-2.635285000	3.313725000

146

C, *E*(SCF Done) = -6133.817633 a.u.

Ni	0.567622000	0.559620000	-0.565611000
C	1.648720000	0.782960000	-2.299604000
C	0.371687000	2.562497000	-0.506645000
C	-1.791874000	-1.531181000	1.175848000
H	-1.797421000	-1.184186000	0.139777000
C	-1.159530000	-2.929359000	1.250684000
H	-0.120782000	-2.893049000	0.923050000
H	-1.190695000	-3.314387000	2.275859000
H	-1.689755000	-3.628119000	0.602500000
C	0.018701000	0.269154000	1.359458000
C	0.050149000	0.344822000	3.643105000
C	-0.903484000	-0.542284000	3.286362000
H	0.340669000	0.696208000	4.618443000
H	-1.593404000	-1.116522000	3.882753000
N	-0.919512000	-0.575701000	1.899735000
N	0.613233000	0.826375000	2.468616000
C	1.710968000	1.819897000	2.464731000
H	1.752369000	2.173623000	1.439484000
C	1.359849000	3.018067000	3.360894000
H	0.370634000	3.407949000	3.104663000
H	1.370929000	2.761318000	4.425709000
H	2.100118000	3.808672000	3.205955000
C	-0.999845000	-1.700566000	-2.667725000
H	-1.188490000	-0.678706000	-2.326946000
C	-0.688646000	-1.659564000	-4.177339000
H	0.194626000	-1.048165000	-4.382679000

H	-0.524115000	-2.665481000	-4.578953000
H	-1.536023000	-1.221589000	-4.711734000
C	0.856371000	-1.382035000	-0.990586000
C	1.943894000	-3.354220000	-1.341355000
C	0.851974000	-3.347273000	-2.140839000
H	2.730787000	-4.081516000	-1.221840000
H	0.510916000	-4.070974000	-2.862678000
N	0.185059000	-2.148296000	-1.906844000
N	1.935316000	-2.149188000	-0.649591000
C	2.992465000	-1.765851000	0.317257000
H	2.891869000	-0.681476000	0.418794000
C	2.718894000	-2.393813000	1.687289000
H	1.733777000	-2.087038000	2.044831000
H	2.745206000	-3.488333000	1.642153000
H	3.459469000	-2.053480000	2.415941000
C	-1.793253000	6.967597000	-0.968207000
C	-1.889385000	5.600764000	-0.814445000
C	-0.731889000	4.789007000	-0.644608000
C	0.539301000	5.444782000	-0.690524000
C	0.609571000	6.856188000	-0.826921000
C	-0.534378000	7.609700000	-0.956534000
H	-2.696636000	7.555897000	-1.105136000
H	-2.864088000	5.130093000	-0.836417000
C	-0.803525000	3.341899000	-0.459167000
C	1.711543000	4.647925000	-0.652639000
H	1.589819000	7.326991000	-0.849687000
H	-0.472677000	8.688630000	-1.070906000
C	1.615416000	3.279873000	-0.626218000
H	2.683429000	5.135139000	-0.707025000
H	2.538331000	2.709494000	-0.697602000
C	-4.617873000	4.081411000	3.020042000
C	-5.161954000	3.327710000	2.002009000
C	-4.369334000	2.862971000	0.920728000
C	-2.974290000	3.199355000	0.858926000
C	-2.448928000	3.977671000	1.933388000
C	-3.240632000	4.399179000	2.981105000
H	-5.978939000	1.812455000	-0.080496000
H	-5.237043000	4.430622000	3.841951000
H	-6.220807000	3.074988000	2.009173000
C	-4.919704000	2.058434000	-0.113654000
C	-2.175151000	2.764856000	-0.253998000
H	-1.400286000	4.254022000	1.915184000
H	-2.801188000	4.989430000	3.781716000

C	-2.739395000	1.909849000	-1.223032000
C	-4.132338000	1.583394000	-1.126179000
H	-4.551635000	0.967278000	-1.915293000
O	-2.060487000	1.429784000	-2.253378000
H	2.568982000	0.647178000	-1.717228000
H	1.838603000	1.724540000	-2.842324000
H	1.621048000	-0.014271000	-3.049317000
Mg	-0.296917000	2.056356000	-2.703490000
Br	0.098596000	3.078265000	-4.860369000
C	-3.222450000	-1.501499000	1.727151000
C	-3.999129000	-2.644996000	1.764054000
C	-3.789507000	-0.283143000	2.194505000
C	-5.328116000	-2.633253000	2.266284000
H	-3.616573000	-3.592389000	1.400573000
C	-5.063339000	-0.243759000	2.711289000
H	-3.210116000	0.631557000	2.143236000
C	-6.131778000	-3.805580000	2.294768000
C	-5.871217000	-1.409183000	2.769377000
H	-5.464902000	0.700090000	3.069720000
C	-7.410103000	-3.769441000	2.805910000
H	-5.722132000	-4.731001000	1.896810000
C	-7.192108000	-1.405080000	3.293061000
C	-7.944674000	-2.558350000	3.312660000
H	-8.016227000	-4.671381000	2.821264000
H	-7.600323000	-0.472638000	3.676111000
H	-8.954520000	-2.543569000	3.714027000
C	3.073959000	1.228217000	2.816707000
C	4.122531000	1.372824000	1.928693000
C	3.329880000	0.598226000	4.068126000
C	5.432160000	0.904281000	2.222799000
H	3.956678000	1.869958000	0.975602000
C	4.583961000	0.129967000	4.386133000
H	2.528915000	0.480774000	4.790520000
C	6.510439000	1.046493000	1.308459000
C	5.671473000	0.264774000	3.481241000
H	4.758894000	-0.346852000	5.347963000
C	7.765906000	0.576852000	1.623704000
H	6.326875000	1.528272000	0.351263000
C	6.978372000	-0.205980000	3.778317000
C	8.002574000	-0.053984000	2.870242000
H	8.581494000	0.686486000	0.914692000
H	7.156935000	-0.688421000	4.736557000
H	8.998481000	-0.418795000	3.105842000

C	4.372148000	-2.072822000	-0.266160000
C	5.300035000	-2.851977000	0.393726000
C	4.724646000	-1.517528000	-1.531721000
C	6.589008000	-3.105412000	-0.153090000
H	5.070847000	-3.287221000	1.361245000
C	5.960169000	-1.743056000	-2.088392000
H	3.998571000	-0.913523000	-2.067078000
C	7.548310000	-3.906693000	0.521834000
C	6.930564000	-2.540857000	-1.422592000
H	6.208644000	-1.310110000	-3.054545000
C	8.787641000	-4.139770000	-0.032980000
H	7.288162000	-4.333849000	1.487570000
C	8.215892000	-2.798205000	-1.968535000
C	9.124801000	-3.580356000	-1.289933000
H	9.512726000	-4.754995000	0.492843000
H	8.470913000	-2.367715000	-2.934046000
H	10.105206000	-3.771774000	-1.717521000
C	-2.273668000	-2.504656000	-2.410888000
C	-2.318867000	-3.818255000	-1.990790000
C	-3.498884000	-1.836757000	-2.703090000
C	-3.553528000	-4.508937000	-1.834258000
H	-1.409529000	-4.359296000	-1.749346000
C	-4.706887000	-2.477820000	-2.570188000
H	-3.458904000	-0.797684000	-3.017174000
C	-3.611111000	-5.857113000	-1.387439000
C	-4.776749000	-3.827927000	-2.132090000
H	-5.631208000	-1.948474000	-2.788555000
C	-4.820548000	-6.501500000	-1.241707000
H	-2.681336000	-6.376401000	-1.164743000
C	-6.006962000	-4.518928000	-1.971899000
C	-6.030554000	-5.825977000	-1.536774000
H	-4.849973000	-7.533486000	-0.902297000
H	-6.933366000	-3.995944000	-2.196425000
H	-6.978217000	-6.344281000	-1.418967000

146

TS3, $E_{\text{(SCF Done)}}$ = -6133.762984 a.u., IF = -381.81 cm^{-1}

Ni	0.148191000	0.236739000	-0.435923000
C	0.511726000	1.375258000	-2.149588000
C	1.233608000	1.789238000	-0.415506000
C	-2.749880000	-0.935468000	0.846022000
H	-2.389212000	-0.633028000	-0.139361000
C	-2.735079000	-2.466579000	0.915847000
H	-1.731652000	-2.826022000	0.673600000

H	-2.996712000	-2.826286000	1.917147000
H	-3.436923000	-2.893888000	0.195310000
C	-0.453900000	-0.044761000	1.432064000
C	-0.746035000	-0.026610000	3.705937000
C	-1.927401000	-0.387914000	3.153206000
H	-0.471634000	0.119748000	4.737944000
H	-2.883412000	-0.606849000	3.602207000
N	-1.735361000	-0.401091000	1.780317000
N	0.143752000	0.171553000	2.654250000
C	1.565928000	0.488119000	2.844701000
H	1.898047000	0.871694000	1.881907000
C	1.761609000	1.598134000	3.894883000
H	1.162892000	2.479418000	3.647905000
H	1.499712000	1.257324000	4.902303000
H	2.812751000	1.898637000	3.913721000
C	-2.084194000	-1.022900000	-3.033521000
H	-2.033622000	-0.119232000	-2.421994000
C	-1.885106000	-0.616042000	-4.505857000
H	-0.920097000	-0.116785000	-4.636319000
H	-1.929687000	-1.485469000	-5.170148000
H	-2.677161000	0.073414000	-4.812119000
C	-0.161490000	-1.530067000	-1.488511000
C	0.514360000	-3.481996000	-2.478392000
C	-0.544734000	-3.021874000	-3.186163000
H	1.139819000	-4.348545000	-2.625518000
H	-1.022505000	-3.418484000	-4.067760000
N	-0.957720000	-1.845124000	-2.565840000
N	0.729858000	-2.570371000	-1.454621000
C	1.794749000	-2.723066000	-0.442871000
H	1.761515000	-1.780990000	0.116696000
C	1.439018000	-3.862105000	0.518893000
H	0.438933000	-3.693590000	0.927763000
H	1.449408000	-4.835542000	0.015725000
H	2.138553000	-3.896114000	1.357333000
C	2.970206000	5.873812000	1.806939000
C	1.940587000	5.208297000	1.178928000
C	2.117271000	3.880165000	0.659833000
C	3.437483000	3.304803000	0.789127000
C	4.475040000	4.027975000	1.429984000
C	4.252040000	5.286443000	1.944583000
H	2.793596000	6.870637000	2.202883000
H	0.965989000	5.673853000	1.089016000
C	1.047435000	3.154453000	0.016145000

C	3.653995000	2.034971000	0.178844000
H	5.458793000	3.568645000	1.496003000
H	5.052176000	5.831804000	2.435970000
C	2.625646000	1.342071000	-0.412439000
H	4.663946000	1.632177000	0.148933000
H	2.863536000	0.399710000	-0.895450000
C	-3.455931000	4.497743000	2.400321000
C	-3.478098000	5.086202000	1.152905000
C	-2.419067000	4.895431000	0.228072000
C	-1.302924000	4.065260000	0.592332000
C	-1.324708000	3.458801000	1.880905000
C	-2.364498000	3.672634000	2.761007000
H	-3.255868000	6.156032000	-1.323232000
H	-4.267992000	4.664234000	3.103005000
H	-4.306977000	5.729827000	0.863038000
C	-2.405131000	5.538672000	-1.040674000
C	-0.203640000	3.919498000	-0.305749000
H	-0.495696000	2.819312000	2.160487000
H	-2.347272000	3.199251000	3.739492000
C	-0.187316000	4.639091000	-1.517028000
C	-1.331454000	5.416329000	-1.883603000
H	-1.298610000	5.930228000	-2.840087000
O	0.863931000	4.631674000	-2.326725000
H	-0.546028000	1.156990000	-2.337771000
H	1.153455000	0.731650000	-2.758988000
H	0.625594000	2.406437000	-2.480763000
Mg	2.548605000	3.915079000	-1.793270000
Br	4.575578000	3.922325000	-3.057462000
C	-4.113897000	-0.293235000	1.074673000
C	-5.231213000	-1.025558000	1.421721000
C	-4.245485000	1.111861000	0.880363000
C	-6.505151000	-0.412912000	1.579996000
H	-5.167686000	-2.099694000	1.566885000
C	-5.461861000	1.734592000	1.026109000
H	-3.367794000	1.698970000	0.626149000
C	-7.662965000	-1.158569000	1.929775000
C	-6.626384000	0.998601000	1.377388000
H	-5.536284000	2.808498000	0.880797000
C	-8.884373000	-0.537904000	2.075259000
H	-7.569344000	-2.231870000	2.079331000
C	-7.899261000	1.609200000	1.534579000
C	-9.003741000	0.859722000	1.876276000
H	-9.762409000	-1.119345000	2.343680000

H	-7.986819000	2.682254000	1.380850000
H	-9.972110000	1.338519000	1.994671000
C	2.416742000	-0.736146000	3.183929000
C	1.911664000	-1.900184000	3.725418000
C	3.821499000	-0.638436000	2.963823000
C	2.759245000	-2.989074000	4.073790000
H	0.844237000	-2.010441000	3.894096000
C	4.668213000	-1.672475000	3.284316000
H	4.223065000	0.271450000	2.526025000
C	2.249808000	-4.191230000	4.636441000
C	4.169400000	-2.876685000	3.851076000
H	5.736096000	-1.580910000	3.100838000
C	3.092730000	-5.230384000	4.964657000
H	1.178945000	-4.276507000	4.806788000
C	5.012310000	-3.966198000	4.198295000
C	4.487650000	-5.117420000	4.743586000
H	2.689571000	-6.142438000	5.396502000
H	6.082449000	-3.874538000	4.027550000
H	5.142349000	-5.943414000	5.007832000
C	-3.462017000	-1.645555000	-2.806834000
C	-3.692815000	-2.992481000	-2.618846000
C	-4.576515000	-0.757130000	-2.820582000
C	-5.009276000	-3.506075000	-2.448996000
H	-2.867079000	-3.696087000	-2.578855000
C	-5.860023000	-1.218971000	-2.656767000
H	-4.402796000	0.308778000	-2.948289000
C	-5.256890000	-4.892147000	-2.255303000
C	-6.119887000	-2.603324000	-2.469432000
H	-6.694207000	-0.522046000	-2.654962000
C	-6.541212000	-5.362901000	-2.091888000
H	-4.413077000	-5.578360000	-2.240084000
C	-7.431743000	-3.118492000	-2.294590000
C	-7.639286000	-4.467893000	-2.111047000
H	-6.716913000	-6.425432000	-1.946930000
H	-8.271004000	-2.427364000	-2.306072000
H	-8.647382000	-4.851462000	-1.979773000
C	3.161624000	-2.847847000	-1.114986000
C	4.116487000	-3.746851000	-0.685083000
C	3.487267000	-1.972545000	-2.192802000
C	5.405409000	-3.812055000	-1.283796000
H	3.908321000	-4.426174000	0.135580000
C	4.724210000	-2.005913000	-2.791106000
H	2.738487000	-1.272194000	-2.550329000

C	6.392476000	-4.735498000	-0.845748000
C	5.721242000	-2.922327000	-2.359393000
H	4.952603000	-1.324573000	-3.607054000
C	7.633463000	-4.776215000	-1.442521000
H	6.151718000	-5.410661000	-0.027712000
C	7.009314000	-2.987671000	-2.953888000
C	7.945312000	-3.893934000	-2.506226000
H	8.380114000	-5.486669000	-1.098049000
H	7.244934000	-2.307536000	-3.768871000
H	8.928270000	-3.934713000	-2.967445000

39

3a-Mg, $E_{(\mathrm{SCF\ Done})}$ = -3658.320298 a.u.

C	-0.450265000	1.366183000	1.638009000
C	-1.451352000	1.066261000	2.728285000
Mg	1.749187000	-1.101909000	0.099761000
C	1.632535000	0.554983000	-2.723604000
C	0.634833000	0.270145000	-1.808522000
C	0.550963000	0.977070000	-0.561426000
C	1.530208000	2.001497000	-0.305805000
C	2.524915000	2.276314000	-1.283155000
C	2.584893000	1.568402000	-2.462109000
H	1.671682000	0.007158000	-3.660812000
H	-0.132468000	-0.463298000	-2.039380000
C	-0.459685000	0.670239000	0.425090000
C	1.475301000	2.700360000	0.926005000
H	3.255526000	3.052452000	-1.071233000
H	3.362493000	1.779557000	-3.189427000
C	0.519338000	2.382905000	1.860441000
H	2.211219000	3.474052000	1.127441000
H	0.503343000	2.911263000	2.810177000
C	-5.584569000	0.534756000	-0.578060000
C	-5.168256000	-0.777308000	-0.508848000
C	-3.809653000	-1.106278000	-0.270706000
C	-2.847608000	-0.057397000	-0.088794000
C	-3.310240000	1.286109000	-0.181418000
C	-4.639095000	1.572988000	-0.416499000
H	-4.098990000	-3.251840000	-0.345274000
H	-6.628506000	0.772673000	-0.761920000
H	-5.881035000	-1.588602000	-0.640884000
C	-3.367218000	-2.457552000	-0.213903000
C	-1.475519000	-0.394096000	0.153921000
H	-2.596658000	2.098275000	-0.076892000
H	-4.960842000	2.609212000	-0.484303000

C	-1.067813000	-1.739223000	0.170194000
C	-2.048759000	-2.764646000	-0.008459000
H	-1.703733000	-3.793621000	0.029084000
O	0.195528000	-2.103618000	0.352115000
Br	4.084543000	-1.146053000	0.466326000
H	-1.118925000	1.477317000	3.686392000
H	-2.431663000	1.500068000	2.496091000
H	-1.602631000	-0.011251000	2.841644000

140

TS1′′, $E_{(SCF\ Done)}$ = -3319.617261 a.u., IF = -363.18 cm^{-1}

Ni	-0.210029000	-0.076238000	-0.731706000
C	5.602288000	-0.145839000	2.965219000
C	6.890128000	-0.610202000	3.347924000
C	7.526235000	-1.592850000	2.623002000
H	3.215476000	-0.693674000	0.551170000
H	7.413332000	-2.931008000	0.914480000
H	5.178900000	-2.166446000	0.210233000
C	3.687394000	-0.251353000	1.426768000
C	4.912930000	0.868900000	3.677775000
H	7.364838000	-0.174932000	4.224560000
H	8.510832000	-1.940730000	2.924671000
C	3.667620000	1.299298000	3.276143000
C	3.030939000	0.737647000	2.136340000
H	5.387579000	1.312939000	4.549961000
H	3.176842000	2.089805000	3.835519000
C	1.642597000	1.183704000	1.681082000
H	1.620831000	1.172702000	0.589196000
C	1.217852000	2.577185000	2.156789000
H	0.265133000	2.843799000	1.690426000
H	1.085823000	2.616682000	3.243660000
H	1.963957000	3.323582000	1.869548000
C	-0.270672000	-0.400143000	1.211289000
C	-0.539886000	-1.175473000	3.345689000
C	0.472057000	-0.277004000	3.370345000
H	-0.954228000	-1.783449000	4.133261000
H	1.114529000	0.040739000	4.176184000
N	0.621970000	0.185666000	2.072102000
N	-0.988856000	-1.238517000	2.030037000
C	-6.782535000	0.725841000	4.565245000
C	-5.472598000	0.370605000	4.328313000
C	-5.130387000	-0.463101000	3.228394000
C	-6.175197000	-0.930197000	2.367277000
C	-7.515658000	-0.544343000	2.636461000

C	-7.814539000	0.265012000	3.710705000
H	-3.006879000	-0.462795000	3.612651000
H	-7.029370000	1.361757000	5.411223000
H	-4.679768000	0.723111000	4.984361000
C	-3.785658000	-0.840618000	2.955896000
C	-5.823936000	-1.758815000	1.267563000
H	-8.303838000	-0.899349000	1.976722000
H	-8.843994000	0.551503000	3.908245000
C	-4.514610000	-2.105329000	1.034612000
C	-3.465548000	-1.647474000	1.883383000
H	-6.608790000	-2.107002000	0.600606000
H	-4.262395000	-2.731443000	0.182358000
C	-2.055073000	-2.141975000	1.572383000
H	-1.943153000	-2.177984000	0.489548000
C	-1.838198000	-3.566630000	2.116098000
H	-0.833573000	-3.923060000	1.872058000
H	-1.989177000	-3.613462000	3.200319000
H	-2.559386000	-4.245639000	1.653502000
C	6.903126000	-2.157071000	1.481048000
C	6.732545000	3.589923000	0.726771000
C	5.752156000	2.936603000	0.013880000
C	4.531912000	3.590962000	-0.306955000
C	4.333392000	4.940530000	0.129513000
C	5.364989000	5.586977000	0.861947000
C	6.538803000	4.927780000	1.152401000
H	3.664694000	1.923986000	-1.377098000
H	7.659262000	3.077510000	0.969497000
H	5.894715000	1.907423000	-0.304684000
C	3.500278000	2.946257000	-1.041987000
C	3.104041000	5.578778000	-0.188171000
H	5.211859000	6.612784000	1.189599000
H	7.321895000	5.431613000	1.712736000
C	2.126525000	4.925636000	-0.903383000
C	2.313744000	3.586012000	-1.344982000
H	2.944078000	6.602211000	0.143842000
H	1.196277000	5.441673000	-1.119695000
C	1.285619000	2.860562000	-2.208714000
H	1.512822000	1.796253000	-2.176234000
C	1.351102000	3.324652000	-3.673162000
H	0.622428000	2.780944000	-4.283629000
H	1.164270000	4.399584000	-3.772407000
H	2.353451000	3.133002000	-4.067465000
C	-0.706345000	1.937448000	-0.999775000

C	-2.111738000	3.722123000	-1.272378000
C	-0.940572000	4.037503000	-1.876083000
H	-3.038510000	4.269520000	-1.199997000
H	-0.659596000	4.918285000	-2.430238000
N	-0.094314000	2.948952000	-1.695677000
N	-1.949657000	2.450953000	-0.742625000
C	-9.032505000	1.547241000	-1.767516000
C	-7.968250000	1.799113000	-0.929256000
C	-6.635695000	1.538903000	-1.347818000
C	-6.414327000	1.005053000	-2.657060000
C	-7.531391000	0.757613000	-3.498137000
C	-8.812224000	1.022875000	-3.064822000
H	-5.714495000	2.189941000	0.486579000
H	-10.047210000	1.750364000	-1.435612000
H	-8.134430000	2.197042000	0.069152000
C	-5.518115000	1.791967000	-0.504096000
C	-5.076430000	0.741300000	-3.061046000
H	-7.358531000	0.352847000	-4.492665000
H	-9.659377000	0.829003000	-3.717194000
C	-4.020008000	0.991852000	-2.219021000
C	-4.228332000	1.527441000	-0.913895000
H	-4.897969000	0.334202000	-4.053662000
H	-3.005015000	0.786320000	-2.548069000
C	-3.015105000	1.739461000	-0.010642000
H	-2.575311000	0.761906000	0.210138000
C	-3.297964000	2.451233000	1.316081000
H	-2.364695000	2.554302000	1.877790000
H	-3.719297000	3.451051000	1.162037000
H	-3.996497000	1.871449000	1.923809000
C	4.972092000	-0.717426000	1.811253000
C	5.655975000	-1.730827000	1.083278000
C	0.927908000	-6.238374000	0.234211000
C	1.331258000	-4.961381000	-0.120129000
C	0.525445000	-4.108238000	-0.923822000
C	-0.801989000	-4.587039000	-1.228471000
C	-1.166960000	-5.910171000	-0.894788000
C	-0.316029000	-6.743176000	-0.190868000
H	1.571907000	-6.844246000	0.867221000
H	2.255195000	-4.573924000	0.292242000
C	0.888380000	-2.769482000	-1.341482000
C	-1.750995000	-3.687751000	-1.813191000
H	-2.160516000	-6.255600000	-1.175666000
H	-0.620766000	-7.752613000	0.071098000

C	-1.481788000	-2.346810000	-1.873202000
C	-0.204549000	-1.849928000	-1.430736000
H	-2.716513000	-4.075322000	-2.133681000
H	-2.234017000	-1.643484000	-2.220925000
C	6.161750000	-3.521776000	-2.536122000
C	5.740303000	-2.289983000	-2.985233000
C	4.399347000	-1.853906000	-2.820152000
C	3.452885000	-2.678761000	-2.106716000
C	3.919009000	-3.964038000	-1.719679000
C	5.223653000	-4.375216000	-1.921142000
H	4.698528000	-0.069398000	-4.013563000
H	7.189861000	-3.842967000	-2.681338000
H	6.429913000	-1.630084000	-3.507690000
C	3.979525000	-0.641961000	-3.432881000
C	2.101073000	-2.182020000	-1.889923000
H	3.227982000	-4.669021000	-1.289794000
H	5.519399000	-5.373951000	-1.609582000
C	1.758620000	-0.967867000	-2.549507000
C	2.669054000	-0.243146000	-3.349722000
H	2.306885000	0.621497000	-3.895936000
O	0.469775000	-0.560282000	-2.512872000

87

INT, $E_{(\text{SCF Done})}$ = -2167.399900 a.u.

C	7.458489000	1.740034000	1.949653000
C	6.236282000	1.220091000	1.588492000
C	5.635722000	1.567432000	0.346935000
C	6.326405000	2.469661000	-0.526807000
C	7.587564000	2.987683000	-0.124778000
C	8.141173000	2.632420000	1.084524000
H	3.854588000	0.364098000	0.609197000
H	7.906451000	1.468034000	2.901570000
H	5.709954000	0.535756000	2.249735000
C	4.378441000	1.048554000	-0.055734000
C	5.721516000	2.806520000	-1.765737000
H	8.109245000	3.671276000	-0.790695000
H	9.105740000	3.035106000	1.381650000
C	4.498497000	2.284347000	-2.125145000
C	3.803052000	1.392883000	-1.264463000
H	6.243328000	3.485267000	-2.436564000
H	4.069936000	2.552859000	-3.086289000
C	2.434278000	0.816200000	-1.615116000
H	2.358453000	-0.176557000	-1.159739000
C	2.142763000	0.679231000	-3.113172000

H	1.186934000	0.165171000	-3.253157000
H	2.079683000	1.649271000	-3.618114000
H	2.928626000	0.091541000	-3.598550000
C	0.507348000	1.017048000	-0.030332000
C	0.024142000	3.222415000	-0.347645000
C	1.077828000	2.919836000	-1.149467000
H	-0.514327000	4.147659000	-0.213840000
H	1.646697000	3.540542000	-1.823978000
N	1.358932000	1.577562000	-0.945741000
N	-0.309006000	2.058040000	0.326260000
C	-6.775007000	4.819693000	-0.140761000
C	-5.563753000	4.655419000	0.494909000
C	-4.766546000	3.503839000	0.256929000
C	-5.243173000	2.509943000	-0.657160000
C	-6.496481000	2.707605000	-1.295060000
C	-7.246168000	3.836170000	-1.044566000
H	-3.175328000	4.066481000	1.599633000
H	-7.374782000	5.705517000	0.050390000
H	-5.201080000	5.408843000	1.190730000
C	-3.512169000	3.306522000	0.899429000
C	-4.441477000	1.357250000	-0.885781000
H	-6.855563000	1.949644000	-1.987390000
H	-8.203463000	3.975786000	-1.539557000
C	-3.233173000	1.200546000	-0.251920000
C	-2.748068000	2.184468000	0.657234000
H	-4.803359000	0.587772000	-1.563530000
H	-2.633715000	0.311338000	-0.429563000
C	-1.397422000	1.931416000	1.317418000
H	-1.352109000	0.873742000	1.601976000
C	-1.092062000	2.776733000	2.556958000
H	-0.134429000	2.464379000	2.984514000
H	-1.026449000	3.845955000	2.327280000
H	-1.872336000	2.637133000	3.311847000
Ni	0.417888000	-0.794772000	0.538640000
C	-3.697917000	-2.996396000	-2.292997000
C	-2.555823000	-2.881411000	-1.527204000
C	-2.608263000	-2.910229000	-0.107150000
C	-3.907386000	-2.966748000	0.514830000
C	-5.058226000	-3.113188000	-0.305038000
C	-4.962940000	-3.141366000	-1.678845000
H	-3.624096000	-2.962373000	-3.376855000
H	-1.602259000	-2.717708000	-2.013991000
C	-1.463537000	-2.788972000	0.747092000

C	-4.039578000	-2.819689000	1.925868000
H	-6.030152000	-3.180299000	0.178817000
H	-5.855596000	-3.247795000	-2.289258000
C	-2.945646000	-2.573118000	2.724711000
C	-1.685681000	-2.549762000	2.108062000
H	-5.033797000	-2.867495000	2.362063000
H	-3.032848000	-2.411547000	3.794305000
C	2.911956000	-4.681422000	-1.951668000
C	3.288379000	-4.046769000	-0.781366000
C	2.337137000	-3.442586000	0.072057000
C	0.949875000	-3.436047000	-0.315207000
C	0.594091000	-4.148231000	-1.482935000
C	1.549400000	-4.751234000	-2.287062000
H	3.812616000	-2.942580000	1.587751000
H	3.659320000	-5.144700000	-2.590005000
H	4.334860000	-4.025056000	-0.484558000
C	2.755227000	-2.902581000	1.338933000
C	0.004114000	-2.761016000	0.570616000
H	-0.450145000	-4.252159000	-1.744553000
H	1.235609000	-5.287675000	-3.178866000
C	0.489209000	-2.324139000	1.847694000
C	1.856151000	-2.394150000	2.237052000
H	2.151246000	-2.058827000	3.226784000
O	-0.534882000	-2.280431000	2.797194000

87

TS1′, $E_{(SCF\ Done)}$ = -2167.363375 a.u., IF = -325.84 cm^{-1}

C	4.793857000	4.530028000	0.618490000
C	3.976710000	3.423789000	0.551836000
C	3.105602000	3.235483000	-0.556672000
C	3.094990000	4.210799000	-1.606426000
C	3.951476000	5.340316000	-1.505768000
C	4.781004000	5.497200000	-0.418074000
H	2.264278000	1.370732000	0.142561000
H	5.456076000	4.664720000	1.469424000
H	3.988629000	2.679759000	1.344285000
C	2.249184000	2.107533000	-0.657319000
C	2.230719000	4.003177000	-2.713687000
H	3.941622000	6.078957000	-2.304084000
H	5.432281000	6.364640000	-0.350889000
C	1.416975000	2.894398000	-2.782450000
C	1.410429000	1.925788000	-1.740700000
H	2.225768000	4.734073000	-3.519209000
H	0.783202000	2.756728000	-3.654069000

C	0.503451000	0.701163000	-1.778424000
H	0.951336000	-0.072148000	-1.149563000
C	0.274297000	0.107856000	-3.173999000
H	-0.305528000	-0.816324000	-3.088262000
H	-0.273592000	0.791627000	-3.831167000
H	1.235082000	-0.127163000	-3.642718000
C	-1.349307000	0.159016000	-0.177434000
C	-2.730780000	1.911701000	-0.626817000
C	-1.627475000	2.052705000	-1.405283000
H	-3.620580000	2.516171000	-0.546118000
H	-1.360595000	2.821756000	-2.112413000
N	-0.796786000	0.979588000	-1.120478000
N	-2.547660000	0.752530000	0.112432000
C	-9.645902000	1.047303000	0.348968000
C	-8.408582000	1.267734000	0.913516000
C	-7.264795000	0.559568000	0.457523000
C	-7.417108000	-0.391274000	-0.601466000
C	-8.705188000	-0.596761000	-1.162309000
C	-9.795787000	0.106248000	-0.698774000
H	-5.883356000	1.487809000	1.827830000
H	-10.513189000	1.595199000	0.707130000
H	-8.291841000	1.989063000	1.719135000
C	-5.974052000	0.766978000	1.020268000
C	-6.265751000	-1.096852000	-1.049101000
H	-8.816994000	-1.320790000	-1.965985000
H	-10.776742000	-0.059605000	-1.135635000
C	-5.034732000	-0.872077000	-0.483689000
C	-4.870383000	0.073972000	0.569628000
H	-6.375334000	-1.825158000	-1.849212000
H	-4.164379000	-1.418929000	-0.838294000
C	-3.474185000	0.260372000	1.152491000
H	-3.071519000	-0.727889000	1.406977000
C	-3.384272000	1.144333000	2.400373000
H	-2.347859000	1.172421000	2.749237000
H	-3.702718000	2.172842000	2.198903000
H	-4.009956000	0.739728000	3.202378000
Ni	-0.614304000	-1.412537000	0.569333000
C	5.639601000	-0.211832000	2.318308000
C	4.670835000	-0.896827000	1.611063000
C	3.289032000	-0.783267000	1.931800000
C	2.954325000	-0.013691000	3.103928000
C	3.969046000	0.695521000	3.792263000
C	5.291584000	0.619610000	3.403710000

H	6.683553000	-0.323738000	2.036462000
H	4.977715000	-1.551807000	0.806976000
C	2.225888000	-1.450267000	1.224656000
C	1.623673000	-0.049792000	3.624274000
H	3.685330000	1.286118000	4.661079000
H	6.059223000	1.164313000	3.946491000
C	0.634110000	-0.772683000	3.014231000
C	0.911139000	-1.364550000	1.749360000
H	1.413704000	0.495381000	4.542140000
H	-0.363496000	-0.832762000	3.437003000
C	4.795685000	-3.506115000	-3.083259000
C	3.843202000	-4.394521000	-2.634238000
C	2.973391000	-4.066364000	-1.560925000
C	3.137626000	-2.805253000	-0.886375000
C	4.089567000	-1.891600000	-1.416436000
C	4.897013000	-2.230799000	-2.483294000
H	1.818499000	-5.894521000	-1.703525000
H	5.446273000	-3.770981000	-3.912333000
H	3.725071000	-5.362688000	-3.116193000
C	1.913409000	-4.942257000	-1.187801000
C	2.259562000	-2.500167000	0.208417000
H	4.153428000	-0.894358000	-0.995630000
H	5.607217000	-1.505394000	-2.871573000
C	1.144372000	-3.332089000	0.405249000
C	0.985350000	-4.573656000	-0.241515000
H	0.141378000	-5.206376000	0.014559000
O	0.245857000	-2.893073000	1.345076000

87

A′, $E_{(\text{SCF Done})}$ = -2167.403450 a.u.

C	4.000605000	5.598004000	0.995412000
C	3.369157000	4.387390000	0.820610000
C	2.500068000	4.176476000	-0.285020000
C	2.292378000	5.243329000	-1.218680000
C	2.959935000	6.480458000	-1.009522000
C	3.793877000	6.654219000	0.072116000
H	1.993979000	2.137297000	0.224014000
H	4.663457000	5.749008000	1.842997000
H	3.525701000	3.572899000	1.523581000
C	1.835241000	2.940345000	-0.492372000
C	1.429075000	5.016533000	-2.322674000
H	2.802140000	7.288400000	-1.720211000
H	4.299950000	7.604048000	0.222149000
C	0.801207000	3.802931000	-2.496944000

C	0.994475000	2.740728000	-1.571089000
H	1.274507000	5.818230000	-3.041145000
H	0.161726000	3.657303000	-3.362734000
C	0.299272000	1.393207000	-1.724954000
H	0.929256000	0.630251000	-1.262328000
C	0.009480000	0.968714000	-3.169591000
H	-0.384792000	-0.052188000	-3.180726000
H	-0.725086000	1.621158000	-3.653486000
H	0.932027000	0.988644000	-3.758202000
C	-1.343171000	0.276335000	-0.185210000
C	-2.929823000	1.896751000	-0.110658000
C	-1.915405000	2.351720000	-0.890901000
H	-3.868968000	2.350549000	0.163810000
H	-1.788190000	3.291802000	-1.403483000
N	-0.955405000	1.352232000	-0.924818000
N	-2.564448000	0.627626000	0.312514000
C	-9.601862000	0.083674000	0.718753000
C	-8.369948000	0.322754000	1.287200000
C	-7.184141000	-0.181964000	0.689834000
C	-7.288095000	-0.946712000	-0.515729000
C	-8.572015000	-1.176987000	-1.076592000
C	-9.703953000	-0.673138000	-0.474097000
H	-5.843347000	0.623806000	2.173247000
H	-10.501802000	0.474578000	1.185441000
H	-8.289928000	0.902004000	2.204207000
C	-5.897876000	0.049207000	1.252945000
C	-6.095156000	-1.451140000	-1.103676000
H	-8.647200000	-1.759758000	-1.991526000
H	-10.681317000	-0.855795000	-0.912113000
C	-4.869561000	-1.209045000	-0.533346000
C	-4.753417000	-0.446028000	0.664156000
H	-6.167425000	-2.040581000	-2.014505000
H	-3.968949000	-1.606057000	-0.996188000
C	-3.359218000	-0.216347000	1.236606000
H	-2.828012000	-1.175291000	1.242055000
C	-3.306470000	0.357055000	2.655937000
H	-2.266677000	0.414024000	2.989968000
H	-3.738509000	1.361942000	2.709630000
H	-3.853594000	-0.291940000	3.346522000
Ni	-0.453247000	-1.403075000	0.032881000
C	5.298624000	-2.140356000	3.045969000
C	4.354275000	-2.322608000	2.059611000
C	3.248552000	-1.435250000	1.909238000

C	3.118069000	-0.382753000	2.877872000
C	4.127637000	-0.199841000	3.862932000
C	5.203112000	-1.052531000	3.945478000
H	6.121239000	-2.844890000	3.137019000
H	4.441311000	-3.170538000	1.392690000
C	2.234471000	-1.607335000	0.885790000
C	1.954872000	0.425901000	2.868308000
H	4.015167000	0.618481000	4.571211000
H	5.961887000	-0.909878000	4.710523000
C	0.932248000	0.155693000	1.985541000
C	1.051279000	-0.871072000	1.015217000
H	1.858885000	1.226885000	3.599005000
H	0.024175000	0.748572000	2.032783000
C	5.926207000	-3.174934000	-2.571970000
C	4.881342000	-4.068862000	-2.650703000
C	3.698107000	-3.890367000	-1.888352000
C	3.593279000	-2.783882000	-0.976534000
C	4.681958000	-1.862963000	-0.950233000
C	5.808467000	-2.050638000	-1.722977000
H	2.681401000	-5.621721000	-2.704758000
H	6.823468000	-3.319117000	-3.167855000
H	4.938142000	-4.925620000	-3.319443000
C	2.588981000	-4.767730000	-2.036830000
C	2.394136000	-2.611402000	-0.198757000
H	4.614241000	-0.986641000	-0.315965000
H	6.612732000	-1.320023000	-1.681378000
C	1.268281000	-3.392956000	-0.526568000
C	1.405409000	-4.516501000	-1.395479000
H	0.531164000	-5.143774000	-1.544737000
O	0.061666000	-3.119682000	-0.046871000

93

B′, $E_{\text{(SCF Done)}}$ = -4981.535151 a.u.

C	5.120383000	4.873181000	1.435502000
C	4.305743000	3.806155000	1.130417000
C	3.462354000	3.844267000	-0.014166000
C	3.472617000	5.009899000	-0.847130000
C	4.325783000	6.093433000	-0.504003000
C	5.130686000	6.026864000	0.611100000
H	2.606954000	1.882042000	0.282941000
H	5.761471000	4.835026000	2.311952000
H	4.293473000	2.919528000	1.759696000
C	2.612798000	2.760449000	-0.356295000
C	2.629954000	5.030955000	-1.989608000

H	4.333217000	6.976803000	-1.138342000
H	5.779665000	6.860887000	0.864297000
C	1.818190000	3.960794000	-2.294685000
C	1.795119000	2.801918000	-1.469905000
H	2.639022000	5.908421000	-2.632113000
H	1.199370000	4.004632000	-3.186247000
C	0.897964000	1.606847000	-1.774163000
H	1.370193000	0.715170000	-1.356565000
C	0.632751000	1.367938000	-3.266146000
H	0.077701000	0.435006000	-3.403813000
H	0.046414000	2.176264000	-3.715575000
H	1.581495000	1.286932000	-3.806014000
C	-1.017115000	0.659645000	-0.434364000
C	-2.283221000	2.532360000	-0.265023000
C	-1.157228000	2.862043000	-0.943103000
H	-3.139297000	3.123336000	0.017488000
H	-0.827711000	3.805039000	-1.348304000
N	-0.392676000	1.707477000	-1.039123000
N	-2.191236000	1.176948000	0.031222000
C	-9.098344000	2.603652000	1.093912000
C	-7.848977000	2.268595000	1.567952000
C	-6.865400000	1.723495000	0.700084000
C	-7.192274000	1.526859000	-0.679135000
C	-8.488469000	1.881182000	-1.137611000
C	-9.421202000	2.408034000	-0.271265000
H	-5.346314000	1.522178000	2.213056000
H	-9.842888000	3.018540000	1.767692000
H	-7.600112000	2.415819000	2.616330000
C	-5.566697000	1.368721000	1.161132000
C	-6.198598000	0.981096000	-1.537452000
H	-8.733697000	1.728226000	-2.185884000
H	-10.410569000	2.674762000	-0.632504000
C	-4.954587000	0.649047000	-1.059278000
C	-4.617065000	0.837459000	0.313141000
H	-6.439325000	0.824925000	-2.586277000
H	-4.210302000	0.238611000	-1.735691000
C	-3.225603000	0.425298000	0.789197000
H	-3.062004000	-0.617714000	0.503789000
C	-2.991934000	0.546689000	2.298260000
H	-2.001398000	0.163691000	2.553642000
H	-3.057650000	1.586171000	2.637517000
H	-3.734051000	-0.045114000	2.843029000
Ni	-0.342620000	-1.111748000	-0.252913000

C	5.278350000	-1.632473000	3.169136000
C	4.406140000	-1.812729000	2.118217000
C	3.238632000	-1.006943000	1.969017000
C	2.975709000	-0.038901000	2.996668000
C	3.909605000	0.142616000	4.054445000
C	5.043664000	-0.630364000	4.140445000
H	6.153003000	-2.271887000	3.255567000
H	4.602980000	-2.595291000	1.396204000
C	2.296154000	-1.166567000	0.882728000
C	1.763850000	0.692149000	2.952749000
H	3.692777000	0.893519000	4.811354000
H	5.745035000	-0.490766000	4.958801000
C	0.833604000	0.438487000	1.970055000
C	1.061627000	-0.504795000	0.930549000
H	1.567638000	1.436235000	3.722679000
H	-0.093922000	1.001412000	1.987750000
C	6.180316000	-2.135733000	-2.569971000
C	5.255678000	-3.144909000	-2.711240000
C	4.052502000	-3.151036000	-1.955825000
C	3.807813000	-2.107687000	-0.997801000
C	4.779223000	-1.068930000	-0.897840000
C	5.925641000	-1.080558000	-1.662050000
H	3.266840000	-4.961544000	-2.851074000
H	7.093390000	-2.138669000	-3.158990000
H	5.424541000	-3.953286000	-3.419495000
C	3.069488000	-4.156850000	-2.146799000
C	2.593318000	-2.116987000	-0.228243000
H	4.603207000	-0.249175000	-0.211071000
H	6.640728000	-0.267393000	-1.567568000
C	1.607290000	-3.045802000	-0.565340000
C	1.868202000	-4.094481000	-1.485354000
H	1.096790000	-4.842191000	-1.655967000
O	0.370896000	-2.937913000	-0.026413000
C	-1.837631000	-1.876306000	-1.665099000
H	-1.889312000	-2.831062000	-2.237478000
H	-1.300704000	-1.229605000	-2.369536000
H	-2.869917000	-1.514809000	-1.618413000
Mg	-1.457281000	-3.458800000	-0.181942000
Br	-2.946760000	-5.149321000	0.557421000

93

TS2′, $E_{\text{(SCF Done)}}$ = -4981.508901 a.u., IF = -114.89 cm^{-1}

C	4.767092000	6.053319000	1.123270000
C	4.168689000	4.825698000	0.946717000

C	3.077957000	4.674210000	0.047836000
C	2.613748000	5.818503000	-0.677778000
C	3.253839000	7.070422000	-0.474382000
C	4.305446000	7.185994000	0.407182000
H	2.798844000	2.559562000	0.402652000
H	5.600067000	6.158561000	1.813070000
H	4.523777000	3.951239000	1.484777000
C	2.435654000	3.422137000	-0.150859000
C	1.528933000	5.652768000	-1.579818000
H	2.899558000	7.936908000	-1.027893000
H	4.788002000	8.148221000	0.555753000
C	0.931817000	4.425255000	-1.753410000
C	1.377415000	3.283193000	-1.027578000
H	1.176573000	6.514969000	-2.141265000
H	0.109824000	4.330698000	-2.456906000
C	0.715605000	1.923165000	-1.201792000
H	1.408852000	1.157388000	-0.849945000
C	0.338469000	1.605353000	-2.656131000
H	0.010478000	0.565448000	-2.746844000
H	-0.464538000	2.254696000	-3.020337000
H	1.215167000	1.734994000	-3.297316000
C	-1.106187000	0.612429000	-0.027606000
C	-2.247903000	2.343625000	0.887196000
C	-1.179403000	2.862183000	0.233635000
H	-3.043839000	2.825474000	1.431918000
H	-0.852658000	3.881976000	0.120869000
N	-0.488135000	1.795845000	-0.320458000
N	-2.192547000	0.970426000	0.717532000
C	-9.103872000	2.211700000	1.309860000
C	-7.878145000	1.878428000	1.842971000
C	-6.857502000	1.323106000	1.025726000
C	-7.122591000	1.112533000	-0.365130000
C	-8.396335000	1.464883000	-0.884487000
C	-9.365480000	2.003021000	-0.066518000
H	-5.407200000	1.129825000	2.607569000
H	-9.876754000	2.635421000	1.945148000
H	-7.676372000	2.036103000	2.899991000
C	-5.581522000	0.970376000	1.547256000
C	-6.096158000	0.550022000	-1.172835000
H	-8.594720000	1.301616000	-1.941048000
H	-10.336653000	2.268803000	-0.474711000
C	-4.875014000	0.220295000	-0.636833000
C	-4.597534000	0.432035000	0.744384000

H	-6.294782000	0.374587000	-2.227412000
H	-4.107487000	-0.219829000	-1.268181000
C	-3.215618000	0.048393000	1.267572000
H	-2.955748000	-0.923100000	0.839265000
C	-3.087123000	-0.025308000	2.792291000
H	-2.080455000	-0.352257000	3.064493000
H	-3.266701000	0.946406000	3.263795000
H	-3.808645000	-0.741325000	3.198329000
Ni	-0.664953000	-1.094512000	-0.757303000
C	4.123092000	-3.938920000	2.744217000
C	3.600796000	-3.093645000	1.789844000
C	2.220271000	-2.740186000	1.786494000
C	1.382287000	-3.345958000	2.781043000
C	1.954905000	-4.196176000	3.765376000
C	3.299544000	-4.484322000	3.756698000
H	5.178614000	-4.195529000	2.712469000
H	4.248170000	-2.700077000	1.016390000
C	1.642211000	-1.845385000	0.802896000
C	-0.015653000	-3.120880000	2.732868000
H	1.300021000	-4.632993000	4.515843000
H	3.725753000	-5.143804000	4.507758000
C	-0.564536000	-2.352353000	1.732345000
C	0.252738000	-1.724548000	0.753021000
H	-0.654509000	-3.596453000	3.473962000
H	-1.643219000	-2.260046000	1.678909000
C	5.862945000	1.292973000	0.826117000
C	5.622689000	0.959857000	-0.488970000
C	4.512184000	0.155067000	-0.853941000
C	3.629293000	-0.345815000	0.163770000
C	3.890582000	0.048755000	1.509550000
C	4.974431000	0.839712000	1.830242000
H	4.920017000	0.202377000	-2.981411000
H	6.721269000	1.904056000	1.091603000
H	6.283441000	1.315482000	-1.276604000
C	4.241367000	-0.161475000	-2.213050000
C	2.518095000	-1.179353000	-0.202339000
H	3.222494000	-0.287263000	2.294641000
H	5.148624000	1.113527000	2.868016000
C	2.237161000	-1.386864000	-1.563539000
C	3.134844000	-0.893611000	-2.557984000
H	2.906565000	-1.121437000	-3.595125000
O	1.126257000	-1.998793000	-1.962228000
C	-1.918191000	-2.353204000	-2.036680000

H	-1.981517000	-2.816774000	-3.038267000
H	-2.092853000	-1.277451000	-2.279625000
H	-2.802962000	-2.700454000	-1.491172000
Mg	-0.043755000	-3.498834000	-1.780340000
Br	0.177771000	-5.871691000	-1.929673000

93

C′, $E_{(\text{SCF Done})}$ = -4981.535983 a.u.

C	7.463306000	-4.094297000	0.374787000
C	6.104411000	-3.873789000	0.418680000
C	5.521315000	-2.792515000	-0.294891000
C	6.369824000	-1.930690000	-1.062020000
C	7.766912000	-2.184774000	-1.087282000
C	8.302077000	-3.242417000	-0.385458000
H	3.482933000	-3.187271000	0.322193000
H	7.898076000	-4.924875000	0.923917000
H	5.458017000	-4.525866000	1.001029000
C	4.123057000	-2.536467000	-0.268990000
C	5.777338000	-0.848193000	-1.768920000
H	8.408622000	-1.529597000	-1.671627000
H	9.372299000	-3.427927000	-0.412474000
C	4.420441000	-0.631429000	-1.727019000
C	3.568058000	-1.485223000	-0.969444000
H	6.417980000	-0.189303000	-2.350289000
H	3.993596000	0.204981000	-2.274129000
C	2.062449000	-1.274322000	-0.950363000
H	1.608408000	-2.087814000	-0.379987000
C	1.474654000	-1.294707000	-2.366736000
H	0.376703000	-1.328555000	-2.342840000
H	1.802618000	-0.432848000	-2.957688000
H	1.802326000	-2.205937000	-2.876089000
C	0.509875000	0.635807000	-0.314428000
C	1.863504000	1.671935000	1.179316000
C	2.540840000	0.602912000	0.691987000
H	2.160164000	2.431171000	1.885089000
H	3.528663000	0.236600000	0.913867000
N	1.711861000	-0.011212000	-0.234232000
N	0.629618000	1.679888000	0.552969000
C	1.704461000	8.623848000	1.293745000
C	1.198823000	7.420930000	1.735594000
C	0.938420000	6.363451000	0.823610000
C	1.209096000	6.567325000	-0.567198000
C	1.728722000	7.818634000	-0.991784000
C	1.971890000	8.824743000	-0.082573000

H	0.210346000	4.981005000	2.308990000
H	1.899091000	9.425215000	2.001135000
H	0.991251000	7.265783000	2.791830000
C	0.417323000	5.109844000	1.250340000
C	0.940421000	5.503867000	-1.472649000
H	1.931447000	7.970260000	-2.049307000
H	2.369483000	9.778345000	-0.418729000
C	0.437204000	4.306411000	-1.025864000
C	0.168310000	4.089932000	0.356380000
H	1.133779000	5.655409000	-2.531998000
H	0.234046000	3.505224000	-1.732902000
C	-0.390570000	2.732785000	0.774893000
H	-1.197596000	2.471767000	0.087998000
C	-0.938018000	2.651786000	2.201633000
H	-1.377929000	1.665893000	2.369490000
H	-0.159104000	2.812972000	2.954319000
H	-1.718148000	3.404914000	2.347522000
Ni	-0.969728000	0.201763000	-1.519984000
C	-4.525663000	-2.419467000	3.468432000
C	-3.515930000	-2.361171000	2.532590000
C	-3.382853000	-1.242786000	1.662303000
C	-4.353377000	-0.192213000	1.768564000
C	-5.377464000	-0.278356000	2.749360000
C	-5.461732000	-1.365642000	3.588197000
H	-4.607696000	-3.287680000	4.116686000
H	-2.811775000	-3.179648000	2.444793000
C	-2.317731000	-1.147293000	0.683582000
C	-4.293822000	0.897267000	0.861466000
H	-6.103873000	0.528238000	2.813578000
H	-6.252519000	-1.425003000	4.330992000
C	-3.324102000	0.945489000	-0.111832000
C	-2.311622000	-0.060755000	-0.201186000
H	-5.052660000	1.674275000	0.918883000
H	-3.337914000	1.756130000	-0.835142000
C	1.532045000	-2.896211000	3.773167000
C	1.458792000	-3.770983000	2.710185000
C	0.536970000	-3.570248000	1.650540000
C	-0.353112000	-2.440924000	1.681389000
C	-0.243782000	-1.557656000	2.795894000
C	0.668994000	-1.776747000	3.806411000
H	1.121352000	-5.331718000	0.527769000
H	2.241366000	-3.063736000	4.578959000
H	2.109332000	-4.643045000	2.669398000

C	0.464662000	-4.463590000	0.543685000
C	-1.290570000	-2.238826000	0.615978000
H	-0.902171000	-0.697042000	2.848308000
H	0.721472000	-1.082018000	4.641269000
C	-1.307959000	-3.120679000	-0.488586000
C	-0.410107000	-4.241966000	-0.485631000
H	-0.471758000	-4.918771000	-1.333062000
O	-2.116631000	-2.981823000	-1.514072000
C	-1.997478000	0.152058000	-3.197169000
H	-2.154297000	-0.711016000	-3.868333000
H	-1.120879000	0.671842000	-3.639281000
H	-2.859065000	0.819131000	-3.317669000
Mg	-3.170863000	-1.450246000	-1.936238000
Br	-5.431153000	-1.390290000	-2.747622000

93

TS3′, $E_{(\mathrm{SCF\ Done})}$ = -4981.507303 a.u., IF = -369.84 cm^{-1}

C	-7.971539000	1.072326000	1.214007000
C	-6.647877000	1.040254000	0.837010000
C	-6.175910000	0.052335000	-0.070382000
C	-7.101458000	-0.910453000	-0.589262000
C	-8.461181000	-0.849442000	-0.180405000
C	-8.886870000	0.118949000	0.700763000
H	-4.122931000	0.727815000	-0.075407000
H	-8.321701000	1.831312000	1.908362000
H	-5.939664000	1.765824000	1.227513000
C	-4.817584000	-0.005400000	-0.479924000
C	-6.620619000	-1.887359000	-1.499915000
H	-9.161252000	-1.581026000	-0.577156000
H	-9.928932000	0.156849000	1.006498000
C	-5.297459000	-1.913482000	-1.880199000
C	-4.368893000	-0.965430000	-1.366966000
H	-7.318298000	-2.617450000	-1.903650000
H	-4.966319000	-2.665374000	-2.590119000
C	-2.897813000	-0.984755000	-1.765107000
H	-2.513279000	0.037110000	-1.688996000
C	-2.625929000	-1.497160000	-3.186148000
H	-1.564354000	-1.375152000	-3.423095000
H	-2.872143000	-2.558734000	-3.291486000
H	-3.214561000	-0.929327000	-3.914134000
C	-0.795029000	-1.419836000	-0.466161000
C	-1.426879000	-3.337982000	0.586212000
C	-2.481819000	-2.920116000	-0.157431000
H	-1.311762000	-4.201429000	1.222253000

H	-3.470515000	-3.334056000	-0.273601000
N	-2.084711000	-1.749365000	-0.786017000
N	-0.410298000	-2.414950000	0.389091000
C	3.858887000	-8.033975000	1.296533000
C	3.187281000	-6.936959000	1.790299000
C	2.760738000	-5.895082000	0.924161000
C	3.040675000	-6.001747000	-0.475485000
C	3.735185000	-7.144004000	-0.953837000
C	4.135582000	-8.138644000	-0.088489000
H	1.870832000	-4.687949000	2.472250000
H	4.180260000	-8.824005000	1.969747000
H	2.975153000	-6.854432000	2.853871000
C	2.064554000	-4.750551000	1.405188000
C	2.611443000	-4.951573000	-1.333811000
H	3.946920000	-7.220728000	-2.017735000
H	4.666747000	-9.008085000	-0.466007000
C	1.941144000	-3.860964000	-0.836193000
C	1.654665000	-3.745266000	0.555379000
H	2.826291000	-5.023116000	-2.397536000
H	1.617126000	-3.066637000	-1.504554000
C	0.917679000	-2.496207000	1.031150000
H	1.448616000	-1.621189000	0.645643000
C	0.787457000	-2.342899000	2.549371000
H	0.269960000	-1.407567000	2.780422000
H	0.221997000	-3.163945000	3.003229000
H	1.777866000	-2.311850000	3.013863000
Ni	0.226641000	0.037188000	-1.205139000
C	3.242813000	3.910977000	3.136408000
C	2.270017000	3.614051000	2.203617000
C	2.427027000	2.518905000	1.299639000
C	3.634617000	1.742796000	1.394339000
C	4.609950000	2.076105000	2.365750000
C	4.422998000	3.138439000	3.224840000
H	3.095132000	4.746811000	3.814835000
H	1.359454000	4.201296000	2.155323000
C	1.414662000	2.183927000	0.309905000
C	3.825762000	0.674943000	0.470955000
H	5.517664000	1.479639000	2.414426000
H	5.179851000	3.386075000	3.963345000
C	2.888311000	0.388575000	-0.485996000
C	1.623043000	1.082251000	-0.564570000
H	4.753243000	0.108715000	0.512769000
H	3.091091000	-0.397252000	-1.207820000

C	-3.256150000	2.055256000	2.679902000
C	-3.269504000	3.162147000	1.856412000
C	-2.154202000	3.488454000	1.042293000
C	-0.988017000	2.650123000	1.070256000
C	-1.011634000	1.513545000	1.928835000
C	-2.110480000	1.225318000	2.710403000
H	-3.032026000	5.275883000	0.187994000
H	-4.111686000	1.824643000	3.308933000
H	-4.136370000	3.820123000	1.831682000
C	-2.144997000	4.646110000	0.214952000
C	0.149700000	2.998052000	0.277356000
H	-0.136498000	0.873415000	1.965971000
H	-2.095597000	0.355480000	3.362745000
C	0.144278000	4.179673000	-0.485147000
C	-1.039352000	4.982383000	-0.520553000
H	-1.017424000	5.874999000	-1.138784000
O	1.207134000	4.580901000	-1.172441000
C	1.086480000	1.367897000	-2.401103000
H	0.748682000	2.405270000	-2.397485000
H	0.378974000	0.787605000	-3.041332000
H	2.071033000	1.272384000	-2.864945000
Mg	2.832929000	3.628408000	-1.021370000
Br	5.011508000	3.727767000	-1.954931000

93

D′, *E*(SCF Done) = -4981.556935 a.u.

C	7.770771000	1.544518000	0.837309000
C	6.492799000	1.147502000	0.513076000
C	5.707301000	1.909138000	-0.395311000
C	6.267665000	3.094040000	-0.973754000
C	7.589288000	3.475525000	-0.617570000
C	8.323797000	2.719208000	0.268105000
H	3.968274000	0.630021000	-0.301180000
H	8.363426000	0.955592000	1.532015000
H	6.065631000	0.245767000	0.944892000
C	4.386561000	1.528186000	-0.751428000
C	5.478498000	3.837270000	-1.890995000
H	8.012357000	4.375209000	-1.058393000
H	9.333867000	3.019601000	0.533280000
C	4.203863000	3.434993000	-2.220487000
C	3.632923000	2.266624000	-1.643385000
H	5.901649000	4.732025000	-2.341768000
H	3.636931000	4.015447000	-2.943130000
C	2.219865000	1.806590000	-1.980755000

H	2.143380000	0.743864000	-1.736128000
C	1.818631000	1.991728000	-3.449939000
H	0.833111000	1.547332000	-3.621967000
H	1.765568000	3.047241000	-3.736394000
H	2.545734000	1.498343000	-4.102842000
C	0.312267000	1.711266000	-0.359092000
C	-0.037109000	3.947279000	-0.096966000
C	1.010731000	3.797056000	-0.947521000
H	-0.523917000	4.836005000	0.273068000
H	1.626078000	4.534206000	-1.438164000
N	1.213118000	2.433322000	-1.093241000
N	-0.451141000	2.670633000	0.249184000
C	-6.493762000	6.227850000	1.327516000
C	-5.335120000	5.644994000	1.792365000
C	-4.668539000	4.646498000	1.033227000
C	-5.220905000	4.252267000	-0.227097000
C	-6.416554000	4.870731000	-0.678570000
C	-7.039701000	5.837179000	0.080460000
H	-3.072499000	4.336973000	2.449312000
H	-6.994526000	6.990220000	1.917952000
H	-4.915311000	5.942740000	2.750512000
C	-3.469589000	4.027761000	1.486576000
C	-4.547475000	3.248930000	-0.977976000
H	-6.833921000	4.567637000	-1.635909000
H	-7.954466000	6.303325000	-0.275146000
C	-3.392325000	2.671749000	-0.510548000
C	-2.831158000	3.059390000	0.741044000
H	-4.966205000	2.939799000	-1.932868000
H	-2.890024000	1.904301000	-1.094436000
C	-1.549309000	2.368604000	1.192184000
H	-1.688375000	1.286194000	1.086099000
C	-1.108020000	2.655399000	2.630388000
H	-0.209991000	2.073547000	2.858430000
H	-0.874754000	3.714425000	2.785474000
H	-1.893848000	2.367148000	3.335535000
Ni	0.171755000	-0.200731000	-0.165693000
C	-2.437854000	-2.206423000	3.079538000
C	-1.271314000	-2.237880000	2.338522000
C	-1.289915000	-2.064133000	0.927877000
C	-2.568167000	-1.901579000	0.290406000
C	-3.750827000	-1.894232000	1.075333000
C	-3.688658000	-2.032749000	2.447802000
H	-2.392397000	-2.329193000	4.158247000

H	-0.315201000	-2.395907000	2.826572000
C	-0.051588000	-2.132497000	0.138895000
C	-2.608293000	-1.773535000	-1.146605000
H	-4.708201000	-1.780183000	0.573399000
H	-4.599975000	-2.021532000	3.038497000
C	-1.438884000	-1.694013000	-1.876297000
C	-0.133068000	-1.837922000	-1.279552000
H	-3.572047000	-1.686138000	-1.639252000
H	-1.499686000	-1.564974000	-2.955869000
C	4.529269000	-1.924232000	2.782006000
C	4.304402000	-3.247710000	2.464453000
C	3.146239000	-3.649900000	1.752666000
C	2.187055000	-2.660354000	1.343006000
C	2.453631000	-1.303315000	1.687924000
C	3.587779000	-0.943978000	2.387289000
H	3.619804000	-5.763726000	1.742456000
H	5.415970000	-1.634367000	3.339499000
H	5.017377000	-4.012684000	2.765401000
C	2.893970000	-5.013554000	1.435247000
C	1.019293000	-3.061287000	0.633147000
H	1.731838000	-0.537698000	1.392898000
H	3.755586000	0.099516000	2.643770000
C	0.789177000	-4.421525000	0.350200000
C	1.759677000	-5.388068000	0.763393000
H	1.559834000	-6.428585000	0.524405000
O	-0.289736000	-4.840363000	-0.290563000
C	1.019916000	-2.168127000	-2.211425000
H	0.981321000	-3.220211000	-2.528917000
H	1.987809000	-2.013418000	-1.730218000
H	0.973801000	-1.545262000	-3.112476000
Mg	-1.937587000	-4.164173000	-0.877354000
Br	-3.807186000	-5.319383000	-1.807834000

53

L2, $E_{(\text{SCF Done})}$ = -1152.199103 a.u.

C	6.667993000	-0.460437000	0.032098000
C	5.335642000	-0.775312000	-0.125548000
C	4.334951000	0.236392000	-0.202457000
C	4.765658000	1.605961000	-0.121529000
C	6.147130000	1.894913000	0.045485000
C	7.082363000	0.888775000	0.121809000
H	7.406900000	-1.255583000	0.086073000
H	5.055689000	-1.820685000	-0.202433000
C	2.934697000	-0.039594000	-0.360968000

C	3.806300000	2.648702000	-0.214248000
H	6.452937000	2.936834000	0.108156000
H	8.135366000	1.125539000	0.247471000
C	2.474677000	2.355715000	-0.385613000
C	2.043515000	1.010986000	-0.458213000
H	4.146425000	3.679810000	-0.152490000
H	1.741627000	3.154030000	-0.464437000
C	2.433962000	-1.482762000	-0.475003000
H	3.093643000	-2.127792000	0.113368000
C	2.446685000	-1.975071000	-1.929952000
H	2.161852000	-3.031345000	-1.979930000
H	1.735797000	-1.400842000	-2.526905000
H	3.449895000	-1.856160000	-2.351628000
C	-0.075139000	-1.475295000	-0.553644000
C	-0.431351000	-2.004319000	1.661311000
C	0.910613000	-1.974403000	1.465296000
H	-1.001084000	-2.232922000	2.548657000
H	1.726644000	-2.155138000	2.149757000
N	1.101584000	-1.660627000	0.122580000
N	-1.006605000	-1.696699000	0.428061000
C	-6.045640000	-0.055362000	-1.779481000
C	-4.894850000	-0.518986000	-1.179367000
C	-4.226634000	0.232722000	-0.170339000
C	-4.788986000	1.503538000	0.195295000
C	-5.978877000	1.950006000	-0.440473000
C	-6.599893000	1.190398000	-1.405008000
H	-6.528897000	-0.650597000	-2.549595000
H	-4.492177000	-1.474762000	-1.496484000
C	-3.024123000	-0.210368000	0.481205000
C	-4.144459000	2.298189000	1.180359000
H	-6.390303000	2.913608000	-0.148698000
H	-7.508588000	1.545503000	-1.883317000
C	-2.986325000	1.861199000	1.774763000
C	-2.430601000	0.608707000	1.421072000
H	-4.580739000	3.257622000	1.447898000
H	-2.486076000	2.472890000	2.520751000
C	-2.442648000	-1.586048000	0.144243000
H	-2.491681000	-1.724930000	-0.938315000
C	-3.227812000	-2.718736000	0.830714000
H	-2.808146000	-3.694396000	0.563563000
H	-3.209096000	-2.613813000	1.920692000
H	-4.276367000	-2.691872000	0.522371000
H	0.988918000	0.793342000	-0.599330000

H	-1.508489000	0.295886000	1.899495000

87

(L2)$_2$Ni, $E_{(SCF\ Done)}$ = -2475.449565 a.u.

C	0.239625000	4.659405000	0.291304000
C	-0.865783000	3.825572000	0.196859000
C	-0.922559000	2.766189000	-0.735553000
C	0.257838000	2.500353000	-1.513677000
C	1.351451000	3.391765000	-1.426682000
C	1.347189000	4.463389000	-0.548950000
H	0.243526000	5.464017000	1.021998000
H	-1.688448000	3.968986000	0.884525000
C	-2.057577000	1.875219000	-0.907351000
C	0.365509000	1.310682000	-2.340732000
H	2.221545000	3.202798000	-2.052215000
H	2.202927000	5.131321000	-0.496380000
C	-0.698386000	0.372731000	-2.399438000
C	-1.860577000	0.710163000	-1.648354000
H	1.157561000	1.287570000	-3.087007000
H	-0.773079000	-0.382451000	-3.178092000
C	-6.394517000	4.210742000	1.504710000
C	-6.637127000	2.884672000	1.221474000
C	-5.679946000	2.082467000	0.545908000
C	-4.401394000	2.654371000	0.203309000
C	-4.214721000	4.038205000	0.456979000
C	-5.178941000	4.796026000	1.091419000
H	-6.985083000	0.355433000	0.445744000
H	-7.143512000	4.809911000	2.015381000
H	-7.587658000	2.431001000	1.493735000
C	-6.010095000	0.746522000	0.167289000
C	-3.442671000	1.800101000	-0.449793000
H	-3.316159000	4.523474000	0.101121000
H	-5.003392000	5.855399000	1.259378000
C	-3.895836000	0.545659000	-0.876170000
C	-5.143148000	-0.018316000	-0.576024000
H	-5.383513000	-1.021931000	-0.911207000
O	-2.954943000	-0.104268000	-1.626983000
Ni	0.649502000	-0.193847000	-1.051168000
C	1.624600000	-1.151375000	0.281033000
N	1.500043000	-2.470917000	0.632583000
N	2.613579000	-0.688371000	1.105515000
C	0.517077000	-3.360735000	-0.004540000
C	2.388782000	-2.810042000	1.647349000
C	3.088988000	-1.685795000	1.943861000

C	3.040496000	0.729254000	1.145973000
H	0.346272000	-2.887729000	-0.976896000
C	1.114489000	-4.760536000	-0.229764000
C	-0.814636000	-3.420490000	0.746236000
H	2.464913000	-3.805635000	2.053794000
H	3.891415000	-1.519604000	2.645182000
H	2.662565000	1.148599000	0.208965000
C	2.310976000	1.453792000	2.283928000
C	4.563120000	0.836120000	1.162326000
H	2.057058000	-4.695106000	-0.783708000
H	1.295470000	-5.277423000	0.718655000
H	0.413887000	-5.374438000	-0.801805000
C	-2.011256000	-3.789394000	0.040707000
C	-0.886688000	-3.154226000	2.099111000
H	1.233390000	1.384990000	2.110962000
H	2.528930000	1.013781000	3.263078000
H	2.587640000	2.512234000	2.302967000
C	5.317539000	0.527993000	-0.023350000
C	5.236656000	1.229641000	2.302797000
C	-2.048006000	-4.057171000	-1.357964000
C	-3.245012000	-3.885454000	0.770514000
C	-2.104951000	-3.248911000	2.812355000
H	0.006585000	-2.852749000	2.636409000
C	4.712634000	0.125790000	-1.248284000
C	6.749082000	0.635640000	0.016932000
C	6.648477000	1.331581000	2.338907000
H	4.679869000	1.479094000	3.200005000
C	-3.220709000	-4.402436000	-1.994044000
H	-1.140683000	-3.982495000	-1.947560000
C	-4.436028000	-4.246875000	0.084482000
C	-3.259618000	-3.611508000	2.163820000
H	-2.117138000	-3.028292000	3.876156000
C	5.477170000	-0.149953000	-2.362177000
H	3.633967000	0.024904000	-1.314942000
C	7.504955000	0.341942000	-1.149683000
C	7.388651000	1.039301000	1.220128000
H	7.137451000	1.645363000	3.257075000
C	-4.429884000	-4.502927000	-1.267751000
H	-3.216901000	-4.595028000	-3.063404000
H	-5.359404000	-4.317676000	0.654796000
H	-4.200800000	-3.686283000	2.702983000
C	6.886485000	-0.043148000	-2.317044000
H	4.989596000	-0.454179000	-3.284665000

H	8.587899000	0.430152000	-1.101623000
H	8.473268000	1.116453000	1.235997000
H	-5.347960000	-4.779424000	-1.778963000
H	7.475791000	-0.264125000	-3.202870000

87

INT^{L2}, $E_{(SCF\ Done)}$ = -2167.398013 a.u.

C	2.190494000	1.562869000	-1.325020000
H	2.081223000	0.504402000	-1.067803000
C	2.229095000	1.704791000	-2.858451000
H	1.320599000	1.283809000	-3.301916000
H	2.314099000	2.754255000	-3.159993000
H	3.095646000	1.178978000	-3.266494000
C	0.128075000	1.513140000	0.080079000
C	-0.669704000	3.597474000	-0.390741000
C	0.476035000	3.434314000	-1.100199000
H	-1.363026000	4.422627000	-0.350819000
H	0.965881000	4.098319000	-1.794273000
N	0.959230000	2.167114000	-0.794466000
N	-0.868804000	2.423149000	0.316696000
C	-1.963551000	2.191127000	1.287099000
H	-2.004236000	1.101147000	1.381358000
C	-1.557077000	2.746971000	2.657945000
H	-0.644172000	2.240797000	2.984897000
H	-1.355485000	3.823027000	2.624750000
H	-2.342884000	2.560616000	3.397026000
Ni	0.242021000	-0.265645000	0.738505000
C	-2.590851000	-3.665438000	-2.584568000
C	-1.725344000	-3.198795000	-1.616017000
C	-2.131804000	-3.060696000	-0.261010000
C	-3.513091000	-3.325960000	0.054449000
C	-4.367861000	-3.829592000	-0.962413000
C	-3.920558000	-4.012705000	-2.252138000
H	-2.249856000	-3.754391000	-3.612793000
H	-0.727301000	-2.887270000	-1.898991000
C	-1.284166000	-2.589909000	0.794393000
C	-4.025989000	-3.036675000	1.351651000
H	-5.401265000	-4.049788000	-0.704024000
H	-4.591169000	-4.394198000	-3.017284000
C	-3.235457000	-2.461387000	2.320974000
C	-1.885118000	-2.242724000	2.009487000
H	-5.072802000	-3.245996000	1.554814000
H	-3.620168000	-2.194110000	3.299912000
C	3.872144000	-3.932756000	-0.439196000

C	3.830364000	-3.086129000	0.654545000
C	2.610216000	-2.562193000	1.138190000
C	1.388008000	-2.868121000	0.441669000
C	1.460877000	-3.780147000	-0.635797000
C	2.671636000	-4.297338000	-1.071416000
H	3.546981000	-1.586928000	2.839439000
H	4.820262000	-4.334344000	-0.786921000
H	4.746977000	-2.824575000	1.177928000
C	2.596721000	-1.792091000	2.353765000
C	0.152631000	-2.281457000	0.955558000
H	0.549910000	-4.111872000	-1.114659000
H	2.684972000	-4.997828000	-1.902594000
C	0.214112000	-1.608411000	2.220839000
C	1.429050000	-1.360776000	2.921140000
H	1.404861000	-0.849122000	3.878406000
O	-1.012274000	-1.661840000	2.889149000
C	-3.291731000	2.701630000	0.736753000
C	-3.950511000	1.987252000	-0.323920000
C	-3.876072000	3.848772000	1.239454000
C	-3.434902000	0.787932000	-0.891702000
C	-5.194209000	2.494241000	-0.833040000
C	-5.101607000	4.345378000	0.733427000
H	-3.397062000	4.390144000	2.048701000
C	-4.108964000	0.129033000	-1.898093000
H	-2.498599000	0.373990000	-0.531956000
C	-5.858750000	1.793788000	-1.875121000
C	-5.744815000	3.683243000	-0.283092000
H	-5.526512000	5.250995000	1.157667000
C	-5.330627000	0.635904000	-2.398670000
H	-3.698996000	-0.791669000	-2.302736000
H	-6.799707000	2.191158000	-2.249007000
H	-6.687118000	4.055173000	-0.678712000
H	-5.850817000	0.106570000	-3.192537000
C	3.466340000	2.095440000	-0.666148000
C	4.682357000	1.333053000	-0.771464000
C	3.492750000	3.305572000	-0.001175000
C	4.751182000	0.045212000	-1.375604000
C	5.895448000	1.878885000	-0.227601000
C	4.687571000	3.830432000	0.547194000
H	2.579389000	3.878216000	0.115033000
C	5.940683000	-0.645976000	-1.462370000
H	3.852670000	-0.423273000	-1.760746000
C	7.105770000	1.143393000	-0.341020000

C 5.867331000 3.139727000 0.425720000
H 4.660093000 4.787883000 1.060346000
C 7.135138000 -0.090126000 -0.950116000
H 5.954044000 -1.631535000 -1.918756000
H 8.014217000 1.576751000 0.071080000
H 6.792358000 3.539760000 0.833957000
H 8.068017000 -0.641791000 -1.028593000
140
TS1^{L2}, $E_{(SCF\ Done)}$ = -3319.584087 a.u., IF = -346.03 cm^{-1}
Ni 0.040032000 -0.029321000 -0.955703000
O 0.780837000 -0.338955000 -2.822450000
C -0.289661000 -1.486728000 -2.174842000
C 1.870576000 0.638232000 1.629174000
H 1.895734000 0.728016000 0.541016000
C 1.589688000 2.052758000 2.162756000
H 0.880587000 2.539247000 1.486815000
H 1.141526000 2.052476000 3.159935000
H 2.505919000 2.648875000 2.186990000
C -0.114139000 -0.718934000 0.872770000
C -0.658589000 -1.613945000 2.907336000
C 0.392829000 -0.786557000 3.105258000
H -1.220579000 -2.206896000 3.608843000
H 0.943395000 -0.552394000 4.002260000
N 0.715432000 -0.252967000 1.866855000
N -0.957631000 -1.571719000 1.549309000
C -3.525423000 -4.642059000 4.588064000
C -3.022658000 -4.012291000 3.469944000
C -3.797968000 -3.088240000 2.708460000
C -5.147265000 -2.851234000 3.158898000
C -5.633606000 -3.515842000 4.317768000
C -4.845699000 -4.392758000 5.024743000
H -2.898634000 -5.341285000 5.135013000
H -2.012435000 -4.242671000 3.164924000
C -3.330472000 -2.393524000 1.534912000
C -5.991609000 -1.960001000 2.446752000
H -6.654262000 -3.314048000 4.634515000
H -5.232310000 -4.893882000 5.907762000
C -5.535219000 -1.324488000 1.319403000
C -4.214164000 -1.554398000 0.877501000
H -7.004606000 -1.799128000 2.807793000
H -6.175683000 -0.648385000 0.760108000
C -1.930130000 -2.485926000 0.903315000
H -2.035741000 -2.084065000 -0.103773000

C	-1.310242000	-3.886109000	0.741424000
H	-0.522165000	-3.829172000	-0.012134000
H	-0.858550000	-4.262905000	1.661871000
H	-2.062336000	-4.602755000	0.397953000
C	3.229928000	7.118163000	0.708401000
C	4.134534000	6.106043000	0.929159000
C	3.990112000	4.836847000	0.306082000
C	2.875628000	4.596336000	-0.573904000
C	1.962262000	5.670694000	-0.768268000
C	2.131482000	6.891348000	-0.150703000
H	5.775611000	4.013260000	1.212203000
H	3.354692000	8.083798000	1.190615000
H	4.984728000	6.260977000	1.589405000
C	4.942171000	3.809828000	0.544137000
C	2.754451000	3.304438000	-1.199167000
H	1.098872000	5.529734000	-1.400811000
H	1.409994000	7.685298000	-0.325097000
C	3.715928000	2.346008000	-0.927402000
C	4.807602000	2.585813000	-0.061256000
H	3.640885000	1.371117000	-1.400145000
C	1.708515000	2.910683000	-2.254252000
H	1.835265000	1.842344000	-2.423059000
C	1.956973000	3.616874000	-3.597505000
H	1.236729000	3.281838000	-4.351362000
H	1.910903000	4.707383000	-3.518050000
H	2.962608000	3.362424000	-3.945091000
C	-0.355562000	2.058325000	-1.102325000
C	-1.775256000	3.767680000	-1.652344000
C	-0.572733000	4.047296000	-2.208465000
H	-2.708819000	4.300408000	-1.711107000
H	-0.287848000	4.861055000	-2.854329000
N	0.288172000	3.020644000	-1.842449000
N	-1.625039000	2.570681000	-0.968901000
C	-5.128952000	5.565369000	0.655717000
C	-4.355078000	4.465019000	0.356366000
C	-4.863555000	3.374822000	-0.409607000
C	-6.216651000	3.488019000	-0.889906000
C	-6.990386000	4.632251000	-0.554170000
C	-6.466491000	5.650492000	0.207023000
H	-4.703117000	6.376817000	1.239624000
H	-3.327778000	4.448501000	0.694674000
C	-4.105792000	2.197127000	-0.749019000
C	-6.767685000	2.466222000	-1.707301000

H	-8.011674000	4.689415000	-0.923215000
H	-7.069025000	6.520176000	0.453541000
C	-6.014652000	1.372425000	-2.055420000
C	-4.693511000	1.248087000	-1.569019000
H	-7.790988000	2.572952000	-2.058868000
H	-6.426194000	0.595451000	-2.693298000
C	-2.702586000	1.863306000	-0.228854000
H	-2.530589000	0.813440000	-0.474148000
C	-2.509890000	1.966516000	1.293988000
H	-1.565347000	1.487868000	1.559984000
H	-2.487676000	2.992499000	1.664148000
H	-3.321206000	1.433817000	1.800415000
C	-0.433783000	-6.263202000	-3.360994000
C	0.316194000	-5.159465000	-3.004306000
C	-0.286325000	-3.898013000	-2.728192000
C	-1.709734000	-3.804121000	-2.950416000
C	-2.449741000	-4.958322000	-3.299113000
C	-1.836658000	-6.181159000	-3.485832000
H	0.070649000	-7.207096000	-3.554807000
H	1.392080000	-5.258121000	-2.949359000
C	0.424490000	-2.718504000	-2.320434000
C	-2.340638000	-2.520390000	-2.934269000
H	-3.524234000	-4.856400000	-3.441944000
H	-2.418783000	-7.058926000	-3.752562000
C	-1.642118000	-1.379672000	-2.643303000
H	-3.391944000	-2.462461000	-3.212124000
H	-2.103874000	-0.399452000	-2.728240000
C	5.504581000	-4.514515000	-1.819308000
C	5.497221000	-3.296665000	-2.465103000
C	4.293185000	-2.564245000	-2.648988000
C	3.049909000	-3.137616000	-2.202074000
C	3.105478000	-4.369865000	-1.495376000
C	4.296090000	-5.042235000	-1.308637000
H	5.243872000	-0.864338000	-3.601188000
H	6.436007000	-5.057901000	-1.683449000
H	6.425565000	-2.865231000	-2.834219000
C	4.303839000	-1.265858000	-3.230941000
C	1.838324000	-2.394725000	-2.403521000
H	2.191710000	-4.766964000	-1.066977000
H	4.306248000	-5.977148000	-0.754427000
C	1.946170000	-1.047375000	-2.804359000
C	3.154014000	-0.506513000	-3.290936000
H	3.162142000	0.488667000	-3.725253000

H	-3.879003000	-1.053720000	-0.024375000
H	-4.121709000	0.366631000	-1.845322000
H	5.524966000	1.791834000	0.122546000
C	3.181672000	-0.070518000	2.010891000
C	3.846512000	-0.015484000	3.285772000
C	3.730737000	-0.855504000	1.009123000
C	3.379602000	0.720096000	4.415291000
C	5.068750000	-0.761378000	3.456652000
C	4.922103000	-1.593172000	1.183894000
H	3.220866000	-0.918426000	0.051519000
C	4.060558000	0.728579000	5.613515000
H	2.468188000	1.295277000	4.340876000
C	5.749137000	-0.724927000	4.704523000
C	5.583344000	-1.538241000	2.386483000
H	5.300995000	-2.195839000	0.363749000
C	5.262793000	0.001519000	5.765594000
H	3.667679000	1.302608000	6.448675000
H	6.669673000	-1.295697000	4.803692000
H	6.505113000	-2.094198000	2.541535000
H	5.793198000	0.016486000	6.713758000

140

A^{L2}, *E*$_{\text{(SCF Done)}}$ = -3319.626629 a.u.

Ni	0.011725000	-0.227105000	-1.042727000
O	1.179350000	-0.206879000	-2.588477000
C	-0.428611000	-1.989612000	-1.642654000
C	1.686962000	0.818981000	1.531598000
H	1.775434000	0.767413000	0.446376000
C	1.485794000	2.301497000	1.884394000
H	0.851335000	2.758740000	1.121216000
H	0.986086000	2.442734000	2.846290000
H	2.440849000	2.831676000	1.900628000
C	-0.398111000	-0.511508000	0.907235000
C	-1.015636000	-1.029775000	3.048088000
C	0.085269000	-0.252782000	3.128825000
H	-1.621278000	-1.468371000	3.822495000
H	0.647498000	0.085723000	3.983711000
N	0.452028000	0.052185000	1.828480000
N	-1.301190000	-1.189874000	1.698317000
C	-3.873371000	-3.758321000	5.218456000
C	-3.389719000	-3.287454000	4.016788000
C	-4.154694000	-2.419508000	3.182179000
C	-5.470140000	-2.062154000	3.652153000
C	-5.937480000	-2.565024000	4.897073000

C	-5.161531000	-3.394882000	5.670938000
H	-3.256363000	-4.419844000	5.820506000
H	-2.403496000	-3.599305000	3.705033000
C	-3.705784000	-1.889384000	1.919206000
C	-6.299193000	-1.213005000	2.874028000
H	-6.932970000	-2.276376000	5.226132000
H	-5.532885000	-3.771849000	6.619685000
C	-5.860843000	-0.732650000	1.665461000
C	-4.573380000	-1.081051000	1.201807000
H	-7.287298000	-0.959532000	3.250340000
H	-6.492996000	-0.091850000	1.057461000
C	-2.346275000	-2.143967000	1.244909000
H	-2.499299000	-1.897363000	0.201606000
C	-1.787764000	-3.578583000	1.252828000
H	-1.069334000	-3.674128000	0.434890000
H	-1.265683000	-3.828258000	2.179274000
H	-2.592569000	-4.303200000	1.095272000
C	3.701530000	7.068474000	0.377303000
C	4.513409000	6.009365000	0.709491000
C	4.284309000	4.708251000	0.185306000
C	3.177909000	4.481035000	-0.709363000
C	2.366519000	5.607264000	-1.023085000
C	2.615832000	6.858581000	-0.501579000
H	5.975528000	3.830484000	1.213282000
H	3.890917000	8.057911000	0.784326000
H	5.355098000	6.150544000	1.383624000
C	5.146881000	3.635759000	0.536661000
C	2.965238000	3.152414000	-1.227898000
H	1.521374000	5.486537000	-1.681597000
H	1.969665000	7.690283000	-0.769980000
C	3.847830000	2.153556000	-0.851480000
C	4.934386000	2.381209000	0.024243000
H	3.713040000	1.152107000	-1.246965000
C	1.907457000	2.733048000	-2.262813000
H	1.999312000	1.654882000	-2.392352000
C	2.145463000	3.366375000	-3.643437000
H	1.415244000	2.988061000	-4.366315000
H	2.108850000	4.460119000	-3.636798000
H	3.144643000	3.081250000	-3.986697000
C	-0.233090000	1.900185000	-1.192144000
C	-1.548133000	3.714170000	-1.659654000
C	-0.317657000	3.964716000	-2.161339000
H	-2.458125000	4.290450000	-1.702933000

H	0.021790000	4.795772000	-2.754728000
N	0.483105000	2.874338000	-1.848604000
N	-1.478987000	2.463206000	-1.069421000
C	-5.916380000	4.973486000	-0.149124000
C	-4.900932000	4.044602000	-0.222159000
C	-4.928858000	2.968252000	-1.158141000
C	-6.062644000	2.911745000	-2.048538000
C	-7.097994000	3.879607000	-1.936869000
C	-7.035700000	4.890571000	-1.007557000
H	-5.852276000	5.779258000	0.577213000
H	-4.061259000	4.151718000	0.447997000
C	-3.903749000	1.965316000	-1.288820000
C	-6.136009000	1.905975000	-3.046622000
H	-7.943624000	3.807282000	-2.616722000
H	-7.832641000	5.625485000	-0.937252000
C	-5.124615000	0.987766000	-3.183373000
C	-4.023037000	1.026395000	-2.301961000
H	-7.000464000	1.886052000	-3.705743000
H	-5.164923000	0.225544000	-3.956065000
C	-2.640990000	1.816746000	-0.419080000
H	-2.389136000	0.755995000	-0.447610000
C	-2.704340000	2.201408000	1.069422000
H	-1.900828000	1.683369000	1.598554000
H	-2.557434000	3.271639000	1.233279000
H	-3.657281000	1.900194000	1.513793000
C	0.581419000	-6.115065000	-4.105538000
C	1.021810000	-4.984096000	-3.451754000
C	0.118032000	-4.083401000	-2.815062000
C	-1.283626000	-4.360605000	-2.970314000
C	-1.707253000	-5.546569000	-3.629824000
C	-0.797475000	-6.419398000	-4.178861000
H	1.305023000	-6.775465000	-4.577058000
H	2.082270000	-4.774092000	-3.430754000
C	0.532044000	-2.891212000	-2.098350000
C	-2.225803000	-3.400123000	-2.528768000
H	-2.775569000	-5.737746000	-3.710666000
H	-1.132484000	-7.320349000	-4.686454000
C	-1.797887000	-2.239975000	-1.924806000
H	-3.285178000	-3.578337000	-2.706403000
H	-2.546516000	-1.494870000	-1.667480000
C	5.379054000	-4.789288000	-1.077122000
C	5.528040000	-3.598955000	-1.756469000
C	4.405293000	-2.800717000	-2.099112000

C	3.081198000	-3.250954000	-1.764044000
C	2.969458000	-4.481685000	-1.049936000
C	4.080861000	-5.223726000	-0.713483000
H	5.534805000	-1.236362000	-3.087736000
H	6.247638000	-5.388200000	-0.816240000
H	6.518699000	-3.243934000	-2.034946000
C	4.545194000	-1.553241000	-2.764072000
C	1.945468000	-2.446164000	-2.096483000
H	1.981632000	-4.825993000	-0.760743000
H	3.959748000	-6.151222000	-0.159031000
C	2.143574000	-1.114037000	-2.533611000
C	3.458643000	-0.739493000	-2.968144000
H	3.573496000	0.221568000	-3.462142000
H	-4.260375000	-0.697520000	0.234963000
H	-3.230282000	0.296368000	-2.435636000
H	5.585113000	1.553236000	0.288922000
C	2.914095000	0.069405000	2.083982000
C	3.531926000	0.260176000	3.370940000
C	3.428252000	-0.899576000	1.237006000
C	3.100011000	1.196723000	4.357134000
C	4.670493000	-0.557195000	3.711793000
C	4.538157000	-1.702984000	1.578949000
H	2.958646000	-1.061108000	0.270582000
C	3.734235000	1.323039000	5.574485000
H	2.255791000	1.839344000	4.155890000
C	5.304511000	-0.396298000	4.974101000
C	5.151470000	-1.527206000	2.794709000
H	4.894023000	-2.447609000	0.873739000
C	4.852834000	0.521065000	5.892931000
H	3.369177000	2.050895000	6.294454000
H	6.161601000	-1.026397000	5.200552000
H	6.010456000	-2.131308000	3.077460000
H	5.347047000	0.629763000	6.854395000

146

B^{L2}, $E_{(\text{SCF Done})}$ = -6133.746115 a.u.

Ni	-0.009053000	-0.263797000	-0.669208000
O	1.290502000	-0.133469000	-2.204017000
C	-0.335712000	-1.986401000	-1.447737000
C	1.326469000	0.575615000	2.220363000
H	1.584174000	0.634684000	1.163196000
C	0.964196000	2.000354000	2.668199000
H	0.431362000	2.497999000	1.854737000
H	0.304732000	2.010496000	3.539828000

H	1.861801000	2.578630000	2.896159000
C	-0.589791000	-0.763923000	1.179138000
C	-1.452231000	-1.524347000	3.153489000
C	-0.399329000	-0.736912000	3.456685000
H	-2.133658000	-2.059517000	3.792289000
H	0.037954000	-0.479148000	4.407221000
N	0.117302000	-0.284581000	2.254648000
N	-1.560229000	-1.544980000	1.770367000
C	-4.502799000	-4.493221000	4.674595000
C	-3.882022000	-3.895798000	3.598714000
C	-4.550884000	-2.944310000	2.772522000
C	-5.918882000	-2.640287000	3.112558000
C	-6.528318000	-3.274626000	4.229358000
C	-5.841761000	-4.182123000	5.000466000
H	-3.954822000	-5.214628000	5.274623000
H	-2.860730000	-4.172526000	3.381016000
C	-3.959971000	-2.280178000	1.637701000
C	-6.658791000	-1.712291000	2.334531000
H	-7.560545000	-3.023721000	4.461735000
H	-6.321379000	-4.659179000	5.850384000
C	-6.083283000	-1.102076000	1.248369000
C	-4.744546000	-1.398159000	0.912012000
H	-7.688896000	-1.502354000	2.611789000
H	-6.644451000	-0.399438000	0.639243000
C	-2.526848000	-2.459850000	1.106269000
H	-2.562612000	-2.115556000	0.080737000
C	-1.944980000	-3.883722000	1.050595000
H	-1.134769000	-3.899944000	0.317692000
H	-1.528327000	-4.210899000	2.005811000
H	-2.712081000	-4.598484000	0.737649000
C	2.836764000	6.850138000	2.238471000
C	3.720112000	5.842656000	2.547981000
C	3.733746000	4.621774000	1.820342000
C	2.806708000	4.425100000	0.735272000
C	1.908087000	5.492927000	0.455749000
C	1.920553000	6.666299000	1.179157000
H	5.350248000	3.769971000	2.981882000
H	2.840052000	7.779007000	2.802049000
H	4.431651000	5.963965000	3.361424000
C	4.660009000	3.599413000	2.159299000
C	2.843437000	3.179427000	0.010289000
H	1.180581000	5.385729000	-0.333833000
H	1.216864000	7.456462000	0.930868000

C	3.773187000	2.225521000	0.388379000
C	4.678977000	2.421780000	1.456088000
H	3.824341000	1.289608000	-0.159734000
C	2.048236000	2.848209000	-1.264130000
H	2.253412000	1.805171000	-1.495659000
C	2.542895000	3.691350000	-2.452157000
H	2.054004000	3.399869000	-3.386147000
H	2.419089000	4.765996000	-2.286987000
H	3.612888000	3.505603000	-2.578121000
C	-0.229995000	1.868890000	-0.656144000
C	-1.500914000	3.658400000	-1.293531000
C	-0.212203000	3.955971000	-1.576961000
H	-2.405038000	4.211754000	-1.482538000
H	0.199690000	4.812273000	-2.082626000
N	0.559427000	2.882159000	-1.154699000
N	-1.495848000	2.398782000	-0.720264000
C	-5.516130000	5.266377000	0.142806000
C	-4.628425000	4.219040000	0.020141000
C	-4.842457000	3.153247000	-0.902912000
C	-6.018351000	3.238059000	-1.731948000
C	-6.918843000	4.325887000	-1.571319000
C	-6.683011000	5.320589000	-0.651764000
H	-5.311686000	6.060021000	0.856373000
H	-3.737379000	4.228674000	0.631753000
C	-3.958158000	2.028339000	-1.077721000
C	-6.265994000	2.248580000	-2.719577000
H	-7.801066000	4.360333000	-2.206052000
H	-7.378272000	6.148215000	-0.543703000
C	-5.384469000	1.213263000	-2.903118000
C	-4.244251000	1.111398000	-2.075743000
H	-7.157172000	2.337744000	-3.335937000
H	-5.551786000	0.468094000	-3.674853000
C	-2.719303000	1.715104000	-0.225212000
H	-2.505986000	0.658160000	-0.391329000
C	-2.864151000	1.890796000	1.297888000
H	-2.082680000	1.317212000	1.799483000
H	-2.762847000	2.928339000	1.620067000
H	-3.836271000	1.515651000	1.631460000
C	1.190047000	-5.865004000	-4.039797000
C	1.486751000	-4.781151000	-3.241631000
C	0.462904000	-3.957789000	-2.687673000
C	-0.889481000	-4.255806000	-3.071835000
C	-1.164891000	-5.392821000	-3.879210000

C	-0.150234000	-6.193686000	-4.348016000
H	1.999097000	-6.467977000	-4.443878000
H	2.523864000	-4.549048000	-3.040738000
C	0.714556000	-2.816963000	-1.829295000
C	-1.922140000	-3.358142000	-2.704675000
H	-2.200296000	-5.602691000	-4.138816000
H	-0.370956000	-7.056143000	-4.971205000
C	-1.638284000	-2.242134000	-1.951472000
H	-2.932795000	-3.542721000	-3.063876000
H	-2.439007000	-1.530846000	-1.764971000
C	5.407649000	-4.618515000	-0.166691000
C	5.618488000	-3.378497000	-0.728378000
C	4.532302000	-2.588884000	-1.194479000
C	3.195631000	-3.107748000	-1.112296000
C	3.015471000	-4.385946000	-0.507990000
C	4.087435000	-5.117179000	-0.045034000
H	5.743107000	-0.922069000	-1.867719000
H	6.246723000	-5.211289000	0.187627000
H	6.625910000	-2.977770000	-0.819407000
C	4.729100000	-1.294492000	-1.742256000
C	2.097262000	-2.323189000	-1.594676000
H	2.008666000	-4.779359000	-0.412557000
H	3.918902000	-6.086045000	0.417876000
C	2.326144000	-0.987729000	-1.957416000
C	3.658544000	-0.508371000	-2.097863000
H	3.807640000	0.470407000	-2.540566000
H	-4.321247000	-0.910259000	0.039167000
H	-3.557580000	0.288927000	-2.248819000
H	5.378502000	1.631735000	1.712025000
C	2.502194000	-0.151564000	2.899086000
C	2.900803000	-0.043960000	4.279334000
C	3.205398000	-1.009725000	2.069386000
C	2.262030000	0.775911000	5.257296000
C	4.025306000	-0.827122000	4.729678000
C	4.299664000	-1.780870000	2.517774000
H	2.903698000	-1.102947000	1.029936000
C	2.692668000	0.824864000	6.565853000
H	1.418312000	1.389314000	4.978707000
C	4.446494000	-0.747913000	6.085244000
C	4.704676000	-1.684068000	3.825937000
H	4.808404000	-2.439632000	1.821254000
C	3.799617000	0.057619000	6.991945000
H	2.174384000	1.464861000	7.275073000

H	5.298551000	-1.349727000	6.392734000
H	5.547620000	-2.265324000	4.192254000
H	4.132157000	0.105171000	8.025105000
C	-1.424750000	0.602570000	-4.475037000
H	-1.912268000	1.414388000	-3.916407000
H	-1.989704000	-0.317245000	-4.268050000
H	-1.564029000	0.830881000	-5.541237000
Mg	0.624636000	0.401587000	-4.030141000
Br	2.562962000	1.089390000	-5.361250000

146

TS2^{L2}, $E_{(SCF\ Done)}$ = -6133.737223 a.u., IF = -100.95 cm^{-1}

C	0.897749000	1.092564000	2.192445000
H	1.394092000	1.032016000	1.224652000
C	0.223154000	2.474964000	2.280431000
H	-0.131153000	2.780621000	1.298047000
H	-0.640354000	2.474692000	2.949206000
H	0.938217000	3.223989000	2.630454000
C	-0.562340000	-0.690985000	1.039214000
C	-1.537366000	-1.461991000	2.956834000
C	-0.701075000	-0.464283000	3.309864000
H	-2.190980000	-2.071269000	3.556916000
H	-0.459354000	-0.062041000	4.279149000
N	-0.111504000	-0.001249000	2.144226000
N	-1.446618000	-1.599624000	1.580032000
C	-4.506106000	-4.562266000	4.318110000
C	-3.775508000	-4.021542000	3.281887000
C	-4.386511000	-3.258818000	2.242778000
C	-5.816910000	-3.092703000	2.317839000
C	-6.539451000	-3.661101000	3.402216000
C	-5.905199000	-4.379792000	4.387531000
H	-3.998209000	-5.140012000	5.085557000
H	-2.710659000	-4.202430000	3.260521000
C	-3.681787000	-2.660986000	1.136177000
C	-6.506119000	-2.368766000	1.310049000
H	-7.616620000	-3.515572000	3.433362000
H	-6.471372000	-4.809482000	5.208980000
C	-5.821131000	-1.831285000	0.249394000
C	-4.420322000	-1.990620000	0.174740000
H	-7.584723000	-2.258822000	1.391082000
H	-6.339134000	-1.286251000	-0.534202000
C	-2.164805000	-2.696782000	0.878341000
H	-2.049974000	-2.456796000	-0.171971000
C	-1.427821000	-4.030229000	1.094904000

H	-0.501632000	-4.016238000	0.516251000
H	-1.157667000	-4.203354000	2.139047000
H	-2.042224000	-4.866367000	0.747994000
C	2.951011000	8.063831000	-1.113456000
C	3.555572000	7.362959000	-0.097052000
C	3.413520000	5.953156000	0.018158000
C	2.607632000	5.231844000	-0.935480000
C	2.015999000	6.000566000	-1.981297000
C	2.178990000	7.366501000	-2.069015000
H	4.676627000	5.826065000	1.770004000
H	3.071812000	9.140719000	-1.189922000
H	4.164739000	7.879090000	0.641304000
C	4.074232000	5.258374000	1.064948000
C	2.468565000	3.804340000	-0.781791000
H	1.438293000	5.506944000	-2.749261000
H	1.714314000	7.908899000	-2.888001000
C	3.168383000	3.186161000	0.242152000
C	3.965552000	3.894582000	1.168332000
H	3.115980000	2.106490000	0.335762000
C	1.621662000	2.860006000	-1.652192000
H	1.965354000	1.849652000	-1.435549000
C	1.734985000	3.002619000	-3.180003000
H	1.362976000	2.088400000	-3.648824000
H	1.153087000	3.830027000	-3.592189000
H	2.782401000	3.136087000	-3.467206000
C	-0.534950000	1.729474000	-0.890207000
C	-1.864853000	3.585451000	-0.951917000
C	-0.612122000	3.975556000	-1.277505000
H	-2.779319000	4.150984000	-0.891263000
H	-0.228290000	4.952114000	-1.516723000
N	0.187856000	2.843046000	-1.241698000
N	-1.809117000	2.219395000	-0.721431000
C	-6.177549000	4.662007000	0.019264000
C	-5.196601000	3.698946000	-0.082353000
C	-5.193179000	2.727569000	-1.127340000
C	-6.269341000	2.806853000	-2.083972000
C	-7.267987000	3.809143000	-1.946543000
C	-7.231609000	4.721519000	-0.919046000
H	-6.137027000	5.382114000	0.832103000
H	-4.410880000	3.695127000	0.658838000
C	-4.198070000	1.696951000	-1.294660000
C	-6.330599000	1.891958000	-3.167067000
H	-8.066609000	3.841470000	-2.683817000

H	-8.000641000	5.483379000	-0.828965000
C	-5.364725000	0.929196000	-3.317132000
C	-4.314055000	0.840858000	-2.377777000
H	-7.151559000	1.974428000	-3.875054000
H	-5.396907000	0.232218000	-4.149430000
C	-3.007618000	1.420843000	-0.355518000
H	-2.700061000	0.395748000	-0.560963000
C	-3.293289000	1.498485000	1.156409000
H	-2.570512000	0.887534000	1.697503000
H	-3.213890000	2.514037000	1.549630000
H	-4.294800000	1.116454000	1.374059000
H	-3.904780000	-1.566895000	-0.681557000
H	-3.557082000	0.078852000	-2.531155000
H	4.482284000	3.350103000	1.953352000
C	1.977992000	0.786494000	3.248630000
C	1.963064000	1.196020000	4.633070000
C	3.053487000	0.047080000	2.787798000
C	0.908508000	1.917505000	5.269332000
C	3.087534000	0.841167000	5.463475000
C	4.143053000	-0.310615000	3.612162000
H	3.065897000	-0.276898000	1.752265000
C	0.960588000	2.272662000	6.600558000
H	0.032158000	2.201271000	4.706397000
C	3.113278000	1.230668000	6.830270000
C	4.163056000	0.089767000	4.923736000
H	4.948589000	-0.905242000	3.195744000
C	2.076676000	1.933789000	7.396390000
H	0.130998000	2.820963000	7.039076000
H	3.978629000	0.949825000	7.426006000
H	4.996143000	-0.168881000	5.573004000
H	2.109724000	2.221675000	8.443355000
Ni	-0.009583000	-0.283659000	-0.837922000
C	0.264746000	-2.150946000	-1.330243000
C	-0.791636000	0.040203000	-3.596306000
Mg	1.176053000	-0.800944000	-3.463061000
C	2.997535000	-6.028549000	-2.620690000
C	2.898155000	-4.792287000	-2.019639000
C	1.646564000	-4.123966000	-1.889663000
C	0.508226000	-4.755218000	-2.493000000
C	0.635878000	-6.040144000	-3.085337000
C	1.853592000	-6.676545000	-3.139216000
H	3.971359000	-6.502945000	-2.707842000
H	3.796733000	-4.313340000	-1.655964000

C	1.487031000	-2.830701000	-1.239287000
C	-0.716792000	-4.044770000	-2.555035000
H	-0.249150000	-6.498450000	-3.520985000
H	1.944498000	-7.654766000	-3.603663000
C	-0.810959000	-2.776467000	-2.034061000
H	-1.563674000	-4.497285000	-3.067384000
H	-1.736591000	-2.227217000	-2.186297000
C	5.453065000	-3.986541000	2.043018000
C	5.778557000	-2.808343000	1.408984000
C	4.860550000	-2.162833000	0.536592000
C	3.586929000	-2.772217000	0.268385000
C	3.279542000	-3.980480000	0.960968000
C	4.179461000	-4.564966000	1.824875000
H	6.140466000	-0.469276000	0.097909000
H	6.162464000	-4.467505000	2.711005000
H	6.749698000	-2.344170000	1.571408000
C	5.163509000	-0.916493000	-0.072755000
C	2.676832000	-2.151041000	-0.650398000
H	2.311999000	-4.443398000	0.798596000
H	3.910047000	-5.482631000	2.341637000
C	2.956945000	-0.852517000	-1.112189000
C	4.231645000	-0.268924000	-0.845950000
H	4.452415000	0.691012000	-1.303172000
O	2.068436000	-0.149565000	-1.827817000
Br	2.489874000	-1.582197000	-5.339472000
H	-1.232732000	-0.662966000	-4.322647000
H	-1.498202000	0.092362000	-2.761837000
H	-0.784651000	1.035819000	-4.060627000

146

C^{L2}, $E_{(SCF\ Done)}$ = -6133.770911 a.u.

Ni	0.334958000	0.416178000	-1.028145000
C	0.972147000	0.647794000	-2.979715000
C	-0.111120000	2.363340000	-0.964419000
C	-1.322163000	-1.850008000	1.232966000
H	-1.615653000	-1.520599000	0.236284000
C	-0.511561000	-3.151129000	1.070180000
H	0.086946000	-3.103839000	0.162255000
H	0.177518000	-3.319331000	1.900624000
H	-1.181457000	-4.010920000	0.990752000
C	0.219002000	0.195611000	1.015999000
C	0.644644000	0.406032000	3.254998000
C	-0.211624000	-0.625150000	3.106730000
H	1.091253000	0.813440000	4.144526000

H	-0.677427000	-1.257795000	3.842667000
N	-0.459631000	-0.750124000	1.748850000
N	0.905063000	0.903790000	1.986353000
C	3.091242000	2.758239000	6.112465000
C	2.673173000	2.585117000	4.810439000
C	3.553830000	2.124704000	3.786925000
C	4.920117000	1.876802000	4.175146000
C	5.316108000	2.057004000	5.528569000
C	4.425919000	2.484231000	6.484887000
H	2.385351000	3.116167000	6.856952000
H	1.648831000	2.829247000	4.568764000
C	3.180204000	1.914949000	2.408811000
C	5.875727000	1.468749000	3.207857000
H	6.351531000	1.853830000	5.791431000
H	4.744283000	2.620440000	7.514474000
C	5.512810000	1.325149000	1.892685000
C	4.172210000	1.555453000	1.512932000
H	6.899653000	1.291316000	3.527484000
H	6.239526000	1.037016000	1.138765000
C	1.777262000	2.097168000	1.798327000
H	1.931001000	2.143241000	0.723460000
C	1.038770000	3.389230000	2.193839000
H	0.362038000	3.676414000	1.389684000
H	0.440815000	3.270907000	3.100302000
H	1.752183000	4.204147000	2.348725000
C	-2.793191000	-7.032488000	-1.253011000
C	-3.820421000	-6.125355000	-1.140205000
C	-3.640310000	-4.756083000	-1.475820000
C	-2.356910000	-4.295162000	-1.942174000
C	-1.323209000	-5.269248000	-2.036002000
C	-1.530068000	-6.591703000	-1.705899000
H	-5.683000000	-4.219602000	-1.007680000
H	-2.947151000	-8.076289000	-0.993945000
H	-4.799459000	-6.442615000	-0.788985000
C	-4.723993000	-3.847145000	-1.359334000
C	-2.206360000	-2.900940000	-2.276842000
H	-0.335882000	-4.971538000	-2.350707000
H	-0.709964000	-7.299890000	-1.791888000
C	-3.300283000	-2.061814000	-2.136959000
C	-4.556050000	-2.525315000	-1.682560000
H	-3.195956000	-1.013370000	-2.405072000
C	-1.000508000	-2.237540000	-2.967231000
H	-1.191867000	-1.165688000	-2.909159000

C	-0.946443000	-2.624722000	-4.456701000
H	-0.113084000	-2.128571000	-4.965406000
H	-0.860636000	-3.706502000	-4.601938000
H	-1.878168000	-2.305934000	-4.931451000
C	0.899166000	-1.486747000	-1.458110000
C	2.405525000	-3.105914000	-2.018349000
C	1.273297000	-3.346719000	-2.715274000
H	3.356081000	-3.612485000	-2.028898000
H	1.076308000	-4.092167000	-3.466148000
N	0.344678000	-2.379520000	-2.343724000
N	2.161324000	-1.976755000	-1.249588000
C	6.528835000	-4.390086000	-0.535145000
C	5.491077000	-3.484382000	-0.485868000
C	5.581235000	-2.199666000	-1.098899000
C	6.809588000	-1.894015000	-1.788815000
C	7.862478000	-2.848752000	-1.814134000
C	7.733683000	-4.072587000	-1.201341000
H	6.416813000	-5.358480000	-0.055145000
H	4.586571000	-3.770700000	0.031227000
C	4.534497000	-1.209923000	-1.085872000
C	6.964654000	-0.647844000	-2.450126000
H	8.778352000	-2.589461000	-2.339673000
H	8.546078000	-4.792989000	-1.231536000
C	5.947289000	0.273487000	-2.444426000
C	4.746948000	-0.017450000	-1.759723000
H	7.900026000	-0.441680000	-2.964296000
H	6.055512000	1.225575000	-2.955608000
C	3.175049000	-1.337022000	-0.376908000
H	2.800992000	-0.318323000	-0.271257000
C	3.159280000	-1.948627000	1.036487000
H	2.271556000	-1.597934000	1.567186000
H	3.120038000	-3.039974000	1.026063000
H	4.042029000	-1.633059000	1.599608000
C	-2.704431000	6.558132000	-0.864826000
C	-2.657676000	5.183258000	-0.774319000
C	-1.423963000	4.474365000	-0.843812000
C	-0.238862000	5.248547000	-1.063166000
C	-0.313504000	6.664611000	-1.135751000
C	-1.521758000	7.313644000	-1.030910000
H	-3.664354000	7.065025000	-0.813776000
H	-3.579470000	4.628301000	-0.659224000
C	-1.339267000	3.019773000	-0.730319000
C	0.989644000	4.567923000	-1.259040000

H	0.605109000	7.224127000	-1.296954000
H	-1.572286000	8.397223000	-1.095710000
C	1.025816000	3.196684000	-1.259961000
H	1.888271000	5.148121000	-1.459978000
H	1.972348000	2.714817000	-1.496935000
C	-4.832875000	3.064303000	3.168982000
C	-5.428182000	2.403419000	2.117544000
C	-4.718255000	2.129476000	0.920116000
C	-3.352057000	2.553551000	0.782223000
C	-2.774753000	3.236642000	1.892126000
C	-3.486924000	3.479541000	3.047181000
H	-6.384633000	1.183709000	-0.095389000
H	-5.387785000	3.266013000	4.081168000
H	-6.464514000	2.077997000	2.188435000
C	-5.333182000	1.456426000	-0.170485000
C	-2.635107000	2.307423000	-0.438486000
H	-1.748981000	3.574694000	1.819375000
H	-3.008443000	4.001995000	3.872363000
C	-3.248045000	1.549186000	-1.458709000
C	-4.620483000	1.159567000	-1.301409000
H	-5.087007000	0.662571000	-2.147566000
H	3.915917000	1.449347000	0.464662000
H	3.958575000	0.729920000	-1.767077000
H	-5.376766000	-1.821323000	-1.588116000
C	-2.627841000	-1.974307000	2.040712000
C	-2.832250000	-2.787118000	3.217136000
C	-3.697132000	-1.253866000	1.541001000
C	-1.823245000	-3.550215000	3.877869000
C	-4.151412000	-2.834006000	3.797417000
C	-4.983739000	-1.299524000	2.120761000
H	-3.552688000	-0.633198000	0.663276000
C	-2.088612000	-4.303399000	5.001974000
H	-0.810859000	-3.544576000	3.502419000
C	-4.392993000	-3.626066000	4.952432000
C	-5.209270000	-2.083336000	3.222402000
H	-5.782044000	-0.712958000	1.679672000
C	-3.389182000	-4.351369000	5.549291000
H	-1.284475000	-4.865633000	5.469498000
H	-5.400565000	-3.640027000	5.361594000
H	-6.195502000	-2.138374000	3.677272000
H	-3.589234000	-4.949967000	6.433561000
O	-2.645780000	1.218737000	-2.586968000
H	2.001097000	0.316002000	-2.811426000

H	1.123040000	1.639007000	-3.442726000
H	0.586216000	-0.015981000	-3.767166000
Mg	-0.953442000	1.950344000	-3.105445000
Br	-0.754170000	3.191592000	-5.172798000

146

TS3^{L2}, $E_{(SCF\ Done)}$ = -6133.724972 a.u., IF = -396.24 cm^{-1}

Ni	0.334558000	0.336678000	-0.613840000
C	0.792724000	1.773377000	-2.155610000
C	1.045965000	2.106879000	-0.378268000
C	-2.305166000	-1.534237000	0.751409000
H	-2.319774000	-0.742246000	0.001899000
C	-2.146064000	-2.868423000	-0.003827000
H	-1.483620000	-2.726851000	-0.855691000
H	-1.710371000	-3.654088000	0.616747000
H	-3.114726000	-3.213620000	-0.375353000
C	-0.000029000	-0.496800000	1.187639000
C	0.241102000	-1.322884000	3.313999000
C	-0.963799000	-1.730415000	2.862036000
H	0.732457000	-1.509581000	4.253370000
H	-1.732301000	-2.306262000	3.348538000
N	-1.102226000	-1.233463000	1.574614000
N	0.819563000	-0.576901000	2.296717000
C	3.288096000	-1.390812000	6.607794000
C	2.928584000	-0.914259000	5.365481000
C	3.582188000	-1.343668000	4.172558000
C	4.656905000	-2.291947000	4.328571000
C	4.999230000	-2.766356000	5.624291000
C	4.332262000	-2.332539000	6.745290000
H	2.764656000	-1.032421000	7.490032000
H	2.135681000	-0.182413000	5.309229000
C	3.250384000	-0.889417000	2.845250000
C	5.378297000	-2.746278000	3.193710000
H	5.811162000	-3.484700000	5.709945000
H	4.606744000	-2.702906000	7.728950000
C	5.067822000	-2.278363000	1.941536000
C	4.011876000	-1.353711000	1.785803000
H	6.185696000	-3.459846000	3.338998000
H	5.624478000	-2.605893000	1.068194000
C	2.130256000	0.095882000	2.465413000
H	2.375338000	0.440846000	1.464666000
C	1.990290000	1.362101000	3.330649000
H	1.547795000	2.159338000	2.730863000
H	1.348638000	1.212532000	4.202505000

H	2.970963000	1.705038000	3.673759000
C	-5.951845000	-3.640969000	-3.227994000
C	-6.326284000	-2.441931000	-2.667872000
C	-5.407669000	-1.364423000	-2.545717000
C	-4.055880000	-1.524747000	-3.014302000
C	-3.710622000	-2.783852000	-3.581870000
C	-4.625968000	-3.809189000	-3.687126000
H	-6.845305000	-0.031336000	-1.631042000
H	-6.665574000	-4.455510000	-3.314267000
H	-7.340638000	-2.296802000	-2.303972000
C	-5.818129000	-0.131986000	-1.972589000
C	-3.144849000	-0.418207000	-2.883495000
H	-2.699725000	-2.958321000	-3.920167000
H	-4.322555000	-4.757180000	-4.123640000
C	-3.598625000	0.752372000	-2.298019000
C	-4.929605000	0.906093000	-1.847461000
H	-2.923460000	1.597411000	-2.190570000
C	-1.742751000	-0.336929000	-3.499064000
H	-1.320481000	0.596514000	-3.132102000
C	-1.814462000	-0.225228000	-5.032490000
H	-0.815209000	-0.107892000	-5.465230000
H	-2.305820000	-1.086996000	-5.494391000
H	-2.404554000	0.659424000	-5.286798000
C	0.039106000	-1.252744000	-1.957663000
C	0.664922000	-3.055025000	-3.220450000
C	-0.352937000	-2.442662000	-3.869136000
H	1.259134000	-3.910372000	-3.498501000
H	-0.794095000	-2.670886000	-4.824863000
N	-0.739923000	-1.357793000	-3.087848000
N	0.891195000	-2.321379000	-2.065184000
C	3.854926000	-6.384745000	-2.754948000
C	3.264915000	-5.244083000	-2.254641000
C	3.897297000	-3.968665000	-2.341134000
C	5.183378000	-3.922063000	-2.990895000
C	5.766606000	-5.119358000	-3.488145000
C	5.123265000	-6.329109000	-3.375518000
H	3.337711000	-7.336584000	-2.669300000
H	2.294133000	-5.331292000	-1.788228000
C	3.333795000	-2.740962000	-1.843712000
C	5.858905000	-2.683452000	-3.145354000
H	6.739047000	-5.055362000	-3.970458000
H	5.580381000	-7.235042000	-3.763243000
C	5.292390000	-1.520001000	-2.686656000

C	4.038224000	-1.563639000	-2.039629000
H	6.827301000	-2.674217000	-3.639328000
H	5.799499000	-0.567530000	-2.810307000
C	1.991985000	-2.585762000	-1.109438000
H	2.062102000	-1.644255000	-0.561627000
C	1.595121000	-3.650018000	-0.071022000
H	0.894778000	-3.203587000	0.639213000
H	1.099025000	-4.516805000	-0.514219000
H	2.473771000	-3.991905000	0.483630000
C	1.224654000	6.586538000	1.815227000
C	0.497205000	5.591784000	1.182811000
C	1.116613000	4.376558000	0.744294000
C	2.533144000	4.256894000	0.981733000
C	3.240363000	5.287428000	1.631292000
C	2.604902000	6.443742000	2.051922000
H	0.712481000	7.489062000	2.139427000
H	-0.570056000	5.717437000	1.038055000
C	0.377625000	3.321755000	0.062474000
C	3.185523000	3.077744000	0.495632000
H	4.309340000	5.159559000	1.788516000
H	3.162839000	7.232148000	2.548068000
C	2.497237000	2.102653000	-0.156568000
H	4.262563000	2.991021000	0.626424000
H	3.060977000	1.269297000	-0.572894000
C	-3.792026000	2.975705000	3.197209000
C	-4.249098000	3.524251000	2.017917000
C	-3.391928000	3.684632000	0.898366000
C	-2.015448000	3.278534000	0.991897000
C	-1.590945000	2.686129000	2.215575000
C	-2.448439000	2.543077000	3.285591000
H	-4.887276000	4.593929000	-0.382916000
H	-4.456217000	2.869564000	4.050536000
H	-5.279361000	3.866538000	1.932475000
C	-3.842983000	4.295317000	-0.305263000
C	-1.110173000	3.541530000	-0.087795000
H	-0.562765000	2.356250000	2.295536000
H	-2.087415000	2.096330000	4.208778000
C	-1.577959000	4.243607000	-1.218283000
C	-2.972579000	4.553656000	-1.331754000
H	-3.295313000	5.062267000	-2.235898000
H	3.796680000	-0.989546000	0.786511000
H	3.605139000	-0.630941000	-1.687675000
H	-5.227921000	1.851038000	-1.404385000

C	-3.612017000	-1.386723000	1.553269000
C	-4.256115000	-2.422065000	2.328176000
C	-4.230071000	-0.153265000	1.458933000
C	-3.720578000	-3.723070000	2.566505000
C	-5.533527000	-2.135631000	2.931928000
C	-5.479013000	0.121622000	2.059347000
H	-3.746709000	0.637785000	0.895624000
C	-4.387786000	-4.666206000	3.319289000
H	-2.756771000	-3.990897000	2.159412000
C	-6.199007000	-3.132666000	3.695305000
C	-6.124604000	-0.855350000	2.772031000
H	-5.912659000	1.109207000	1.946029000
C	-5.646820000	-4.376794000	3.889025000
H	-3.935903000	-5.642433000	3.474440000
H	-7.162778000	-2.883646000	4.133541000
H	-7.091692000	-0.665754000	3.232023000
H	-6.166611000	-5.127415000	4.477850000
O	-0.763144000	4.675719000	-2.172938000
H	0.927897000	0.828827000	-2.694289000
H	1.642406000	2.369106000	-2.501040000
H	-0.136607000	2.250550000	-2.467137000
Mg	1.083124000	4.710761000	-1.720894000
Br	3.027700000	5.339937000	-2.950326000

20

COD, $E_{\text{(SCF Done)}}$ = -311.853468 a.u.

C	-1.923188000	-0.009807000	-0.030235000
C	1.923176000	0.009780000	-0.030276000
C	-1.087032000	-1.106175000	0.665009000
C	1.213735000	-1.239647000	-0.492543000
C	-0.009589000	-1.706848000	-0.213195000
H	-0.673784000	-0.721482000	1.600743000
H	2.747446000	-0.281867000	0.640822000
H	1.820530000	-1.849675000	-1.164156000
H	2.416503000	0.454072000	-0.907712000
H	-1.777769000	-1.906990000	0.957634000
H	-0.291273000	-2.640577000	-0.701177000
C	1.087116000	1.106268000	0.664973000
C	0.009519000	1.706823000	-0.213181000
H	0.291208000	2.640537000	-0.701190000
C	-1.213770000	1.239591000	-0.492593000
H	-1.820561000	1.849571000	-1.164251000
H	-2.416673000	-0.454167000	-0.907545000
H	-2.747400000	0.281883000	0.640931000

H	0.674055000	0.721681000	1.600818000
H	1.777917000	1.907110000	0.957329000

74

Ni(**L1**)(cod), ***E*****(SCF Done) = -1635.087684 a.u.**

C	6.200536000	-2.598006000	-2.386409000
C	5.088313000	-1.900096000	-1.974968000
C	4.730316000	-1.845876000	-0.598821000
C	5.549967000	-2.532945000	0.355750000
C	6.691852000	-3.245584000	-0.102547000
C	7.010641000	-3.277968000	-1.440964000
H	2.969537000	-0.622519000	-0.871856000
H	6.463644000	-2.631346000	-3.440222000
H	4.465596000	-1.377971000	-2.697680000
C	3.592897000	-1.134682000	-0.141591000
C	5.184518000	-2.471245000	1.724350000
H	7.311026000	-3.765461000	0.624973000
H	7.886078000	-3.825329000	-1.779882000
C	4.070949000	-1.769421000	2.134091000
C	3.251394000	-1.084080000	1.198560000
H	5.799539000	-2.990553000	2.455822000
H	3.823642000	-1.742984000	3.190156000
C	2.022941000	-0.274263000	1.620810000
H	2.168841000	0.761311000	1.295022000
C	1.732558000	-0.254033000	3.126612000
H	0.853687000	0.370639000	3.310539000
H	1.526604000	-1.256834000	3.517541000
H	2.576624000	0.166707000	3.683499000
C	0.010265000	0.177744000	0.208132000
C	-0.799679000	-1.971965000	0.112215000
C	0.347210000	-2.008791000	0.830831000
H	-1.491861000	-2.756275000	-0.152591000
H	0.863014000	-2.836626000	1.292269000
N	0.832218000	-0.702986000	0.876112000
N	-0.995469000	-0.643799000	-0.256204000
C	-7.756148000	-2.828493000	-0.704567000
C	-6.553007000	-2.501813000	-1.291468000
C	-5.604680000	-1.695256000	-0.607567000
C	-5.918808000	-1.224153000	0.707026000
C	-7.166866000	-1.577016000	1.284632000
C	-8.066294000	-2.361658000	0.595943000
H	-4.139061000	-1.705716000	-2.189263000
H	-8.472884000	-3.446129000	-1.239061000
H	-6.313407000	-2.858646000	-2.290672000

C	-4.353764000	-1.342306000	-1.188251000
C	-4.962570000	-0.416041000	1.383239000
H	-7.401989000	-1.216326000	2.283370000
H	-9.018353000	-2.625052000	1.048750000
C	-3.764420000	-0.095706000	0.793939000
C	-3.440286000	-0.559754000	-0.514580000
H	-5.196983000	-0.051015000	2.380617000
H	-3.040377000	0.520012000	1.321932000
C	-2.092946000	-0.150099000	-1.100184000
H	-2.008135000	0.942233000	-1.016492000
C	-1.862668000	-0.532645000	-2.566058000
H	-0.887997000	-0.152530000	-2.885885000
H	-1.868181000	-1.618904000	-2.709343000
H	-2.633827000	-0.095122000	-3.208875000
Ni	0.122655000	2.073061000	-0.089139000
C	0.014311000	4.416749000	-1.943104000
C	1.628671000	3.903619000	1.520109000
C	-0.435077000	5.016957000	-0.584681000
C	0.167376000	3.507348000	1.431395000
C	-0.720309000	3.954695000	0.463973000
H	0.318189000	5.721869000	-0.217533000
H	1.746987000	4.860466000	2.055522000
H	-0.235510000	3.019532000	2.317397000
H	2.141209000	3.155533000	2.138934000
H	-1.340355000	5.615111000	-0.751241000
H	-1.774048000	3.717332000	0.618733000
C	2.367667000	4.013956000	0.147896000
C	1.914637000	3.006248000	-0.895845000
H	2.571976000	2.152599000	-1.051523000
C	0.903157000	3.198047000	-1.810083000
H	0.833234000	2.479258000	-2.627215000
H	-0.879461000	4.115013000	-2.505247000
H	0.497470000	5.199427000	-2.552420000
H	2.256234000	5.028763000	-0.247028000
H	3.441799000	3.882514000	0.328641000

107

TS1''', $E_{(\text{SCF Done})}$ = -2479.224458 a.u., IF = -318.34 cm^{-1}

Ni	-0.110400000	0.215520000	0.612941000
O	-0.315296000	1.308688000	-1.065964000
C	1.389941000	0.832636000	-0.565447000
C	-8.128687000	-2.411360000	0.372650000
C	-6.773440000	-2.530642000	0.155618000
C	-6.064382000	-1.543231000	-0.579757000

C	-6.783589000	-0.416908000	-1.090863000
C	-8.179781000	-0.321184000	-0.850696000
C	-8.839437000	-1.296442000	-0.135505000
H	-4.142153000	-2.501573000	-0.410787000
H	-8.658518000	-3.173937000	0.937089000
H	-6.226742000	-3.385637000	0.546918000
C	-4.665184000	-1.643406000	-0.821529000
C	-6.060553000	0.568242000	-1.816307000
H	-8.720815000	0.537665000	-1.241106000
H	-9.908037000	-1.213374000	0.042858000
C	-4.710081000	0.438657000	-2.033295000
C	-3.981758000	-0.685327000	-1.542089000
H	-6.593166000	1.436573000	-2.197160000
H	-4.182360000	1.212451000	-2.585077000
C	-2.484684000	-0.727502000	-1.841578000
H	-2.026389000	0.182926000	-1.454033000
C	-2.188056000	-0.784083000	-3.352571000
H	-1.106967000	-0.752598000	-3.517679000
H	-2.595080000	-1.689889000	-3.815483000
H	-2.633763000	0.075766000	-3.859927000
C	-0.811737000	-1.633331000	-0.192343000
C	-1.099976000	-3.855540000	-0.671933000
C	-1.948693000	-3.159911000	-1.465349000
H	-0.945162000	-4.918448000	-0.580722000
H	-2.657702000	-3.501261000	-2.202668000
N	-1.769961000	-1.817618000	-1.156961000
N	-0.411404000	-2.918827000	0.090519000
C	6.845586000	-3.464692000	1.019167000
C	5.589101000	-3.156040000	1.489612000
C	4.428535000	-3.515216000	0.751358000
C	4.587204000	-4.201334000	-0.496106000
C	5.897789000	-4.504740000	-0.954393000
C	7.001931000	-4.146227000	-0.214045000
H	3.010804000	-2.678981000	2.148023000
H	7.725050000	-3.183818000	1.592044000
H	5.467594000	-2.629542000	2.433203000
C	3.118498000	-3.210769000	1.204311000
C	3.420856000	-4.544520000	-1.229622000
H	6.013994000	-5.025171000	-1.902241000
H	7.999583000	-4.382903000	-0.573660000
C	2.164107000	-4.239041000	-0.756503000
C	1.994689000	-3.561262000	0.481502000
H	3.532780000	-5.057035000	-2.182327000

H	1.296226000	-4.504540000	-1.351498000
C	0.621924000	-3.264545000	1.083671000
H	0.712630000	-2.366339000	1.692831000
C	0.151615000	-4.424022000	1.980933000
H	-0.830841000	-4.210122000	2.414873000
H	0.096405000	-5.370266000	1.431895000
H	0.869931000	-4.563504000	2.794102000
C	5.358862000	3.474412000	-1.666159000
C	4.039901000	3.439670000	-1.262225000
C	3.362940000	2.209494000	-1.023894000
C	4.078196000	1.003616000	-1.347036000
C	5.441925000	1.071908000	-1.732300000
C	6.085541000	2.281296000	-1.874466000
H	5.839378000	4.435285000	-1.833372000
H	3.506202000	4.372924000	-1.145935000
C	1.992085000	2.106750000	-0.589520000
C	3.379937000	-0.234107000	-1.384321000
H	5.964774000	0.140552000	-1.939455000
H	7.129298000	2.320653000	-2.174320000
C	2.041443000	-0.312566000	-1.085626000
H	3.917403000	-1.130718000	-1.684389000
H	1.518036000	-1.254318000	-1.172679000
C	1.044545000	7.236429000	0.837787000
C	-0.059605000	6.720705000	0.196363000
C	-0.105064000	5.363564000	-0.219353000
C	1.050890000	4.527704000	-0.027297000
C	2.147700000	5.079475000	0.692795000
C	2.146650000	6.393934000	1.111155000
H	-2.154684000	5.455902000	-0.914228000
H	1.059668000	8.274513000	1.158574000
H	-0.933186000	7.344437000	0.018315000
C	-1.295326000	4.806103000	-0.765929000
C	0.989199000	3.164840000	-0.469162000
H	2.987819000	4.440202000	0.940311000
H	2.996808000	6.781038000	1.667015000
C	-0.254721000	2.640212000	-0.853619000
C	-1.388333000	3.463258000	-1.047939000
H	-2.312897000	3.022021000	-1.408387000
C	-0.508280000	2.464235000	2.719963000
C	-1.973931000	-1.080575000	3.055641000
C	-1.909933000	2.083191000	3.242096000
C	-2.725860000	-0.085298000	2.201156000
C	-2.707611000	1.252723000	2.272117000

H	-1.828126000	1.569537000	4.204207000
H	-2.587999000	-1.355525000	3.928752000
H	-3.371256000	-0.532464000	1.447314000
H	-1.884274000	-1.999078000	2.464193000
H	-2.452158000	3.015842000	3.445219000
H	-3.307790000	1.804480000	1.548423000
C	-0.560221000	-0.695833000	3.563839000
C	0.402853000	-0.014637000	2.587591000
H	1.381320000	-0.495233000	2.533634000
C	0.415229000	1.351516000	2.244181000
H	1.386357000	1.745179000	1.953444000
H	-0.624555000	3.189239000	1.907264000
H	0.013822000	3.012525000	3.521863000
H	-0.651696000	-0.077891000	4.464879000
H	-0.090969000	-1.626165000	3.911995000

127

Ni(L1)$_2$(cod), $E_{(SCF\ Done)}$ = -2787.355285 a.u.

Ni	0.342758000	0.268446000	1.270710000
C	6.291795000	2.700429000	-0.976530000
C	7.539172000	3.364197000	-0.818662000
C	7.870169000	3.963996000	0.375095000
H	3.441028000	1.988066000	0.809302000
H	7.242467000	4.405172000	2.406172000
H	5.058955000	3.264080000	2.185218000
C	4.135067000	2.002613000	-0.028148000
C	5.909039000	2.066657000	-2.186628000
H	8.228994000	3.389831000	-1.659212000
H	8.826350000	4.468579000	0.484742000
C	4.691071000	1.430413000	-2.304229000
C	3.777445000	1.389867000	-1.216568000
H	6.594546000	2.089614000	-3.030908000
H	4.434667000	0.950506000	-3.243174000
C	2.416152000	0.697868000	-1.310664000
H	2.291488000	0.055874000	-0.432702000
C	2.204869000	-0.163116000	-2.560580000
H	1.240351000	-0.673027000	-2.487429000
H	2.205822000	0.434429000	-3.479302000
H	2.987350000	-0.924428000	-2.641735000
C	0.349855000	1.580332000	-0.196910000
C	0.059485000	3.432622000	-1.528239000
C	1.167251000	2.793747000	-1.971451000
H	-0.395453000	4.351362000	-1.862426000
H	1.860699000	3.048832000	-2.757819000

N	1.325485000	1.674848000	-1.164208000
N	-0.436253000	2.684965000	-0.460694000
C	-5.861721000	2.300375000	-4.262609000
C	-4.617377000	2.540758000	-3.722868000
C	-4.414193000	2.510295000	-2.315679000
C	-5.530152000	2.223464000	-1.464823000
C	-6.800454000	1.977057000	-2.051860000
C	-6.964441000	2.014796000	-3.419403000
H	-2.303613000	2.947346000	-2.396311000
H	-6.002413000	2.328633000	-5.339835000
H	-3.770086000	2.758900000	-4.369131000
C	-3.139450000	2.749328000	-1.731329000
C	-5.315978000	2.188026000	-0.060827000
H	-7.642639000	1.754883000	-1.400909000
H	-7.941415000	1.827784000	-3.857024000
C	-4.069873000	2.424740000	0.469789000
C	-2.951642000	2.710490000	-0.364543000
H	-6.153771000	1.957185000	0.592624000
H	-3.921051000	2.382025000	1.545237000
C	-1.625678000	3.039033000	0.321895000
H	-1.547133000	2.430477000	1.227243000
C	-1.602925000	4.525752000	0.727042000
H	-0.657536000	4.781843000	1.214385000
H	-1.745332000	5.181910000	-0.138657000
H	-2.419884000	4.726317000	1.426908000
C	6.968108000	3.927981000	1.469291000
C	7.246192000	-2.614194000	-2.103176000
C	6.199878000	-2.109616000	-1.363091000
C	5.214848000	-2.977048000	-0.815837000
C	5.330844000	-4.386346000	-1.043003000
C	6.422343000	-4.874261000	-1.810410000
C	7.359081000	-4.008621000	-2.329647000
H	4.055259000	-1.420740000	0.129164000
H	7.992607000	-1.941336000	-2.516741000
H	6.111911000	-1.039868000	-1.192073000
C	4.121203000	-2.491711000	-0.048447000
C	4.350159000	-5.242936000	-0.476644000
H	6.506561000	-5.945310000	-1.979743000
H	8.190489000	-4.392432000	-2.914813000
C	3.307621000	-4.736874000	0.265037000
C	3.164417000	-3.336023000	0.480880000
H	4.440681000	-6.316134000	-0.628610000
H	2.594533000	-5.426625000	0.707115000

C	2.016322000	-2.761236000	1.306693000
H	2.260778000	-1.727939000	1.557584000
C	1.770335000	-3.530086000	2.613509000
H	0.998158000	-3.022009000	3.196517000
H	1.436225000	-4.556645000	2.428269000
H	2.689860000	-3.571916000	3.207045000
C	-0.044701000	-1.524720000	0.532036000
C	-1.017864000	-3.268269000	-0.609585000
C	0.174608000	-3.710743000	-0.149568000
H	-1.785114000	-3.768892000	-1.179431000
H	0.655023000	-4.668018000	-0.266989000
N	0.758889000	-2.649973000	0.536254000
N	-1.139807000	-1.950167000	-0.186114000
C	-8.355736000	-2.541174000	-0.879192000
C	-7.156266000	-2.017582000	-1.309721000
C	-5.984656000	-2.132870000	-0.513218000
C	-6.071282000	-2.800165000	0.749726000
C	-7.321511000	-3.331153000	1.164832000
C	-8.439289000	-3.206028000	0.369253000
H	-4.700271000	-1.100922000	-1.895219000
H	-9.244755000	-2.445956000	-1.497117000
H	-7.090201000	-1.500048000	-2.263885000
C	-4.733357000	-1.604356000	-0.934594000
C	-4.893166000	-2.900460000	1.538090000
H	-7.382020000	-3.838549000	2.125023000
H	-9.390867000	-3.615603000	0.697578000
C	-3.700916000	-2.372439000	1.103195000
C	-3.599464000	-1.707052000	-0.153667000
H	-4.946541000	-3.405414000	2.500015000
H	-2.813776000	-2.466313000	1.720980000
C	-2.265148000	-1.084956000	-0.567842000
H	-2.106465000	-0.171842000	0.016190000
C	-2.156481000	-0.727779000	-2.055745000
H	-1.167732000	-0.302320000	-2.247348000
H	-2.288279000	-1.611260000	-2.690747000
H	-2.901536000	0.018774000	-2.338245000
C	5.379033000	2.664491000	0.128372000
C	5.752863000	3.293733000	1.348581000
C	0.653800000	2.678770000	3.356094000
C	-1.799368000	-0.193994000	3.860391000
C	-0.385247000	2.693548000	4.499858000
C	-2.369732000	1.199334000	3.833795000
C	-1.807896000	2.386618000	4.095075000

H	-0.047911000	2.017587000	5.291219000
H	-2.416033000	-0.778239000	4.564696000
H	-3.427856000	1.212836000	3.560280000
H	-1.985973000	-0.642460000	2.874505000
H	-0.374335000	3.699113000	4.941025000
H	-2.455324000	3.260203000	3.995545000
C	-0.321134000	-0.435729000	4.210407000
C	0.757020000	0.000362000	3.217892000
H	1.618112000	-0.669614000	3.289237000
C	1.092253000	1.326164000	2.788522000
H	2.142994000	1.396428000	2.483870000
H	0.308515000	3.348951000	2.557335000
H	1.553961000	3.165992000	3.764870000
H	-0.121162000	-0.045420000	5.220714000
H	-0.228518000	-1.526333000	4.313900000

54

NiL2, $E_{(\text{SCF Done})}$ = -1323.165042 a.u.

C	6.758546000	-0.317157000	0.562179000
C	5.424138000	-0.630969000	0.419164000
C	4.472166000	0.337979000	-0.012461000
C	4.956496000	1.659692000	-0.305918000
C	6.338099000	1.950075000	-0.143973000
C	7.224842000	0.987699000	0.280711000
H	7.458729000	-1.080058000	0.892061000
H	5.107043000	-1.646391000	0.632436000
C	3.070546000	0.063872000	-0.171844000
C	4.049170000	2.654990000	-0.756032000
H	6.683633000	2.956688000	-0.368234000
H	8.278789000	1.224376000	0.397889000
C	2.717075000	2.360087000	-0.915857000
C	2.232111000	1.063975000	-0.622915000
H	4.429976000	3.649835000	-0.974884000
H	2.023084000	3.119029000	-1.266857000
C	2.528903000	-1.339145000	0.117784000
H	3.127726000	-1.769219000	0.925802000
C	2.650918000	-2.272471000	-1.094941000
H	2.336255000	-3.288409000	-0.833755000
H	2.014331000	-1.917256000	-1.912764000
H	3.692604000	-2.294308000	-1.431371000
C	-0.034674000	-1.316502000	-0.010192000
C	-0.390015000	-1.276801000	2.248866000
C	0.949284000	-1.294165000	2.049381000
H	-0.958241000	-1.274377000	3.165035000

H	1.765282000	-1.296121000	2.755918000
N	1.162695000	-1.325416000	0.676067000
N	-0.982195000	-1.287330000	0.992320000
C	-6.114849000	-0.310543000	-1.386058000
C	-4.932575000	-0.594874000	-0.736943000
C	-4.276990000	0.376372000	0.073398000
C	-4.884035000	1.674175000	0.180460000
C	-6.105875000	1.932129000	-0.497610000
C	-6.714455000	0.963970000	-1.262659000
H	-6.587682000	-1.071528000	-2.001026000
H	-4.495056000	-1.579300000	-0.861267000
C	-3.043674000	0.124543000	0.768647000
C	-4.250581000	2.683204000	0.953712000
H	-6.551453000	2.919656000	-0.401918000
H	-7.647768000	1.176342000	-1.776903000
C	-3.060582000	2.425792000	1.588370000
C	-2.462466000	1.146721000	1.492664000
H	-4.720492000	3.661144000	1.025823000
H	-2.568120000	3.200384000	2.170028000
C	-2.425549000	-1.273182000	0.714884000
H	-2.474766000	-1.634678000	-0.318536000
C	-3.159383000	-2.264505000	1.635992000
H	-2.718701000	-3.263633000	1.551966000
H	-3.119316000	-1.947876000	2.683919000
H	-4.214863000	-2.322793000	1.357754000
Ni	-0.388684000	-1.291089000	-1.761970000
H	1.176917000	0.849804000	-0.766324000
H	-1.517789000	0.978621000	1.998823000

74

Ni(L2)(cod), ***E*****(SCF Done) = -1635.084322 a.u.**

Ni	-0.034286000	0.829869000	0.002320000
C	5.663672000	1.192842000	1.602994000
C	4.580280000	0.366355000	1.393693000
C	4.464692000	-0.426212000	0.215105000
C	5.517608000	-0.329541000	-0.757888000
C	6.622434000	0.529004000	-0.508725000
C	6.700607000	1.274508000	0.645072000
H	5.720249000	1.787186000	2.510973000
H	3.800714000	0.330423000	2.146678000
C	3.351823000	-1.297519000	-0.046441000
C	5.436311000	-1.087178000	-1.956474000
H	7.410268000	0.587215000	-1.256297000
H	7.551132000	1.927392000	0.820855000

C	4.355402000	-1.901556000	-2.189861000
C	3.317153000	-2.001990000	-1.233595000
H	6.239924000	-1.005071000	-2.684374000
H	4.287289000	-2.474397000	-3.110804000
C	2.245230000	-1.457952000	1.000255000
H	1.985848000	-0.466272000	1.386073000
C	2.699302000	-2.345730000	2.174318000
H	1.900640000	-2.437344000	2.918082000
H	2.981045000	-3.347632000	1.833370000
H	3.579157000	-1.912635000	2.656812000
C	-0.001549000	-1.082293000	-0.003060000
C	-0.608951000	-3.281169000	-0.299863000
C	0.624132000	-3.280030000	0.260586000
H	-1.236496000	-4.107723000	-0.592704000
H	1.258903000	-4.105512000	0.540512000
N	0.983189000	-1.945751000	0.440833000
N	-0.978832000	-1.947766000	-0.460567000
C	-5.650503000	1.217600000	-1.564444000
C	-4.570699000	0.382618000	-1.371037000
C	-4.456792000	-0.429772000	-0.205820000
C	-5.507541000	-0.344541000	0.770590000
C	-6.608527000	0.523546000	0.537966000
C	-6.685109000	1.288550000	-0.603083000
H	-5.706188000	1.826934000	-2.462500000
H	-3.792929000	0.355191000	-2.126286000
C	-3.347738000	-1.310454000	0.039301000
C	-5.427951000	-1.123091000	1.955786000
H	-7.394718000	0.573117000	1.287887000
H	-7.532706000	1.948476000	-0.766285000
C	-4.350850000	-1.947005000	2.173217000
C	-3.314661000	-2.035952000	1.213721000
H	-6.229900000	-1.049542000	2.686434000
H	-4.284279000	-2.536049000	3.083995000
C	-2.244090000	-1.459021000	-1.011859000
H	-1.988230000	-0.463003000	-1.389635000
C	-2.700417000	-2.336018000	-2.192767000
H	-1.904641000	-2.418870000	-2.940681000
H	-2.979360000	-3.341708000	-1.860693000
H	-3.582847000	-1.899148000	-2.667298000
H	2.469685000	-2.644053000	-1.449708000
H	-2.469694000	-2.685501000	1.417191000
C	1.450354000	3.015749000	1.277910000
C	-1.433412000	3.089924000	-1.279706000

C	1.531942000	3.397133000	-0.230689000
C	-0.260541000	2.208432000	-1.654556000
C	1.023409000	2.314199000	-1.165715000
H	0.987765000	4.330441000	-0.407513000
H	-1.447730000	4.012390000	-1.884199000
H	-0.421620000	1.549593000	-2.507300000
H	-2.354640000	2.552011000	-1.536750000
H	2.580354000	3.612903000	-0.471249000
H	1.786330000	1.693230000	-1.633325000
C	-1.490327000	3.471577000	0.228324000
C	-1.037275000	2.356853000	1.154364000
H	-1.831493000	1.771213000	1.616353000
C	0.238406000	2.187141000	1.650257000
H	0.365459000	1.526996000	2.507354000
H	2.346674000	2.439778000	1.539447000
H	1.501811000	3.937813000	1.880869000
H	-0.896410000	4.373749000	0.406829000
H	-2.524813000	3.742648000	0.473822000

107

TS1''^{L2}, $E_{(SCF\ Done)}$ = -2479.201013 a.u., IF = -311.07 cm^{-1}

Ni	0.507694000	0.165082000	0.520125000
O	1.483749000	0.560971000	-1.209174000
C	1.410496000	-1.193834000	-0.625356000
C	-4.727106000	5.974646000	-1.071217000
C	-3.526226000	6.412899000	-0.563618000
C	-2.361179000	5.601118000	-0.617294000
C	-2.428958000	4.293424000	-1.216071000
C	-3.693548000	3.879649000	-1.721816000
C	-4.806589000	4.689893000	-1.653480000
H	-1.101509000	7.068478000	0.355548000
H	-5.609489000	6.606733000	-1.023006000
H	-3.447370000	7.396895000	-0.107197000
C	-1.131986000	6.075279000	-0.086127000
C	-1.233408000	3.491827000	-1.266316000
H	-3.798608000	2.896615000	-2.155942000
H	-5.754491000	4.334691000	-2.049176000
C	-0.072049000	4.002189000	-0.713951000
C	-0.008954000	5.288255000	-0.129699000
H	0.825506000	3.393202000	-0.721843000
C	-1.091164000	2.135280000	-1.970581000
H	-0.061863000	1.818782000	-1.816690000
C	-1.301922000	2.217775000	-3.490639000
H	-1.127754000	1.241367000	-3.955334000

H	-2.294237000	2.577003000	-3.777856000
H	-0.573192000	2.923239000	-3.900758000
C	-1.505505000	0.361629000	-0.228394000
C	-3.524626000	-0.392570000	-1.000340000
C	-3.092841000	0.561170000	-1.858742000
H	-4.400996000	-1.016925000	-1.038043000
H	-3.526476000	0.900887000	-2.784377000
N	-1.885070000	1.031747000	-1.365610000
N	-2.563484000	-0.493133000	-0.005238000
C	-6.662022000	-3.366328000	0.216829000
C	-5.479984000	-2.725262000	0.520459000
C	-4.240459000	-3.424610000	0.615263000
C	-4.284673000	-4.847736000	0.388950000
C	-5.519882000	-5.477675000	0.076152000
C	-6.689003000	-4.759767000	-0.012185000
H	-7.582327000	-2.791395000	0.158119000
H	-5.511739000	-1.659999000	0.698316000
C	-2.969722000	-2.816071000	0.923523000
C	-3.098555000	-5.621976000	0.482682000
H	-5.519416000	-6.551969000	-0.092448000
H	-7.625300000	-5.255803000	-0.251602000
C	-1.902658000	-5.025158000	0.791912000
C	-1.852825000	-3.630685000	1.011055000
H	-3.159863000	-6.693789000	0.310414000
H	-0.991255000	-5.610549000	0.871174000
C	-2.714095000	-1.327706000	1.213399000
H	-1.720974000	-1.284118000	1.657309000
C	-3.670827000	-0.647708000	2.212298000
H	-3.209945000	0.268998000	2.587346000
H	-4.628672000	-0.366330000	1.771105000
H	-3.865136000	-1.312740000	3.059972000
C	4.857318000	-4.511408000	-1.667502000
C	4.535240000	-3.223143000	-1.293199000
C	3.186805000	-2.825212000	-1.065318000
C	2.166539000	-3.795129000	-1.363052000
C	2.531384000	-5.118687000	-1.721831000
C	3.851831000	-5.485292000	-1.856598000
H	5.900035000	-4.774562000	-1.826834000
H	5.329887000	-2.497265000	-1.191060000
C	2.786192000	-1.498568000	-0.666625000
C	0.805897000	-3.387002000	-1.402114000
H	1.737325000	-5.836608000	-1.917083000
H	4.118347000	-6.501002000	-2.135931000

C	0.433693000	-2.094613000	-1.118901000
H	0.049346000	-4.113030000	-1.691619000
H	-0.602116000	-1.796410000	-1.206558000
C	7.605356000	0.620863000	0.557406000
C	6.855167000	1.541947000	-0.139517000
C	5.513542000	1.268034000	-0.515192000
C	4.947680000	-0.021417000	-0.220761000
C	5.733397000	-0.923948000	0.549823000
C	7.023618000	-0.613639000	0.927131000
H	5.156697000	3.226154000	-1.370345000
H	8.627366000	0.849556000	0.846911000
H	7.274747000	2.512894000	-0.394406000
C	4.706276000	2.266083000	-1.129533000
C	3.595585000	-0.281313000	-0.620235000
H	5.293197000	-1.862217000	0.869513000
H	7.593198000	-1.320955000	1.524472000
C	2.803543000	0.793819000	-1.054659000
C	3.368661000	2.053050000	-1.368952000
H	2.741819000	2.828289000	-1.798314000
H	-0.892224000	-3.182645000	1.243803000
H	0.932161000	5.641083000	0.282623000
C	2.766393000	1.194431000	2.476538000
C	-0.941035000	1.866332000	3.119935000
C	2.285300000	2.223504000	3.519839000
C	-0.048597000	3.042103000	2.825381000
C	1.268419000	3.205610000	2.991904000
H	1.922078000	1.705052000	4.412096000
H	-1.649601000	2.171507000	3.908355000
H	-1.554573000	1.706848000	2.222956000
H	3.166933000	2.790189000	3.845051000
C	-0.341202000	0.509894000	3.535899000
C	0.542297000	-0.258350000	2.551043000
C	1.845013000	0.032563000	2.118760000
H	3.076646000	1.730858000	1.571667000
H	3.688834000	0.737693000	2.863893000
H	0.186625000	0.621828000	4.491553000
H	-1.195780000	-0.138171000	3.768195000
H	-0.592755000	3.885736000	2.400336000
H	1.681389000	4.163441000	2.672027000
H	2.415017000	-0.836879000	1.804157000
H	0.310706000	-1.325773000	2.537181000

87

TS1′^{L2}, $E_{(SCF\ Done)}$ = -2167.351959 a.u., IF = -326.11 cm^{-1}

C	5.154980000	2.429013000	-1.799299000
C	3.908276000	2.549995000	-1.224266000
C	3.724334000	2.435536000	0.183311000
C	4.883331000	2.156271000	0.984582000
C	6.154475000	2.043659000	0.360749000
C	6.294853000	2.183717000	-1.000530000
H	5.260168000	2.516196000	-2.877266000
H	3.054409000	2.716314000	-1.871450000
C	2.446813000	2.569215000	0.831942000
C	4.743068000	1.985968000	2.387482000
H	7.019769000	1.836175000	0.985756000
H	7.273452000	2.094377000	-1.464075000
C	3.507394000	2.077846000	2.977191000
C	2.364253000	2.371287000	2.195917000
H	5.628639000	1.770342000	2.980175000
H	3.394851000	1.929623000	4.047634000
C	1.225084000	2.958057000	-0.003472000
H	1.189366000	2.311574000	-0.887141000
C	1.274535000	4.428678000	-0.462700000
H	0.404744000	4.662852000	-1.085638000
H	1.289763000	5.112431000	0.392428000
H	2.181374000	4.616536000	-1.041142000
C	-0.883989000	1.646305000	0.353201000
C	-1.792579000	2.920770000	2.009227000
C	-0.607085000	3.485571000	1.672434000
H	-2.520988000	3.220278000	2.744709000
H	-0.117953000	4.363074000	2.063085000
N	-0.060091000	2.698093000	0.668449000
N	-1.952004000	1.805049000	1.197492000
C	-6.415674000	-1.586265000	-0.212399000
C	-5.372801000	-0.787314000	0.205528000
C	-5.344991000	0.608480000	-0.079265000
C	-6.439508000	1.155408000	-0.832834000
C	-7.500330000	0.304014000	-1.244081000
C	-7.496126000	-1.037767000	-0.940551000
H	-6.405207000	-2.648364000	0.017330000
H	-4.552541000	-1.245706000	0.745387000
C	-4.280493000	1.482370000	0.336673000
C	-6.444053000	2.536233000	-1.164569000
H	-8.320280000	0.738013000	-1.811512000
H	-8.313322000	-1.676885000	-1.263077000
C	-5.406300000	3.345645000	-0.774906000
C	-4.329750000	2.814410000	-0.025593000

H	-7.277944000	2.935473000	-1.736710000
H	-5.401730000	4.400150000	-1.036813000
C	-3.137046000	0.929817000	1.195263000
H	-2.778049000	-0.002533000	0.753393000
C	-3.581997000	0.647815000	2.643396000
H	-2.745293000	0.256969000	3.231472000
H	-3.969418000	1.549156000	3.129754000
H	-4.386703000	-0.090231000	2.651817000
Ni	-0.514853000	0.343071000	-0.971389000
H	1.403620000	2.432521000	2.695019000
H	-3.524604000	3.481991000	0.261282000
C	0.648265000	-5.649307000	1.594535000
C	1.015645000	-4.508313000	0.908782000
C	0.053129000	-3.563600000	0.455179000
C	-1.332909000	-3.912325000	0.640615000
C	-1.675334000	-5.078217000	1.368732000
C	-0.708579000	-5.931013000	1.858147000
H	1.418183000	-6.341262000	1.927016000
H	2.062929000	-4.344881000	0.696076000
C	0.366792000	-2.349632000	-0.255693000
C	-2.350770000	-3.143261000	0.000215000
H	-2.730207000	-5.303717000	1.512884000
H	-0.987032000	-6.823596000	2.411685000
C	-2.048855000	-2.038368000	-0.752978000
C	-0.704028000	-1.575039000	-0.765013000
H	-3.383369000	-3.474953000	0.090887000
H	-2.820450000	-1.488422000	-1.281979000
C	5.775542000	-2.280669000	0.028391000
C	5.363304000	-1.857673000	-1.216168000
C	3.986612000	-1.697966000	-1.523691000
C	3.002253000	-2.049386000	-0.534751000
C	3.466542000	-2.418255000	0.756917000
C	4.813876000	-2.534277000	1.031986000
H	4.338515000	-0.902338000	-3.508080000
H	6.834178000	-2.389524000	0.248654000
H	6.094873000	-1.612787000	-1.983091000
C	3.576914000	-1.146829000	-2.771883000
C	1.614530000	-1.906763000	-0.874105000
H	2.742905000	-2.572884000	1.549292000
H	5.136667000	-2.808777000	2.033013000
C	1.295857000	-1.204544000	-2.048855000
C	2.254500000	-0.866778000	-3.025966000
H	1.931597000	-0.400656000	-3.951811000

O	-0.037017000	-0.949951000	-2.239079000
